# α‐Amidoaldehydes as Substrates in Rhodium‐Catalyzed Intermolecular Alkyne Hydroacylation: The Synthesis of α‐Amidoketones

**DOI:** 10.1002/chem.202002478

**Published:** 2020-07-30

**Authors:** Ritashree Pal, Sean C. O'Brien, Michael C. Willis

**Affiliations:** ^1^ Department of Chemistry Chemistry Research Laboratory University of Oxford Mansfield Road Oxford OX1 3TA United Kingdom

**Keywords:** aldehyde, alkyne, catalysis, hydroacylation, rhodium

## Abstract

We show that readily available α‐amidoaldehydes are effective substrates for intermolecular Rh‐catalyzed alkyne hydroacylation reactions. The catalyst [Rh(dppe)(C_6_H_5_F)][BAr^F^_4_] provides good reactivity, and allows a broad range of aldehydes and alkynes to be used as substrates, delivering α‐amidoketone products. High yields and high levels of regioselectivity are achieved. The use of α‐amidoaldehydes as substrates establishes that 1,4‐dicarbonyl motifs can be used as controlling groups in Rh‐catalyzed hydroacylation reactions.

## Graphical abstract

**Synthetically useful** α‐amidoketones can now be prepared in a single step from the corresponding α‐amidoaldehydes (see scheme). A rhodium‐catalyzed alkyne hydroacylation is used to join a broad range of aldehydes and alkynes in good yields and with high regiocontrol. A 6‐membered rhodacycle intermediate is proposed. The use of α‐amidoaldehydes as substrates in hydroacylation reactions adds to the repertoire of synthetically useful building blocks that can be accessed using these methods.

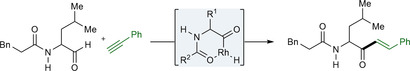

Transition‐metal‐catalyzed hydroacylation has emerged as a powerful and robust method to prepare ketones.1a Hydroacylation involves the addition of an aldehyde across the carbon–carbon π‐bond of an alkene or alkyne, to form a ketone or an enone, respectively (Scheme 1 a).^1^ Rhodium(I) complexes are the most commonly used catalysts, and the success of these reactions is often related to the stability of key metal‐acyl hydride intermediates, which are formed after oxidative addition of the aldehyde to the rhodium complex.^2^ In addition to productive hydroacylation, these metal‐acyl hydride intermediates can undergo reductive decarbonylation, which delivers deactivated rhodium complexes and reduced substrates. A common approach to limit decarbonylation is to use aldehydes containing additional coordinating groups that facilitate the formation of a stable chelated intermediate, which is usually a 5‐membered metallocycle. Chelating motifs featuring phosphines,^3^ alkenes,^4^ amines,^5^ hydroxyl groups,^6^ and sulfides ^[7]^ have been previously reported (Scheme 1 b). Although this strategy has proven effective, the installation and removal, or further transformation, of the chelating group is an inherent limitation. Recently our laboratory has demonstrated that simple carbonyl groups, such as ketones, esters and amides, when positioned β to the aldehyde group, can be used to mediate rhodium‐catalyzed hydroacylation reactions (Scheme 1 c).^8^ The ability to exploit carbonyl groups—arguably the most versatile functional group in synthetic chemistry—in this way, prompted us to investigate new applications of this concept, and in particular to target the preparation of synthetically valuable α‐amidoketones.

**Scheme 1:**
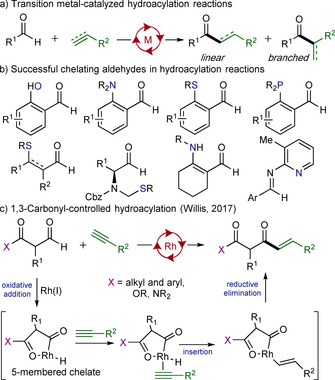
Intermolecular hydroacylation and chelating aldehydes.

α‐Amidoketones are present in many biologically relevant molecules,^9^ as well as being widely used as building blocks in organic synthesis (Scheme 2).^10^ This is exemplified by their use as precursors to oxazoles,^11^ thiazoles^12^ and imidazoles,^13^ which are among the most common heterocycles in a wide range of biological and medicinal applications.^14^ Unsurprisingly, considerable effort has been devoted to their synthesis; a selection of approaches is shown in Scheme 3.^15^ Many of these methods rely on the use of complex substrates, that are themselves synthetically challenging. In this Communication we report a mild, robust and atom economic method to prepare α‐amidoenones exploiting the Rh^I^‐catalyzed hydroacylation of alkynes. Importantly, the reaction uses readily available α‐amidoaldehydes as substrates, and demonstrates that, in the context of Rh‐catalyzed hydroacylation, the presumed 6‐membered metallocycles accessed from 1,4‐dicarbonyl compounds, are effective stabilizing motifs.

**Scheme 2:**
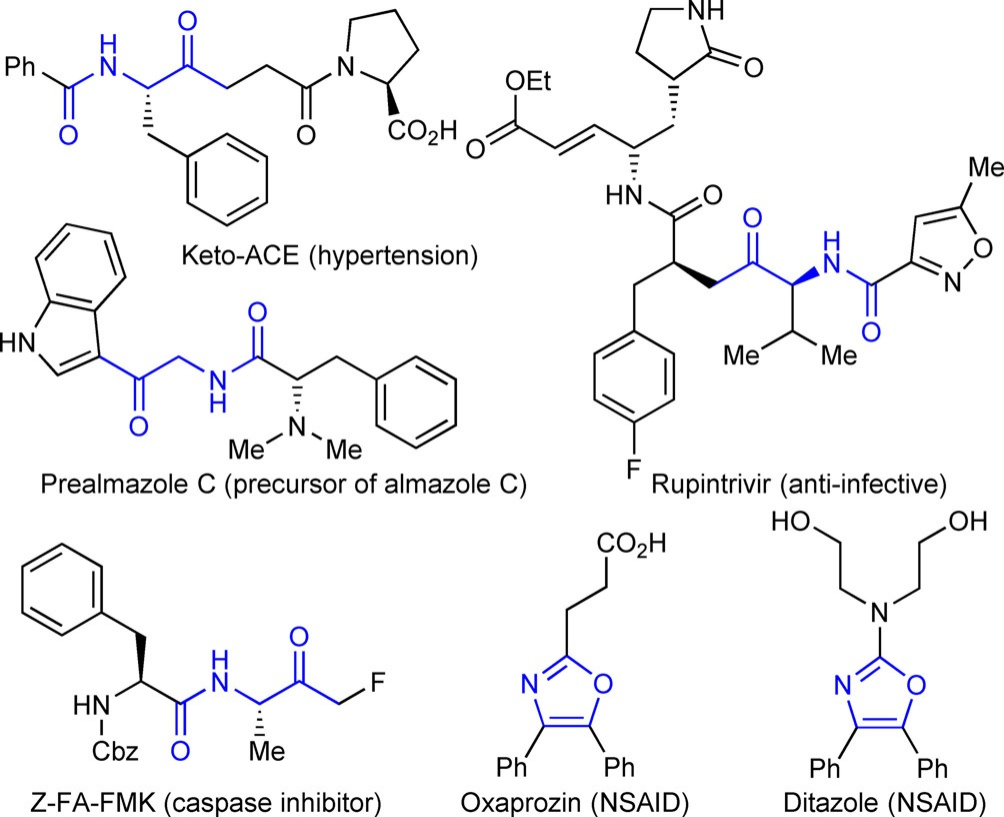
Examples of medicinally relevant compounds containing the α‐amidoketone or oxazole motif.

**Scheme 3:**
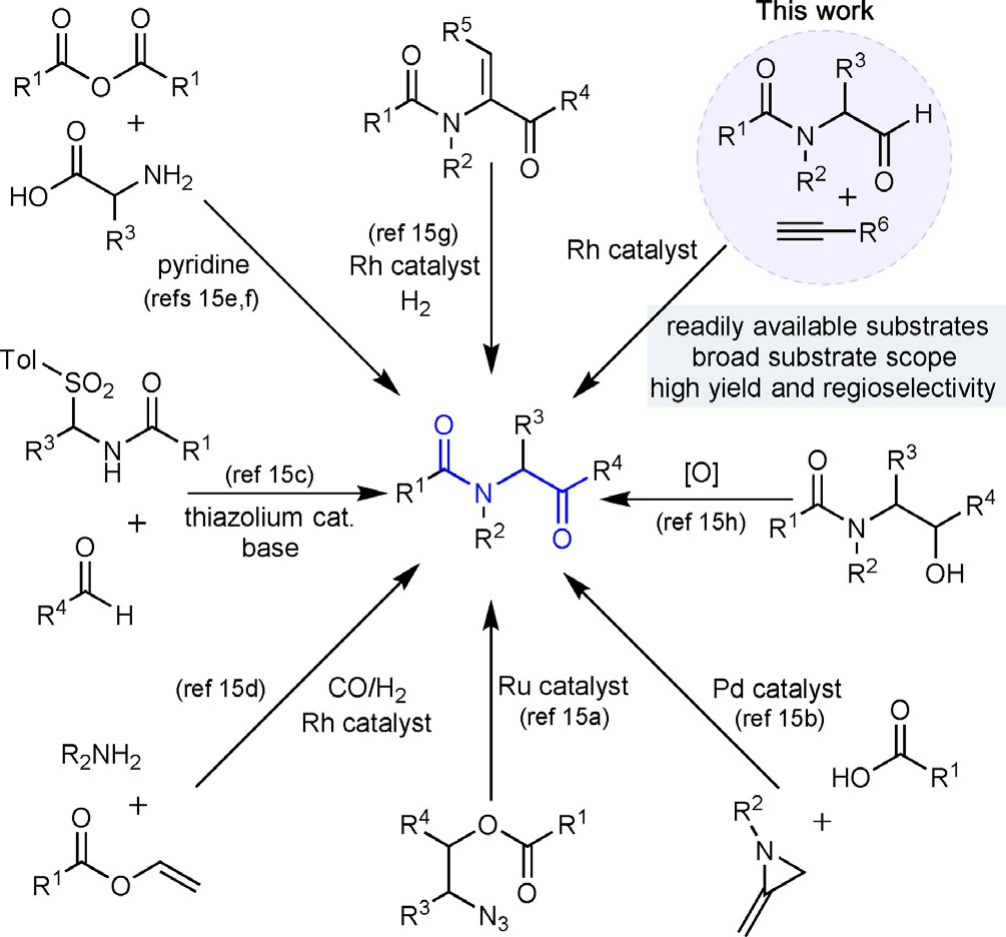
Synthetic routes to α‐amidoketone.

We began our study by investigating the addition of 1‐octyne to aldehyde **1 a** to give enone **2 a** using a variety of Rh‐catalysts (Table 1). Evaluation of a variety of commercial bisphosphines was undertaken, with a focus on ligands that had previously been shown to be effective in hydroacylation chemistry. Initial results revealed that the smallest bite‐angle ligand, dcpm, gave moderate conversion after 18 hours at 80 °C using 1,2‐dichloroethane (DCE) as solvent (entry 1).^16^ Using the larger bite‐angle ligand dcpe resulted in very low conversion (entry 2); however, exchanging the cyclohexyl groups for phenyl groups significantly improved reactivity, with dppe delivering a 93 % conversion (entry 3). Further increasing the bite angle (dppp) resulted in a lower conversion, but with increased regioselectivity (entry 4). Alternative wide bite angle ligands, such as BINAP, DPEphos and Xantphos were not effective (entries 5–7). Having established dppe as the most effective ligand for the reaction, we next evaluated a preformed catalyst [Rh(dppe)(C_6_H_5_F)][BAr^F^_4_],^17^ with the aim of achieving good reactivity with a decreased catalyst loading. Pleasingly, full conversion (80 % isolated yield) and increased regioselectivity (7:1 to 10:1) was achieved using 5 mol % catalyst (entry 8), and importantly, the reaction could be performed at 40 °C in CH_2_Cl_2_, compared to 80 °C needed in the original reaction. We attribute the higher conversions to an effective higher catalyst concentration when using the preformed complex, and the modest increase in regioselectivity is likely a function of the lower reaction temperature. Although we have not undertaken detailed mechanistic studies, by analogy to the 1,3‐dicarbonyl substrates we propose an irreversible alkyne insertion step that is product determining.8b

**Table 1:** Optimization studies of alkyne hydroacylation.^[a]^

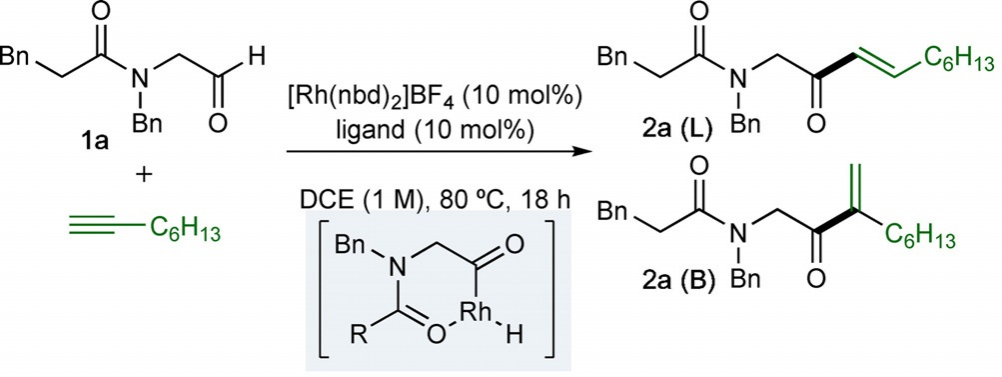			
Entry	Ligand	Conv. [%]^[b]^	**2 a** (L):**2 a** (B)
1	dcpm	42	9:1
2	dcpe	2	–
3	dppe	93	7:1
4	dppp	27	10:1
5	(±)‐BINAP	0	–
6	DPEphos	0	–
7	Xantphos	0	–
8^[c]^	dppe	80	10:1

[a] Reaction conditions: [Rh(nbd)_2_]BF_4_ (10 mol %), ligand (10 mol %), aldehyde **1 a** (0.2 mmol, 1.0 equiv), 1‐octyne (1.2 equiv), DCE (1.0 m), 80 °C for 18 h. [b] Determined by ^1^H NMR spectroscopic analysis of the crude reaction mixture. [c] [Rh(dppe)(C_6_H_5_F)][BAr^F^_4_] (5 mol %), CH_2_Cl_2_ (2.0 m), 40 °C, isolated yield and regioisomer ratio of **2 a**. 
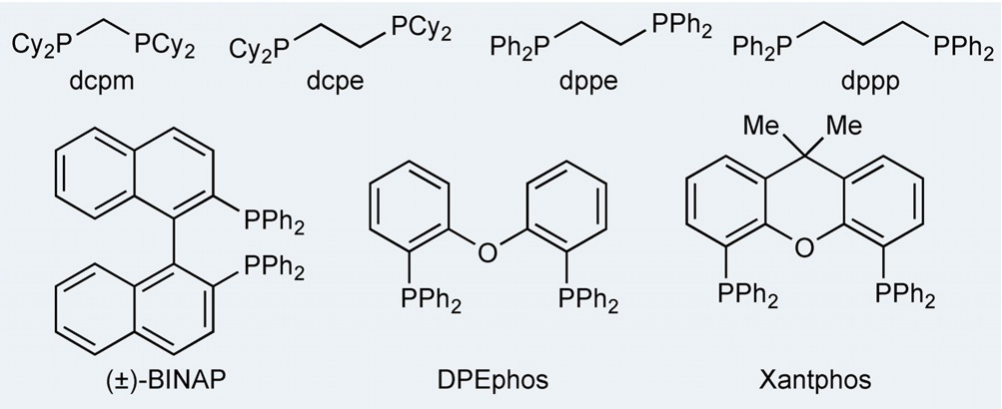

With the optimized reaction conditions in hand, the scope of this process was explored with respect to both the alkynes and aldehydes that can be employed (Scheme 4). First, aldehyde **1 a** and a range of terminal and internal alkynes were subjected to the developed conditions. A wide selection of cyclic and acyclic alkyl substituents was successfully incorporated, providing the hydroacylation adducts in good yields and regioselectivities (**2 a**–**e**). A cyclohexenyl substituted alkyne provided the corresponding linear enone product **2 f** in 84 % yield. Phenylacetylene and other arylalkynes, featuring both electron‐withdrawing and donating groups, delivered the desired products (**2 g**–**i**) in high yields with excellent linear selectivity. A thiophene‐substituted alkyne was also employed successfully, forming enone **2 j** in 89 % yield and a>20:1 linear:branched (L:B) ratio. Excellent isolated yields and selectivities were obtained when alkynes featuring sterically demanding substituents were used (**2 k**, **2 l**). Many functional groups were well tolerated in the reaction, including alkyl halides, alcohols, acetals and phthalimides (**2 m**–**p**). Symmetric internal alkynes showed mixed results under the optimized reaction conditions; diphenylacetylene gave the desired product **2 q** in 77 % yield, while 4‐octyne provided enone **2 r** in only 17 % yield. To demonstrate the scalability of this process, a gram‐scale reaction between aldehyde **1 a** and phenylacetylene, using 4 mol % of catalyst, was performed, and provided 1.03 grams of enone **2 g**, corresponding to a 73 % yield.

**Scheme 4:**
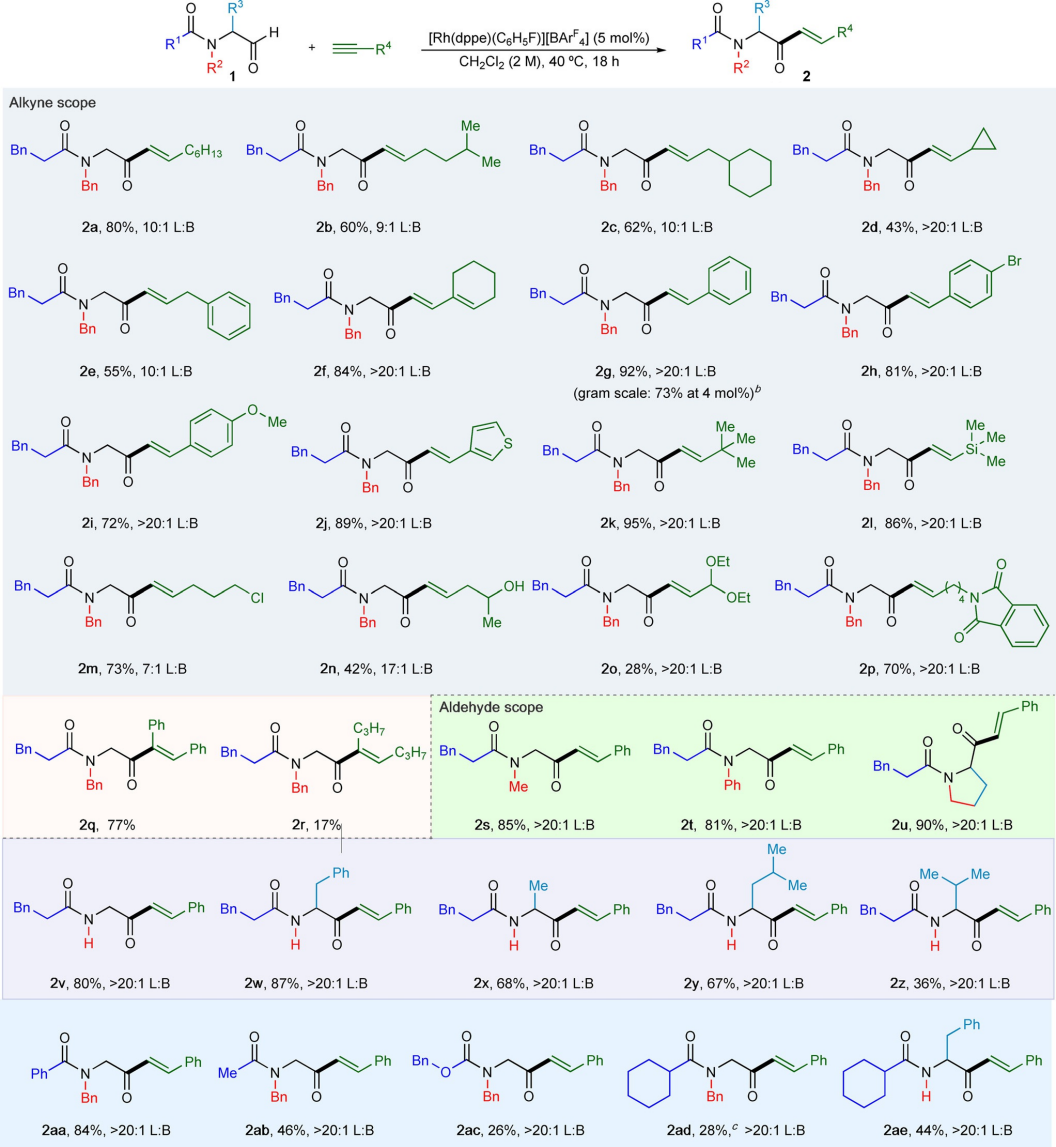
Scope of carbonyl‐controlled alkyne hydroacylation of α‐amidoaldehydes.^[a]^ [a] Reaction conditions: [Rh(dppe)(C_6_H_5_F)][BAr^F^_4_] (5 mol %), aldehyde (0.2 mmol, 1.0 equiv), alkyne (1.2 equiv), CH_2_Cl_2_ (2.0 m), 40 °C for 18 h. Linear to branched ratio (L:B) determined by ^1^H NMR spectroscopic analysis of the crude reaction mixture. [b] Performed with 3.7 mmol of aldehyde (1.04 g), using 4 mol % catalyst. 1.03 g of product isolated. [c] Performed with 10 mol % of [Rh(dppe)(C_6_H_5_F)][BAr^F^_4_].

Having established the robustness of the hydroacylation reaction towards the alkyne component, we next evaluated the scope of the aldehyde partner, in combination with phenylacetylene. Variation of the *N*‐substituent was well tolerated (**2 s**–**u**), and pleasingly an aldehyde featuring an N−H substituent also worked well, delivering adduct **2 v** in 80 % yield and >20:1 linear selectivity. Variation of the α‐substituent was then investigated, with benzyl, methyl and isobutyl substituted aldehydes reacting successfully to provide enones with excellent selectivities (**2 w**–**y**). However, when sterically larger substituents were present in the α‐position the yield of the product enone decreased, as seen in α‐isopropyl enone **2 z**. Variation of the acyl moiety of the amido group gave mixed results; a benzylamide gave the desired product in high yield and excellent regioselectivity (**2 aa**), however, an acetylamide resulted in deminished yields (**2 ab**). Use of a Cbz‐carbamate, in place of an amide, delivered the corresponding ketone (**2 ac**) in only 26 % yield, and likely reflects the less Lewis basic nature of the group. The sterically demanding cyclohexyl amide was also a poor substrate, providing enone **2 ad** in 28 % yield when 10 mol % of catalyst was used. Alleviating potential allylic strain by replacing the *N*‐Bn substituent with a N−H group led to improved yields, with cyclohexyl amide‐substituted enone **2 ae** now produced in 44 % yield.

To illustrate the synthetic utility of the products, we synthesised oxazole **3** from hydroacylation product **2 w** (Scheme 5). Cyclization in presence of POCl_3_ in toluene under reflux conditions gave oxazole **3** in excellent (92 %) yield.^18^

**Scheme 5:**
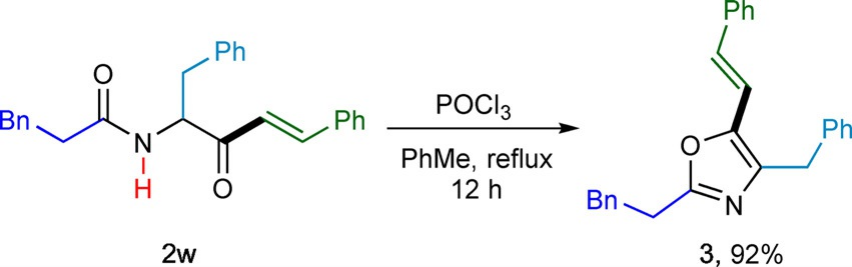
Synthesis of fully substituted oxazole. Reaction conditions: **2 w** (0.26 mmol), POCl_3_ (0.52 mmol) in toluene (2 mL) at reflux for 12 h.

In conclusion, we have developed the Rh^I^‐catalyzed hydroacylation of alkynes as a method to synthesize α‐amidoketones. The use of α‐amidoaldehydes as substrates establishes that 6‐membered rhodacyclic intermediates provide suitable chelating stability to enable productive hydroacylation. This robust method tolerates a wide range of functional groups to form enones in good to excellent yields with generally high levels of regiocontrol. The use of preformed catalyst [Rh(dppe)(C_6_H_5_F)][BAr^F^_4_] was found to be crucial to achieve high reactivity and regioselectivity.

## Conflict of interest

The authors declare no conflict of interest.

## Supporting information

As a service to our authors and readers, this journal provides supporting information supplied by the authors. Such materials are peer reviewed and may be re‐organized for online delivery, but are not copy‐edited or typeset. Technical support issues arising from supporting information (other than missing files) should be addressed to the authors.

Supplementary
