# Supplementary material for: α‐Amidoaldehydes as Substrates in Rhodium‐Catalyzed Intermolecular Alkyne Hydroacylation: The Synthesis of α‐Amidoketones
Source: Chemistry. 2020 Jul 30;26(51):11710–4. doi: 10.1002/chem.202002478 (PMC7540332; doi:10.1002/chem.202002478)

Chemistry–A European Journal

Supporting Information

α-Amidoaldehydes as Substrates in Rhodium-Catalyzed Intermolecular Alkyne Hydroacylation: The Synthesis of α-Amidoketones

Ritashree Pal, Sean C. O'Brien, and Michael C. Willis*[a]

Table of contents

1. **General experimental considerations** **S2**
2. **General procedures** **S3**
3. **Characterisation of compounds** **S4**
    - 3.1 Amide coupling products S4
    - 3.2 α-Amido aldehydes S11
    - 3.3 Hydroacylation products S19
    - 3.4 Oxazole synthesis S38
4. **References** **S38**
5. **NMR Spectra** **S39**

1. General experimental considerations

Reactions were performed under inert atmosphere of nitrogen gas with anhydrous solvent unless otherwise stated. All glassware was oven dried at >80 °C, and allowed to cool to room temperature under a positive argon pressure. Reactions were monitored by TLC until deemed complete using aluminum backed silica plates. Plates were visualized under ultraviolet light and/or by staining with vanillin or $KMnO_4$ stains.[1]

Reagents were purchased from Sigma-Aldrich Chemical Co. Ltd., Acros Organics Ltd., Alfa Aesar or Fluorochem Ltd. and used as supplied, with the exception of alkynes for use in rhodium-catalysed reactions, which were distilled before use. Dry DCM was obtained by passing through anhydrous alumina columns using the Innovative Technology Inc. PS-400-7 solvent purification system, followed by degassing. Petrol refers to the fraction of light petroleum ether, boiling in the range 40-60 °C. Flash chromatography was carried out using matrix 60 silica. $[Rh(dppe)(C_6H_5F)][BAr^F_4]$ (dppe = bis(diphenylphosphino)ethane) was prepared using literature method.[2]

^1H NMR spectra were obtained on a Bruker AVIII400 (400 MHz) spectrometer using the residual solvent as an internal standard. ^{13}C NMR spectra were obtained on a Bruker AVIII400 (100 MHz) spectrometer using the residual solvent as an internal standard. Chemical shifts were reported in parts per million (ppm) with the multiplicities of the spectra reported as following: s, singlet; d, doublet; t, triplet; q, quartet; hept, heptet; m, multiplet; br, broad; app, apparent and coupling constants in Hz, rounded to the nearest 0.5 Hz. Low resolution ESI mass spectra were recorded on a Waters LCT Premier spectrometer. High resolution ESI mass spectrometry measurements were recorded on a Brucker Daltronics microTOF (ESI) spectrometer by the internal service at the Department of Organic Chemistry, University of Oxford. Infra-red spectra were recorded as thin films on a Bruker Tensor 27 FT-IR spectrometer. Melting points were determined using a Stuart Scientific Melting Point Apparatus SMP1.

At 25 °C, tertiary amides give two sets of signals, owing to the presence of rotamers around the tertiary amide. At 90 °C coalescence occurs (example: ^1H-NMR of compound **2g** taken in DMSO-d_6). However, some signals were broad even at this temperature. Therefore, we preferred to report the spectra taken at 25 °C in $CDCl_3$.

2. General procedures

General procedure A for the preparation of amides from carboxylic acids and aminoalcohols:

Following the procedure by Willis *et al.*,[3] to a round bottomed flask containing the carboxylic acid (10.0 mmol, 1.0 equiv.) in CH_2Cl_2 (50 mL, 0.2 M) was added the amine (12.0 mmol, 1.2 equiv.) and EDCI.HCl (12.0 mmol, 1.2 equiv.). The reaction mixture was left to stir for 18 hours, followed by quenching with sat. aq. $NaHCO_3$ solution (50 mL). The layers were separated, and the organic layer was washed with brine (50 mL), dried over $MgSO_4$, filtered and concentrated in *vacuo*. The crude product was subjected to flash column chromatographical purification (FC) to afford the corresponding pure product.

General procedure B for the preparation of aldehydes from alcohols:

To a round bottomed flask containing anhydrous DMSO (15 mmol, 2.2 equiv.) in CH_2Cl_2 (35 mL) was added oxalyl chloride (7.5 mmol, 1.1 equiv.) at −78 °C over 30 min. The reaction mixture was stirred at this temperature for 15 min, then the alcohol (7 mmol, 1.0 equiv.) in CH_2Cl_2 (25 mL) was added over 30 min. The reaction mixture was stirred for a further 30 min before the addition of triethylamine (34 mmol, 4.8 equiv.) over 30 min. The reaction mixture was stirred for 15 min then allowed to warm to RT. Water (35 mL) was added and the product extracted with CH_2Cl_2. The combined organic layers were washed with brine, dried over $MgSO_4$, and the solvent removed in *vacuo*. The crude product was subjected to flash column chromatographical purification (FC) to afford the corresponding pure product.

General procedure C for the hydroacylation of alkynes:

To an oven dried reaction tube containing a magnetic stirrer were added $[Rh(dppe)(C_6H_5F)][BAr^F{}_4]$ (14.6 mg, 0.01 mmol, 5 mol%) and the corresponding aldehyde (0.20 mmol, 1 equiv). The tube was sealed, evacuated and backfilled with N_2 prior to dissolving in DCM (0.1 mL, 2 M). Next, the corresponding alkyne (0.24 mmol, 1.2 equiv.) was added and the reaction mixture was heated at 40 °C for 18 hours. The crude was directly charged onto silica gel and subjected to flash column chromatographical purification (FC) to afford the corresponding pure product.

3. Characterisation of compounds

3.1 Amide coupling products

***N*-benzyl-*N*-(2-hydroxyethyl)-3-phenylpropanamide, OH-1a**

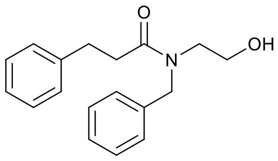

Following general procedure A, the product (2.25 g) was isolated by FC (petrol/EtOAc 1:2) in 79% yield as a colourless oil, starting from 3-phenylpropanoic acid (1.5 g, 10 mmol), 2-(benzylamino)ethan-1-ol (1.7 mL, 12 mmol) and EDCI.HCl (2.3 g, 12 mmol) in CH_2Cl_2 (50 mL); **^{1}H-NMR** (400 MHz, $CDCl_3$) (* denotes minor rotamer) [the compound exists as two rotamers, in a ratio of 3:1] δ 7.29–7.07 (m, 8H, 8 × Ar*H*), 7.04–6.97 (m, 2H, 2 × Ar*H*), 4.60* (s, 2H, NCH_2Ph), 4.42 (s, 2H, NCH_2Ph), 3.69–3.63 (m, 2H, CH_2CH_2OH), 3.58–3.52* (m, 2H, CH_2CH_2OH), 3.51–3.43 (m, 2H, C$H_2$$CH_2$OH), 3.29* (t, *J* = 5.5, 2H, C$H_2$$CH_2$OH), 3.19 (br s, 1H, O*H*), 3.01–2.89 (m, 2H, PhC$H_2$$CH_2$), 2.75–2.67* (m, 2H, Ph$CH_2CH_2$), 2.65–2.57 (m, 2H, Ph$CH_2CH_2$); **^{13}C-NMR** (100 MHz, $CDCl_3$) (* denotes minor rotamer) δ 175.1, 173.1*, 141.6*, 141.1, 137.9*, 136.4, 129.1, 128.8*, 128.7, 128.6, 128.0*, 127.9, 127.5*, 126.4, 126.4, 126.3*, 62.4, 60.4*, 52.7, 50.3, 49.1*, 49.0*, 35.3, 35.2*, 31.8*, 31.7; **IR** (film, cm^{-1}) 3396, 3028, 1622, 1452, 1421, 1210, 1075; **LRMS** *m/z* (ESI$^+$) 284.2 ([M+H]$^+$, 100%); **HRMS** (ESI$^+$) calc. for $C_{18}H_{22}O_2N$ [M+H]$^+$: 284.1645, found: 284.1646.

***N*-(2-hydroxyethyl)-*N*-methyl-3-phenylpropanamide, OH-1s**

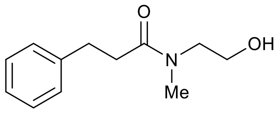

Following general procedure A, the product (1.57 g) was isolated by FC (petrol/EtOAc 1:2) in 76% yield as a colourless oil, starting from 3-phenylpropanoic acid (1.5 g, 10 mmol), 2-(methylamino)ethanol (0.96 mL, 12 mmol) and EDCI.HCl (2.3 g, 12 mmol) in CH_2Cl_2 (50 mL); **^{1}H-NMR** (400 MHz, $CDCl_3$) (* denotes minor rotamer) [the compound exists as two rotamers, in a ratio of 2.5:1] δ 7.33–7.25 (m, 2H, 2 × Ar*H*), 7.25–7.17 (m, 3H, 3 × Ar*H*), 3.79–3.73 (m, 2H, CH_2CH_2OH), 3.73–3.67* (m, 2H, CH_2CH_2OH), 3.55 (t, *J* = 5.5, 2H, C$H_2$$CH_2$OH), 3.40*

(t, J = 5.5, 2H, CH_2CH$_2$OH), 3.28 (s, 1H, CH$_2$CH$_2$OH), 3.01–2.93 (m, 5H, NCH_3, PhCH_2CH$_2$), 2.75–2.70* (m, 2H, PhCH$_2$CH_2), 2.68–2.60 (m, 2H, PhCH$_2$CH_2); **^{13}C-NMR** (100 MHz, CDCl$_3$) (* denotes minor rotamer) δ 174.3, 173.1*, 141.6*, 141.2, 128.6, 128.5*, 128.5, 126.3, 126.2*, 61.7, 59.9*, 51.9*, 51.6, 36.9, 35.6, 35.1*, 33.8*, 31.6*, 31.4; **IR** (film, cm^{-1}) 3379, 2935, 1620, 1494, 1454, 1405, 1075, 1050; **LRMS** *m/z* (ESI$^+$) 208.2 ([M+H]$^+$, 100%); **HRMS** (ESI$^+$) calc. for $C_{12}H_{18}O_2N$ [M+H]$^+$: 208.1332, found: 208.1334.

N-(2-hydroxyethyl)-*N*,3-diphenylpropanamide, OH-1t

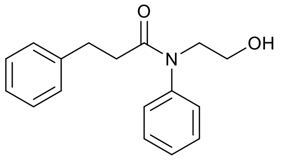

Following general procedure A, the product (2.11 g) was isolated by FC (petrol/EtOAc 1:2) in 78% yield as a colourless oil, starting from 3-phenylpropanoic acid (1.5 g, 10 mmol), *N*-(2-hydroxyethyl)aniline (1.5 mL, 12 mmol) and EDCI.HCl (2.3 g, 12 mmol) in CH_2Cl_2 (50 mL); **^{1}H-NMR** (400 MHz, CDCl$_3$) δ 7.41–7.30 (m, 3H, 3 × ArH), 7.28–7.21 (m, 2H, 2 × ArH), 7.21–7.15 (m, 1H, 1 × ArH), 7.09–6.99 (m, 4H, 4 × ArH), 3.89–3.80 (m, 2H, CH$_2$CH_2OH), 3.79–3.71 (m, 2H, CH_2CH$_2$OH), 3.18–3.07 (m, 1H, CH$_2$CH$_2$OH), 2.91 (t, J = 7.5, 2H, PhCH_2CH$_2$), 2.37 (t, J = 7.5, 1H, PhCH$_2$CH_2); **^{13}C-NMR** (100 MHz, CDCl$_3$) δ 174.6, 142.9, 141.1, 130.0, 128.6, 128.5, 128.3, 128.1, 126.3, 61.9, 53.2, 36.3, 31.9; **IR** (film, cm^{-1}) 3406, 2930, 1633, 1593, 1494, 1404, 1074; **LRMS** *m/z* (ESI$^+$) 270.5 ([M+H]$^+$, 100%); **HRMS** (ESI$^+$) calc. for $C_{17}H_{20}O_2N$ [M+H]$^+$: 270.1489, found: 270.1488.

1-(2-(hydroxymethyl)pyrrolidin-1-yl)-3-phenylpropan-1-one, OH-1u

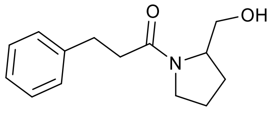

Following general procedure A, the product (1.83 g) was isolated by FC (petrol/EtOAc 1:1) in 79% yield as a white amorphous solid, starting from 3-phenylpropanoic acid (1.5 g, 10 mmol), (±)-2-pyrrolidinemethanol (1.1 mL, 12 mmol) and EDCI.HCl (2.3 g, 12 mmol) in CH_2Cl_2 (50 mL); **^{1}H-NMR** (400 MHz, CDCl$_3$) δ 7.35–7.27 (m, 2H, 2 × ArH), 7.26–7.19 (m, 3H, 3 × ArH), 5.07 (dd, J = 7.5, 2.5, 1H, CHCH$_2$OH), 4.29–4.16 (m, 1H, CHCH$_2$OH), 3.71–3.63 (m, 1H, CHCH_AH$_B$OH), 3.31 – 3.53 (m, 1H, CHCH$_A$$H_B$OH), 3.45–3.37 (m, 1H, NCH_AH$_B$CH$_2$CH$_2$), 3.35–3.28 (m, 1H, NCH$_A$$H_BCH_2CH_2$), 3.01 (t, J = 7.5, 2H, PhCH_2CH$_2$), 2.67–2.57 (m, 2H, PhCH$_2$CH_2), 2.07–1.95

(m, 1H, $NCH_2CH_2CH_AH_B$), 1.95–1.73 (m, 2H, $NCH_2CH_AH_BCH_2$, $NCH_2CH_AH_BCH_2$), 1.62–1.52 (m, 1H, $NCH_2CH_2CH_AH_B$); **^{13}C-NMR** (100 MHz, $CDCl_3$) δ 173.7, 141.1, 128.7, 128.6, 126.4, 67.6, 61.3, 48.2, 37.1, 31.3, 28.4, 24.5; **IR** (film, cm^{-1}) 3380, 2953, 2876, 1616, 1444, 1343, 1052; **LRMS** *m/z* (ESI^+) 234.2 ($[M+H]^+$, 100%); **HRMS** (ESI^+) calc. for $C_{14}H_{20}O_2N$ $[M+H]^+$: 234.14886, found 234.14884; **m.p.** (°C): 44–45.

N-(2,2-dimethoxyethyl)-3-phenylpropanamide, AC-1v

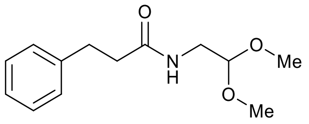

Following general procedure A, the product (4.37 g) was isolated by FC (100% EtOAc) in 77% yield as a yellow oil, starting from 3-phenylpropanoic acid (3 g, 20 mmol), 2,2-dimethoxyethan-1-amine (618 mg, 6 mmol) and EDCI.HCl (4.6 g, 24 mmol) in CH_2Cl_2 (100 mL); **^{1}H-NMR** (400 MHz, $CDCl_3$) δ 7.33–7.27 (m, 2H, 2 × Ar*H*), 7.25–7.17 (m, 3H, 3 × Ar*H*), 5.65 (s, 1H, N*H*), 4.30 (t, *J* = 5.0, 1H, $CH(OCH_3)_2$), 3.45–3.30 (m, 8H, $CH_2CH(OCH_3)_2$), 2.98 (t, *J* = 7.5, 2H, $PhCH_2CH_2$), 2.51 (t, *J* = 7.5, 2H, $PhCH_2CH_2$); **^{13}C-NMR** (100 MHz, $CDCl_3$) δ 172.3, 140.9, 128.6, 128.4, 126.3, 102.7, 54.5, 41.0, 38.4, 31.7; **IR** (film, cm^{-1}) 3304, 2980, 2833, 1646, 1545, 1496, 1453, 1384, 1128, 1058, 750, 699; **LRMS** *m/z* (ESI^+) 260.2 ($[M+Na]^+$, 100%); **HRMS** (ESI^+) calc. for $C_{13}H_{19}O_3NNa$ $[M+Na]^+$: 260.1257, found: 260.1255.

N-(1-hydroxy-3-phenylpropan-2-yl)-3-phenylpropanamide, OH-1w

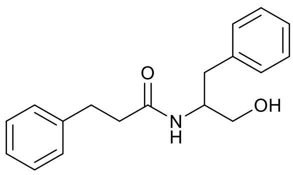

Following general procedure A, the product (1.02 g) was isolated by FC (100% EtOAc) in 72% yield as a white amorphous solid, starting from 3-phenylpropanoic acid (750 mg, 5 mmol), (±)-2-amino-3-phenylpropan-1-ol (907 mg, 6 mmol) and EDCI.HCl (1.15 g, 6 mmol) in CH_2Cl_2 (25 mL); **^{1}H-NMR** (400 MHz, $CDCl_3$) δ 7.27–7.02 (m, 10H, 10 × Ar*H*), 5.46 (d, *J* = 7.0, 1H, N*H*), 4.08–4.00 (m, 1H, NHC*H*), 3.52 (dd, *J* = 11.0, 3.5, 1H, $CHCH_AH_BOH$), 3.43 (dd, *J* = 11.0, 5.0, 1H, $CHCH_AH_BOH$), 2.85 (t, *J* = 7.5, 2H, CH_2CH_2Ph), 2.71 (dd, *J* = 7.5, 2.5, 2H, $CHCH_2Ph$), 2.43–2.30 (m, 2H, CH_2CH_2Ph); **^{13}C-NMR** (100 MHz, $CDCl_3$) δ 172.8, 140.8, 137.7, 129.3, 128.8, 128.7, 128.5, 126.8, 126.5, 64.1, 52.9, 38.7, 37.0, 31.8; **IR** (film, cm^{-1}) 3300, 3062, 3027, 2954, 1642, 1543, 1496,

1453, 1046, 746, 699; **LRMS** *m/z* (ESI$^+$) 306.2 ([M+Na]$^+$, 100%); **HRMS** (ESI$^+$) calc. for $C_{18}H_{21}O_2NNa$ [M+Na]$^+$: 306.1464, found: 306.1463; **m.p.** (°C): 96–97.

N-(1-hydroxypropan-2-yl)-3-phenylpropanamide, OH-1x

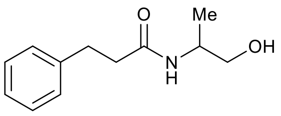

Following general procedure A, the product (390 mg) was isolated by FC (petrol/EtOAc 2:1) in 37% yield as a white amorphous solid, starting from 3-phenylpropanoic acid (750 mg, 5 mmol), (±)-2-aminopropan-1-ol (0.46 mL, 6 mmol) and EDCI.HCl (1.15 g, 6 mmol) in CH_2Cl_2 (25 mL); **^{1}H-NMR** (400 MHz, $CDCl_3$) δ 7.33–7.27 (m, 2H, 2 × Ar*H*), 7.24–7.18 (m, 3H, 3 × Ar*H*), 5.39 (br s, 1H, N*H*), 4.07–3.96 (m, 1H, NHC*H*), 3.63–3.54 (m, 1H, CHC$H_A$$H_B$OH), 3.48–3.39 (m, 1H, CHC$H_A$$H_B$OH), 2.97 (t, J = 7.5, 2H, CH_2CH_2Ph), 2.48 (td, J = 7.5, 3.5, 2H, CH_2CH_2Ph), 1.08 (d, J = 7.0, 2H, CH_3); **^{13}C-NMR** (100 MHz, $CDCl_3$) δ 172.9, 140.8, 128.7, 128.5, 126.5, 67.2, 47.9, 38.8, 32.0, 17.0; **IR** (film, cm^{-1}) 3288, 3085, 3027, 2970. 1637, 1550, 1497, 1452, 987, 749, 698; **LRMS** *m/z* (ESI$^+$) 230.2 ([M+Na]$^+$, 100%); **HRMS** (ESI$^+$) calc. for $C_{12}H_{17}O_2NNa$ [M+Na]$^+$: 230.1152, found: 230.1154; **m.p.** (°C): 79–81.

N-(1-hydroxy-4-methylpentan-2-yl)-3-phenylpropanamide, OH-1y

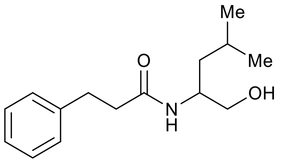

Following general procedure A, the product (742 mg) was isolated by FC (petrol/EtOAc 1:1) in 60% yield as a white amorphous solid, starting from 3-phenylpropanoic acid (750 mg, 5 mmol), (±)-2-amino-4-methylpentan-1-ol (0.76 mL, 6 mmol) and EDCI.HCl (1.15 g, 6 mmol) in CH_2Cl_2 (25 mL); **^{1}H-NMR** (400 MHz, $CDCl_3$) δ 7.34–7.27 (m, 2H, 2 × Ar*H*), 7.26–7.18 (m, 3H, 3 × Ar*H*), 5.47 (d, J = 8.0, 1H, N*H*), 4.06–3.94 (m, 1H, NHC*H*), 3.64–3.54 (m, 1H, C$H_A$$H_B$OH), 3.51–3.40 (m, 1H, C$H_A$$H_B$OH), 3.02–2.92 (m, 2H, PhCH_2CH_2), 2.67–2.59 (m, 1H, O*H*), 2.56–2.49 (m, 2H, PhCH_2CH_2), 1.52–1.39 (m, 1H, C*H*$(CH_3)_2$), 1.31–1.22 (m, 2H, $CH_2CH(CH_3)_2$), 0.89 (app dd, J = 6.5, 3.0, 6H, $CH(CH_3)_2$); **^{13}C-NMR** (100 MHz, $CDCl_3$) δ 173.0, 140.8, 128.7, 128.5, 126.5, 66.3, 50.1, 40.2, 38.7, 31.9, 24.9, 23.1, 22.2; **IR** (film, cm^{-1}) 3285, 3086, 3028, 2955, 2869, 1639, 1547, 1496, 1454, 1367, 1071, 1030, 748, 698; **LRMS** *m/z* (ESI$^+$) 272.2 ([M+Na]$^+$, 100%); **HRMS** (ESI$^+$) calc. for $C_{15}H_{23}O_2NNa$ [M+Na]$^+$: 272.1621, found: 272.1622; **m.p.** (°C): 61–63.

***N*-(1-hydroxy-3-methylbutan-2-yl)-3-phenylpropanamide, OH-1z**

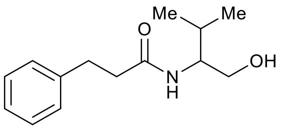

Following general procedure A, the product (750 mg) was isolated by FC (petrol/EtOAc 1:1) in 64% yield as a white amorphous solid, starting from 3-phenylpropanoic acid (750 mg, 5 mmol), (±)-2-amino-3-methylbutan-1-ol (618 mg, 6 mmol) and EDCI.HCl (1.15 g, 6 mmol) in CH_2Cl_2 (25 mL); **^{1}H-NMR** (400 MHz, $CDCl_3$) δ 7.35–7.28 (m, 2H, 2 × Ar*H*), 7.26–7.20 (m, 3H, 3 × Ar*H*), 5.51 (d, J = 8.0, 1H, N*H*), 3.74–3.54 (m, 3H, NHC*H*C*H*$_2$OH), 3.00 (t, J = 7.5, 2H, PhC*H*$_2$CH$_2$), 2.61–2.49 (m, 2H, PhC*H*$_2$CH$_2$), 2.46 (s, 1H, O*H*), 1.80 (h, J = 7.0, 1H, CH(C*H*$_3$)$_2$), 0.90 (d, J = 7.0, 3H, C*H*$_3$), 0.84 (d, J = 7.0, 3H, C*H*$_3$); **^{13}C-NMR** (100 MHz, $CDCl_3$) δ 173.2, 140.8, 128.7, 128.5, 126.5, 64.1, 57.3, 38.8, 32.0, 29.0, 19.5, 18.8; **IR** (film, cm^{-1}) 3294, 3028, 2960, 2873, 1637, 1545, 1496, 1454, 1075, 1028, 748, 698; **LRMS** *m/z* (ESI$^+$) 258.2 ([M+Na]$^+$, 100%); **HRMS** (ESI$^+$) calc. for $C_{14}H_{21}O_2NNa$ [M+Na]$^+$: 258.1465, found: 258.1464; **m.p.** (°C): 66–68.

***N*-benzyl-*N*-(2-hydroxyethyl)benzamide, OH-1aa**

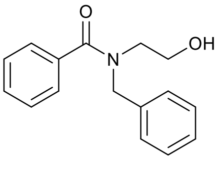

Following general procedure A, the product (468 mg) was isolated by FC (100% EtOAc) in 37% yield as a colourless oil, starting from benzoic acid (0.611 g, 5 mmol), 2-(benzylamino)ethan-1-ol (0.85 mL, 6 mmol) and EDCI.HCl (1.15 g, 6 mmol) in CH_2Cl_2 (25 mL); **^{1}H-NMR** (400 MHz, $CDCl_3$) (* denotes minor rotamer) [the compound exists as two rotamers, in a ratio of 3:1] δ 7.52–7.45 (m, 2H, 2 × Ar*H*), 7.44–7.26 (m, 6H, 6 × Ar*H*), 7.24–7.13 (m, 2H, 2 × Ar*H*), 4.85* (s, 2H, NC*H*$_2$Ph), 4.62 (s, 2H, NC*H*$_2$Ph), 3.89–3.48 (m, 4H, C*H*$_2$C*H*$_2$OH), 3.33 (s, 1H, O*H*); **^{13}C-NMR** (100 MHz, $CDCl_3$) (* denotes minor rotamer) δ 174.0, 172.8*, 136.5, 135.8*, 133.1, 132.5*, 130.0, 129.9*, 129.7*, 128.9, 128.6*, 128.6, 128.4, 128.2*, 128.1*, 127.8, 127.5*, 126.9, 61.4, 59.7*, 54.1, 50.1*, 48.6, 48.0*; **IR** (film, cm^{-1}) 3386, 3029, 1611, 1600, 1496, 1423, 1266, 1057, 1027; **LRMS** *m/z* (ESI$^+$) 256.2

([M+H]$^+$, 100%); **HRMS** (ESI$^+$) calc. for $C_{16}H_{18}O_2N$ [M+H]$^+$: 256.1332, found 256.1335. Data is in agreement with literature.[4]

***N*-benzyl-*N*-(2-hydroxyethyl)acetamide, OH-1ab**

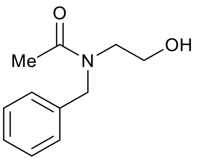

Following general procedure A, the product (370 mg) was isolated by FC (100% EtOAc) in 38% yield as a white crystalline solid, starting from glacial acetic acid (0.29 mL, 5 mmol), 2-(benzylamino)ethan-1-ol (0.85 mL, 6 mmol) and EDCI.HCl (1.15 g, 6 mmol) in CH_2Cl_2 (25 mL); **^{1}H-NMR** (400 MHz, $CDCl_3$) (* denotes minor rotamer) [the compound exists as two rotamers, in a ratio of 3:1] δ 7.42–7.23 (m, 3H, 3 × Ar*H*), 7.23–7.16 (m, 2H, 2 × Ar*H*), 4.67* (s, 2H, NC*H*$_2$Ph), 4.60 (s, 2H, NC*H*$_2$Ph), 3.79–3.67 (m, 2H, CH_2C*H*$_2$OH), 3.57 (t, *J* = 5.0, 2H, C*H*$_2$$CH_2$OH), 3.43* (t, *J* = 5.5, 2H, C*H*$_2$$CH_2$OH), 3.28 (s, 1H, O*H*), 2.24* (s, 3H, C*H*$_3$C(O)), 2.18 (s, 3H, C*H*$_3$C(O)); **^{13}C-NMR** (100 MHz, $CDCl_3$) (* denotes minor rotamer) δ 173.5, 171.7*, 137.9*, 136.4, 129.2, 128.8*, 128.1*, 128.0, 127.5*, 126.4, 62.3, 60.2*, 53.7, 50.0, 50.0*, 48.6*, 22.0*, 21.8; **IR** (film, cm^{-1}) 3383, 2936, 1621, 1424, 1073, 1027; **LRMS** *m/z* (ESI$^+$) 194.0 ([M+H]$^+$, 100%); **HRMS** (ESI$^+$) calc. for $C_{11}H_{16}O_2N$ [M+H]$^+$: 194.1176, found: 194.1178; **m.p.** (°C): 49–50.

Benzyl benzyl(2-hydroxyethyl)carbamate, OH-1ac

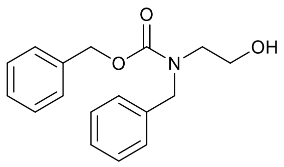

Following the procedure by Willis *et al.*,[5] to a round bottomed flask containing solution of 2-(benzylamino)ethan-1-ol (0.7 mL, 5 mmol) in Et_2O (25 mL) was added potassium carbonate (2 g, 15 mmol) and water (25 mL). The reaction mixture was then cooled to 0 °C and benzyl chloroformate (0.7 mL, 5 mmol) was added. The reaction mixture was then allowed to warm to RT and stirred for 16 hours. The two layers were separated, and the aqueous layer was extracted with Et_2O (2 × 25 mL). The combined organic layers were washed with 5% aqueous citric acid (3 × 30 mL) and brine (2 × 30 mL), dried over $MgSO_4$, filtered and concentrated in *vacuo*. The product (400 mg) was isolated by FC (petrol/EtOAc 2:1) in 28% yield as a colourless oil; **^{1}H-NMR** (400 MHz, $CDCl_3$) δ 7.50–7.15 (m, 10H, 10 × Ar*H*), 5.23 (s, 2H, PhC*H*$_2$O), 4.61 (s, 2H, NC*H*$_2$Ph), 3.79–3.65 (m, 2H, CH_2C*H*$_2$OH), 3.54–3.35 (m, 2H, C*H*$_2$$CH_2$OH); **^{13}C-NMR** (100 MHz, $CDCl_3$) δ 157.6, 137.6,

136.4, 128.7, 128.6, 128.1, 128.0, 127.5, 127.3, 67.6, 61.4, 51.6, 50.0; **IR** (film, cm^{-1}) 3429, 3031, 2939, 1676, 1496, 1472, 1453, 1419, 1223, 1121, 1029, 731, 696; **LRMS** *m/z* (ESI^+) 308.0 ($[M+Na]^+$, 100%); **HRMS** (ESI^+) calc. for $C_{17}H_{19}O_3NNa$ $[M+Na]^+$: 308.1257, found: 308.1255.

N-benzyl-*N*-(2-hydroxyethyl)cyclohexanecarboxamide, OH-1ad

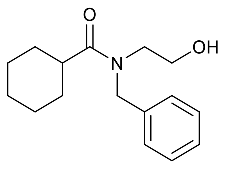

Following general procedure A, the product (600 mg) was isolated by FC (petrol/EtOAc 1:1) in 46% yield as a colourless oil, starting from cyclohexanecarboxylic acid (641 mg, 5 mmol), 2-(benzylamino)ethan-1-ol (0.85 mL, 6 mmol) and EDCI.HCl (1.15 g, 6 mmol) in CH_2Cl_2 (25 mL); **^{1}H-NMR** (400 MHz, $CDCl_3$) [the compound exists as two rotamers, in a ratio of 3:1] δ 7.44–7.16 (m, 5H, 5 × Ar*H*), 4.67* (s, 2H, NC*H*$_2$Ph), 4.64 (s, 2H, NC*H*$_2$Ph), 3.78–3.68 (m, 2H, NCH_2CH_2OH), 3.55 (t, *J* = 5.5, 2H, NC*H*$_2$CH$_2$OH), 3.45* (t, *J* = 5.5, 2H, NC*H*$_2$CH$_2$OH), 2.68* (tt, *J* = 11.5, 3.0, 1H, C*H*(CH$_2$)$_5$), 2.52 (tt, *J* = 11.5, 3.0, 1H, C*H*(CH$_2$)$_5$), 1.87–1.72 (m, 4H, 2 × C*H*$_2$), 1.71–1.53 (m, 3H, 1.5 × C*H*$_2$), 1.37–1.10 (m, 3H, 1.5 × C*H*$_2$); **^{13}C-NMR** (100 MHz, $CDCl_3$) (* denotes minor rotamer) δ 179.4, 136.9, 129.1, 128.8*, 127.9, 127.8*, 127.4*, 126.5, 62.5, 60.6*, 52.5, 50.1, 48.8*, 48.7*, 41.2, 40.7*, 29.8, 25.9*, 25.8; **IR** (film, cm^{-1}) 3391, 2927, 2854, 1612, 1495, 1448, 1205, 1074, 730, 697; **LRMS** *m/z* (ESI^+) 284.2 ($[M+Na]^+$, 100%); **HRMS** (ESI^+) calc. for $C_{16}H_{23}O_2NNa$ $[M+Na]^+$: 284.1619, found: 284.1621.

N-(1-hydroxy-3-phenylpropan-2-yl)cyclohexanecarboxamide, OH-1ae

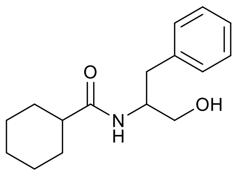

Following general procedure A, the product (842 mg) was isolated by FC (100% EtOAc) in 65% yield as a white amorphous solid, starting from cyclohexanecarboxylic acid (641 mg, 5 mmol), (±)-2-amino-3-phenylpropan-1-ol (907 mg, 6 mmol) and EDCI.HCl (1.15 g, 6 mmol) in CH_2Cl_2 (25 mL); **^{1}H-NMR** (400 MHz, $CDCl_3$) δ 7.35–7.18 (m, 5H, 5 × Ar*H*), 5.70 (d, *J* = 8.5, 1H, N*H*), 4.22–4.09 (m, 1H, NHC*H*), 3.74–3.64 (m, 1H, CHC*H*$_A$H$_B$OH), 3.63–3.54 (m, 1H, CHCH$_A$*H*$_B$OH), 3.03–2.80 (m, 3H, O*H*, CHC*H*$_2$Ph), 2.03 (tt, *J* = 11.5, 3.0, 1H, C*H*(CH$_2$)$_5$), 1.84–1.68 (m, 4H, 2 × C*H*$_2$),

1.69–1.59 (m, 1H, 0.5 × C*H*$_2$), 1.43–1.10 (m, 5H, 2.5 × C*H*$_2$); **^{13}C-NMR** (100 MHz, $CDCl_3$) δ 177.1, 137.8, 129.3, 128.8, 126.8, 64.9, 52.9, 45.6, 37.1, 29.8, 29.7, 25.8, 25.8; **IR** (film, cm^{-1}) 3279, 2980, 2927, 2852, 1641, 1548, 1496, 1446, 1386, 1054, 952, 752, 699; **LRMS** *m/z* (ESI$^+$) 284.2 ([M+Na]$^+$, 100%); **HRMS** (ESI$^+$) calc. for $C_{16}H_{24}O_2N$ [M+H]$^+$: 262.1802, found: 262.1802; **m.p.** (^{o}C): 127–129.

3.2 α-Amido aldehydes

N-benzyl-*N*-(2-oxoethyl)-3-phenylpropanamide, 1a

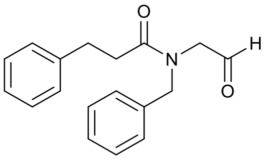

Following general procedure B, the product (943 mg) was isolated by FC (petrol/EtOAc 2:1) in 67% yield as a colourless oil, starting from anhydrous DMSO (0.76 mL, 10.7 mmol) in CH_2Cl_2 (25 mL), oxalyl chloride (0.44 mL, 5.35 mmol), *N*-benzyl-*N*-(2-hydroxyethyl)-3-phenylpropanamide (**OH-1a**) (1.417 g, 5 mmol) in CH_2Cl_2 (18 mL) and triethylamine (3.4 mL, 34 mmol); **^{1}H-NMR** (400 MHz, $CDCl_3$) (* denotes minor rotamer) [the compound exists as two rotamers, in a ratio of 4:1] δ 9.51 (s, 1H, C(O)*H*), 9.38* (s, 1H, C(O)*H*), 7.40–7.28 (m, 5H, 5 × Ar*H*), 7.26–7.17 (m, 3H, 3 × Ar*H*), 7.15–7.08 (m, 2H, 2 × Ar*H*), 4.68* (s, 2H, NC*H*$_2$Ph), 4.56 (s, 2H, NC*H*$_2$Ph), 4.09 (s, 2H, NC*H*$_2$C(O)H), 3.98* (s, 2H, NC*H*$_2$C(O)H), 3.09–3.01 (m, 2H, PhC*H*$_2$CH$_2$), 2.87–2.77 (m, 2H, PhCH$_2$C*H*$_2$), 2.57–2.50* (m, 2H, PhCH$_2$C*H*$_2$); **^{13}C-NMR** (100 MHz, $CDCl_3$) (* denotes minor rotamer) δ 197.4, 197.2*, 173.3, 172.5*, 141.0, 135.9, 129.2, 128.9*, 128.7*, 128.7, 128.6*, 128.6, 128.6*, 128.2, 128.0*, 126.8, 126.4*, 126.4, 57.1*, 56.0, 52.6, 50.3*, 35.3*, 34.8, 31.5*, 31.4; **IR** (film, cm^{-1}) 3028, 1732, 1645, 1452, 1121; **LRMS** *m/z* (ESI$^+$) 282.5 ([M+H]$^+$, 100%); **HRMS** (ESI$^+$) calc. for $C_{18}H_{20}O_2N$ [M+H]$^+$: 282.1489, found: 282.1489.

N-methyl-*N*-(2-oxoethyl)-3-phenylpropanamide, 1s

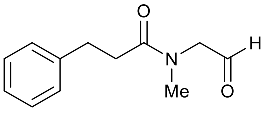

Following general procedure B, the product (596 mg) was isolated by FC (petrol/EtOAc 3:1) in 58% yield as a white amorphous solid, starting from anhydrous DMSO (0.76 mL, 10.7 mmol) in CH_2Cl_2

(25 mL), oxalyl chloride (0.44 mL, 5.4 mmol), *N*-(2-hydroxyethyl)-*N*-methyl-3-phenylpropanamide (**OH-1s**) (1.036 g, 5.0 mmol) in CH_2Cl_2 (18 mL) and triethylamine (3.4 mL, 24.3 mmol); **^{1}H-NMR** (400 MHz, $CDCl_3$) (* denotes minor rotamer) [the compound exists as two rotamers, in a ratio of 6:1] δ 9.50 (s, 1H, C(O)*H*), 9.48* (s, 1H, C(O)*H*), 7.27–7.10 (m, 5H, 5 × Ar*H*), 4.11 (s, 2H, NC*H*$_2$C(O)H), 4.00* (s, 2H, NC*H*$_2$C(O)H), 2.95–2.86 (m, 5H, NC*H*$_3$, PhC*H*$_2$CH$_2$), 2.67–2.59 (m, 2H, PhCH$_2$C*H*$_2$), 2.43–2.35* (m, 2H, PhCH$_2$C*H*$_2$); **^{13}C-NMR** (100 MHz, $CDCl_3$) (* denotes minor rotamer) δ 197.5, 196.9*, 173.1, 141.2, 128.7*, 128.7, 128.6, 128.5*, 126.4*, 126.4, 60.0*, 58.3, 36.8, 35.3*, 35.2*, 35.1, 31.5*, 31.2; **IR** (film, cm^{-1}) 3444, 2936, 1731, 1642, 1494, 1454, 1410, 1129; **LRMS** *m/z* (ESI$^+$) 206.2 ([M+H]$^+$, 100%); **HRMS** (ESI$^+$) calc. for $C_{12}H_{16}O_2N$ [M+H]$^+$: 206.1178, found: 206.1178; **m.p.** (ºC): 30–31.

N-(2-oxoethyl)-N,3-diphenylpropanamide, 1t

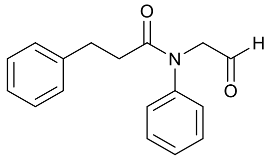

Following general procedure B, the product (729 mg) was isolated by FC (petrol/EtOAc 3:1) in 91% yield as a white amorphous solid, starting from anhydrous DMSO (0.47 mL, 6.0 mmol) in CH_2Cl_2 (15 mL), oxalyl chloride (0.27 mL, 3.3 mmol), *N*-(2-hydroxyethyl)-*N*,3-diphenylpropanamide (**OH-1t**) (0.808 g, 3 mmol) in CH_2Cl_2 (10.8 mL) and triethylamine (2.09 mL, 15 mmol); **^{1}H-NMR** (400 MHz, $CDCl_3$) (* denotes minor rotamer) [the compound exists as two rotamers, in a ratio of 4:1] δ 9.55 (s, 1H, C(O)*H*), 7.33–7.22 (m, 3H, 3 × Ar*H*), 7.20–7.13 (m, 2H, 2 × Ar*H*), 7.12–7.07 (m, 1H, 1 × Ar*H*), 7.06–6.96 (m, 4H, 4 × Ar*H*), 4.32 (s, 2H, NC*H*$_2$C(O)H), 2.91–2.80 (m, 2H, PhC*H*$_2$CH$_2$), 2.39 (m, 2H, PhCH$_2$C*H*$_2$); **^{13}C-NMR** (100 MHz, $CDCl_3$) (* denotes minor rotamer) δ 196.9, 172.9, 142.6, 141.0, 130.1, 128.6, 128.6, 128.5, 128.1, 126.3, 59.7, 35.7, 31.6; **IR** (film, cm^{-1}) 3028, 1732, 1655, 1595, 1494, 1412, 1384, 1075; **LRMS** *m/z* (ESI$^+$) 268.2 ([M+H]$^+$, 100%); **HRMS** (ESI$^+$) calc. for $C_{17}H_{18}O_2N$ [M+H]$^+$: 268.1332, found: 268.1331; **m.p.** (ºC): 47–48.

1-(3-phenylpropanoyl)pyrrolidine-2-carbaldehyde, 1u

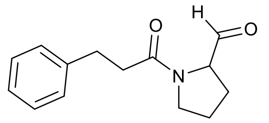

Following general procedure B, the product (943 mg) was isolated by FC (petrol/EtOAc 1:1) in 67% yield as a colourless oil, starting from anhydrous DMSO (0.76 mL, 10.7 mmol) in CH_2Cl_2 (25 mL), oxalyl chloride (0.44 mL, 5.35 mmol), 1-(2-(hydroxymethyl)pyrrolidin-1-yl)-3-phenylpropan-1-one (**OH-1u**) (1.167 g, 5 mmol) in CH_2Cl_2 (18 mL) and triethylamine (3.4 mL, 34 mmol); **^{1}H-NMR** (400 MHz, $CDCl_3$) (* denotes minor rotamer) [the compound exists as two rotamers, in a ratio of 7:1] δ 9.44 (d, J = 2.0, 1H, C(O)*H*), 9.36* (d, J = 2.0, 1H, C(O)*H*), 7.29–7.09 (m, 5H, 5 × Ar*H*), 4.40–4.33 (m, 1H, C*H*C(O)H), 4.09–4.01* (m, 1H, C*H*C(O)H), 3.59–3.50* (m, 2H, NCH_2), 3.46–3.27 (m, 2H, NCH_2), 2.99–2.86 (m, 2H, PhCH_2CH$_2$), 2.69–2.50 (m, 2H, PhCH$_2$CH_2), 2.43–2.30* (m, 2H, PhCH$_2$CH_2), 2.09–1.75 (m, 4H, NCH$_2$CH_2CH_2); **^{13}C-NMR** (100 MHz, $CDCl_3$) (* denotes minor rotamer) δ 199.5, 198.8*, 171.7, 141.2, 128.6, 128.6, 126.4, 126.4*, 65.7*, 64.9, 47.3, 46.8*, 36.9*, 36.5, 31.4*, 31.1, 28.1*, 26.1, 25.1, 22.7*; **IR** (film, cm^{-1}) 3027, 2952, 1730, 1635, 1497, 1429; **LRMS** *m/z* (ESI$^+$) 232.2 ([M+H]$^+$, 100%); **HRMS** (ESI$^+$) calc. for $C_{14}H_{18}O_2N$ [M+H]$^+$: 232.1332, found: 232.1335.

***N*-(2-oxoethyl)-3-phenylpropanamide, 1v**

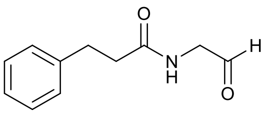

Following the procedure by Willis *et al.*,[5] to a round bottomed flask containing solution of *N*-(2,2-dimethoxyethyl)-3-phenylpropanamide (**AC-1v**) (498 mg, 2.1 mmol) in acetone (20 mL) was added 1 N aqueous hydrochloric acid (20 mL) and stirred for 16 hours at RT. The organic and aqueous layers were separated and extracted with Et_2O (2 × 60 mL). The organic layers were combined and washed with aqueous sodium bicarbonate (60 mL) and brine (60 mL), dried over $MgSO_4$, filtered and concentrated. The product (121 mg) was isolated by FC (petrol/EtOAc 1:1) in 30% yield as a colourless oil; **^{1}H-NMR** (400 MHz, $CDCl_3$) δ 9.62 (d, J = 1.0, 1H, C(O)*H*), 7.33–7.24 (m, 2H, 2 × Ar*H*), 7.24–7.15 (m, 3H, 3 × Ar*H*), 6.13 (br s, 1H, N*H*), 4.17 (dd, J = 5.0, 1.0, 1H, NHCH_2), 3.08–2.95 (m, 2H, PhCH_2CH$_2$), 2.61–2.54 (m, 2H, PhCH$_2$CH_2); **^{13}C-NMR** (100 MHz, $CDCl_3$) δ 196.5, 172.5, 140.7, 128.7, 128.4, 126.5, 50.4, 38.1, 31.6; **IR** (film, cm^{-1}) 3302, 3028, 2930, 1731, 1645, 1540, 1496,

1453, 1075, 750, 699; **LRMS** *m/z* (ESI$^+$) 192.0 ([M+H]$^+$, 100%); **HRMS** (ESI$^+$) calc. for $C_{11}H_{14}O_2N$ [M+H]$^+$: 192.1019, found: 192.1021.

***N*-(1-oxo-3-phenylpropan-2-yl)-3-phenylpropanamide, 1w**

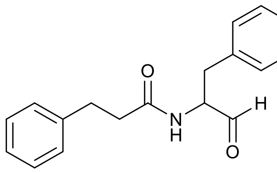

Following general procedure B, the product (289 mg) was isolated by FC (petrol/EtOAc 1:1) in 51% yield as a white amorphous solid, starting from anhydrous DMSO (0.3 mL, 4.4 mmol) in CH_2Cl_2 (10 mL), oxalyl chloride (0.2 mL, 2.2 mmol), *N*-(1-hydroxy-3-phenylpropan-2-yl)-3-phenylpropanamide (**OH-1w**) (566 mg, 2 mmol) in CH_2Cl_2 (8 mL) and triethylamine (1.4 mL, 9.6 mmol); **^{1}H-NMR** (400 MHz, $CDCl_3$) δ 9.58 (s, 1H, C(O)*H*), 7.37–7.13 (m, 8H, 8 × Ar*H*), 7.08–6.94 (m, 2H, 2 × Ar*H*), 5.87 (s, 1H, N*H*), 4.75–4.65 (m, 1H, C*H*C(O)H), 3.21–3.03 (m, 2H, NHCHC*H*$_2$), 3.00–2.92 (m, 2H, PhC*H*$_2$CH$_2$), 2.60–2.45 (m, 2H, PhCH$_2$C*H*$_2$); **^{13}C-NMR** (100 MHz, $CDCl_3$) δ 198.9, 172.2, 140.7, 135.6, 129.4, 128.9, 128.7, 128.5, 127.3, 126.5, 59.8, 38.2, 35.2, 31.6; **IR** (film, cm^{-1}) 3332, 3027, 2929, 2829, 1726, 1633, 1532, 1496, 1453, 740, 697; **LRMS** *m/z* (ESI$^+$) 304.1 ([M+Na]$^+$, 100%); **HRMS** (ESI$^+$) calc. for $C_{18}H_{20}O_2N$ [M+H]$^+$: 282.1489, found: 282.1488; **m.p.** (°C): 85–87.

***N*-(1-oxopropan-2-yl)-3-phenylpropanamide, 1x**

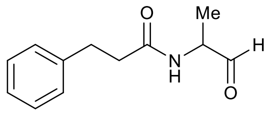

Following general procedure B, the product (78 mg) was isolated by FC (petrol/EtOAc 1:1) in 25% yield as a yellow amorphous solid, starting from anhydrous DMSO (0.22 mL, 3.3 mmol) in CH_2Cl_2 (7.5 mL), oxalyl chloride (0.15 mL, 1.65 mmol), *N*-(1-hydroxypropan-2-yl)-3-phenylpropanamide (**OH-1x**) (310 mg, 1.5 mmol) in CH_2Cl_2 (6 mL) and triethylamine (1 mL, 7.2 mmol); **^{1} H-NMR** (400 MHz, $CDCl_3$) δ 9.49 (s, 1H, C(O)*H*), 7.33–7.25 (m, 2H, 2 × Ar*H*), 7.24–7.17 (m, 3H, 3 × Ar*H*), 5.93 (br s, 1H, N*H*), 4.53–4.43 (m, 1H, NHC*H*), 2.98 (t, J = 7.5, 2H, PhC*H*$_2$CH$_2$), 2.61–2.47 (m, 2H, PhCH$_2$C*H*$_2$), 1.30 (d, J = 7.5, 3H, C*H*$_3$); **^{13}C-NMR** (100 MHz, $CDCl_3$) δ 199.1, 172.1, 140.7, 128.7, 128.5, 126.5, 54.6, 38.3, 31.7, 14.6; **IR** (film, cm^{-1}) 3294, 3028, 2934, 1732, 1644, 1536, 1497,

1453, 751, 700; **LRMS** *m/z* (ESI$^+$) 228.0 ([M+Na]$^+$, 100%); **HRMS** (ESI$^+$) calc. for $C_{12}H_{15}O_2NNa$ [M+Na]$^+$: 228.0995, found: 228.0997; **m.p.** (°C): 66–68.

N-(4-methyl-1-oxopentan-2-yl)-3-phenylpropanamide, 1y

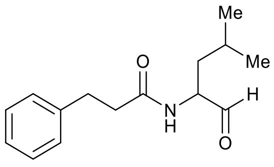

Following general procedure B, the product (221 mg) was isolated by FC (petrol/EtOAc 2:1) in 45% yield as a yellow oil, starting from anhydrous DMSO (0.3 mL, 4.4 mmol) in CH_2Cl_2 (10 mL), oxalyl chloride (0.2 mL, 2.2 mmol), *N*-(1-hydroxy-4-methylpentan-2-yl)-3-phenylpropanamide (**OH-1y**) (498 mg, 2 mmol) in CH_2Cl_2 (8 mL) and triethylamine (1.4 mL, 9.6 mmol); **^{1}H-NMR** (400 MHz, $CDCl_3$) δ 9.45–9.42 (m, 1H, C(O)*H*), 7.24–7.18 (m, 2H, 2 × Ar*H*), 7.16–7.09 (m, 3H, 3 × Ar*H*), 5.73 (br s, 1H, N*H*), 4.52–4.39 (m, 1H, NHC*H*), 2.96–2.85 (m, 2H, PhC*H*$_2$CH$_2$), 2.58–2.41 (m, 2H, PhCH$_2$C*H*$_2$), 1.64–1.41 (m, 2H, C*H*$_2$CH(CH$_3$)$_2$), 1.30–1.20 (m, 1H, C*H*(CH$_3$)$_2$), 0.93–0.73 (m, 6H, CH(C*H*$_3$)$_2$); **^{13}C-NMR** (100 MHz, $CDCl_3$) δ 199.8, 172.4, 140.7, 128.7, 128.5, 126.5, 57.3, 38.2, 38.1, 31.7, 24.8, 23.2, 22.1; **IR** (film, cm^{-1}) 3291, 3028, 2956, 2869, 1733, 1643, 1537, 1496, 1453, 748, 698; **LRMS** *m/z* (ESI$^+$) 248.2 ([M+H]$^+$, 100%); **HRMS** (ESI$^+$) calc. for $C_{15}H_{22}O_2N$ [M+H]$^+$: 248.1645, found: 248.1650.

N-(3-methyl-1-oxobutan-2-yl)-3-phenylpropanamide, 1z

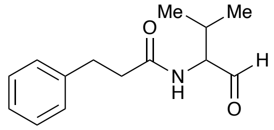

Following general procedure B, the product (363 mg) was isolated by FC (petrol/EtOAc 2:3) in 78% yield as a colourless oil, starting from anhydrous DMSO (0.3 mL, 4.4 mmol) in CH_2Cl_2 (10 mL), oxalyl chloride (0.2 mL, 2.2 mmol), *N*-(1-hydroxy-3-methylbutan-2-yl)-3-phenylpropanamide (**OH-1z**) (538 mg, 2 mmol) in CH_2Cl_2 (8 mL) and triethylamine (1.4 mL, 9.6 mmol); **^{1}H-NMR** (400 MHz, $CDCl_3$) δ 9.51 (s, 1H, C(O)*H*), 7.24–7.17 (m, 2H, 2 × Ar*H*), 7.16–7.09 (m, 3H, 3 × Ar*H*), 5.91 (d, *J* = 7.0, 1H, N*H*), 4.48 (dd, *J* = 7.0, 4.5, 1H, NHC*H*), 2.97–2.85 (m, 2H, PhC*H*$_2$CH$_2$), 2.58–2.44 (m, 2H, PhCH$_2$C*H*$_2$), 2.17 (heptd, *J* = 7.0, 4.5, 1H, C*H*(CH$_3$)$_2$), 0.83 (d, *J* = 7.0, 3H, C*H*$_3$), 0.80 (d, *J* = 7.0, 3H, C*H*$_3$); **^{13}C-NMR** (100 MHz, $CDCl_3$) δ 199.9, 172.4, 140.5, 128.6, 128.3, 126.3, 63.2, 38.2, 31.6, 29.0, 18.9, 17.7; **IR** (film, cm^{-1}) 3294, 3028, 2963, 1730, 1644, 1533, 1497, 1454, 747,

698; **LRMS** *m/z* (ESI$^+$) 234.2 ([M+H]$^+$, 100%); **HRMS** (ESI$^+$) calc. for $C_{14}H_{20}O_2N$ [M+H]$^+$: 234.1489, found: 234.1491.

N-benzyl-*N*-(2-oxoethyl)benzamide, 1aa

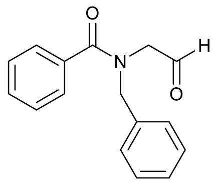

Following general procedure B, the product (187 mg) was isolated by FC (petrol/EtOAc 3:1) in 49% yield as a pale yellow solid, starting from anhydrous DMSO (0.23 mL, 3.21 mmol) in CH_2Cl_2 (7.5 mL), oxalyl chloride (0.13 mL, 1.61 mmol), *N*-benzyl-*N*-(2-hydroxyethyl)benzamide (**OH-1aa**) (0.383 g, 1.50 mmol) in CH_2Cl_2 (5.4 mL) and triethylamine (1.01 mL, 7.3 mmol); **^{1}H-NMR** (400 MHz, $CDCl_3$) (* denotes minor rotamer) [the compound exists as two rotamers, in a ratio of 3:1] δ 9.59 (s, 1H,), 9.40* (s, 1H), 7.60–7.51 (m, 2H, 2 × Ar*H*), 7.47–7.28 (m, 6H, 6 × Ar*H*), 7.22–7.14 (m, 2H, 2 × Ar*H*), 4.81* (s, 2H, NC*H*$_2$Ph), 4.61 (s, 2H, NC*H*$_2$Ph), 4.20 (s, 2H, NC*H*$_2$C(O)H), 4.00* (s, 2H, NC*H*$_2$C(O)H); **^{13}C-NMR** (100 MHz, $CDCl_3$) (* denotes minor rotamer) δ 196.9, 172.8, 138.6, 136.0, 130.3, 130.2*, 129.2, 128.8, 128.3, 127.3, 127.2, 59.6*, 55.1, 54.4; **IR** (film, cm^{-1}) 1733, 1635, 1430, 1254, 1027; **LRMS** *m/z* (ESI$^+$) 254.5 ([M+H]$^+$, 100%); **HRMS** (ESI$^+$) calc. for $C_{16}H_{16}O_2N$ [M+H]$^+$: 254.1176, found: 254.1178; **m.p.** (°C): 47–48. Data is in agreement with literature.[4]

N-benzyl-*N*-(2-oxoethyl)acetamide, 1ab

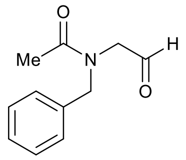

Following general procedure B, the product (194 mg) was isolated by FC (petrol/EtOAc 1:1) in 68% yield as a colourless oil, starting from anhydrous DMSO (0.23 mL, 3.21 mmol) in CH_2Cl_2 (7.5 mL), oxalyl chloride (0.13 mL, 1.61 mmol), *N*-benzyl-*N*-(2-hydroxyethyl)acetamide (**OH-1ab**) (0.290 g, 1.50 mmol) in CH_2Cl_2 (5.4 mL) and triethylamine (1.01 mL, 7.3 mmol); **^{1}H-NMR** (400 MHz, $CDCl_3$) (* denotes minor rotamer) [the compound exists as two rotamers, in a ratio of 4:1] δ 9.50–9.48 (m, 1H, C(O)*H*), 7.42–7.27 (m, 3H, 3 × Ar*H*), 7.25–7.16 (m, 2H, 2 × Ar*H*), 4.65* (s, 2H, NC*H*$_2$Ph), 4.60 (s, 2H, NC*H*$_2$Ph), 4.08 (s, 2H, NC*H*$_2$C(O)H), 4.04* (s, 2H, NC*H*$_2$C(O)H), 2.26

(s, 3H, CH_3), 2.07* (s, 3H, CH_3); **^{13}C-NMR** (100 MHz, $CDCl_3$) δ 197.4, 197.0*, 171.7, 170.9*, 136.6*, 135.9, 129.3, 128.9*, 128.6*, 128.3, 128.0*, 126.9, 57.8*, 55.8, 53.6, 50.1*, 21.7*, 21.2; **IR** (film, cm^{-1}) 3376, 2927, 1730, 1643, 1433, 1242, 1024; **LRMS** *m/z* (ESI$^+$) 192.4 ([M + H]$^+$, 100%); **HRMS** (ESI$^+$) calc. for $C_{11}H_{14}O_2N$ [M+ H]$^+$: 192.1019, found: 192.1020.

Benzyl benzyl(2-oxoethyl)carbamate, 1ac

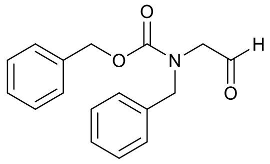

Following general procedure B, the product (200 mg) was isolated by FC (petrol/EtOAc 4:1) in 70% yield as a colourless oil, starting from anhydrous DMSO (0.15 mL, 2.2 mmol) in CH_2Cl_2 (5 mL), oxalyl chloride (0.1 mL, 1.1 mmol), Benzyl benzyl(2-hydroxyethyl)carbamate (**OH-1ac**) (300 mg, 1 mmol) in CH_2Cl_2 (4 mL) and triethylamine (0.7 mL, 4.8 mmol); **^{1}H-NMR** (400 MHz, $CDCl_3$) [the compound exists as two rotamers, in a ratio of 1:1] δ 9.53 (s, 1H, C(O)*H*), 9.44* (s, 1H, C(O)*H*), 7.44–7.25 (m, 8H, 8 × Ar*H*), 7.24–7.18 (m, 2H, 2 × Ar*H*), 5.26 (s, 2H, PhCH_2O), 5.22* (s, 2H, PhCH_2O), 4.63 (s, 2H, PhCH_2N), 4.61* (s, 2H, PhCH_2N), 4.03 (s, 2H, NCH_2C(O)H), 3.94 (s, 2H, NCH_2C(O)H); **^{13}C-NMR** (100 MHz, $CDCl_3$) δ 198.1, 156.7, 136.7, 136.2, 129.0, 128.7, 128.4, 128.4, 128.1, 128.0*, 127.9, 68.1, 68.0*, 56.7, 56.1*, 52.2, 51.9*; **IR** (film, cm^{-1}) 3032, 2921, 1733, 1696, 1496, 1452, 1426, 1230, 1123, 734, 697; **LRMS** *m/z* (ESI$^+$) 306.1 ([M+Na]$^+$, 100%); **HRMS** (ESI$^+$) calc. for $C_{17}H_{17}O_3NNa$ [M+Na]$^+$: 306.1100, found: 306.1098.

***N*-benzyl-*N*-(2-oxoethyl)cyclohexanecarboxamide, 1ad**

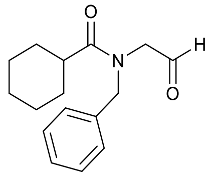

Following general procedure B, the product (361 mg) was isolated by FC (petrol/EtOAc 3:1) in 70% yield as a pale yellow oil, starting from anhydrous DMSO (0.3 mL, 4.4 mmol) in CH_2Cl_2 (10 mL), oxalyl chloride (0.2 mL, 2.2 mmol), *N*-benzyl-*N*-(2-hydroxyethyl)cyclohexanecarboxamide (**OH-1ad**) (522 mg, 2 mmol) in CH_2Cl_2 (8 mL) and triethylamine (1.4 mL, 9.6 mmol); **^{1}H-NMR** (400 MHz, $CDCl_3$) (* denotes minor rotamer) [the compound exists as two rotamers, in a ratio of 4:1] δ 9.43* (s, 1H, C(O)*H*), 9.39 (s, 1H, C(O)*H*), 7.35–7.16 (m, 3H, 3 × Ar*H*), 7.14–7.07 (m, 2H, 2 × Ar*H*),

4.56 (s, 2H, NCH_2Ph), 3.99* (s, 2H, NCH_2C(O)H), 3.93 (s, 2H, NCH_2C(O)H), 2.56 (tt, J = 11.5, 3.0, 1H, CH(CH$_2$)$_5$), 2.18–2.07* (m, 1H, CH(CH$_2$)$_5$), 1.82–1.44 (m, 7H, 7 × CH_2), 1.27–1.04 (m, 3H, 3 × CH_2); **^{13}C-NMR** (100 MHz, $CDCl_3$) (* denotes minor rotamer) δ 197.7*, 197.5, 177.4, 176.6*, 136.9*, 136.3, 129.2, 128.9*, 128.4*, 128.1, 127.8*, 126.8, 56.9*, 55.8, 52.4, 50.0*, 41.1*, 40.6, 29.7, 29.6*, 25.8; **IR** (film, cm^{-1}) 2928, 2854, 1730, 1634, 1495, 1448, 1206, 729, 698; **LRMS** *m/z* (ESI$^+$) 260.1 ([M+H]$^+$, 100%); **HRMS** (ESI$^+$) calc. for $C_{16}H_{22}O_2N$ [M+H]$^+$: 260.1645, found: 260.1645.

N-(1-oxo-3-phenylpropan-2-yl)cyclohexanecarboxamide, 1ae

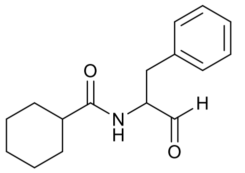

Following general procedure B, the product (237 mg) was isolated by FC (petrol/EtOAc 2:1) in 46% yield as a white amorphous solid, starting from anhydrous DMSO (0.3 mL, 4.4 mmol) in CH_2Cl_2 (10 mL), oxalyl chloride (0.2 mL, 2.2 mmol), *N*-(1-hydroxy-3-phenylpropan-2-yl)cyclohexanecarboxamide (**OH-1ae**) (522 mg, 2 mmol) in CH_2Cl_2 (8 mL) and triethylamine (1.4 mL, 9.6 mmol); **^{1}H-NMR** (400 MHz, $CDCl_3$) δ 9.64 (s, 1H, C(O)H), 7.34–7.22 (m, 3H, 3 × ArH), 7.18–7.10 (m, 2H, 2 × ArH), 5.96 (s, 1H, NH), 4.75–4.68 (m, 1H, NHCH), 3.18 (d, J = 6.5, 2H, CHCH_2Ph), 2.11 (tt, J = 12.0, 3.5, 1H, CH(CH$_2$)$_5$), 1.88–1.56 (m, 5H, 2.5 × CH_2), 1.48–1.32 (m, 2H, CH_2), 1.33–1.13 (m, 3H, 1.5 × CH_2); **^{13}C-NMR** (100 MHz, $CDCl_3$) δ 199.0, 176.0, 135.6, 129.4, 128.7, 127.1, 59.5, 45.2, 35.1, 29.7, 29.5, 25.7, 25.6, 25.6; **IR** (film, cm^{-1}) 3303, 3029, 2928, 2854, 1732, 1638, 1530, 1450, 1257, 1216, 739, 699; **LRMS** *m/z* (ESI$^+$) 260.2 ([M+H]$^+$, 100%); **HRMS** (ESI$^+$) calc. for $C_{16}H_{22}O_2N$ [M+H]$^+$: 260.1645, found: 260.1642; **m.p.** (°C): 97–99.

3.3 Hydroacylation products

(*E*)-*N*-benzyl-*N*-(2-oxodec-3-en-1-yl)-3-phenylpropanamide, 2a

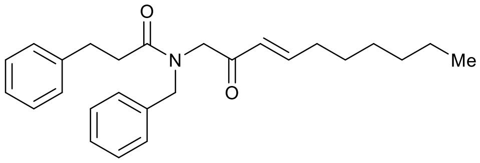

Following general procedure C, the product (63 mg) was isolated by FC (petrol/EtOAc 4:1) in 80% yield as a pale yellow oil, starting from *N*-benzyl-*N*-(2-oxoethyl)-3-phenylpropanamide (**1a**) (56 mg, 0.20 mmol) and 1-octyne (35 µL, 0.24 mmol); **^{1}H-NMR** (400 MHz, $CDCl_3$) (* denotes minor rotamer) [the compound exists as two rotamers, in a ratio of 2.5:1] δ 7.28–7.14 (m, 5H, 5 × Ar*H*), 7.13–7.05 (m, 3H, 3 × Ar*H*), 7.04–6.96 (m, 2H, 2 × Ar*H*), 6.86–6.71 (m, 1H, C(O)CH=C*H*CH_2), 6.01 (dt, *J* = 16.0, 1.5, 1H, C(O)C*H*=$CHCH_2$), 5.95* (dt, *J* = 16.0, 1.5, 1H, C(O)C*H*=$CHCH_2$), 4.53* (s, 2H, NCH_2Ph), 4.47 (s, 2H, NCH_2Ph), 4.22 (s, 2H, NCH_2C(O)), 3.95* (s, 2H, NCH_2C(O)), 2.97–2.88 (m, 2H, PhC$H_2$$CH_2$), 2.71–2.65 (m, 2H, Ph$CH_2CH_2$), 2.42–2.36* (m, 2H, Ph$CH_2CH_2$), 2.14–2.04 (m, 2H, C$H_2$$CH_2$$(CH_2)_3$$CH_3$), 1.41–1.28 (m, 2H, CH_2C$H_2$$(CH_2)_3$$CH_3$), 1.27–1.14 (m, 6H, CH_2CH_2(CH_2)$_3$$CH_3$), 0.85–0.71 (m, 3H, $CH_2CH_2(CH_2)_3$CH_3); **^{13}C-NMR** (100 MHz, $CDCl_3$) (* denotes minor rotamer) δ 194.4, 193.9*, 172.9, 172.9*, 149.9*, 148.9, 141.4*, 141.2, 137.1*, 136.5, 129.0, 128.6*, 128.5, 128.5, 128.4*, 127.8, 127.6, 127.5*, 126.7*, 126.6, 126.2*, 126.14, 54.2*, 52.7, 52.0, 49.9*, 35.1*, 34.8, 32.7*, 32.7, 31.6, 31.6*, 31.5*, 31.3, 28.9, 28.9*, 27.9, 27.9*, 22.6, 22.5*, 14.1, 14.1*; **IR** (film, cm^{-1}) 2928, 1689, 1651, 1453, 1360, 1206; **LRMS** *m/z* (ESI^+) 414.2 ($[M+Na]^+$, 100%); **HRMS** (ESI^+) calc. for $C_{26}H_{33}O_2NNa$ $[M+Na]^+$: 414.2403, found: 414.2398.

(*E*)-*N*-benzyl-*N*-(7-methyl-2-oxooct-3-en-1-yl)-3-phenylpropanamide, 2b

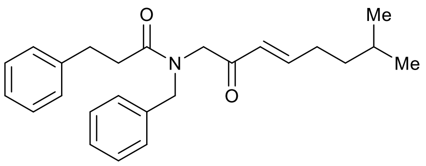

Following general procedure C, the product (45 mg) was isolated by FC (petrol/EtOAc 4:1) in 60% yield as a yellow oil, starting from *N*-benzyl-*N*-(2-oxoethyl)-3-phenylpropanamide (**1a**) (56 mg, 0.20 mmol) and 5-methylhex-1-yne (31 µL, 0.24 mmol); **^{1}H-NMR** (400 MHz, $CDCl_3$) [the compound exists as two rotamers, in a ratio of 2.5:1] δ 7.28–7.15 (m, 5H, 5 × Ar*H*), 7.14–7.06 (m, 3H, 3 × Ar*H*), 7.05–6.97 (m, 2H, 2 × Ar*H*), 6.86–6.72 (m, 1H, C(O)CH=C*H*), 6.02

(dt, J = 16.0, 1.5, 1H, C(O)C*H*=CH), 5.96* (dt, J = 16.0, 1.5, 1H, C(O)C*H*=CH), 4.54* (s, 2H, NCH_2Ph), 4.48 (s, 2H, NCH_2Ph), 4.22 (s, 2H, NCH_2C(O)), 3.96* (s, 2H, NCH_2C(O)), 2.98–2.89 (m, 2H, PhCH_2CH$_2$), 2.74–2.65 (m, 2H, PhCH$_2$CH_2), 2.44–2.35* (m, 2H, PhCH$_2$CH_2), 2.16–2.06 (m, 2H, CH_2CH$_2$CH(CH$_3$)$_2$), 1.53–1.42 (m, 1H, CH$_2$CH$_2$C*H*(CH$_3$)$_2$), 1.28–1.17 (m, 2H, CH$_2$CH_2CH(CH$_3$)$_2$), 0.81 (d, J = 6.5, 6H, CH$_2$CH$_2$CH(CH_3)$_2$); **^{13}C-NMR** (100 MHz, CDCl$_3$) (* denotes minor rotamer) δ 194.4, 193.9*, 172.9, 172.8*, 150.1*, 149.1, 141.3*, 141.2, 137.1*, 136.3, 128.9, 128.6*, 128.5, 128.5, 128.4*, 127.7, 127.5*, 127.5, 126.6, 126.5*, 126.1*, 126.1, 54.2*, 52.7, 52.0, 49.9*, 37.0, 36.9*, 35.1*, 34.8, 31.4*, 31.3, 30.6*, 30.5, 27.6*, 27.6, 22.4, 22.3*; **IR** (film, cm^{-1}) 3027, 2954, 2869, 1686, 1647, 1495, 1452, 1206, 734, 699; **LRMS** *m/z* (ESI$^+$) 400.2 ([M+Na]$^+$, 100%); **HRMS** (ESI$^+$) calc. for $C_{25}H_{32}O_2N$ [M+H]$^+$: 378.2438, found: 378.2429.

(*E*)-*N*-benzyl-*N*-(5-cyclohexyl-2-oxopent-3-en-1-yl)-3-phenylpropanamide, 2c

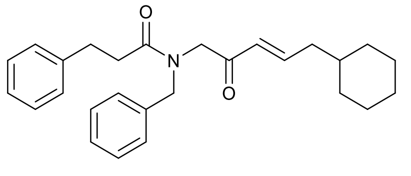

Following general procedure C, the product (50 mg) was isolated by FC (petrol/EtOAc 4:1) in 62% yield as a yellow oil, starting from *N*-benzyl-*N*-(2-oxoethyl)-3-phenylpropanamide (**1a**) (56 mg, 0.20 mmol) and prop-2-yn-1-ylcyclohexane (34 µL, 0.24 mmol); **^{1}H-NMR** (400 MHz, CDCl$_3$) [the compound exists as two rotamers, in a ratio of 2.5:1] δ 7.30–7.15 (m, 5H, 5 × Ar*H*), 7.14–7.07 (m, 3H, 3 × Ar*H*), 7.06–6.94 (m, 2H, 2 × Ar*H*), 6.86–6.70 (m, 1H, C(O)CH=C*H*), 6.00 (dt, J = 16.0, 1.5, 1H, C(O)C*H*=CH), 5.94* (dt, J = 16.0, 1.5, 1H, C(O)C*H*=CH), 4.55* (s, 2H, NCH_2Ph), 4.49 (s, 2H, NCH_2Ph), 4.23 (s, 2H, NCH_2C(O)), 3.95* (s, 2H, NCH_2C(O)), 3.00–2.88 (m, 2H, PhCH_2CH$_2$), 2.73–2.64 (m, 2H, PhCH$_2$CH_2), 2.45–2.37* (m, 2H, PhCH$_2$CH_2), 2.05–1.95 (m, 2H, CH_2CH(CH$_2$)$_5$), 1.71–1.50 (m, 5H, 2.5 × CH_2), 1.39–1.26 (m, 1H, CH$_2$C*H*(CH$_2$)$_5$), 1.21–0.98 (m, 3H, 1.5 × CH_2), 0.92–0.73 (m, 2H, CH_2); **^{13}C-NMR** (100 MHz, CDCl$_3$) (* denotes minor rotamer) δ 194.4, 193.9*, 173.0, 172.9*, 148.9*, 147.8, 141.4*, 141.3, 137.2*, 136.4, 129.0, 128.7, 128.6, 128.6, 128.5*, 127.8, 127.7*, 127.6*, 126.7, 126.2*, 126.2, 54.3*, 52.8, 52.1, 50.0*, 40.7, 37.4, 37.3*, 35.2*, 34.9, 33.3, 31.5*, 31.4, 26.4, 26.3*, 26.3, 26.2*; **IR** (film, cm^{-1}) 3027, 2921, 2850, 1686, 1647, 1495, 1448, 1205, 734, 698; **LRMS** *m/z* (ESI$^+$) 426.2 ([M+Na]$^+$, 100%); **HRMS** (ESI$^+$) calc. for $C_{27}H_{34}O_2N$ [M+H]$^+$: 404.2584, found: 404.2580.

(*E*)-*N*-benzyl-*N*-(4-cyclopropyl-2-oxobut-3-en-1-yl)-3-phenylpropanamide, 2d

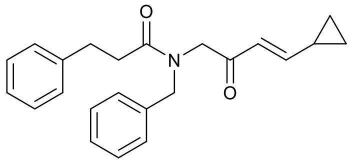

Following general procedure C, the product (30 mg) was isolated by FC (petrol/EtOAc 4:1) in 43% yield as a pale yellow oil, starting from *N*-benzyl-*N*-(2-oxoethyl)-3-phenylpropanamide (**1a**) (56 mg, 0.20 mmol) and ethynylcyclopropane (20 µL, 0.24 mmol); **^{1}H-NMR** (400 MHz, $CDCl_3$) [the compound exists as two rotamers, in a ratio of 2:1] δ 7.28–7.15 (m, 5H, 5 × Ar*H*), 7.15–7.05 (m, 3H, 3 × Ar*H*), 7.04–6.99 (m, 2H, 2 × Ar*H*), 6.35–6.23 (m, 1H, C(O)CH=C*H*), 6.16–6.04 (m, 1H, C(O)C*H*=CH), 4.55* (s, 2H, NCH_2Ph), 4.48 (s, 2H, NCH_2Ph), 4.17 (s, 2H, NCH_2C(O)), 3.91* (s, 2H, NCH_2C(O)), 3.00–2.89 (m, 2H, PhCH_2CH$_2$), 2.74–2.64 (m, 2H, PhCH$_2$CH_2), 2.46–2.37* (m, 2H, PhCH$_2$CH_2), 1.52–1.40 (m, 1H, CH=CHC*H*CH$_2$), 0.98–0.85 (m, 2H, CH=CHCHCH_2), 0.64–0.52 (m, 2H, CH=CHCHCH_2); **^{13}C-NMR** (100 MHz, $CDCl_3$) (* denotes minor rotamer) δ 193.6, 193.1*, 173.0, 155.2*, 154.1, 141.4*, 141.4, 137.2*, 136.5, 129.0, 128.7*, 128.6, 128.6, 128.6, 128.5*, 127.8, 127.6*, 126.7, 126.2*, 124.5, 123.3*, 54.5*, 52.9, 52.1, 50.0*, 35.2*, 34.9, 31.5*, 31.4, 15.3*, 15.1, 9.6*, 9.3; **IR** (film, cm^{-1}) 3027, 2924, 1646, 1495, 1452, 1208, 947, 736, 700; **LRMS** *m/z* (ESI$^+$) 370.2 ([M+Na]$^+$, 100%); **HRMS** (ESI$^+$) calc. for $C_{23}H_{25}O_2NNa$ [M+Na]$^+$: 370.1777, found: 370.1772.

(*E*)-*N*-benzyl-*N*-(2-oxo-5-phenylpent-3-en-1-yl)-3-phenylpropanamide, 2e

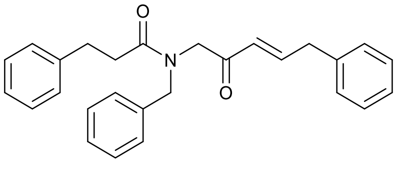

Following general procedure C, the product (44 mg) was isolated by FC (petrol/EtOAc 4:1) in 55% yield as a yellow oil, starting from *N*-benzyl-*N*-(2-oxoethyl)-3-phenylpropanamide (**1a**) (56 mg, 0.20 mmol) and prop-2-yn-1-ylbenzene (29 µL, 0.24 mmol); **^{1}H-NMR** (400 MHz, $CDCl_3$) [the compound exists as two rotamers, in a ratio of 3:1] δ 7.31–6.83 (m, 16H, C(O)CH=C*H*, 15 × Ar*H*), 5.99 (dt, *J* = 16.0, 1.5, 1H, C(O)C*H*=CH), 5.92* (dt, *J* = 16.0, 1.5, 1H, C(O)C*H*=CH), 4.53* (s, 2H, NCH_2Ph), 4.46 (s, 2H, NCH_2Ph), 4.20 (s, 2H, NCH_2C(O)), 3.94* (s, 2H, NCH_2C(O)), 3.46–3.37 (m, 2H, CH=CHCH_2Ph), 2.98–2.88 (m, 2H, PhCH_2CH$_2$), 2.72–2.62 (m, 2H, PhCH$_2$CH_2), 2.43–2.34* (m, 2H, PhCH$_2$CH_2); **^{13}C-NMR** (100 MHz, $CDCl_3$) (* denotes minor rotamer) δ 194.4, 193.9*, 173.0, 172.9*, 147.7*, 146.7, 141.4*, 141.3, 137.4, 137.1*, 137.0*, 136.3, 129.0, 129.0, 129.0*, 128.9*, 128.9, 128.7*, 128.6, 128.6, 128.5*, 128.4, 127.9, 127.6*, 127.5*, 127.1*, 126.9, 126.7, 126.3*, 126.2, 54.4*,

52.9, 52.1, 49.9*, 39.0, 35.2*, 34.8, 31.5*, 31.4; **IR** (film, cm^{-1}) 3027, 2980, 1687, 1645, 1603, 1495, 1452, 1207, 1076, 750, 698; **LRMS** *m/z* (ESI^+) 420.2 ($[M+Na]^+$, 100%); **HRMS** (ESI^+) calc. for $C_{27}H_{28}O_2N$ $[M+H]^+$: 398.2115, found: 398.2124.

(*E*)-*N*-benzyl-*N*-(4-(cyclohex-1-en-1-yl)-2-oxobut-3-en-1-yl)-3-phenylpropanamide, 2f

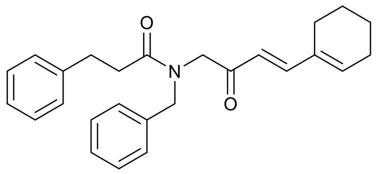

Following general procedure C, the product (65 mg) was isolated by FC (petrol/EtOAc 4:1) in 84% yield as a yellow oil, starting from *N*-benzyl-*N*-(2-oxoethyl)-3-phenylpropanamide (**1a**) (56 mg, 0.20 mmol) and 1-ethynylcyclohex-1-ene (28 µL, 0.24 mmol); **^{1}H-NMR** (400 MHz, $CDCl_3$) [the compound exists as two rotamers, in a ratio of 2:1] δ 7.28–7.14 (m, 5H, 5 × Ar*H*), 7.14–7.06 (m, 4H, C(O)CH=C*H,* 3 × Ar*H*), 7.05–6.99 (m, 2H, 2 × Ar*H*), 6.19–6.10 (m, 1H, C(O)CH=CHC=C*H*), 5.98 (d, *J* = 16.0, 1H, C(O)C*H*=CH), 5.90* (d, *J* = 16.0, 1H, C(O)C*H*=CH), 4.55* (s, 2H, NCH_2Ph), 4.49 (s, 2H, NCH_2Ph), 4.25 (s, 2H, NCH_2C(O)), 3.99* (s, 2H, NCH_2C(O)), 2.98–2.89 (m, 2H, PhCH_2CH$_2$), 2.74–2.64 (m, 2H, PhCH$_2$CH_2), 2.45–2.38* (m, 2H, PhCH$_2$CH_2), 2.18–2.08 (m, 2H, C(O)CH=CHC=CHCH$_2$), 2.06–1.93 (m, 2H, C(O)CH=CHCCH_2), 1.67–1.45 (m, 4H, CH$_2$(CH_2)$_2$CH$_2$); **^{13}C-NMR** (100 MHz, $CDCl_3$) (* denotes minor rotamer) δ 194.9, 194.3*, 173.0, 147.9*, 147.2, 142.3*, 141.4*, 141.3, 141.0, 137.2*, 136.5, 135.2, 135.1*, 129.0, 128.7*, 128.6, 128.5, 128.5, 127.8, 127.6*, 126.7, 126.2*, 120.4, 119.2*, 54.6*, 53.1, 52.0, 50.0*, 35.1*, 34.9, 31.5*, 31.4, 26.8*, 26.8, 24.1, 24.1*, 22.1, 22.0, 22.0*, 21.9*; **IR** (film, cm^{-1}) 3027, 2930, 1645, 1595, 1495, 1451, 1193, 1078, 982, 733, 699; **LRMS** *m/z* (ESI^+) 388.2 ($[M+H]^+$, 100%); **HRMS** (ESI^+) calc. for $C_{26}H_{29}O_2NNa$ $[M+Na]^+$: 410.2101, found: 410.2088.

(*E*)-*N*-benzyl-*N*-(2-oxo-4-phenylbut-3-en-1-yl)-3-phenylpropanamide, 2g

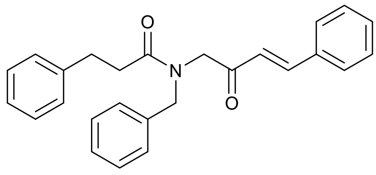

Following general procedure C, the product (71 mg) was isolated by FC (petrol/EtOAc 4:1) in 92% yield as a yellow amorphous solid, starting from *N*-benzyl-*N*-(2-oxoethyl)-3-phenylpropanamide (**1a**) (56 mg, 0.20 mmol) and ethynylbenzene (26 µL, 0.24 mmol); **^{1}H-NMR** (400 MHz, $CDCl_3$)

[the compound exists as two rotamers, in a ratio of 2.5:1] δ 7.66 (d, *J* = 16.0, 1H, C(O)CH=C*H*), 7.61* (d, *J* = 16.0, 1H, C(O)CH=C*H*), 7.57–7.49 (m, 2H, 2 × Ar*H*), 7.47–7.10 (m, 13H, 13 × Ar*H*), 6.76 (d, *J* = 16.0, 1H, C(O)C*H*=CH), 6.68* (d, *J* = 16.0, 1H, C(O)C*H*=CH), 4.72* (s, 2H, NCH_2Ph), 4.64 (s, 2H, NCH_2Ph), 4.46 (s, 2H, NCH_2C(O)), 4.20* (s, 2H, NCH_2C(O)), 3.12–3.02 (m, 2H, PhCH_2CH$_2$), 2.87–2.80 (m, 2H, PhCH$_2$CH_2), 2.62–2.53* (m, 2H, PhCH$_2$CH_2); **^{13}C-NMR** (100 MHz, $CDCl_3$) (* denotes minor rotamer) δ 194.5, 194.0*, 173.0, 172.9*, 144.5*, 143.7, 141.3*, 141.3, 137.1*, 136.3, 134.3, 133.9*, 131.2*, 130.8, 129.1*, 129.0, 128.7*, 128.6, 128.6, 128.5, 128.5, 127.9, 127.6*, 126.8, 126.2, 123.4, 122.1*, 55.0*, 53.4, 52.1, 50.0*, 35.1*, 34.8, 31.5*, 31.4; **IR** (film, cm^{-1}) 3027, 2922, 1698, 1644, 1611, 1576, 1494, 1450, 1186, 1090, 979, 748, 697; **LRMS** *m/z* (ESI$^+$) 406.2 ([M+Na]$^+$, 100%); **HRMS** (ESI$^+$) calc. for $C_{26}H_{25}O_2NNa$ [M+Na]$^+$: 406. 1777, found: 406.1774; **m.p.** (°C): 97–99.

Gram scale: Following general procedure C, the product (1.03 g) was isolated by FC (petrol/EtOAc 4:1) in 73% yield as a yellow amorphous solid, starting from *N*-benzyl-*N*-(2-oxoethyl) 3-phenylpropanamide (**1a**) (1.04 g, 3.7 mmol), ethynylbenzene (0.47 mL, 4.44 mmol) and [Rh(dppe)(C_6H_5F)][BAr$^F_4$] (216 mg, 0.15 mmol, 4 mol%) in DCM (1.8 mL, 2 M).

(*E*)-*N*-benzyl-*N*-(4-(4-bromophenyl)-2-oxobut-3-en-1-yl)-3-phenylpropanamide, 2h

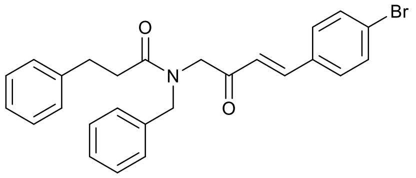

Following general procedure C, the product (75 mg) was isolated by FC (petrol/EtOAc 4:1) in 81% yield as a yellow oil, starting from *N*-benzyl-*N*-(2-oxoethyl)-3-phenylpropanamide (**1a**) (56 mg, 0.20 mmol) and 1-bromo-4-ethynylbenzene (36 mg, 0.24 mmol); **^{1}H-NMR** (400 MHz, $CDCl_3$) [the compound exists as two rotamers, in a ratio of 3:1] δ 7.49–7.37 (m, 3H, C(O)CH=C*H*, 2 × Ar*H*), 7.29–7.06 (m, 10H, 10 × Ar*H*), 7.05–6.97 (m, 2H, 2 × Ar*H*), 6.61 (d, *J* = 16.0, 1H, C(O)C*H*=CH), 6.51* (d, *J* = 16.0, 1H, C(O)C*H*=CH), 4.58* (s, 2H, NCH_2Ph), 4.51 (s, 2H, NCH_2Ph), 4.30 (s, 2H, NCH_2C(O)), 4.06* (s, 2H, NCH_2C(O)), 2.97–2.90 (m, 2H, PhCH_2CH$_2$), 2.74–2.68 (m, 2H, PhCH$_2$CH_2), 2.46–2.40* (m, 2H, PhCH$_2$CH_2); **^{13}C-NMR** (100 MHz, $CDCl_3$) (* denotes minor rotamer) δ 194.3, 193.9*, 173.0, 172.9*, 143.0*, 142.2, 141.3*, 141.2, 137.0*, 136.2, 133.3, 132.8*, 132.4*, 132.3, 129.9*, 129.8, 129.1, 128.8*, 128.6, 128.5, 127.9, 127.7*, 126.8*, 126.8, 126.2, 125.1*, 123.9, 122.4*, 55.2*, 53.6, 52.1, 50.1*, 35.2*, 34.8, 31.5*, 31.4; **IR** (film, cm^{-1}) 3027, 2922, 1643,

1612, 1585, 1486, 1451, 1402, 1186, 1069, 1008, 804, 731, 699; **LRMS** *m/z* (ESI^+) 484.0 ($[M(Br^{79})+Na]^+$, 100%); **HRMS** (ESI^+) calc. for $C_{26}H_{25}O_2N^{79}Br$ $[M+H]^+$: 462.1063, found: 462.1060.

(*E*)-*N*-benzyl-*N*-(4-(4-methoxyphenyl)-2-oxobut-3-en-1-yl)-3-phenylpropanamide, 2i

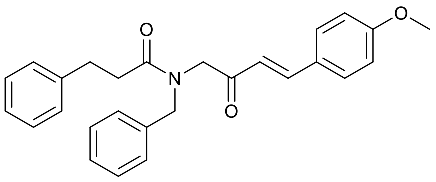

Following general procedure C, the product (60 mg) was isolated by FC (petrol/EtOAc 4:1) in 72% yield as a yellow oil, starting from *N*-benzyl-*N*-(2-oxoethyl)-3-phenylpropanamide (**1a**) (56 mg, 0.20 mmol) and 1-ethynyl-4-methoxybenzene (32 mg, 0.24 mmol); **^{1}H-NMR** (400 MHz, $CDCl_3$) [the compound exists as two rotamers, in a ratio of 2:1] δ 7.47 (t, J = 16.5, 1H, C(O)CH=C*H*), 7.40–7.31 (m, 2H, 2 × Ar*H*), 7.28–7.01 (m, 10H, 10 × Ar*H*), 6.83–6.76 (m, 2H, 2 × Ar*H*), 6.52 (d, J = 16.0, 1H, C(O)C*H*=CH), 6.43* (d, J = 16.0, 1H, C(O)C*H*=CH), 4.59* (s, 2H, NCH_2Ph), 4.51 (s, 2H, NCH_2Ph), 4.31 (s, 2H, $NCH_2C(O)$), 4.05* (s, 2H, $NCH_2C(O)$), 3.74* (s, 3H, OCH_3), 3.73 (s, 3H, OCH_3), 2.95 (t, J = 8.0, 2H, $PhCH_2CH_2$), 2.75–2.65 (m, 2H, $PhCH_2CH_2$), 2.48–2.41* (m, 2H, $PhCH_2CH_2$); **^{13}C-NMR** (100 MHz, $CDCl_3$) (* denotes minor rotamer) δ 194.3, 193.8*, 172.9, 172.9*, 162.2*, 161.8, 144.2*, 143.4, 141.3*, 141.3, 137.1*, 136.4, 130.4*, 130.2, 129.0, 128.7*, 128.5, 128.5, 127.8, 127.6*, 127.0*, 126.7, 126.5*, 126.1, 121.2, 119.6*, 114.6*, 114.5, 55.5*, 55.4, 54.9*, 53.2, 52.0, 50.0*, 35.1*, 34.8, 31.4*, 31.4; **IR** (film, cm^{-1}) 3028, 2931, 1645, 1596, 1572, 1511, 1452, 1423, 1253, 1171, 1090, 1028, 816, 737, 700; **LRMS** *m/z* (ESI^+) 436.2 ($[M+Na]^+$, 100%); **HRMS** (ESI^+) calc. for $C_{27}H_{28}O_3N$ $[M+H]^+$: 414.2063, found: 414.2051.

(*E*)-*N*-benzyl-*N*-(2-oxo-4-(thiophen-3-yl)but-3-en-1-yl)-3-phenylpropanamide, 2j

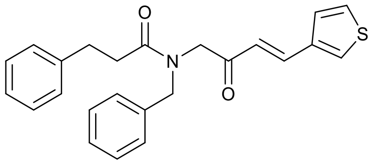

Following general procedure C, the product (70 mg) was isolated by FC (petrol/EtOAc 4:1) in 89% yield as a yellow oil, starting from *N*-benzyl-*N*-(2-oxoethyl)-3-phenylpropanamide (**1a**) (56 mg, 0.20 mmol) and 3-ethynylthiophene (23 μL, 0.24 mmol); **^{1}H-NMR** (400 MHz, $CDCl_3$) [the compound exists as two rotamers, in a ratio of 2.5:1] δ 7.57–7.38 (m, 2H, C(O)CH=C*H*, 1 × Ar*H*),

7.29–7.06 (m, 10H, 10 × Ar*H*), 7.05–7.00 (m, 2H, 2 × Ar*H*), 6.45 (d, *J* = 16.0, 1H, C(O)C*H*=CH), 6.36* (d, *J* = 16.0, 1H, C(O)C*H*=CH), 4.58* (s, 2H, NC*H*$_2$Ph), 4.51 (s, 2H, NC*H*$_2$Ph), 4.29 (s, 2H, NC*H*$_2$C(O)), 4.04* (s, 2H, NC*H*$_2$C(O)), 2.99–2.90 (m, 2H, PhC*H*$_2$CH$_2$), 2.75–2.65 (m, 2H, PhCH$_2$C*H*$_2$), 2.47–2.38* (m, 2H, PhCH$_2$C*H*$_2$); **^{13}C-NMR** (100 MHz, CDCl$_3$) (* denotes minor rotamer) δ 194.7, 194.2*, 173.0, 172.9*, 141.3*, 141.3, 137.7*, 137.6, 137.3*, 137.1*, 137.0, 136.3, 130.1*, 129.4, 129.0, 128.7*, 128.6, 128.5, 127.9, 127.6*, 127.4*, 127.2, 126.7, 126.2*, 125.2, 125.1*, 123.2, 121.7*, 54.9*, 53.3, 52.1, 50.0*, 35.1*, 34.8, 31.5*, 31.4; **IR** (film, cm^{-1}) 3027, 2923, 1643, 1604, 1495, 1451, 1194, 1088, 968, 779, 732, 699; **LRMS** *m/z* (ESI$^+$) 412.2 ([M+Na]$^+$, 100%); **HRMS** (ESI$^+$) calc. for $C_{24}H_{24}O_2NS$ [M+H]$^+$: 390.1533, found: 390.1537.

(*E*)-*N*-benzyl-*N*-(5,5-dimethyl-2-oxohex-3-en-1-yl)-3-phenylpropanamide, 2k

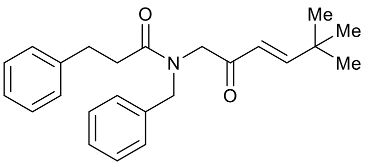

Following general procedure C, the product (69 mg) was isolated by FC (petrol/EtOAc 4:1) in 95% yield as a white amorphous solid, starting from *N*-benzyl-*N*-(2-oxoethyl)-3-phenylpropanamide (**1a**) (56 mg, 0.20 mmol) and 3,3-dimethylbut-1-yne (29 µL, 0.24 mmol); **^{1}H-NMR** (400 MHz, CDCl$_3$) [the compound exists as two rotamers, in a ratio of 3:1] δ 7.38–7.26 (m, 5H, 5 × Ar*H*), 7.26–7.18 (m, 3H, 3 × Ar*H*), 7.17–7.10 (m, 2H, 2 × Ar*H*), 6.90 (t, *J* = 16.0, 1H, C(O)CH=C*H*), 6.05 (d, *J* = 16.0, 1H, C(O)C*H*=CH), 5.98* (d, *J* = 16.0, 1H, C(O)C*H*=CH), 4.66* (s, 2H, NC*H*$_2$Ph), 4.60 (s, 2H, NC*H*$_2$Ph), 4.35 (s, 2H, NC*H*$_2$C(O)), 4.09* (s, 2H, NC*H*$_2$C(O)), 3.09–3.01 (m, 2H, PhC*H*$_2$CH$_2$), 2.84–2.76 (m, 2H, PhCH$_2$C*H*$_2$), 2.55–2.49* (m, 2H, PhCH$_2$C*H*$_2$), 1.09 (s, 9H, C(C*H*$_3$)$_3$), 1.08* (s, 9H, C(C*H*$_3$)$_3$); **^{13}C-NMR** (100 MHz, CDCl$_3$) (* denotes minor rotamer) δ 194.9, 194.4*, 173.0, 172.9*, 159.2*, 158.2, 141.4*, 141.3, 137.2*, 136.4, 129.0, 128.7*, 128.6*, 128.5, 128.5, 128.4*, 127.8, 127.6*, 126.7, 126.2*, 126.2, 122.9, 121.9*, 54.5*, 53.0, 52.0, 49.9*, 35.1*, 34.8, 34.1*, 34.0, 31.5*, 31.4, 28.6, 28.6*; **IR** (film, cm^{-1}) 2980, 1687, 1648, 1495, 1452, 1207, 1167, 1095, 1077, 945, 735, 699; **LRMS** *m/z* (ESI$^+$) 386.2 ([M+Na]$^+$, 100%); **HRMS** (ESI$^+$) calc. for $C_{24}H_{30}O_2N$ [M+H]$^+$: 364.2282, found: 364.2272; **m.p.** (°C): 78–80.

(*E*)-*N*-benzyl-*N*-(2-oxo-4-(trimethylsilyl)but-3-en-1-yl)-3-phenylpropanamide, 2l

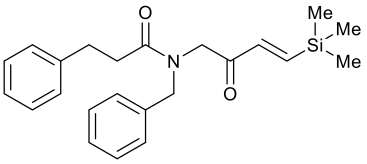

Following general procedure C, the product (65 mg) was isolated by FC (petrol/EtOAc 4:1) in 86% yield as a yellow oil, starting from *N*-benzyl-*N*-(2-oxoethyl)-3-phenylpropanamide (**1a**) (56 mg, 0.20 mmol) and ethynyltrimethylsilane (33 µL, 0.24 mmol); **^{1}H-NMR** (400 MHz, $CDCl_3$) [the compound exists as two rotamers, in a ratio of 3:1] δ 7.23–7.11 (m, 5H, C(O)CH=C*H*, 4 × Ar*H*), 7.10–7.01 (m, 4H, 4 × Ar*H*), 7.00–6.91 (m, 2H, 2 × Ar*H*), 6.36 (d, *J* = 19.0, 1H, C(O)C*H*=CH), 6.29* (d, *J* = 19.0, 1H, C(O)C*H*=CH), 4.50* (s, 2H, NCH_2Ph), 4.44 (s, 2H, NCH_2Ph), 4.25 (s, 2H, NCH_2C(O)), 4.01* (s, 2H, NCH_2C(O)), 2.94–2.84 (m, 2H, PhCH_2CH_2), 2.69–2.60 (m, 2H, PhCH_2CH_2), 2.39–2.31* (m, 2H, PhCH_2CH_2), 0.00 (s, 9H, Si(CH_3)$_3$); **^{13}C-NMR** (100 MHz, $CDCl_3$) (* denotes minor rotamer) δ 194.1, 193.6*, 173.0, 172.9*, 149.8*, 148.5, 141.4*, 141.3, 139.7, 138.8*, 137.2*, 136.4, 129.0, 128.7*, 128.6*, 128.6, 128.6, 128.4*, 127.8, 127.6*, 126.7, 126.2*, 126.2, 53.9*, 52.6, 52.1, 49.9*, 35.2*, 34.8, 31.5*, 31.4, -1.8; **IR** (film, cm^{-1}) 3028, 2954, 1691, 1648, 1495, 1452, 1248, 1206, 1156, 1077, 994, 840, 733, 698; **LRMS** *m/z* (ESI$^+$) 402.2 ([M+Na]$^+$, 100%); **HRMS** (ESI$^+$) calc. for $C_{23}H_{30}O_2NSi$ [M+H]$^+$: 380.2051, found: 380.2040.

(*E*)-*N*-benzyl-*N*-(7-chloro-2-oxohept-3-en-1-yl)-3-phenylpropanamide, 2m

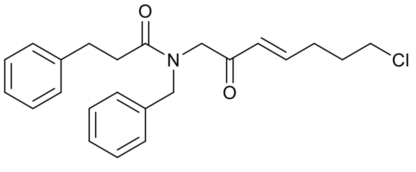

Following general procedure C, the product (56 mg) was isolated by FC (petrol/EtOAc 4:1) in 73% yield as a pale yellow oil, starting from *N*-benzyl-*N*-(2-oxoethyl)-3-phenylpropanamide (**1a**) (56 mg, 0.20 mmol) and 5-chloropent-1-yne (25 µL, 0.24 mmol); **^{1}H-NMR** (400 MHz, $CDCl_3$) [the compound exists as two rotamers, in a ratio of 3:1] δ 7.39–7.25 (m, 5H, 5 × Ar*H*), 7.25–7.16 (m, 3H, 3 × Ar*H*), 7.15–7.05 (m, 2H, 2 × Ar*H*), 6.94–6.79 (m, 1H, C(O)CH=C*H*CH_2), 6.17 (dt, *J* = 16.0, 1.5, 1H, C(O)C*H*=CHCH_2), 6.10* (dt, *J* = 16.0, 1.5, 1H, C(O)C*H*=CHCH_2), 4.64* (s, 2H, NCH_2Ph), 4.58 (s, 2H, NCH_2Ph), 4.31 (s, 2H, NCH_2C(O)), 4.06* (s, 2H, NCH_2C(O)), 3.57–3.51 (m, 2H, CH_2CH_2CH_2Cl), 3.09–2.99 (m, 2H, PhCH_2CH_2), 2.86–2.75 (m, 2H, PhCH_2CH_2), 2.55–2.46* (m, 2H, PhCH_2CH_2), 2.44–2.27 (m, 2H, CH_2CH_2CH_2Cl), 1.99–1.85 (m, 2H, CH_2CH_2CH_2Cl); **^{13}C-NMR** (100 MHz, $CDCl_3$) (* denotes minor rotamer) δ 194.2, 193.7*, 173.0, 172.9*, 147.4*, 146.4, 141.4*, 141.3, 137.1*, 136.3, 129.0, 128.7*, 128.6, 128.5, 128.5, 127.9, 127.6*, 127.4*, 126.7, 126.2*,

126.2, 54.5*, 53.0, 52.1, 50.0*, 44.0*, 43.9, 35.1, 34.8,* 31.5*, 31.4, 30.7, 30.6*, 29.7*, 29.7; **IR** (film, cm^{-1}) 3027, 2922, 1686, 1640, 1495, 1451, 1205, 1107, 1076, 978, 735, 699; **LRMS** *m/z* (ESI^+) 406.2 ($[M(Cl^{35})+Na]^+$, 100%); **HRMS** (ESI^+) calc. for $C_{23}H_{27}O_2N^{35}Cl$ $[M+H]^+$: 384.1725, found: 384.1723.

(*E*)-*N*-benzyl-*N*-(6-hydroxy-2-oxohept-3-en-1-yl)-3-phenylpropanamide, 2n

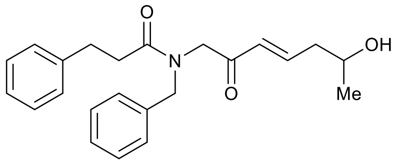

Following general procedure C, the product (31 mg) was isolated by FC (petrol/EtOAc 4:1) in 42% yield as a pale yellow oil, starting from *N*-benzyl-*N*-(2-oxoethyl)-3-phenylpropanamide (**1a**) (56 mg, 0.20 mmol) and pent-4-yn-2-ol (22 µL, 0.24 mmol); **^{1}H-NMR** (400 MHz, $CDCl_3$) [the compound exists as two rotamers, in a ratio of 3:1] δ 7.29–7.16 (m, 5H, 5 × Ar*H*), 7.15–7.06 (m, 3H, 3 × Ar*H*), 7.05–6.99 (m, 2H, 2 × Ar*H*), 6.90–6.74 (m, 1H, C(O)CH=C*H*), 6.10 (dt, *J* = 16.0, 1.5, 1H, C(O)C*H*=CH), 6.03* (dt, *J* = 16.0, 1.5, 1H, C(O)C*H*=CH), 4.54* (s, 2H, NCH_2Ph), 4.49 (s, 2H, NCH_2Ph), 4.23 (s, 2H, $NCH_2C(O)$), 3.99* (s, 2H, $NCH_2C(O)$), 3.92–3.81 (m, 1H, $CH{=}CHCH_2CH(OH)CH_3$), 2.99–2.88 (m, 2H, $PhCH_2CH_2$), 2.72–2.65 (m, 2H, $PhCH_2CH_2$), 2.45–2.36* (m, 2H, $PhCH_2CH_2$), 2.31–2.20 (m, 2H, $CH{=}CHCH_2$), 1.81 (br s, 1H, $CH(OH)CH_3$), 1.69* (br s, 1H, $CH(OH)CH_3$), 1.17–1.13 (m, 3H, $CH(OH)CH_3$); **^{13}C-NMR** (100 MHz, $CDCl_3$) (* denotes minor rotamer) δ 194.3, 193.8*, 173.1, 173.0*, 145.7*, 144.7, 141.4*, 141.3, 137.1*, 136.3, 130.0, 129.1, 128.9*, 128.7*, 128.6, 128.6, 128.5*, 127.9, 127.7*, 126.8*, 126.7, 126.3, 66.8, 66.7*, 54.4*, 53.0, 52.2, 50.0*, 42.4, 42.2*, 35.2*, 34.9, 31.5*, 31.4, 23.6*, 23.5; **IR** (film, cm^{-1}) 3412, 3028, 2925, 1686, 1631, 1495, 1452, 1209, 1077, 736, 700; **LRMS** *m/z* (ESI^+) 388.2 ($[M+Na]^+$, 100%); **HRMS** (ESI^+) calc. for $C_{23}H_{28}O_3N$ $[M+H]^+$: 366.2063, found: 366.2060.

(*E*)-*N*-benzyl-*N*-(5,5-diethoxy-2-oxopent-3-en-1-yl)-3-phenylpropanamide, 2o

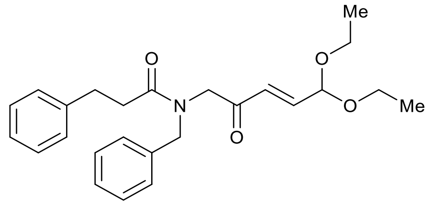

Following general procedure C, the product (23 mg) was isolated by FC (petrol/EtOAc 4:1) in 28% yield as a yellow oil, starting from *N*-benzyl-*N*-(2-oxoethyl)-3-phenylpropanamide (**1a**) (56 mg, 0.20 mmol) and 3,3-diethoxyprop-1-yne (34 µL, 0.24 mmol); **^{1}H-NMR** (400 MHz, $CDCl_3$) [the compound exists as two rotamers, in a ratio of 2:1] δ 7.28–7.17 (m, 5H, 5 × Ar*H*), 7.15–7.06 (m, 3H, 3 × Ar*H*), 7.05–6.97 (m, 2H, 2 × Ar*H*), 6.65–6.55 (m, 1H, C(O)CH=C*H*), 6.31 (dd, *J* = 16.0, 1.5, 1H, C(O)C*H*=CH), 6.25* (dd, *J* = 16.0, 1.5, 1H, C(O)C*H*=CH), 4.95 (dd, *J* = 4.0, 1.5, 1H, C*H*(OCH_2CH_3)$_2$), 4.93* (dd, *J* = 4.0, 1.5, 1H, C*H*(OCH_2CH_3)$_2$), 4.55* (s, 2H, NCH_2Ph), 4.48 (s, 2H, NCH_2Ph), 4.26 (s, 2H, NCH_2C(O)), 4.01* (s, 2H, NCH_2C(O)), 3.60–3.49 (m, 2H, CHOC$H_2$$CH_3$), 3.48–3.37 (m, 2H, CHOC$H_2$$CH_3$), 2.98–2.89 (m, 2H, PhC$H_2$$CH_2$), 2.73–2.66 (m, 2H, PhCH$_2$CH_2), 2.44–2.35* (m, 2H, PhCH$_2$CH_2), 1.14 (t, *J* = 7.0, 6H, C*H*(OCH_2CH_3)$_2$); **^{13}C-NMR** (100 MHz, $CDCl_3$) (* denotes minor rotamer) δ 194.4, 194.0*, 173.1, 172.9*, 143.3*, 142.5, 141.3, 137.0*, 136.2, 129.1, 129.0*, 128.8*, 128.6, 128.6, 128.6, 128.5*, 128.0*, 127.9, 127.7*, 126.8, 126.3*, 126.3, 99.4, 99.2*, 61.9*, 61.7, 54.7*, 53.2, 52.1, 49.9*, 35.2*, 34.9, 31.5*, 31.4, 15.3; **IR** (film, cm^{-1}) 2979, 1695, 1647, 1495, 1452, 1133, 1055, 735, 700; **LRMS** *m/z* (ESI^+) 432.2 ($[M+Na]^+$, 100%); **HRMS** (ESI^+) calc. for $C_{25}H_{31}O_4NNa$ $[M+Na]^+$: 432.2145, found: 432.2143.

(*E*)-*N*-benzyl-*N*-(8-(1,3-dioxoisoindolin-2-yl)-2-oxooct-3-en-1-yl)-3-phenylpropanamide, 2p

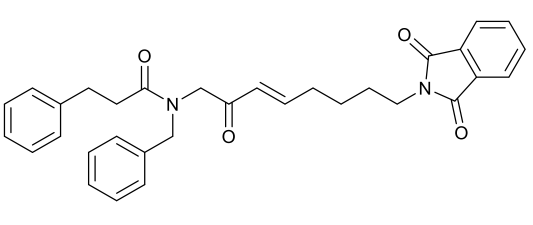

Following general procedure C, the product (71 mg) was isolated by FC (petrol/EtOAc 3:1) in 70% yield as a colourless oil, starting from *N*-benzyl-*N*-(2-oxoethyl)-3-phenylpropanamide (**1a**) (56 mg, 0.20 mmol) and 2-(hex-5-yn-1-yl)isoindoline-1,3-dione (54 mg, 0.24 mmol); **^{1}H-NMR** (400 MHz, $CDCl_3$) [the compound exists as two rotamers, in a ratio of 2.5:1] δ 7.87–7.79 (m, 2H, 2 × Ar*H*), 7.75–7.67 (m, 2H, 2 × Ar*H*), 7.37–7.22 (m, 5H, 5 × Ar*H*), 7.21–7.14

(m, 3H, 3 × Ar*H*), 7.12–7.07 (m, 2H, 2 × Ar*H*), 6.94–6.77 (m, 1H, C(O)CH=C*H*), 6.10 (dt, *J* = 16.0, 1.5, 1H, C(O)C*H*=CH), 6.04* (dt, *J* = 16.0, 1.5, 1H, C(O)C*H*=CH), 4.61* (s, 2H, NCH_2Ph), 4.56 (s, 2H, NCH_2Ph), 4.29 (s, 2H, NCH_2C(O)), 4.04* (s, 2H, NCH_2C(O)), 3.71–3.64 (m, 2H, $(CH_2)_3CH_2N$), 3.07–2.95 (m, 2H, PhCH_2CH$_2$), 2.81–2.71 (m, 2H, PhCH$_2$CH_2), 2.64–2.53* (m, 2H, PhCH$_2$CH_2), 2.51–2.43* (m, 2H, C(O)CH=CHCH_2), 2.30–2.19 (m, 2H, C(O)CH=CHCH_2), 1.79–1.66 (m, 2H, C(O)CH=CH$(CH_2)_2$CH_2), 1.56–1.41 (m, 2H, C(O)CH=CHCH$_2$CH_2); **^{13}C-NMR** (100 MHz, $CDCl_3$) δ 194.4, 173.1, 168.5, 148.8*, 147.9, 141.3, 136.4, 134.1, 134.1*, 134.0*, 132.3, 132.2*, 132.2, 129.1*, 128.7*, 128.6, 128.5*, 128.0, 127.9, 127.6*, 127.2*, 126.8, 126.2, 123.4, 123.3*, 123.3*, 54.4*, 52.9, 52.1, 50.0*, 38.0*, 37.6, 37.5*, 35.2*, 34.9, 32.2, 31.5*, 31.4, 28.2, 28.2*, 25.3, 25.1*; **IR** (film, cm^{-1}) 3028, 2935, 1706, 1643, 1495, 1436, 1369, 1361, 1207, 720, 700; **LRMS** *m/z* (ESI$^+$) 531.2 ([M+Na]$^+$, 100%); **HRMS** (ESI$^+$) calc. for $C_{32}H_{32}O_4N_2Na$ [M+Na]$^+$: 531.2254, found: 531.2257.

(*E*)-*N*-benzyl-*N*-(2-oxo-3,4-diphenylbut-3-en-1-yl)-3-phenylpropanamide, 2q

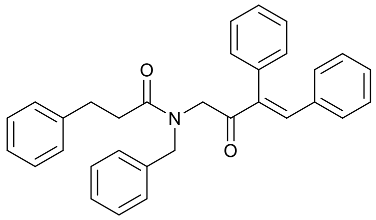

Following general procedure C, the product (71 mg) was isolated by FC (petrol/EtOAc 4:1) in 77% yield as a white amorphous solid, starting from *N*-benzyl-*N*-(2-oxoethyl)-3-phenylpropanamide (**1a**) (56 mg, 0.20 mmol) and 1,2-diphenylethyne (36 mg, 0.24 mmol); **^{1}H-NMR** (400 MHz, $CDCl_3$) [the compound exists as two rotamers, in a ratio of 3:1] δ 7.60 (s, 1H, C(O)C=C*H*), 7.49* (s, 1H, C(O)C=C*H*), 7.37–6.87 (m, 20H, 20 × Ar*H*), 4.54* (s, 2H, NCH_2Ph), 4.50 (s, 2H, NCH_2Ph), 4.23 (s, 2H, NCH_2C(O)), 3.92* (s, 2H, NCH_2C(O)), 2.98–2.88 (m, 2H, PhCH_2CH$_2$), 2.73–2.62 (m, 2H, PhCH$_2$CH_2), 2.43–2.34* (m, 2H, PhCH$_2$CH_2); **^{13}C-NMR** (100 MHz, $CDCl_3$) δ 195.8, 195.4, 173.1, 173.0*, 141.5*, 141.4, 139.4*, 139.1, 138.8, 137.2*, 136.5, 136.1, 135.8*, 134.5, 134.1*, 131.2*, 131.1, 129.9*, 129.7, 129.6*, 129.5*, 129.4*, 129.3, 129.0, 128.8*, 128.7*, 128.6, 128.5, 128.5*, 128.4, 128.3, 127.8, 127.6*, 126.8, 126.2, 54.3*, 53.3, 52.2, 50.0*, 35.2*, 35.0, 31.6*, 31.4; **IR** (film, cm^{-1}) 3026, 2926, 1689, 1646, 1607, 1494, 1447, 1195, 1131, 1100, 1075, 755, 732, 697; **LRMS** *m/z* (ESI$^+$) 460.2 ([M+H]$^+$, 100%); **HRMS** (ESI$^+$) calc. for $C_{32}H_{30}O_2N$ [M+H]$^+$: 460.2271, found: 460.2274; **m.p.** (°C): 89–90.

(*E*)-*N*-benzyl-*N*-(2-oxo-3-propylhept-3-en-1-yl)-3-phenylpropanamide, 2r

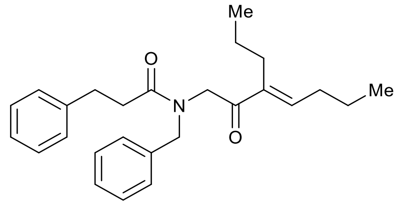

Following general procedure C, the product (13 mg) was isolated by FC (petrol/EtOAc 4:1) in 17% yield as a pale yellow oil, starting from *N*-benzyl-*N*-(2-oxoethyl)-3-phenylpropanamide (**1a**) (56 mg, 0.20 mmol) and oct-4-yne (27 µL, 0.24 mmol); **^{1}H-NMR** (400 MHz, $CDCl_3$) [the compound exists as two rotamers, in a ratio of 2.5:1] *δ* 7.38–7.23 (m, 5H, 5 × Ar*H*), 7.22–7.15 (m, 3H, 3 × Ar*H*), 7.14–7.03 (m, 2H, 2 × Ar*H*), 6.55 (t, *J* = 7.5, 1H, C(O)C=C*H*), 6.35* (t, *J* = 7.5, 1H, C(O)C=C*H*), 4.60* (s, 2H, NCH_2Ph), 4.57 (s, 2H, NCH_2Ph), 4.48 (s, 2H, NCH_2C(O)), 4.20* (s, 2H, NCH_2C(O)), 3.08–2.97 (m, 2H, PhCH_2CH$_2$), 2.82–2.69 (m, 2H, PhCH$_2$CH_2), 2.48–2.39* (m, 2H, PhCH$_2$CH_2), 2.32–2.13 (m, 4H, CH_2C=CHCH_2), 1.53–1.20 (m, 4H, CH_2CH$_2$C=CH CH$_2$CH_2), 1.01–0.79 (m, 6H, 2 × CH_3); **^{13}C-NMR** (100 MHz, $CDCl_3$) *δ* 195.6, 194.9*, 177.2*, 173.1, 143.4, 141.4, 140.7, 136.7, 129.0, 128.7*, 128.6*, 128.6, 128.6, 127.8, 126.8, 126.2, 52.1*, 51.9, 50.9, 50.0*, 35.2*, 35.0, 31.7*, 31.5, 31.0, 31.0*, 28.0, 27.9*, 22.6, 22.5*, 22.2, 22.2*, 14.3, 14.2*, 14.1, 14.1*; **IR** (film, cm^{-1}) 3028, 2959, 2930, 2870, 1680, 1650, 1495, 1453, 1205, 1104, 734, 699; **LRMS** *m/z* (ESI$^+$) 414.2 ([M+Na]$^+$, 100%); **HRMS** (ESI$^+$) calc. for $C_{26}H_{33}O_2NNa$ [M+Na]$^+$: 414.2403, found: 414.2398.

(*E*)-*N*-methyl-*N*-(2-oxo-4-phenylbut-3-en-1-yl)-3-phenylpropanamide, 2s

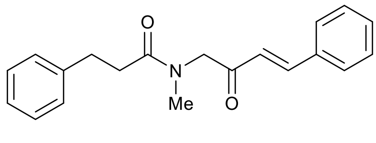

Following general procedure C, the product (52 mg) was isolated by FC (petrol/EtOAc 4:1) in 85% yield as a yellow oil, starting from *N*-methyl-*N*-(2-oxoethyl)-3-phenylpropanamide (**1s**) (41 mg, 0.20 mmol) and ethynylbenzene (26 µL, 0.24 mmol); **^{1}H-NMR** (400 MHz, $CDCl_3$) [the compound exists as two rotamers, in a ratio of 5:1] *δ* 7.60–7.53 (m, 1H, C(O)CH=C*H*), 7.49–7.41 (m, 2H, 2 × Ar*H*), 7.35–7.27 (m, 3H, 3 × Ar*H*), 7.24–7.05 (m, 5H, 5 × Ar*H*), 6.70–6.60 (m, 1H, C(O)C*H*=CH), 4.39 (s, 2H, NCH_2C(O)), 4.19* (s, 2H, NCH_2C(O)), 3.16–2.85 (m, 5H, NCH_3, PhCH_2CH$_2$), 2.74–2.57 (m, 2H, PhCH$_2$CH_2), 2.45–2.30* (m, 2H, PhCH$_2$CH_2); **^{13}C-NMR** (100 MHz, $CDCl_3$) (* denotes minor rotamer) *δ* 194.7, 194.0*, 172.8, 172.8*, 144.6*, 143.8, 141.4, 141.4*, 134.3, 133.9*, 131.3*, 130.8, 129.2*, 129.1, 128.6*, 128.6, 128.6*, 128.5, 126.2, 123.3, 121.9*, 58.2*, 56.0, 36.6, 35.3*, 35.1, 35.0*, 31.4*, 31.2; **IR** (film, cm^{-1}) 3026, 2924, 1678, 1642, 1612,

1494, 1451, 1408, 1192, 1090, 980, 750, 699; **LRMS** *m/z* (ESI^+) 330.2 ($[M+Na]^+$, 100%); **HRMS** (ESI^+) calc. for $C_{20}H_{22}O_2N$ $[M+H]^+$: 308.1645, found: 308.1647.

(*E*)-*N*-(2-oxo-4-phenylbut-3-en-1-yl)-*N*,3-diphenylpropanamide, 2t

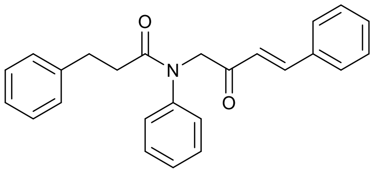

Following general procedure C, the product (60 mg) was isolated by FC (petrol/EtOAc 4:1) in 81% yield as a yellow oil, starting from *N*-(2-oxoethyl)-*N*,3-diphenylpropanamide (**1t**) (53 mg, 0.20 mmol) and ethynylbenzene (26 µL, 0.24 mmol); **^{1}H-NMR** (400 MHz, $CDCl_3$) δ 7.57 (d, *J* = 16.0, 1H, C(O)CH=C*H*), 7.47–7.40 (m, 2H, 2 × Ar*H*), 7.34–7.19 (m, 6H, 6 × Ar*H*), 7.18–7.11 (m, 4H, 4 × Ar*H*), 7.10–7.03 (m, 1H, Ar*H*), 7.03–6.95 (m, 2H, 2 × Ar*H*), 6.70 (d, *J* = 16.0, 1H, C(O)C*H*=CH), 4.63 (s, 2H, $NCH_2C(O)$), 2.91–2.82 (m, 2H, $PhCH_2CH_2$), 2.44–2.34 (m, 2H, $PhCH_2CH_2$); **^{13}C-NMR** (100 MHz, $CDCl_3$) δ 193.8, 172.5, 143.6, 143.0, 141.3, 134.4, 130.8, 129.8, 129.0, 128.5, 128.5, 128.4, 128.3, 128.3, 126.1, 123.3, 58.0, 35.9, 31.6; **IR** (film, cm^{-1}) 3026, 2980, 1654, 1612, 1595, 1494, 1450, 1413, 1385, 1188, 1102, 1072, 749, 699; **LRMS** *m/z* (ESI^+) 392.2 ($[M+Na]^+$, 100%); **HRMS** (ESI^+) calc. for $C_{25}H_{24}O_2N$ $[M+H]^+$: 370.1802, found: 370.1798.

(*E*)-3-phenyl-1-(1-(3-phenylpropanoyl)pyrrolidin-2-yl)prop-2-en-1-one, 2u

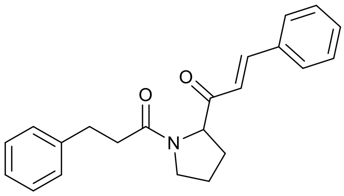

Following general procedure C, the product (60 mg) was isolated by FC (petrol/EtOAc 2:1) in 90% yield as a yellow oil, starting from 1-(3-phenylpropanoyl)pyrrolidine-2-carbaldehyde (**1u**) (46 mg, 0.20 mmol) and ethynylbenzene (26 µL, 0.24 mmol); **^{1}H-NMR** (400 MHz, $CDCl_3$) [the compound exists as two rotamers, in a ratio of 4:1] δ 7.72 (d, *J* = 16.0, 1H, C(O)CH=C*H*), 7.68–7.65* (m, 1H, 1 × Ar*H*), 7.62–7.52 (m, 2H, 2 × Ar*H*), 7.48–7.32 (m, 3H, 3 × Ar*H*), 7.36–7.11 (m, 5H, 5 × Ar*H*), 6.88 (d, *J* = 16.0, 1H, C(O)C*H*=CH), 6.79* (d, *J* = 16.0, 1H, C(O)C*H*=CH), 4.95 (dd, *J* = 9.0, 4.0, 1H, C*H*C(O)CH=CH), 4.48* (dd, *J* = 9.0, 4.0, 1H, C*H*C(O)CH=CH), 3.77–3.67* (m, 2H, $NCH_AH_BCH_2$), 3.68–3.57 (m, 1H, $NCH_AH_BCH_2$), 3.50–3.41 (m, 1H, $NCH_AH_BCH_2$), 3.10–2.89

(m, 2H, PhC*H*$_2$CH$_2$), 2.77–2.57 (m, 2H, PhCH$_2$C*H*$_2$), 2.47–2.23* (m, 2H, PhCH$_2$C*H*$_2$), 2.24–2.08* (m, 4H, NCH$_2$C*H*$_2$C*H*$_2$), 2.11–1.78 (m, 4H, NCH$_2$C*H*$_2$C*H*$_2$); **^{13}C-NMR** (100 MHz, CDCl$_3$) (* denotes minor rotamer) δ 197.7, 171.4*, 170.9, 145.3*, 143.8, 141.5, 141.3*, 135.9*, 135.1, 134.6, 134.0*, 131.2*, 130.6, 129.1*, 128.9, 128.7*, 128.5, 128.5, 128.4*, 127.8*, 126.1, 123.2, 120.6*, 65.3*, 63.6, 47.3, 46.9*, 36.6*, 36.4, 31.3*, 31.1*, 30.9, 28.6, 24.7, 22.7*; **IR** (film, cm^{-1}) 3026, 2971, 1636, 1609, 1575, 1495, 1428, 1331, 1176, 1101, 1075, 982, 757, 699; **LRMS** *m/z* (ESI$^+$) 356.2 ([M+Na]$^+$, 100%); **HRMS** (ESI$^+$) calc. for $C_{22}H_{24}O_2N$ [M+H]$^+$: 334.1802, found: 334.1803.

(*E*)-*N*-(2-oxo-4-phenylbut-3-en-1-yl)-3-phenylpropanamide, 2v

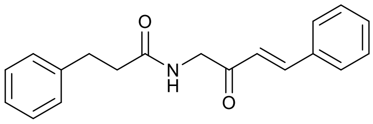

Following general procedure C, the product (47 mg) was isolated by FC (petrol/EtOAc 1:1) in 80% yield as a white amorphous solid, starting from *N*-(2-oxoethyl)-3-phenylpropanamide (**1v**) (38 mg, 0.20 mmol) and ethynylbenzene (26 µL, 0.24 mmol); **^{1}H-NMR** (400 MHz, CDCl$_3$) δ 7.68 (d, *J* = 16.0, 1H, C(O)CH=C*H*), 7.60–7.51 (m, 2H, 2 × Ar*H*), 7.46–7.38 (m, 3H, 3 × Ar*H*), 7.33–7.26 (m, 2H, 2 × Ar*H*), 7.25–7.17 (m, 3H, 3 × Ar*H*), 6.74 (d, *J* = 16.0, 1H, C(O)C*H*=CH), 6.44 (t, *J* = 4.5, 1H, C(O)N*H*), 4.44 (d, *J* = 4.5, 2H, NC*H*$_2$C(O)), 3.05–2.96 (m, 2H, PhC*H*$_2$CH$_2$), 2.66–2.56 (m, 2H, PhCH$_2$C*H*$_2$); **^{13}C-NMR** (100 MHz, CDCl$_3$) δ 194.1, 172.3, 144.6, 140.9, 134.0, 131.3, 129.2, 128.7, 128.6, 128.4, 126.4, 123.2, 47.9, 38.2, 31.7; **IR** (film, cm^{-1}) 3352, 3028, 1642, 1613, 1516, 1450, 1353, 1077, 980, 746, 690; **LRMS** *m/z* (ESI$^+$) 316.2 ([M+Na]$^+$, 100%); **HRMS** (ESI$^+$) calc. for $C_{19}H_{19}O_2NNa$ [M+Na]$^+$: 316.1308, found: 316.1311; **m.p.** (°C): 119–121.

(*E*)-*N*-(3-oxo-1,5-diphenylpent-4-en-2-yl)-3-phenylpropanamide, 2w

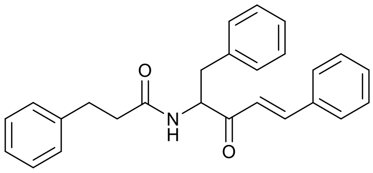

Following general procedure C, the product (67 mg) was isolated by FC (petrol/EtOAc 4:1) in 87% yield as a white amorphous solid, starting from *N*-(1-oxo-3-phenylpropan-2-yl)-3-phenylpropanamide (**1w**) (56 mg, 0.20 mmol) and ethynylbenzene (26 µL, 0.24 mmol); **^{1}H-NMR** (400 MHz, CDCl$_3$) δ 7.66 (d, *J* = 16.0, 1H, C(O)CH=C*H*), 7.56–7.48 (m, 2H, 2 × Ar*H*), 7.48–7.38 (m, 3H, 3 × Ar*H*), 7.34–7.19 (m, 8H, 8 × Ar*H*), 7.07–7.00 (m, 2H, 2 × Ar*H*), 6.72 (d, *J* = 16.0, 1H, C(O)C*H*=CH), 6.31

(t, J = 6.5, 1H, C(O)NH), 5.25 (q, J = 6.0, 1H, C(O)NHCH), 3.15 (d, J = 6.0, 2H, NHCHCH_2), 3.06–2.93 (m, 2H, PhCH_2CH$_2$), 2.66–2.48 (m, 2H, PhCH$_2$CH_2); **^{13}C-NMR** (100 MHz, CDCl$_3$) δ 196.9, 171.6, 144.9, 140.8, 136.0, 134.2, 131.2, 129.7, 129.1, 128.7, 128.7, 128.6, 128.5, 127.1, 126.4, 123.1, 57.8, 38.4, 38.2, 31.6.; **IR** (film, cm^{-1}) 3298, 3023, 2980, 1694, 1641, 1613, 1534, 1495, 1449, 752, 697; **LRMS** *m/z* (ESI$^+$) 406.2 ([M+Na]$^+$, 100%); **HRMS** (ESI$^+$) calc. for $C_{26}H_{25}O_2NNa$ [M+Na]$^+$: 406.1777, found: 406.1779; **m.p.** (°C): 194–196.

(*E*)-*N*-(3-oxo-5-phenylpent-4-en-2-yl)-3-phenylpropanamide, 2x

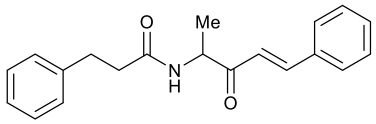

Following general procedure C, the product (42 mg) was isolated by FC (petrol/EtOAc 4:1) in 68% yield as a white amorphous solid, starting from *N*-(1-oxopropan-2-yl)-3-phenylpropanamide (**1x**) (41 mg, 0.20 mmol) and ethynylbenzene (26 µL, 0.24 mmol); **^{1}H-NMR** (400 MHz, CDCl$_3$) δ 7.64 (d, J = 16.0, 1H, C(O)CH=CH), 7.52–7.45 (m, 2H, 2 × ArH), 7.38–7.28 (m, 3H, 3 × ArH), 7.24–7.16 (m, 2H, 2 × ArH), 7.16–7.07 (m, 3H, 3 × ArH), 6.70 (d, J = 16.0, 1H, C(O)CH=CH), 6.36 (d, J = 7.0, 1H, NH), 4.89 (p, J = 7.0, 1H, NHCH), 2.96–2.85 (m, 2H, PhCH_2CH$_2$), 2.55–2.41 (m, 2H, PhCH$_2$CH_2), 1.29 (d, J = 7.0, 3H, CH_3); **^{13}C-NMR** (100 MHz, CDCl$_3$) δ 198.1, 171.6, 145.0, 140.8, 134.2, 131.2, 129.2, 128.7, 128.6, 128.5, 126.4, 122.4, 52.6, 38.5, 31.8, 18.7; **IR** (film, cm^{-1}) 3292, 3027, 2930, 1644, 1607, 1532, 1495, 1449, 1069, 980, 760, 698; **LRMS** *m/z* (ESI$^+$) 330.2 ([M+Na]$^+$, 100%); **HRMS** (ESI$^+$) calc. for $C_{20}H_{21}O_2NNa$ [M+Na]$^+$: 330.1464, found: 330.1461; **m.p.** (°C): 94–96.

(*E*)-*N*-(6-methyl-3-oxo-1-phenylhept-1-en-4-yl)-3-phenylpropanamide, 2y

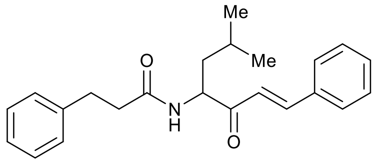

Following general procedure C, the product (47 mg) was isolated by FC (petrol/EtOAc 4:1) in 67% yield as a yellow oil, starting from *N*-(4-methyl-1-oxopentan-2-yl)-3-phenylpropanamide (**1y**) (49 mg, 0.20 mmol) and ethynylbenzene (26 µL, 0.24 mmol); **^{1}H-NMR** (400 MHz, CDCl$_3$) δ 7.63 (d, J = 16.0, 1H, C(O)CH=CH), 7.54–7.45 (m, 2H, 2 × ArH), 7.39–7.31 (m, 3H, 3 × ArH), 7.23–7.17 (m, 2H, 2 × ArH), 7.16–7.08 (m, 3H, 3 × ArH), 6.71 (d, J = 16.0, 1H, C(O)CH=CH), 6.00

(d, J = 8.0, 1H, NH), 5.03–4.93 (m, 1H, NHCH), 2.98–2.85 (m, 2H, PhCH_2CH$_2$), 2.56–2.42 (m, 2H, PhCH$_2$CH_2), 1.58–1.42 (m, 2H, CH_2CH(CH$_3$)$_2$), 1.35–1.25 (m, 1H, CH(CH$_3$)$_2$), 0.92 (d, J = 6.5, 3H, CH_3), 0.81 (d, J = 6.5, 3H, CH_3); **^{13}C-NMR** (100 MHz, CDCl$_3$) δ 198.6, 171.9, 144.7, 140.8, 134.3, 131.1, 129.2, 128.7, 128.6, 128.5, 126.4, 123.1, 55.0, 42.0, 38.5, 31.7, 25.0, 23.5, 22.2; **IR** (film, cm^{-1}) 3293, 3028, 2956, 2869, 1643, 1609, 1537, 1496, 1450, 1070, 980, 755, 697; **LRMS** *m/z* (ESI$^+$) 372.2 ([M+Na]$^+$, 100%); **HRMS** (ESI$^+$) calc. for $C_{23}H_{27}O_2NNa$ [M+Na]$^+$: 372.1934, found: 372.1936.

(*E*)-*N*-(2-methyl-4-oxo-6-phenylhex-5-en-3-yl)-3-phenylpropanamide, 2z

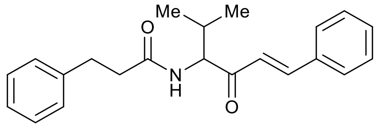

Following general procedure C, the product (24 mg) was isolated by FC (petrol/EtOAc 4:1) in 36% yield as a white amorphous solid, starting from *N*-(3-methyl-1-oxobutan-2-yl)-3-phenylpropanamide (**1z**) (47 mg, 0.20 mmol) and ethynylbenzene (26 µL, 0.24 mmol); **^{1}H-NMR** (400 MHz, CDCl$_3$) δ 7.61 (d, J = 16.0, 1H, C(O)CH=CH), 7.52–7.47 (m, 2H, 2 × ArH), 7.38–7.30 (m, 3H, 3 × ArH), 7.23–7.08 (m, 5H, 5 × ArH), 6.73 (d, J = 16.0, 1H, C(O)CH=CH), 6.12 (d, J = 8.5, 1H, C(O)NH), 4.92 (dd, J = 8.5, 4.5, 1H, C(O)NHCH), 2.98–2.85 (m, 2H, PhCH_2CH$_2$), 2.57–2.44 (m, 2H, PhCH$_2$CH_2), 2.10 (heptd, J = 7.0, 4.5, 1H, CH(CH$_3$)$_2$), 0.87 (d, J = 7.0, 3H, CH_3), 0.67 (d, J = 7.0, 3H, CH_3); **^{13}C-NMR** (100 MHz, CDCl$_3$) δ 198.2, 172.2, 144.5, 140.8, 134.3, 131.1, 129.2, 128.7, 128.7, 128.5, 126.4, 123.7, 61.0, 38.5, 31.8, 31.2, 19.9, 17.1; **IR** (film, cm^{-1}) 3305, 3027, 2962, 2930, 1644, 1608, 1531, 1496, 1450, 1072, 982, 755, 698; **LRMS** *m/z* (ESI$^+$) 358.2 ([M+Na]$^+$, 100%); **HRMS** (ESI$^+$) calc. for $C_{22}H_{25}O_2N^{23}Na$ [M+Na]$^+$: 358.1777, found: 358.1777; **m.p.** (°C): 86–88.

(*E*)-*N*-benzyl-*N*-(2-oxo-4-phenylbut-3-en-1-yl)benzamide, 2aa

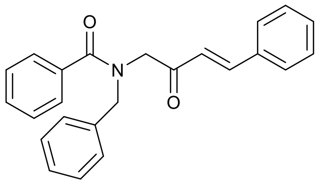

Following general procedure C, the product (60 mg) was isolated by FC (petrol/EtOAc 4:1) in 84% yield as a white amorphous solid, starting from *N*-benzyl-*N*-(2-oxoethyl)benzamide (**1aa**) (51 mg, 0.20 mmol) and ethynylbenzene (26 µL, 0.24 mmol); **^{1}H-NMR** (400 MHz, CDCl$_3$) [the

compound exists as two rotamers, in a ratio of 2:1] δ 7.65 (d, J = 16.0, 1H, C(O)CH=CH), 7.61–7.50 (m, 3H, 3 × ArH), 7.49–7.27 (m, 10H, 10 × ArH), 7.26–7.17 (m, 2H, 2 × ArH), 6.78 (d, J = 16.0, 1H, C(O)CH=CH), 6.55* (d, J = 16.0, 1H, C(O)CH=CH), 4.86* (s, 2H, NCH_2Ph), 4.65 (s, 2H, NCH_2Ph), 4.53 (s, 2H, NCH_2C(O)), 4.20* (s, 2H, NCH_2C(O)); **^{13}C-NMR** (100 MHz, $CDCl_3$) (* denotes minor rotamer) δ 194.3*, 193.9, 172.6, 144.2*, 143.7, 136.8*, 136.5, 135.6, 134.3, 131.2*, 130.9, 130.0, 129.1, 129.0, 128.9*, 128.6, 128.5, 127.9, 127.2, 127.1, 126.6*, 123.5, 122.3*, 56.2*, 53.9, 52.4, 49.2*; **IR** (film, cm^{-1}) 3060, 2922, 1634, 1612, 1576, 1495, 1450, 1429, 1088, 982, 732, 698; **LRMS** *m/z* (ESI$^+$) 378.2 ([M+Na]$^+$, 100%); **HRMS** (ESI$^+$) calc. for $C_{24}H_{21}O_2NNa$ [M+Na]$^+$: 378.1464, found: 378.1465; **m.p.** (°C): 117–119.

(*E*)-*N*-benzyl-*N*-(2-oxo-4-phenylbut-3-en-1-yl)acetamide, 2ab

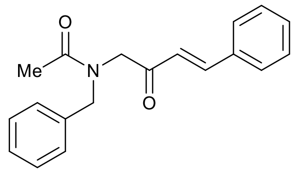

Following general procedure C, the product (27 mg) was isolated by FC (petrol/EtOAc 4:1) in 46% yield as a yellow oil, starting from *N*-benzyl-*N*-(2-oxoethyl)acetamide (**1ab**) (38 mg, 0.20 mmol) and ethynylbenzene (26 µL, 0.24 mmol); **^{1}H-NMR** (400 MHz, $CDCl_3$) [the compound exists as two rotamers, in a ratio of 3:1] δ 7.58–7.49 (m, 1H, C(O)CH=CH), 7.47–7.40 (m, 2H, 2 × ArH), 7.37–7.16 (m, 6H, 6 × ArH), 7.15–7.10 (m, 2H, 2 × ArH), 6.68–6.58 (m, 1H, C(O)CH=CH), 4.59* (s, 2H, NCH_2Ph), 4.57 (s, 2H, NCH_2Ph), 4.35 (s, 2H, NCH_2C(O)), 4.17* (s, 2H, NCH_2C(O)), 2.18 (s, 3H, CH_3), 2.00* (s, 3H, CH_3); **^{13}C-NMR** (100 MHz, $CDCl_3$) (* denotes minor rotamer) δ 194.5, 194.0*, 171.5, 171.4*, 144.6*, 143.7, 137.1*, 136.4, 134.4, 133.9*, 131.3*, 130.9, 129.2*, 129.1, 129.1, 128.8*, 128.7*, 128.6*, 128.5, 128.0, 127.7*, 126.9, 123.5, 122.1*, 55.9*, 53.1, 53.0, 49.9*, 21.6*, 21.4; **IR** (film, cm^{-1}) 3028, 2923, 1677, 1645, 1613, 1495, 1449, 1240, 1191, 1095, 985, 749, 692; **LRMS** *m/z* (ESI$^+$) 316.2 ([M+Na]$^+$, 100%); **HRMS** (ESI$^+$) calc. for $C_{19}H_{20}O_2N$ [M+H]$^+$: 294.1499, found: 294.1492.

Benzyl (*E*)-benzyl(2-oxo-4-phenylbut-3-en-1-yl)carbamate, 2ac

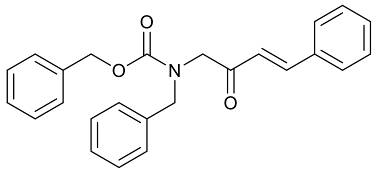

Following general procedure C, the product (20 mg) was isolated by FC (petrol/EtOAc 1:4) in 26% yield as a yellow oil, starting from benzyl benzyl(2-oxoethyl)carbamate (**1ac**) (57 mg, 0.20 mmol) and ethynylbenzene (26 µL, 0.24 mmol); **^{1}H-NMR** (400 MHz, $CDCl_3$) [the compound exists as two rotamers, in a ratio of 1:1] δ 7.53 (d, J = 16.0, 1H, C(O)CH=C*H*), 7.49–7.34 (m, 2H, 2 × Ar*H*), 7.33–7.16 (m, 11H, 11 × Ar*H*), 7.15–7.08 (m, 2H, 2 × Ar*H*), 6.63 (d, J = 16.0, 1H, C(O)C*H*=CH), 6.57* (d, J = 16.0, 1H, C(O)C*H*=CH), 5.17 (s, 2H, OCH_2Ph), 5.11* (s, 2H, OCH_2Ph), 4.56 (s, 2H, NCH_2Ph), 4.54* (s, 2H, NCH_2Ph), 4.22 (s, 2H, NCH_2C(O)), 4.11* (s, 2H, NCH_2C(O)); **^{13}C-NMR** (100 MHz, $CDCl_3$) δ 194.9, 194.8*, 156.8, 156.7*, 143.8, 137.1, 136.6, 136.6*, 134.4, 134.2*, 131.0, 130.9*, 129.1, 128.8, 128.6, 128.6*, 128.6, 128.5*, 128.2, 128.1*, 128.0, 128.0*, 127.9, 127.8*, 127.7, 123.1, 122.5*, 67.9, 67.8*, 54.4, 54.3*, 51.9, 51.4*; **IR** (film, cm^{-1}) 3031, 2938, 1700, 1613, 1495, 1451, 1230, 1116, 948, 749, 697; **LRMS** *m/z* (ESI^+) 408.2 ($[M+Na]^+$, 100%); **HRMS** (ESI^+) calc. for $C_{25}H_{23}O_3NNa$ $[M+Na]^+$: 408.1570, found: 408.1572.

(*E*)-*N*-benzyl-*N*-(2-oxo-4-phenylbut-3-en-1-yl)cyclohexanecarboxamide, 2ad

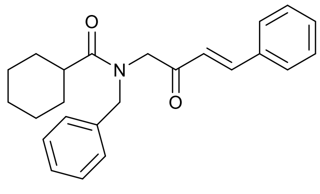

Following general procedure C, the product (20 mg) was isolated by FC (petrol/EtOAc 4:1) in 28% yield as a yellow oil, starting from N-benzyl-N-(2-oxoethyl)cyclohexanecarboxamide (**1ad**) (52 mg, 0.20 mmol),ethynylbenzene (26 µL, 0.24 mmol) and $[Rh(dppe)(C_6H_5F)][BAr^F{}_4]$ (29 mg, 0.02 mmol, 10 mol%); **^{1}H-NMR** (400 MHz, $CDCl_3$) [the compound exists as two rotamers, in a ratio of 3:1] δ 7.60–7.49 (m, 1H, C(O)CH=C*H*), 7.48–7.40 (m, 2H, 2 × Ar*H*), 7.38–7.17 (m, 6H, 6 × Ar*H*), 7.16–7.11 (m, 2H, 2 × Ar*H*), 6.69–6.59 (m, 1H, C(O)C*H*=CH), 4.62–4.57 (m, 2H, NCH_2Ph), 4.28 (s, 2H, NCH_2C(O)), 4.18* (s, 2H, NCH_2C(O)), 2.57 (tt, J = 11.5, 3.0, 1H, C(O)C*H*(CH_2)$_5$), 2.14* (tt, J = 11.5, 3.0, 1H, C(O)C*H*(CH_2)$_5$), 1.85–1.66 (m, 4H, 2 × CH_2), 1.65–1.46 (m, 3H, 1.5 × CH_2), 1.29–1.06 (m, 3H, 1.5 × CH_2); **^{13}C-NMR** (100 MHz, $CDCl_3$) δ 194.9, 177.2, 144.5*, 143.6, 136.9, 134.5, 130.8, 129.2*, 129.1, 129.1, 128.8*, 128.7*, 128.5, 128.4*, 127.9, 127.6*, 126.9, 123.5, 122.0*, 55.1*, 53.3, 52.0, 49.8*, 41.2*, 40.7, 29.7,

29.7*, 25.9*, 25.9; **IR** (film, cm^{-1}) 2927, 2853, 1639, 1613, 1494, 1449, 1202, 1176, 1090, 749, 693; **LRMS** *m/z* (ESI^+) 384.2 ($[M+Na]^+$, 100%); **HRMS** (ESI^+) calc. for $C_{24}H_{27}O_2NNa$ $[M+Na]^+$: 384.1934, found: 384.1930.

(*E*)-*N*-(3-oxo-1,5-diphenylpent-4-en-2-yl)cyclohexanecarboxamide, 2ae

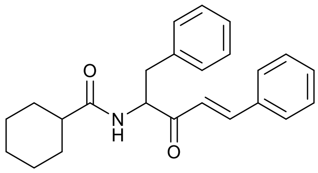

Following general procedure C, the product (32 mg) was isolated by FC (petrol/EtOAc 4:1) in 44% yield as a pale yellow solid, starting from *N*-(1-oxo-3-phenylpropan-2-yl)cyclohexanecarboxamide (**1ae**) (52 mg, 0.20 mmol) and ethynylbenzene (26 µL, 0.24 mmol); **^{1}H-NMR** (400 MHz, $CDCl_3$) δ 7.57 (d, *J* = 16.0, 1H, C(O)CH=C*H*), 7.48–7.37 (m, 2H, 2 × Ar*H*), 7.39–7.26 (m, 3H, 3 × Ar*H*), 7.24–7.09 (m, 3H, 3 × Ar*H*), 7.10–6.99 (m, 2H, 2 × Ar*H*), 6.65 (d, *J* = 16.0, 1H, C(O)C*H*=CH), 6.23 (d, *J* = 7.5, 1H, N*H*), 5.19–5.08 (m, 1H, NHC*H*), 3.10 (app qd, *J* = 14.0, 6.0, 2H, CH_2Ph), 2.05 (tt, *J* = 12.0, 3.5, 1H, C*H*(CH_2)$_5$), 1.83–1.49 (m, 5H, 2.5 × CH_2), 1.44–1.25 (m, 2H, CH_2), 1.27–1.05 (m, 3H, 1.5 × CH_2); **^{13}C-NMR** (100 MHz, $CDCl_3$) δ 197.1, 175.5, 144.7, 136.0, 134.1, 131.0, 129.6, 129.0, 128.6, 128.4, 127.0, 123.0, 57.5, 45.4, 38.1, 29.7, 29.4, 25.7, 25.6; **IR** (film, cm^{-1}) 3292, 3028, 2927, 2853, 1642, 1609, 1576, 1495. 1449, 1332, 1070, 981, 759, 729, 698; **LRMS** *m/z* (ESI^+) 384.2 ($[M+Na]^+$, 100%); **HRMS** (ESI^+) calc. for $C_{24}H_{27}O_2NNa$ $[M+Na]^+$: 384.1934, found: 384.1926; **m.p.** (°C): 111–113.

3.4 Oxazole synthesis

(*E*)-4-benzyl-2-phenethyl-5-styryloxazole, 3

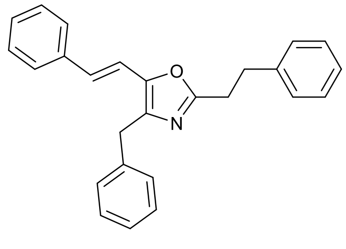

Compound **3** was synthesised by modifying a literature procedure.[6] To a solution of (*E*)-*N*-(3-oxo-1,5-diphenylpent-4-en-2-yl)-3-phenylpropanamide (**2w**) (100 mg, 0.26 mmol) in dry toluene (2 mL) was added $POCl_3$ (0.5 mL, 0.52 mmol, 2 equiv.) at room temperature. The solution was stirred at 95 °C for 12 hours. After cooling to room temperature, the mixture was poured into water. By addition of an aqueous solution of NaOH (4 M) at 0 °C, the pH was adjusted to 7. The mixture was extracted with EtOAc. The combined organic layers were dried over $MgSO_4$, filtered and concentrated in *vacuo*. The crude product was subjected to FC (petrol/EtOAc 5:1) to afford the the title compound as a yellow amorphous solid (90 mg, 92%); **^{1}H-NMR** (400 MHz, $CDCl_3$) δ 7.50–7.45 (m, 2H, 2 × Ar*H*), 7.44–7.24 (m, 13H, 13 × Ar*H*), 7.00 (d, *J* = 16.0, 1H, PhC*H*=CH), 6.79 (d, *J* = 16.0, 1H, PhCH=C*H*), 4.02 (s, 2H, PhCH_2), 3.25–3.08 (m, 4H, PhCH_2CH_2); **^{13}C-NMR** (100 MHz, $CDCl_3$) δ 162.9, 145.6, 140.5, 138.8, 136.9, 136.6, 128.8, 128.8, 128.7, 128.6, 128.5, 127.9, 127.8, 126.6, 126.4, 112.7, 33.4, 32.6, 30.4.; **IR** (film, cm^{-1}) 3026, 2360, 1601, 1572, 1494, 1453, 1428, 1157, 1075, 1029, 953, 750, 717, 694; **LRMS** *m/z* (ESI$^+$) 366.2 ([M+H]$^+$, 100%); **HRMS** (ESI$^+$) calc. for $C_{26}H_{24}ON$ [M+H]$^+$: 366.1852, found: 366.1843; **m.p.** (°C): 73–75.

4. References

1. Stahl, E. *Thin Layer Chromatography*; Springer-Verlag, Berlin, 1969.
2. M. Fernandez, M. Castaing, M. C. Willis, *Chem. Sci.* **2017**, *8*, 536-540.
3. T. J. Coxon, M. Fernandez, J. Barwick-Silk, A. I. McKay, L. E. Britton, A. S. Weller, M. C. Willis, *J. Am. Chem. Soc.* **2017**, *139*, 10142-10149.
4. D. C. Beshore, C. J. Dinsmore, *Org. Lett.* **2002**, *4*, 1201-1204.
5. J. F. Hooper, S. Seo, F. R. Truscott, J. D. Neuhaus, M. C. Willis, *J. Am. Chem. Soc.* **2016**, *138*, 1630-1634.
6. R. F. Meyer, A. D. Essenburg, R. D. Smith, H. R. Kaplan, *J. Med. Chem.* **1982**, *25*, 996-999.

5. NMR spectra

N-benzyl-*N*-(2-hydroxyethyl)-3-phenylpropanamide, OH-1a

^{1}H-NMR (400 MHz, $CDCl_3$)

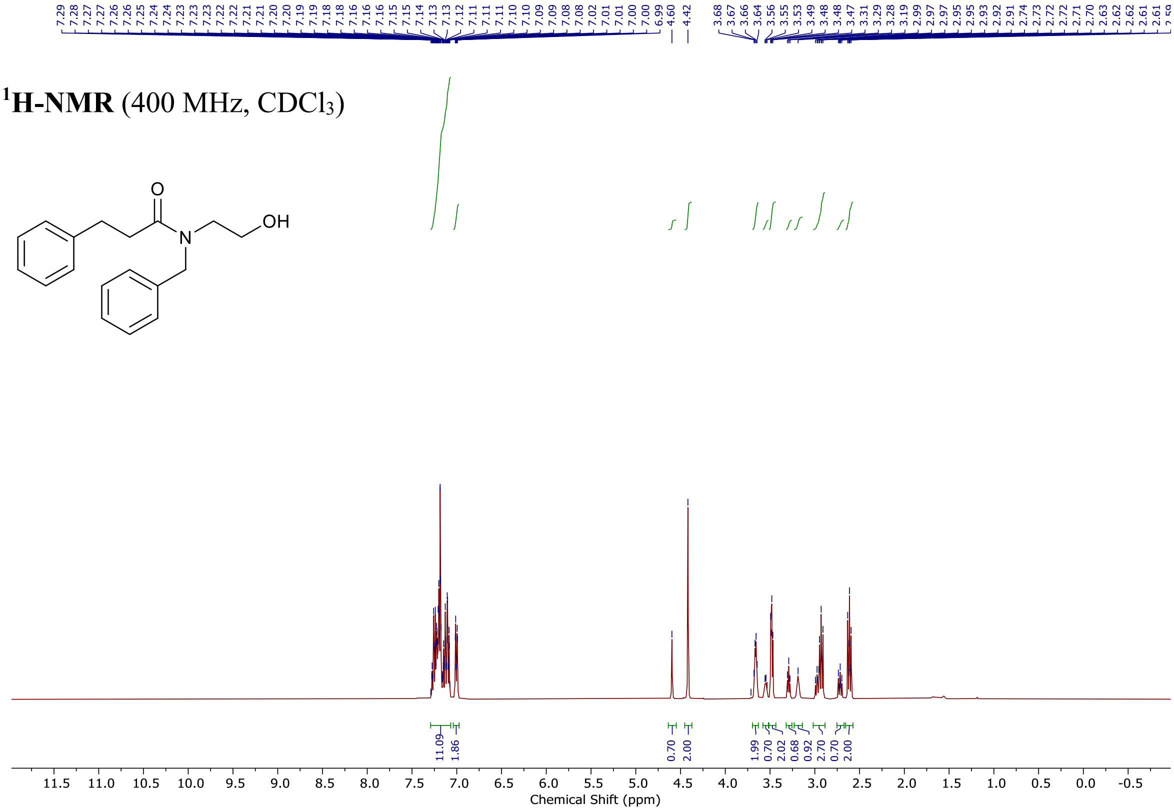

^{13}C-NMR (100 MHz, $CDCl_3$)

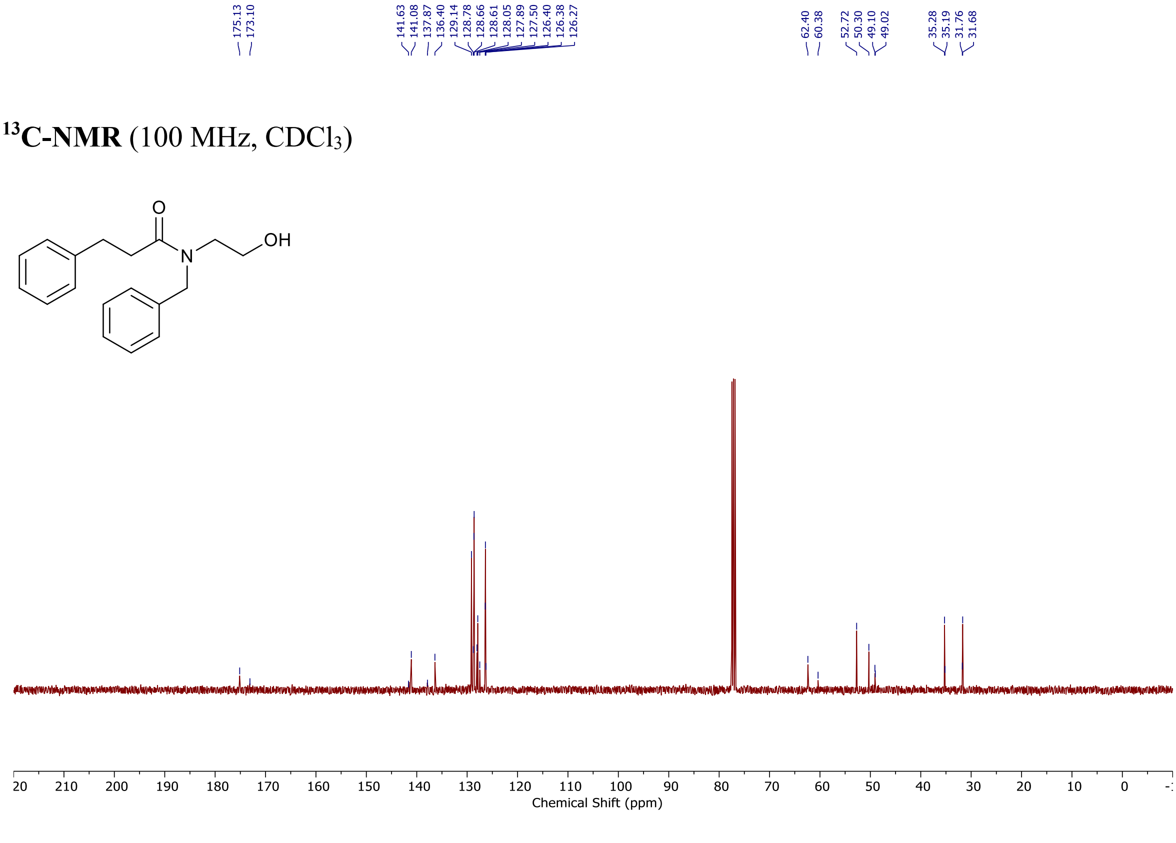

N-(2-hydroxyethyl)-*N*-methyl-3-phenylpropanamide, OH-1s

^{1}H-NMR (400 MHz, $CDCl_3$)

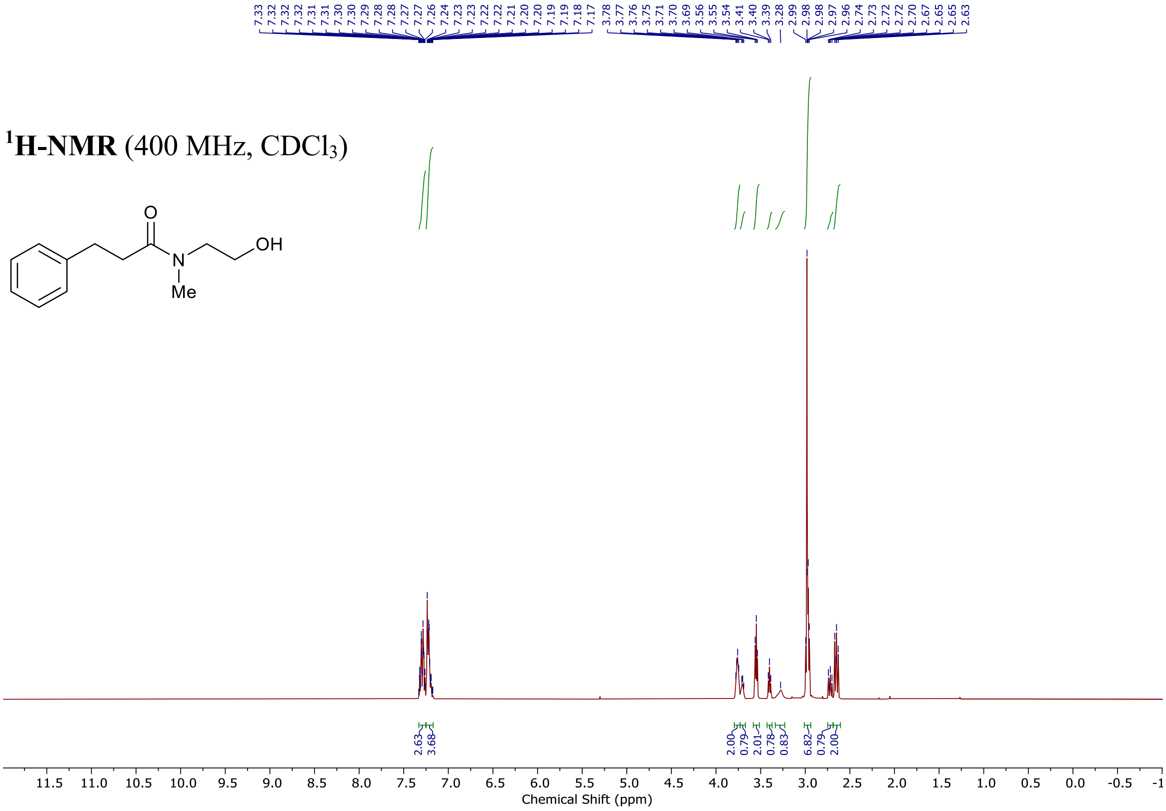

^{13}C-NMR (100 MHz, $CDCl_3$)

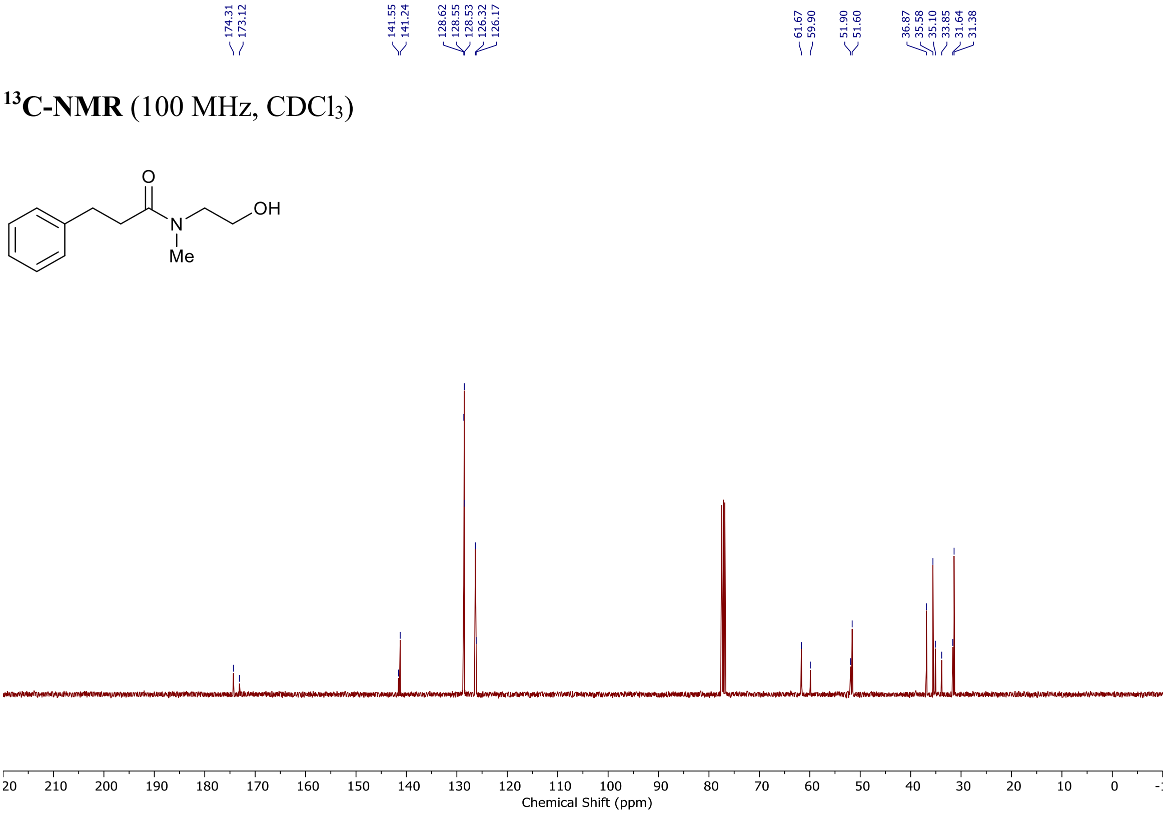

_N_-(2-hydroxyethyl)-_N_,3-diphenylpropanamide, OH-1t

^{1}H-NMR (400 MHz, $CDCl_3$)

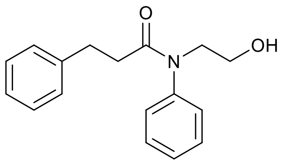

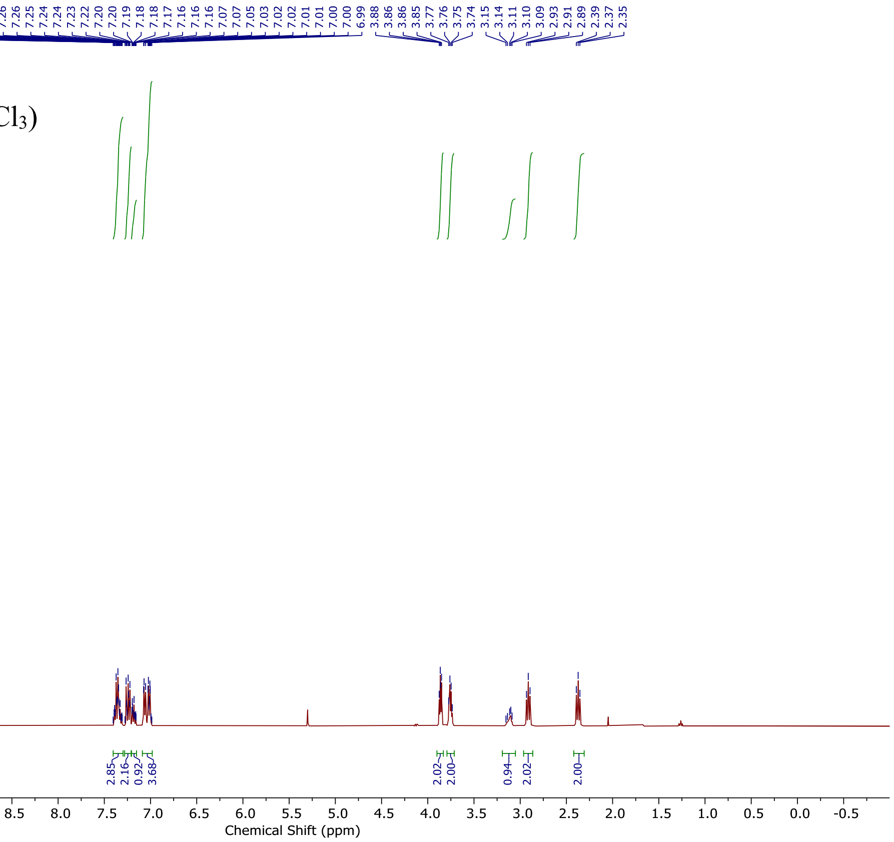

^{13}C-NMR (100 MHz, $CDCl_3$)

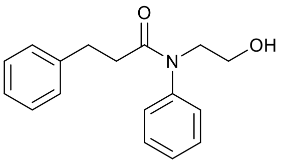

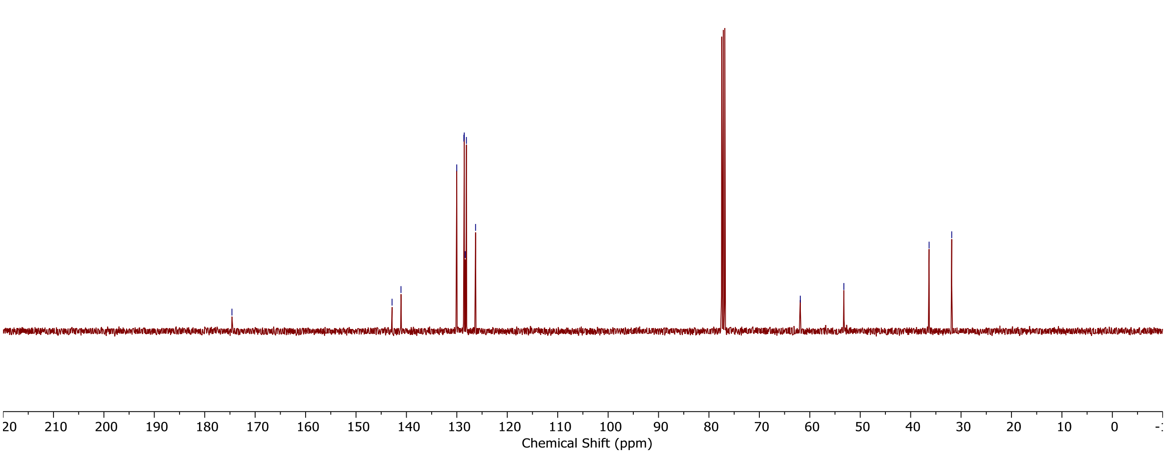

1-(2-(hydroxymethyl)pyrrolidin-1-yl)-3-phenylpropan-1-one, OH-1u

^{1}H-NMR (400 MHz, $CDCl_3$)

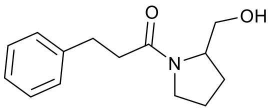

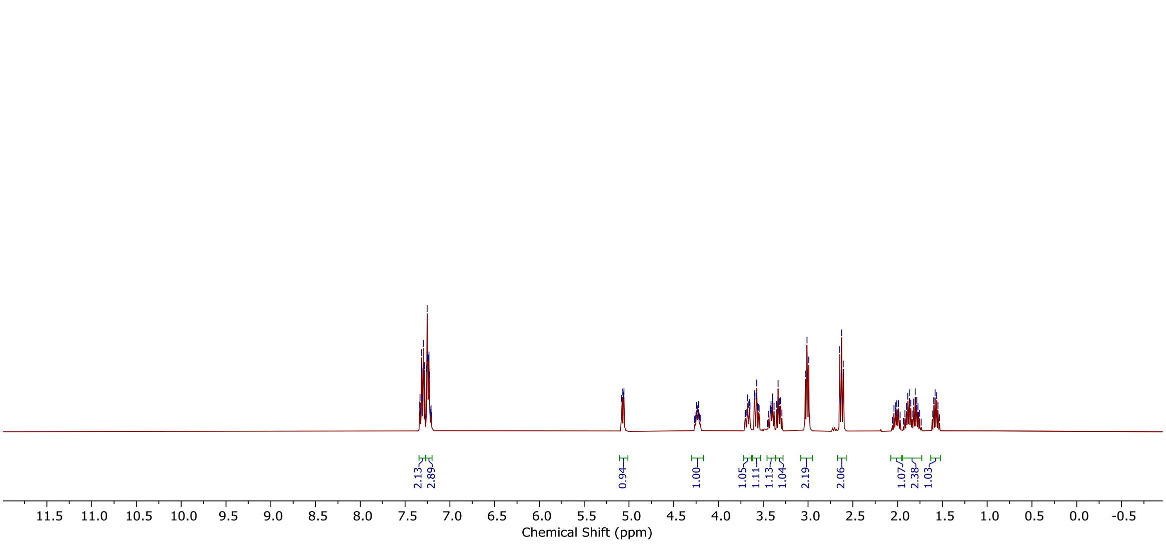

^{13}C-NMR (100 MHz, $CDCl_3$)

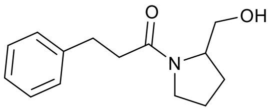

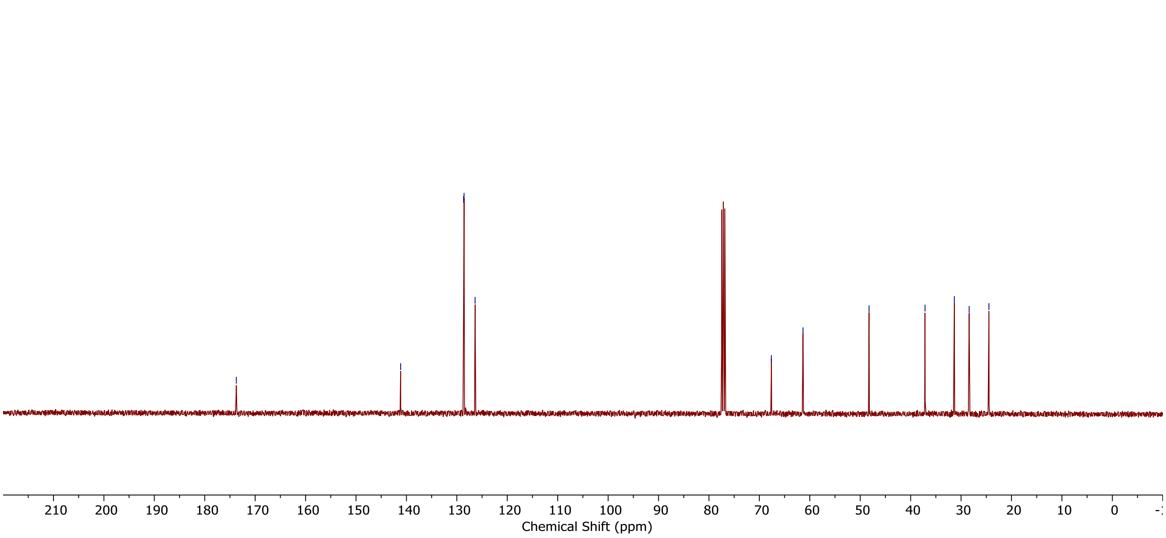

N-(2,2-dimethoxyethyl)-3-phenylpropanamide, AC-1v

^{1}H-NMR (400 MHz, $CDCl_3$)

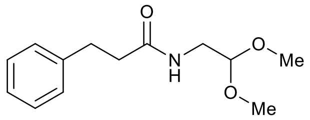

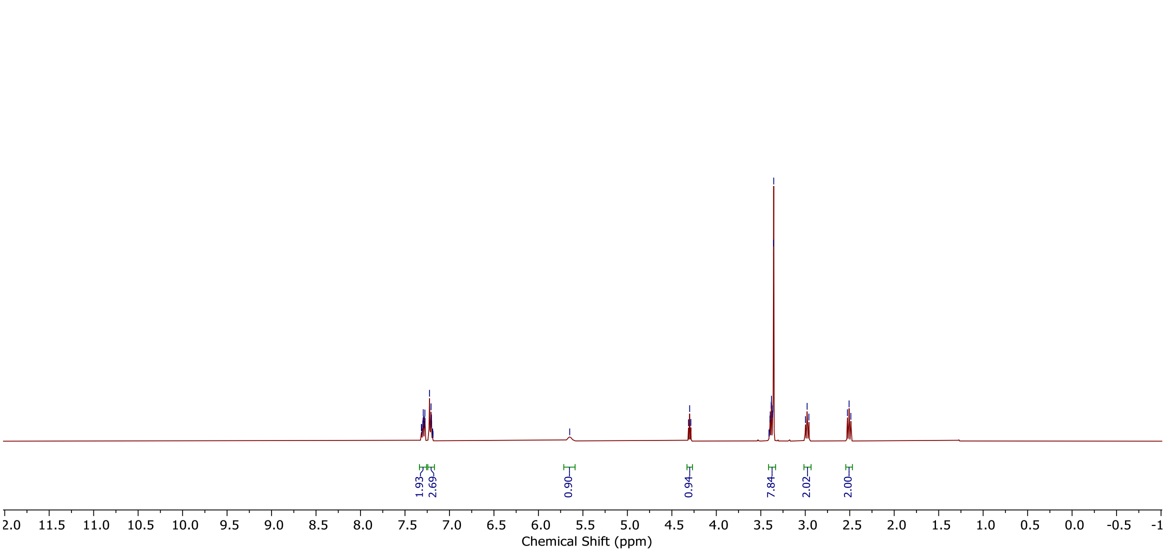

^{13}C-NMR (100 MHz, $CDCl_3$)

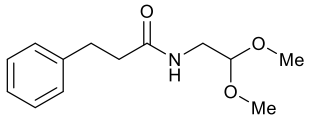

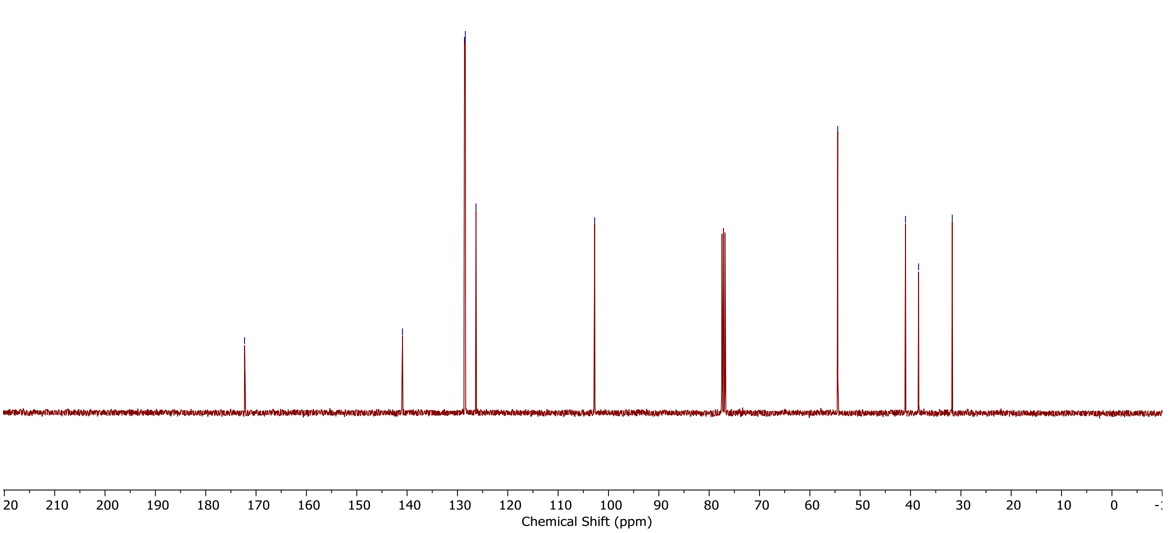

N-(1-hydroxy-3-phenylpropan-2-yl)-3-phenylpropanamide, OH-1w

^{1}H-NMR (400 MHz, $CDCl_3$)

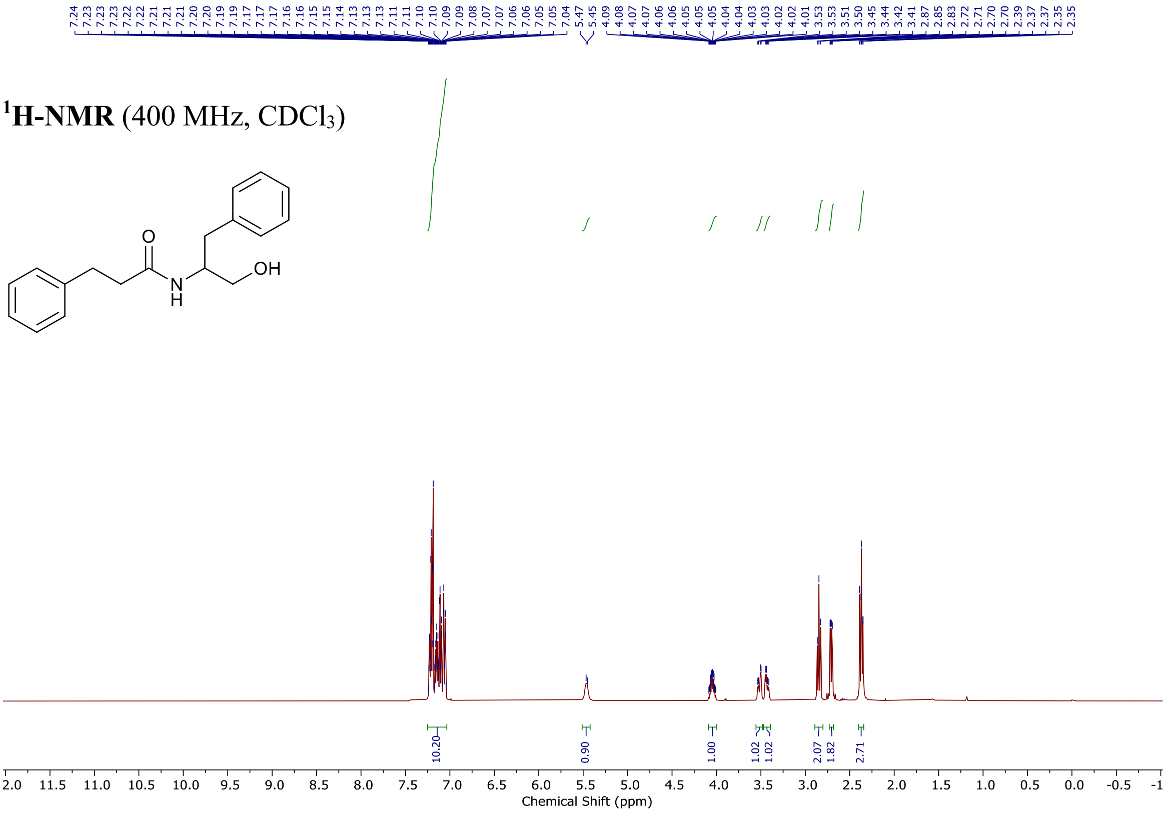

^{13}C-NMR (100 MHz, $CDCl_3$)

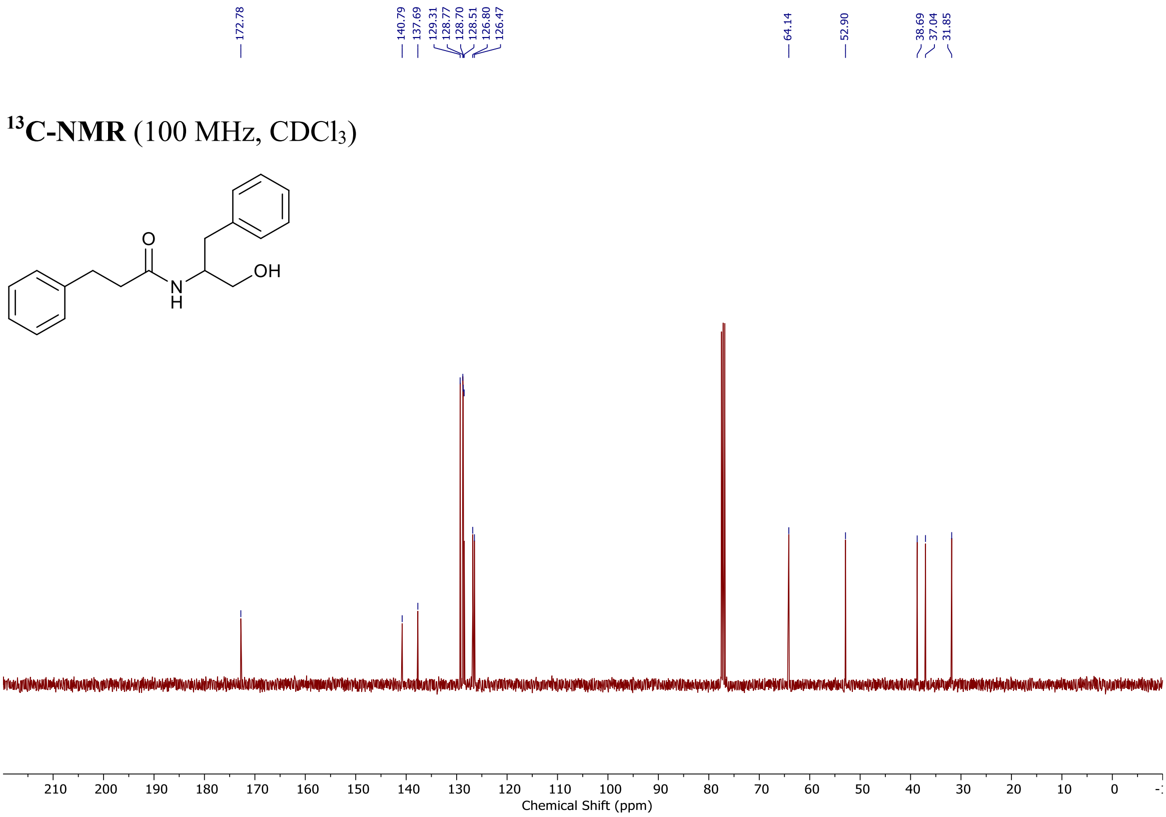

N-(1-hydroxypropan-2-yl)-3-phenylpropanamide, OH-1x

^{1}H-NMR (400 MHz, $CDCl_3$)

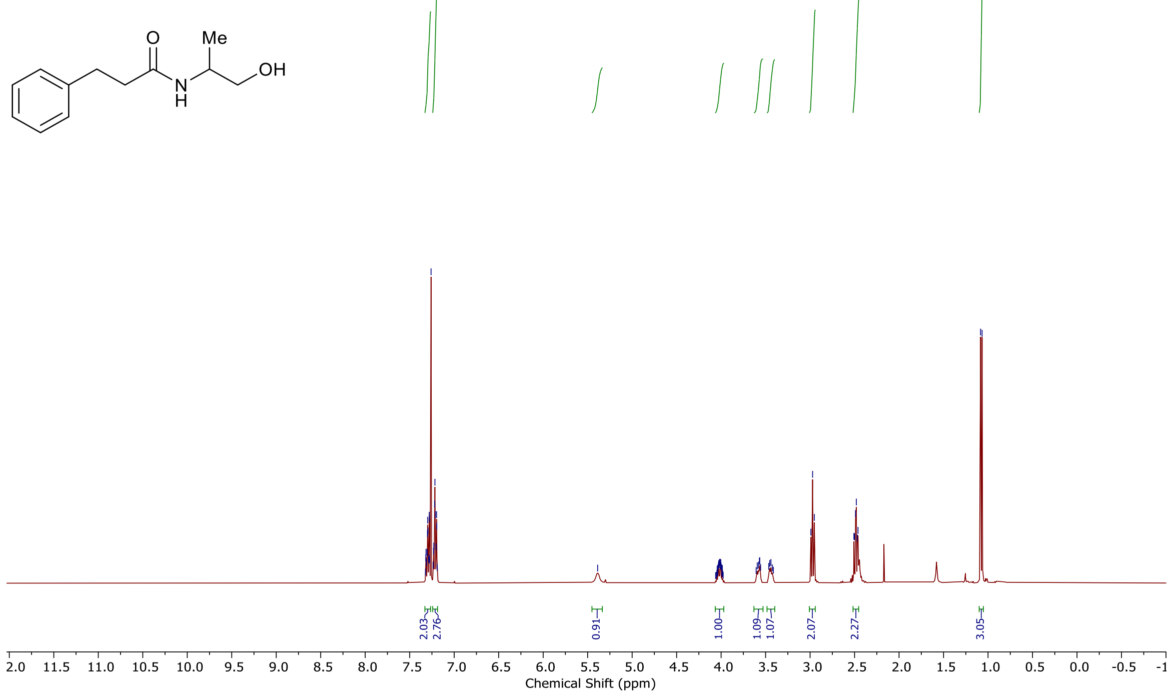

^{13}C-NMR (100 MHz, $CDCl_3$)

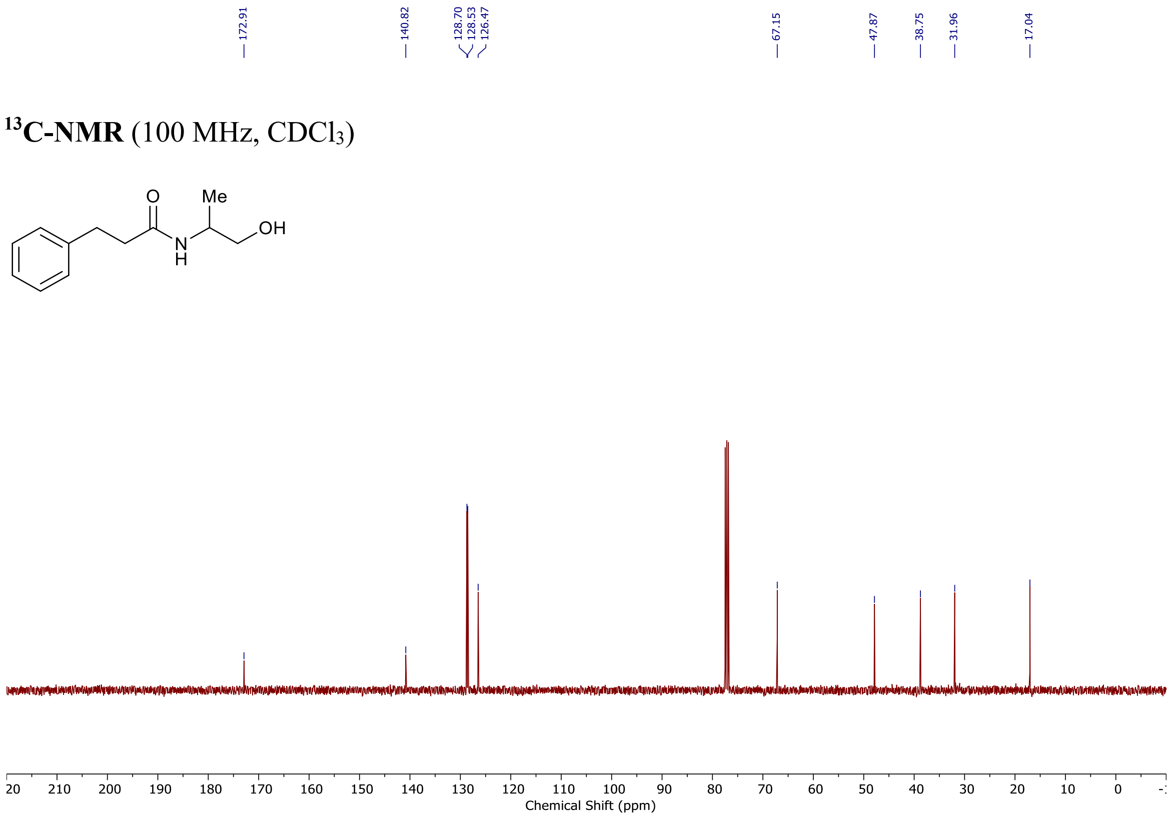

N-(1-hydroxy-4-methylpentan-2-yl)-3-phenylpropanamide, OH-1y

^{1}H-NMR (400 MHz, $CDCl_3$)

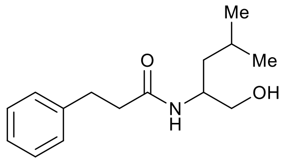

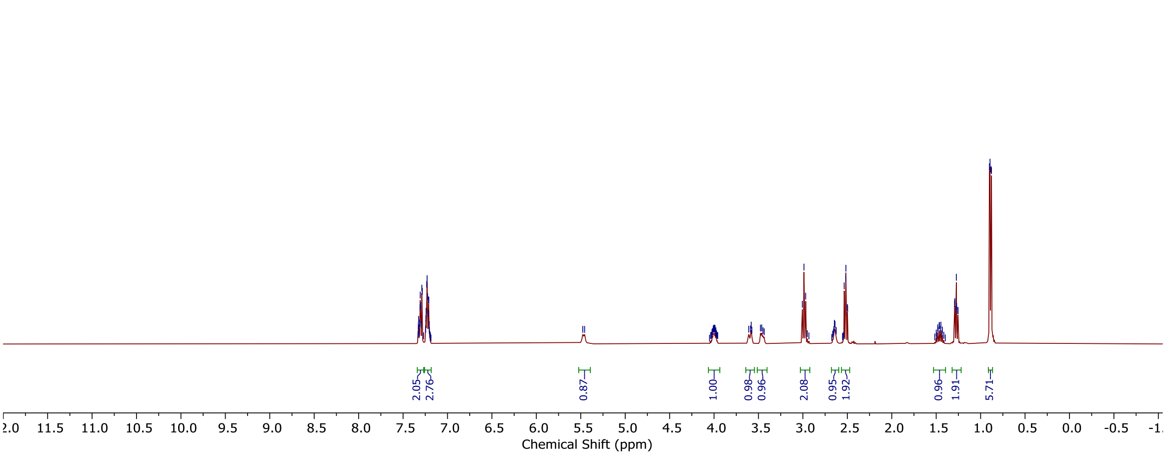

^{13}C-NMR (100 MHz, $CDCl_3$)

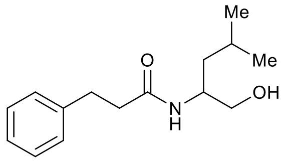

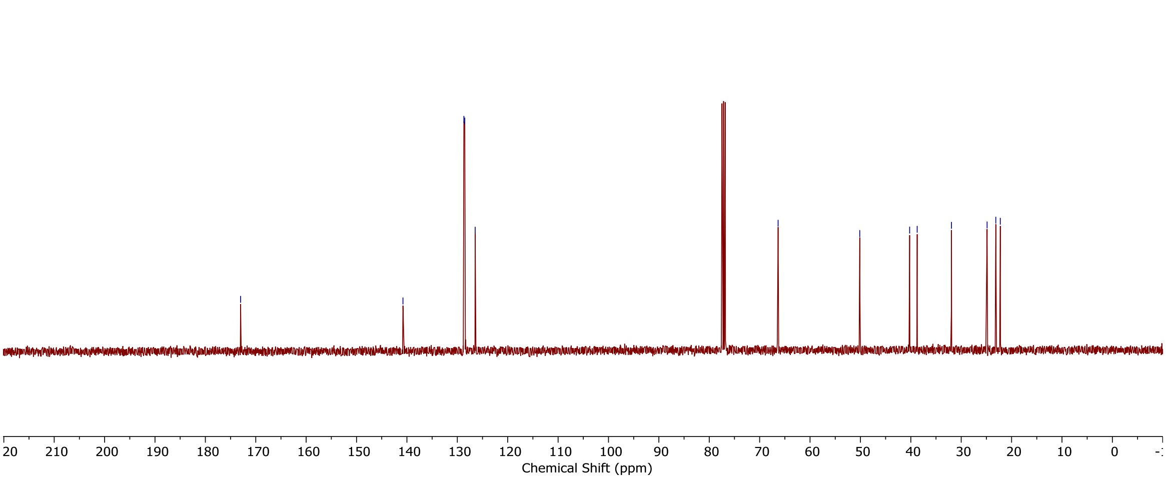

N-(1-hydroxy-3-methylbutan-2-yl)-3-phenylpropanamide, OH-1z

^{1}H-NMR (400 MHz, $CDCl_3$)

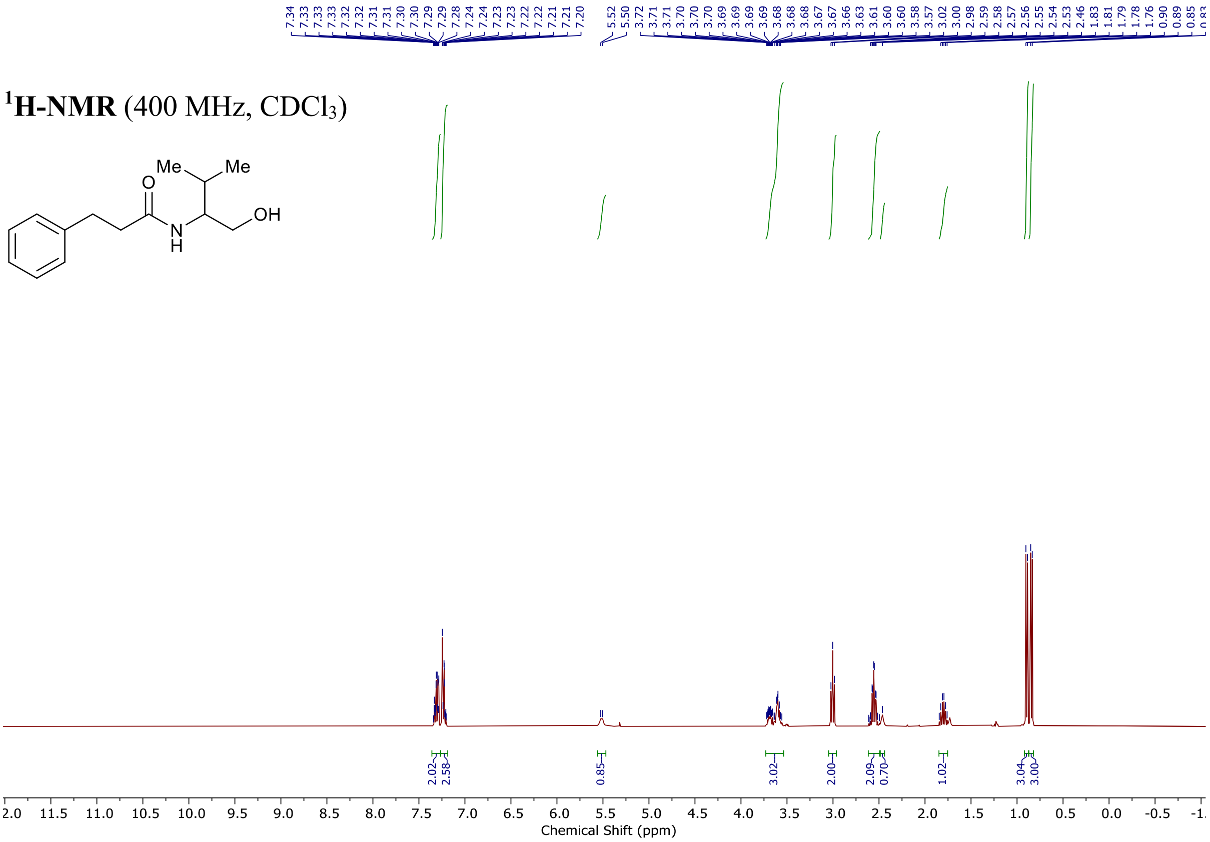

^{13}C-NMR (100 MHz, $CDCl_3$)

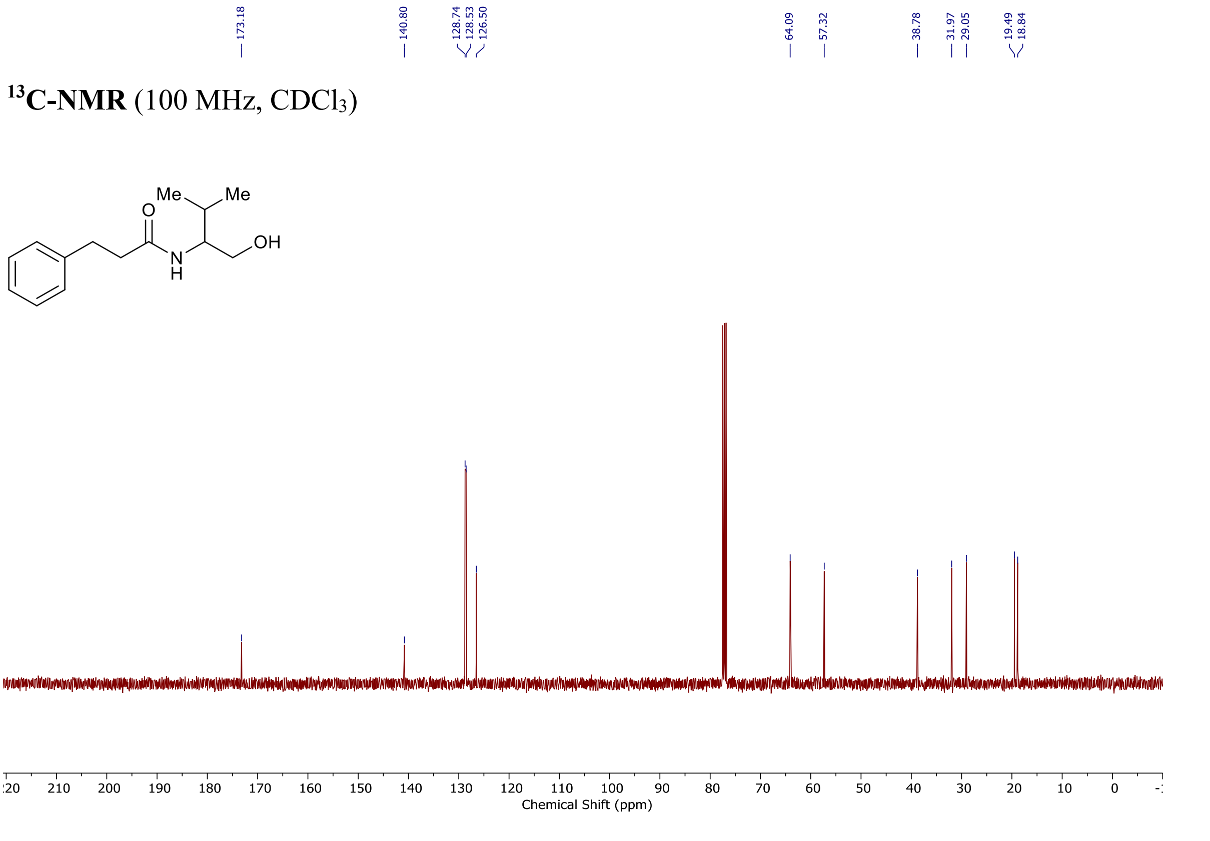

N-benzyl-*N*-(2-hydroxyethyl)acetamide, OH-1ab

^{1}H-NMR (400 MHz, $CDCl_3$)

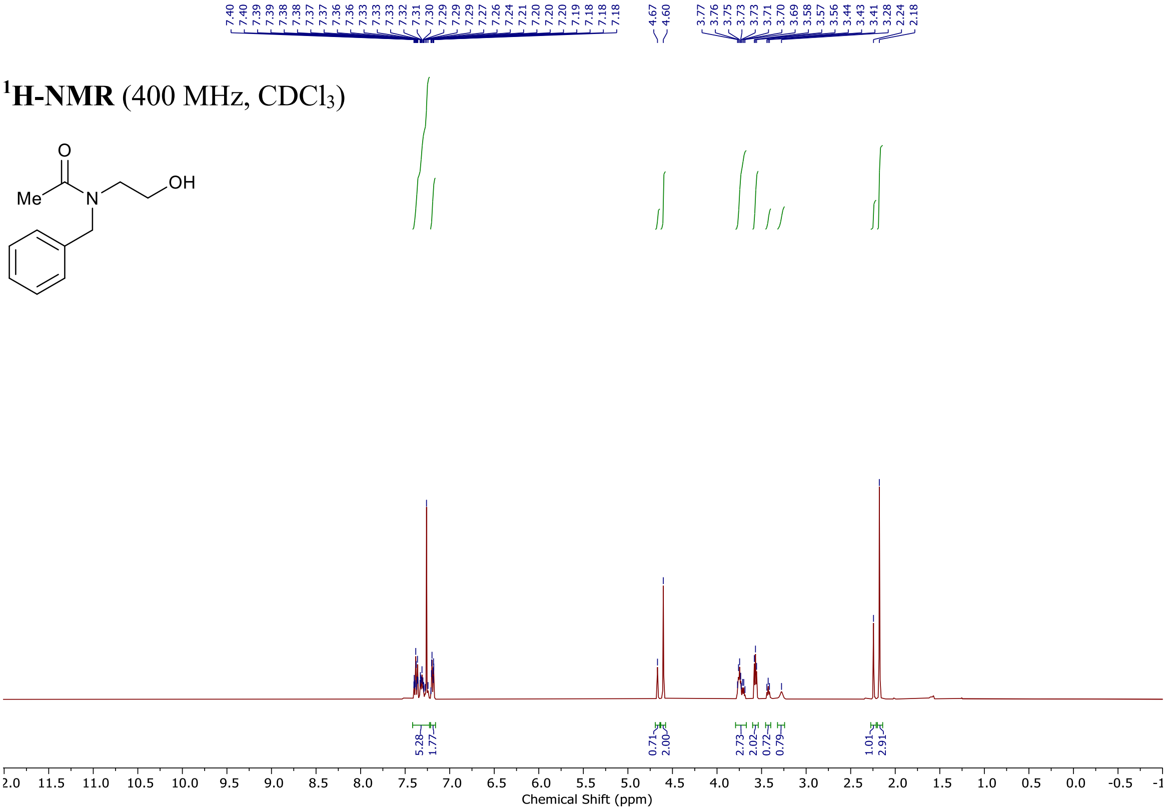

^{13}C-NMR (100 MHz, $CDCl_3$)

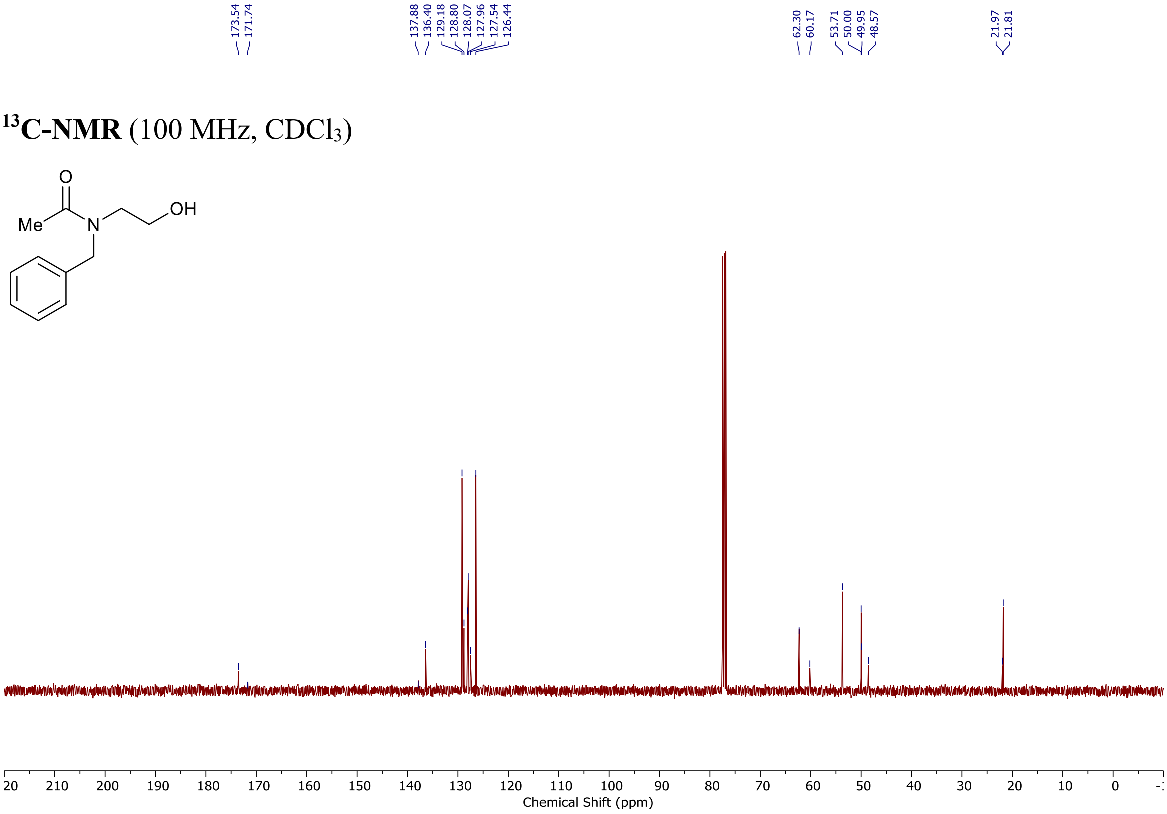

Benzyl benzyl(2-hydroxyethyl)carbamate, OH-1ac

^{1}H-NMR (400 MHz, $CDCl_3$)

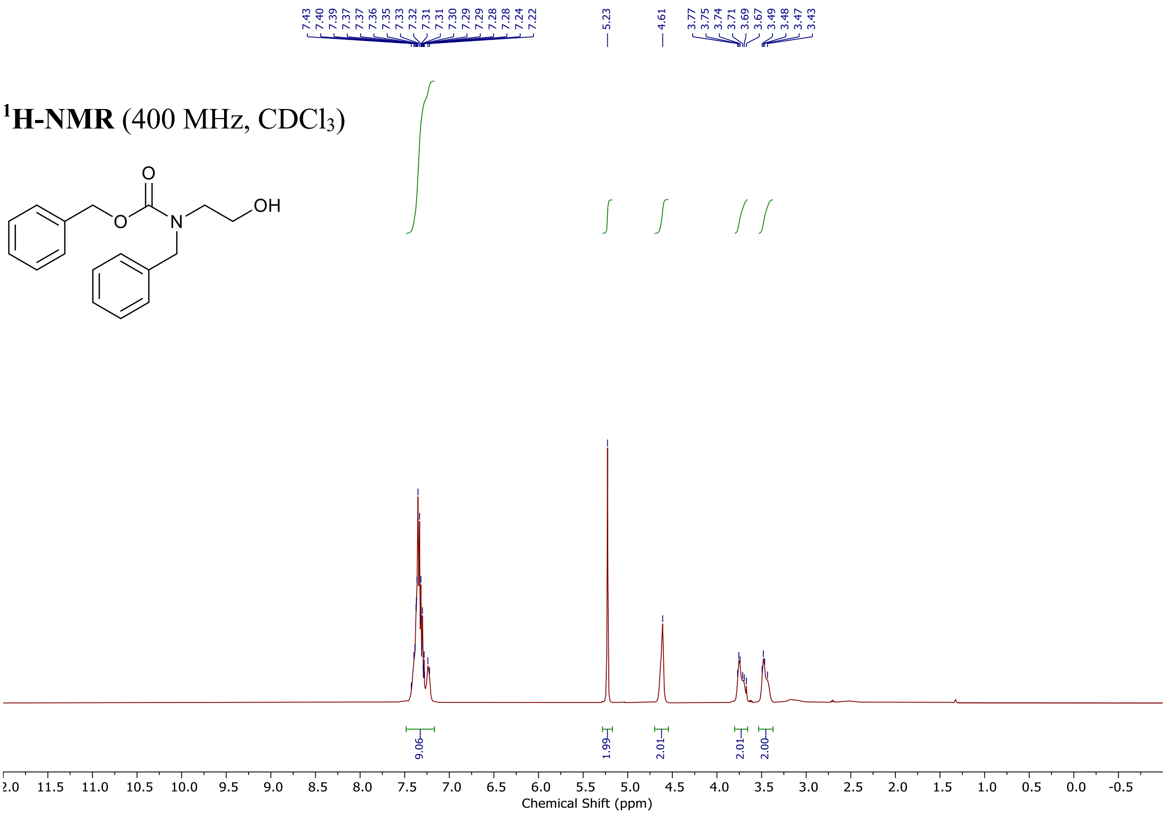

^{13}C-NMR (100 MHz, $CDCl_3$)

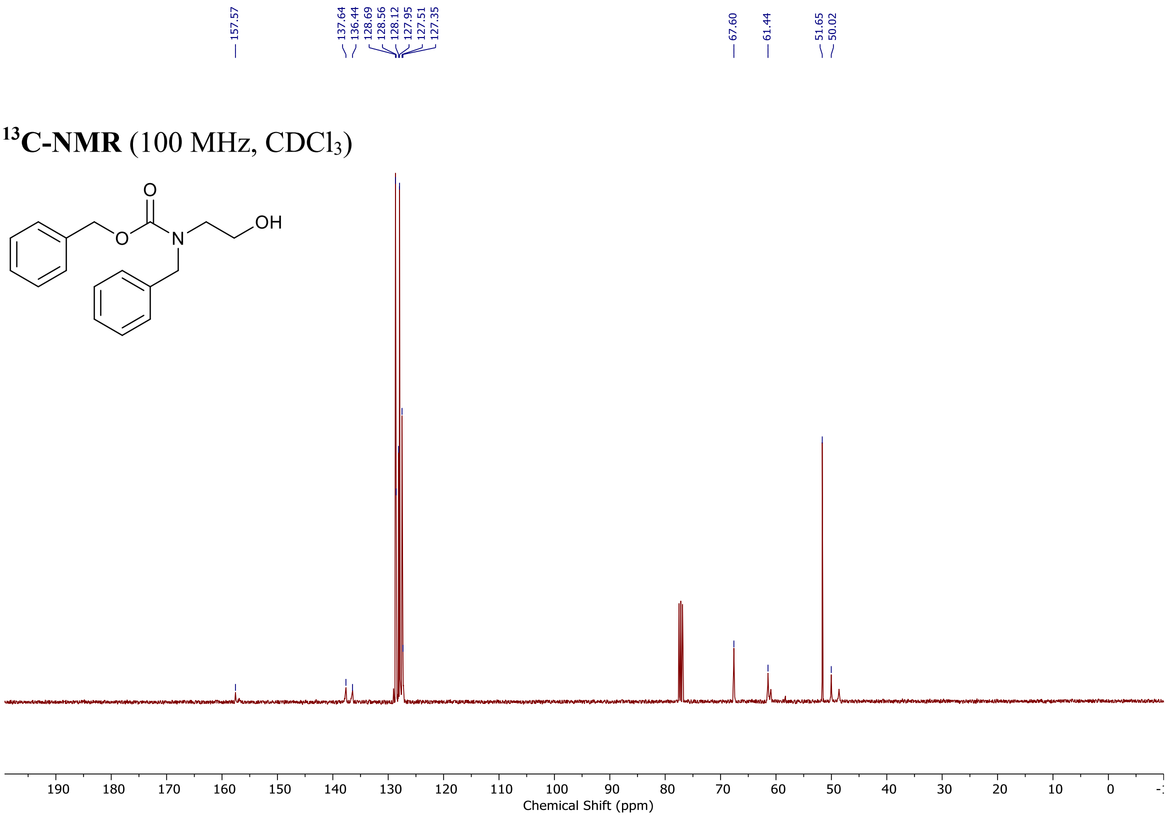

N-benzyl-*N*-(2-hydroxyethyl)cyclohexanecarboxamide, OH-1ad

^{1}H-NMR (400 MHz, $CDCl_3$)

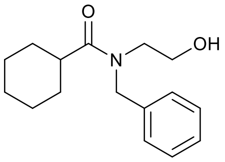

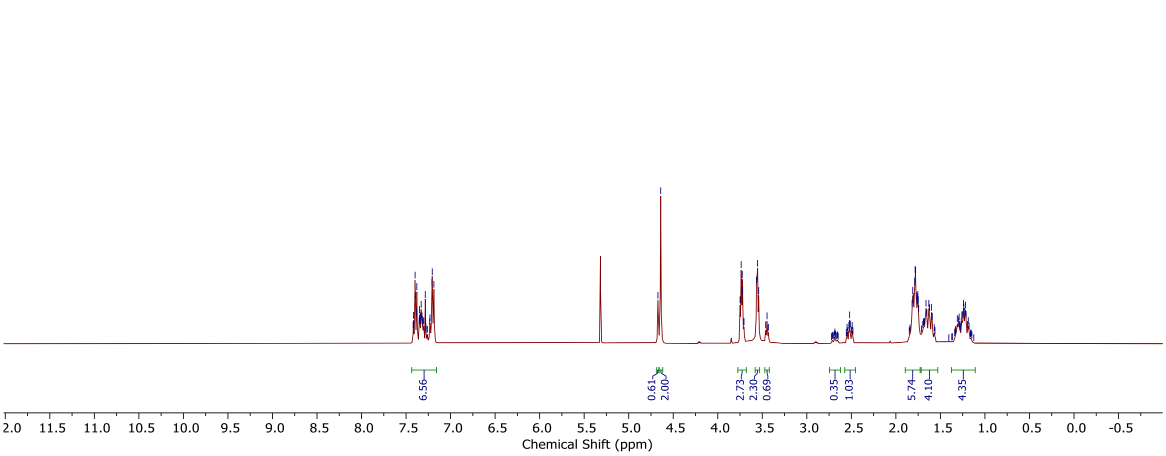

^{13}C-NMR (100 MHz, $CDCl_3$)

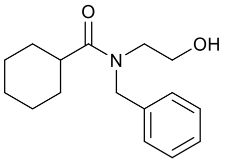

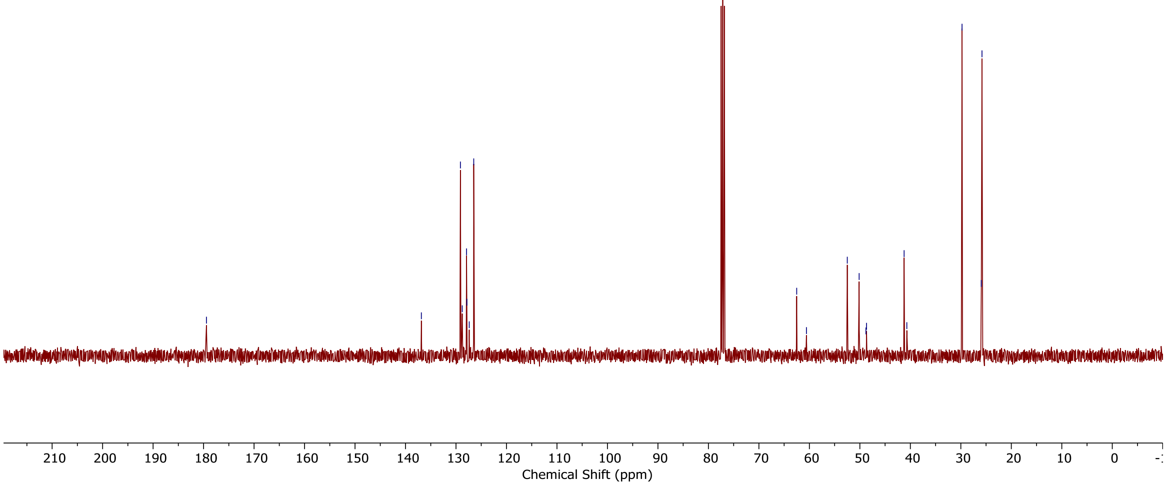

N-(1-hydroxy-3-phenylpropan-2-yl)cyclohexanecarboxamide, OH-1ae

^{1}H-NMR (400 MHz, $CDCl_3$)

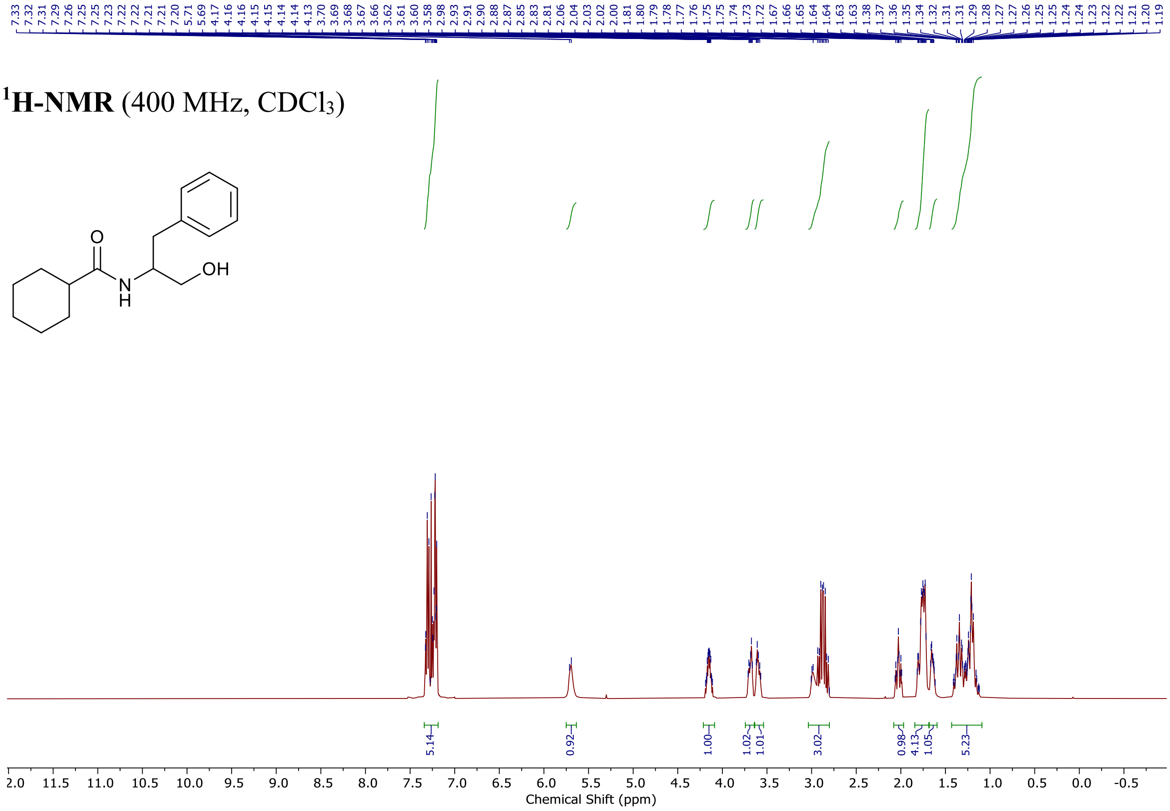

^{13}C-NMR (100 MHz, $CDCl_3$)

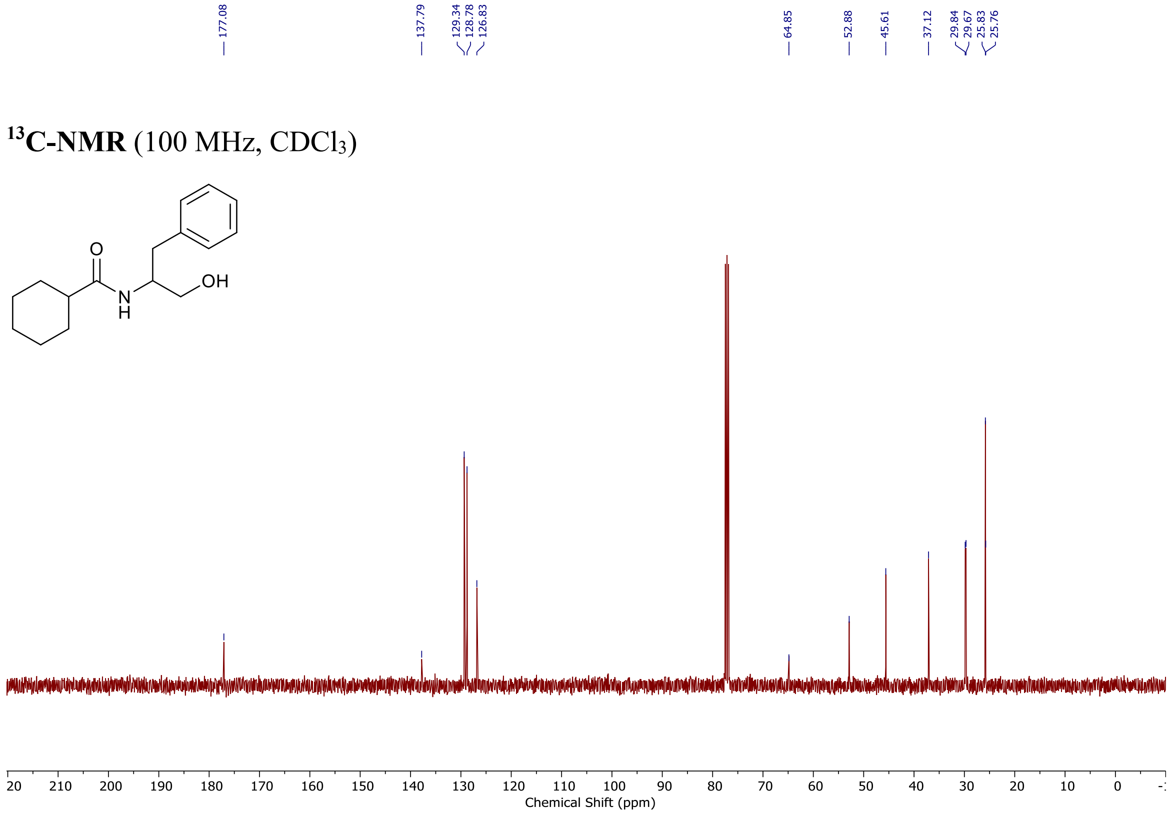

N-benzyl-*N*-(2-oxoethyl)-3-phenylpropanamide, 1a

[1]**H-NMR** (400 MHz, $CDCl_3$)

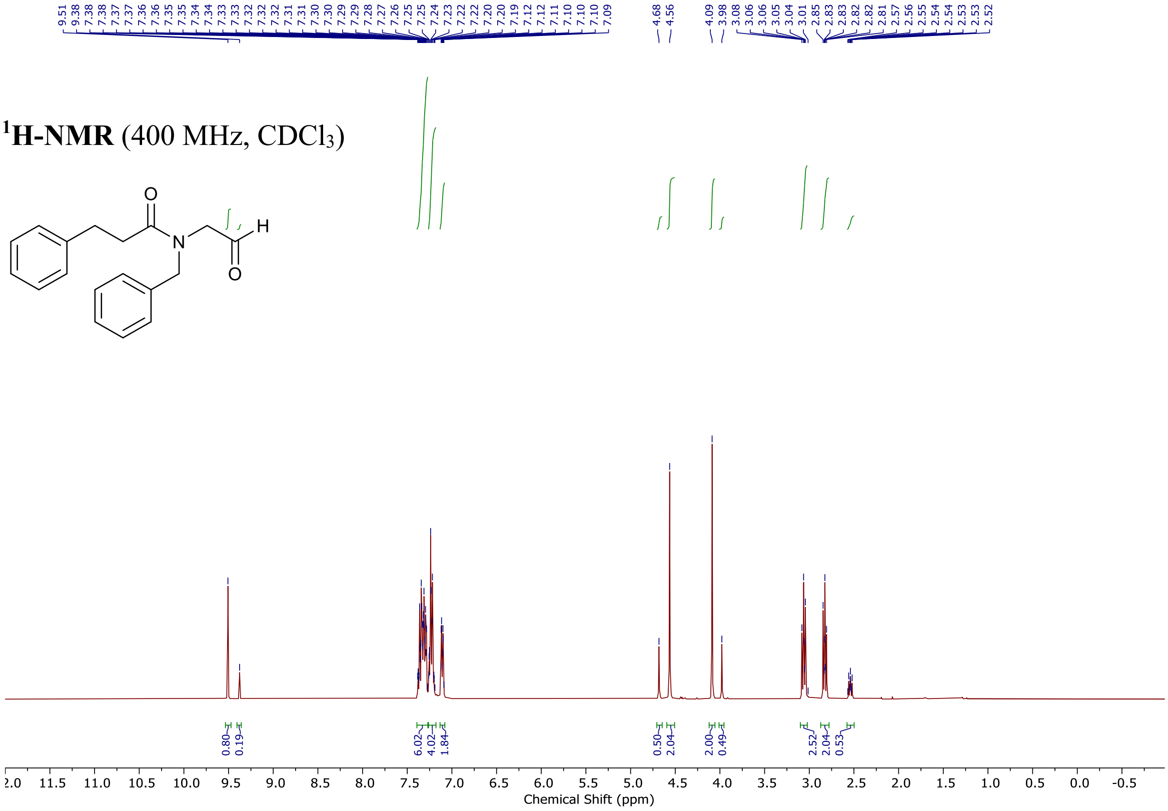

[13]**C-NMR** (100 MHz, $CDCl_3$)

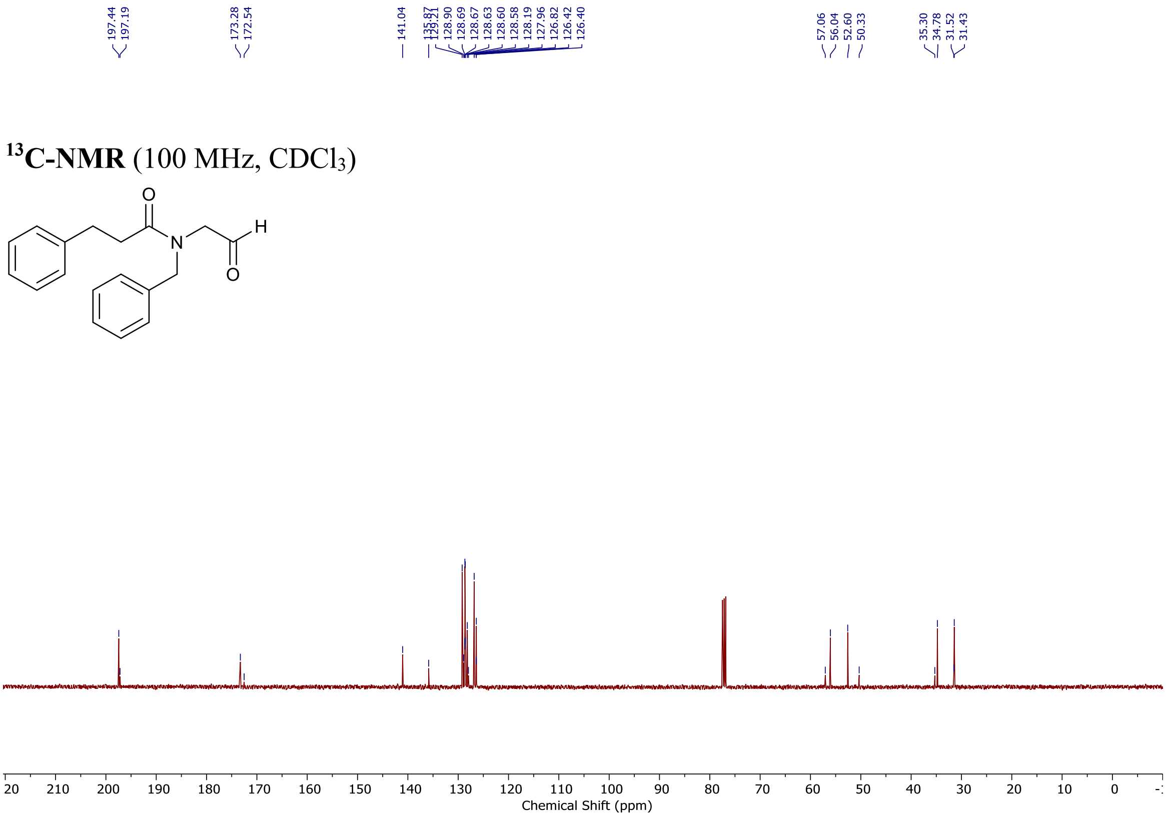

N-methyl-*N*-(2-oxoethyl)-3-phenylpropanamide, 1s

^{1}H-NMR (400 MHz, $CDCl_3$)

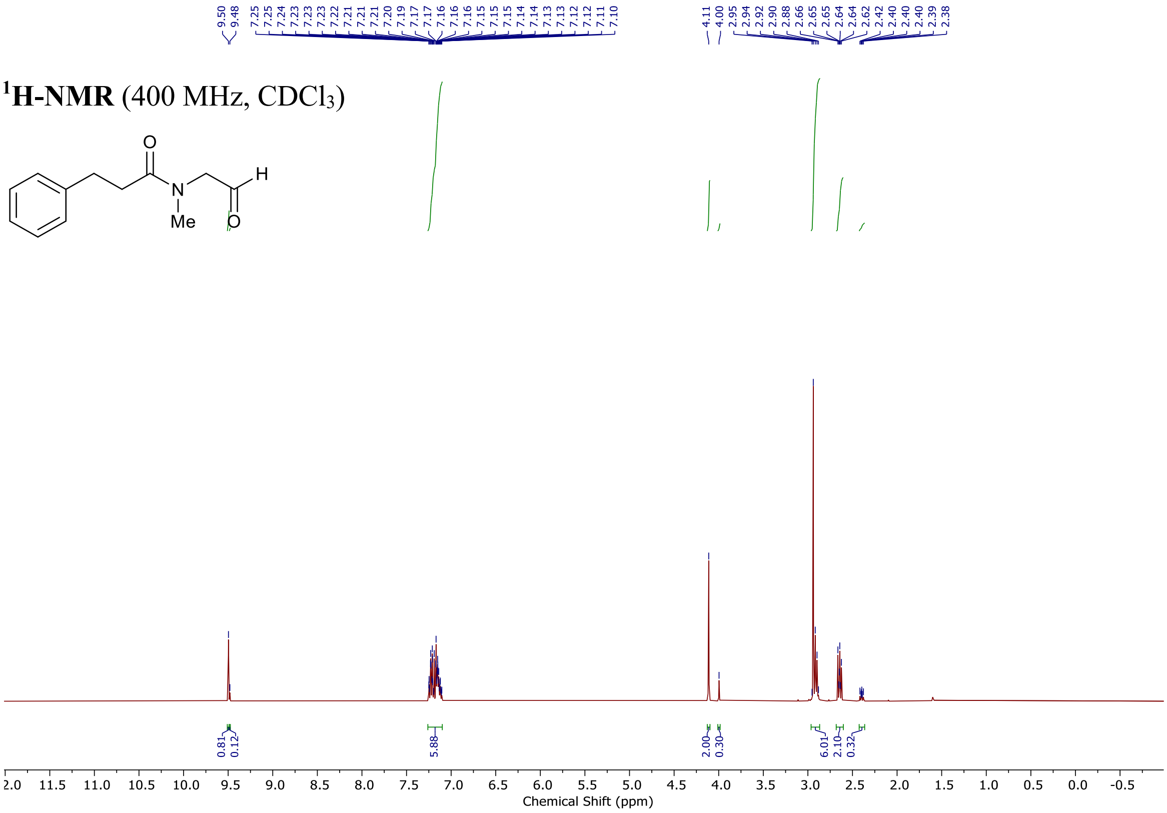

^{13}C-NMR (100 MHz, $CDCl_3$)

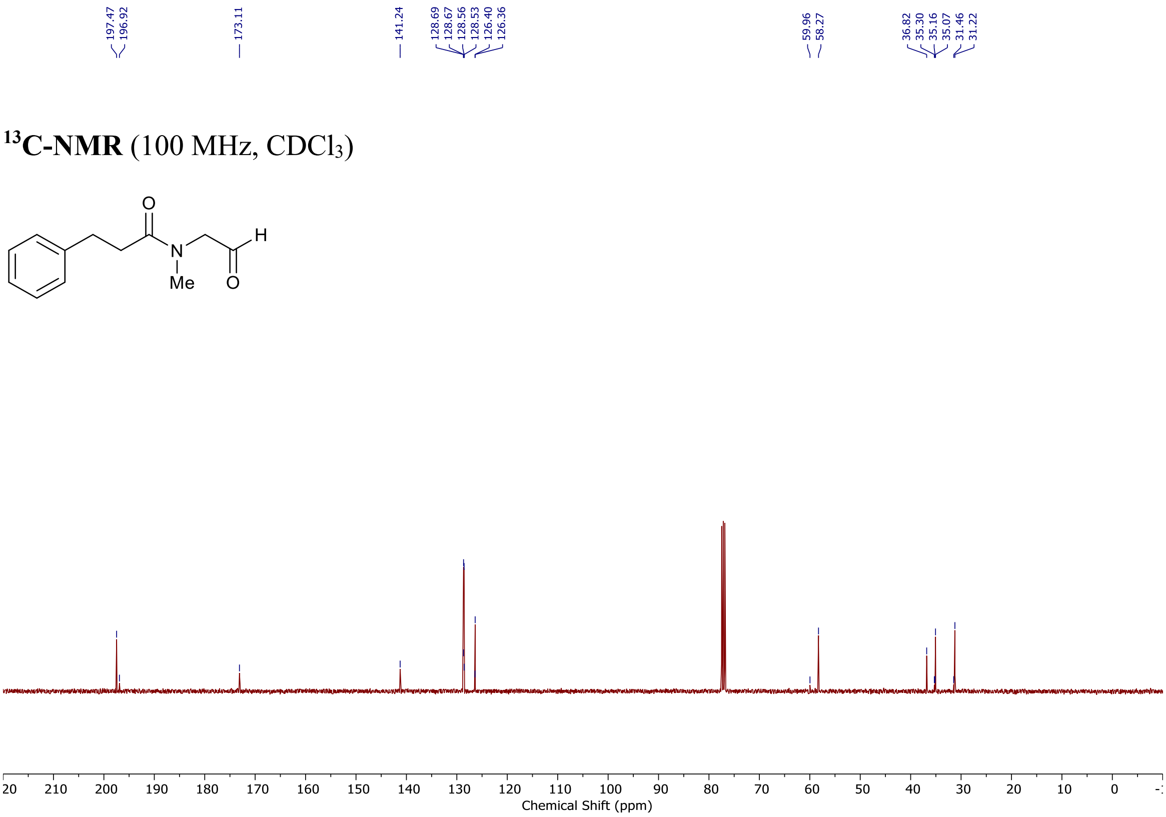

N-(2-oxoethyl)-N,3-diphenylpropanamide, 1t

^{1}H-NMR (400 MHz, $CDCl_3$)

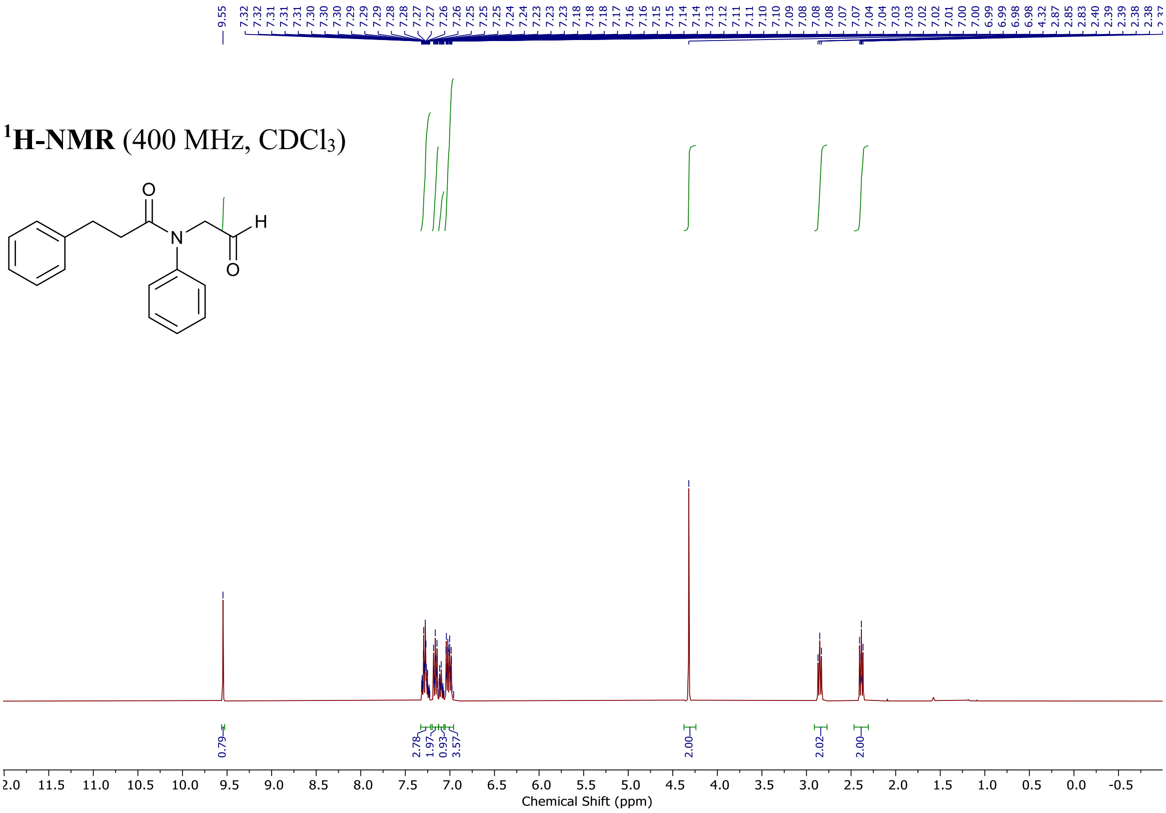

^{13}C-NMR (100 MHz, $CDCl_3$)

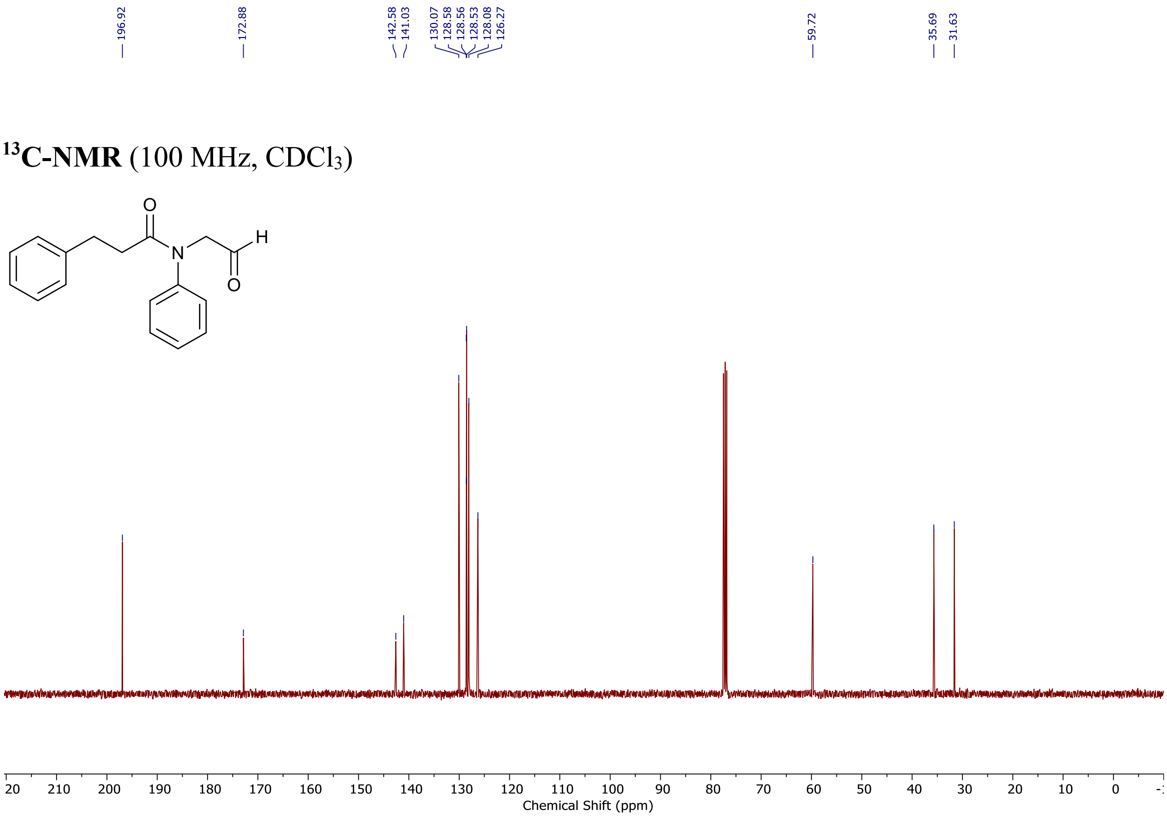

1-(3-phenylpropanoyl)pyrrolidine-2-carbaldehyde, 1u

^{1}H-NMR (400 MHz, $CDCl_3$)

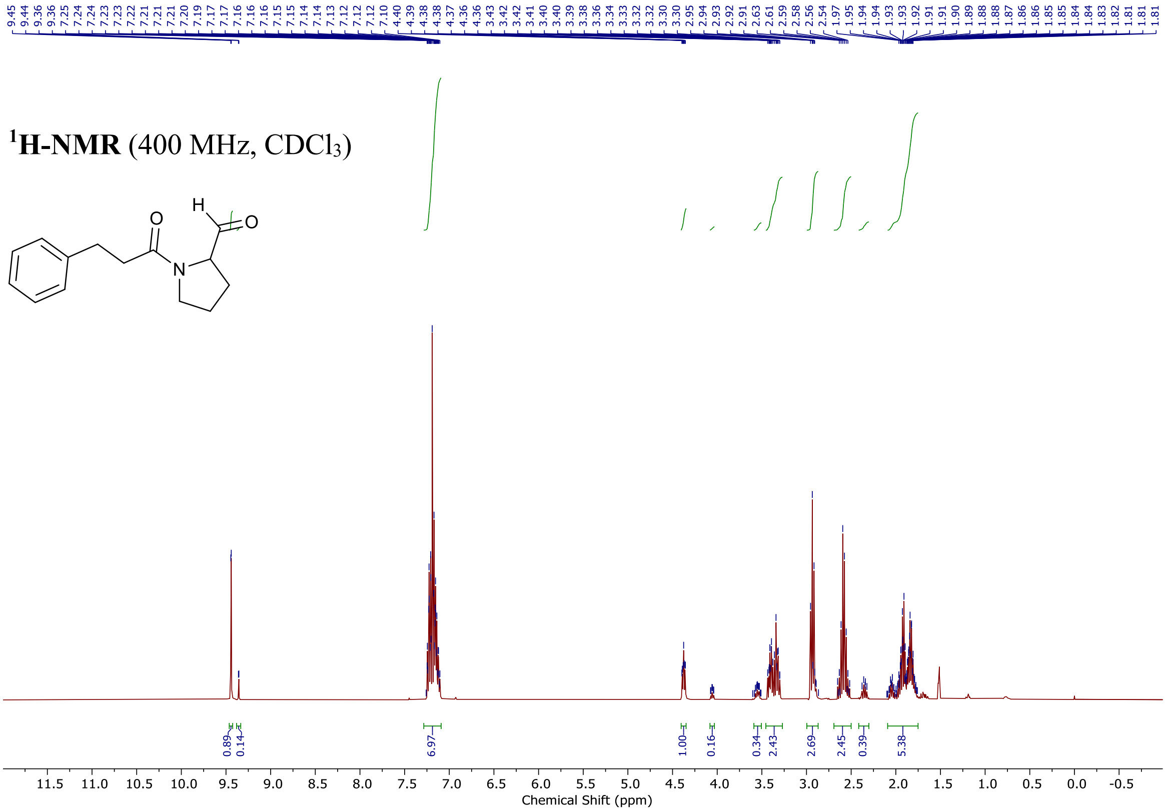

^{13}C-NMR (100 MHz, $CDCl_3$)

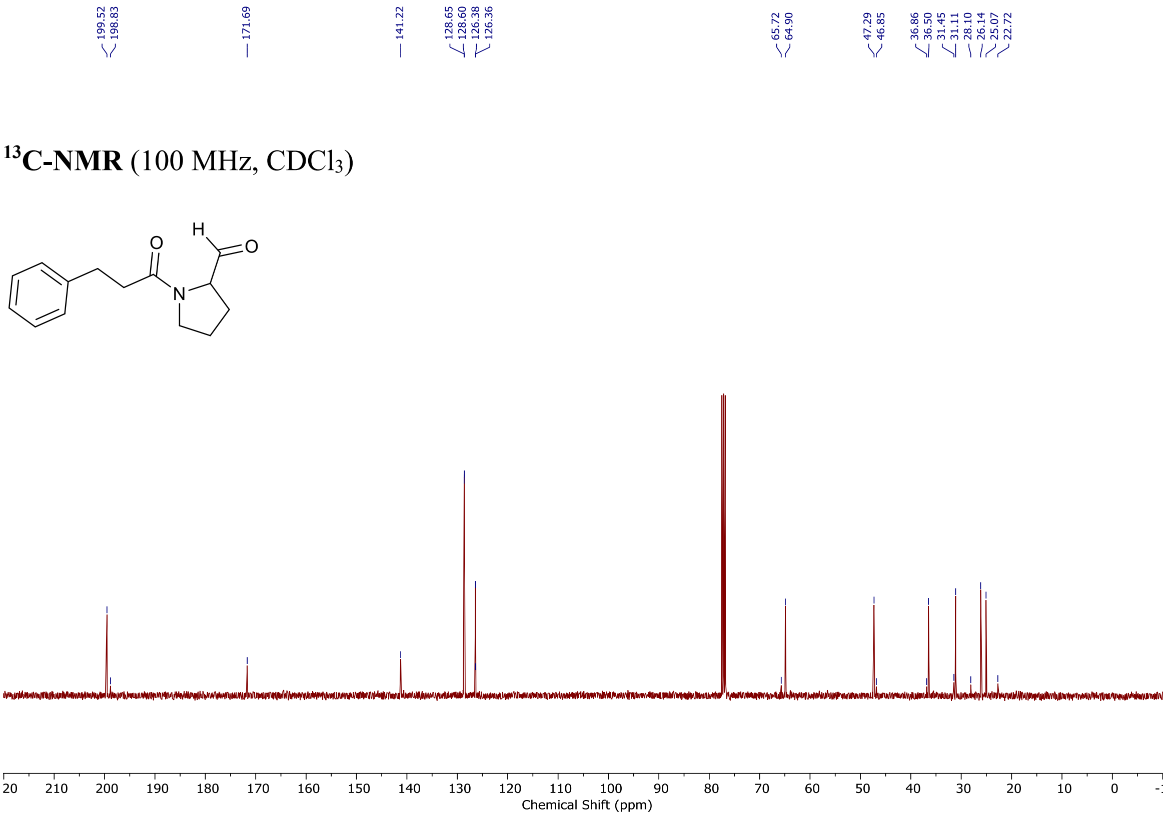

N-(2-oxoethyl)-3-phenylpropanamide, 1v

^{1}H-NMR (400 MHz, $CDCl_3$)

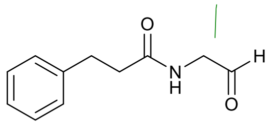

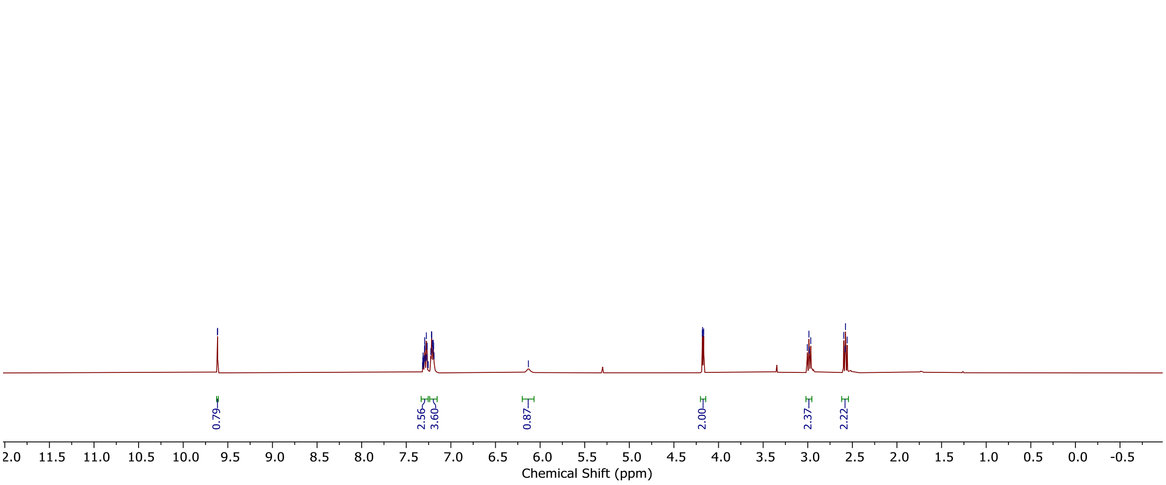

^{13}C-NMR (100 MHz, $CDCl_3$)

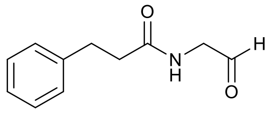

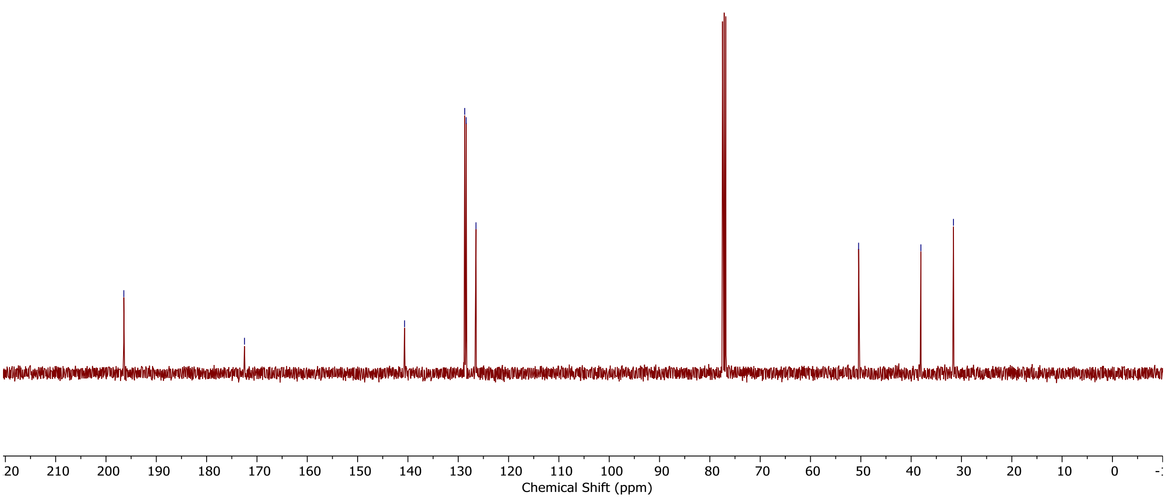

N-(1-oxo-3-phenylpropan-2-yl)-3-phenylpropanamide, 1w

^{1}H-NMR (400 MHz, $CDCl_3$)

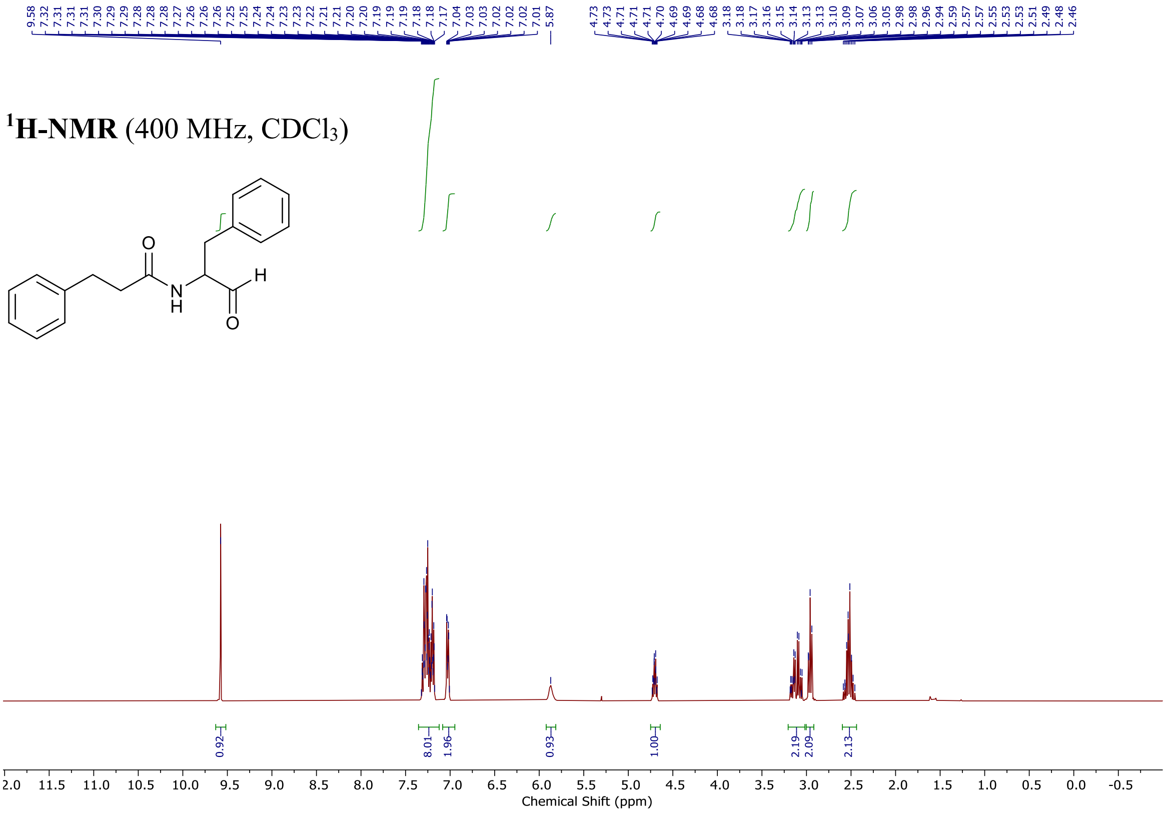

^{13}C-NMR (100 MHz, $CDCl_3$)

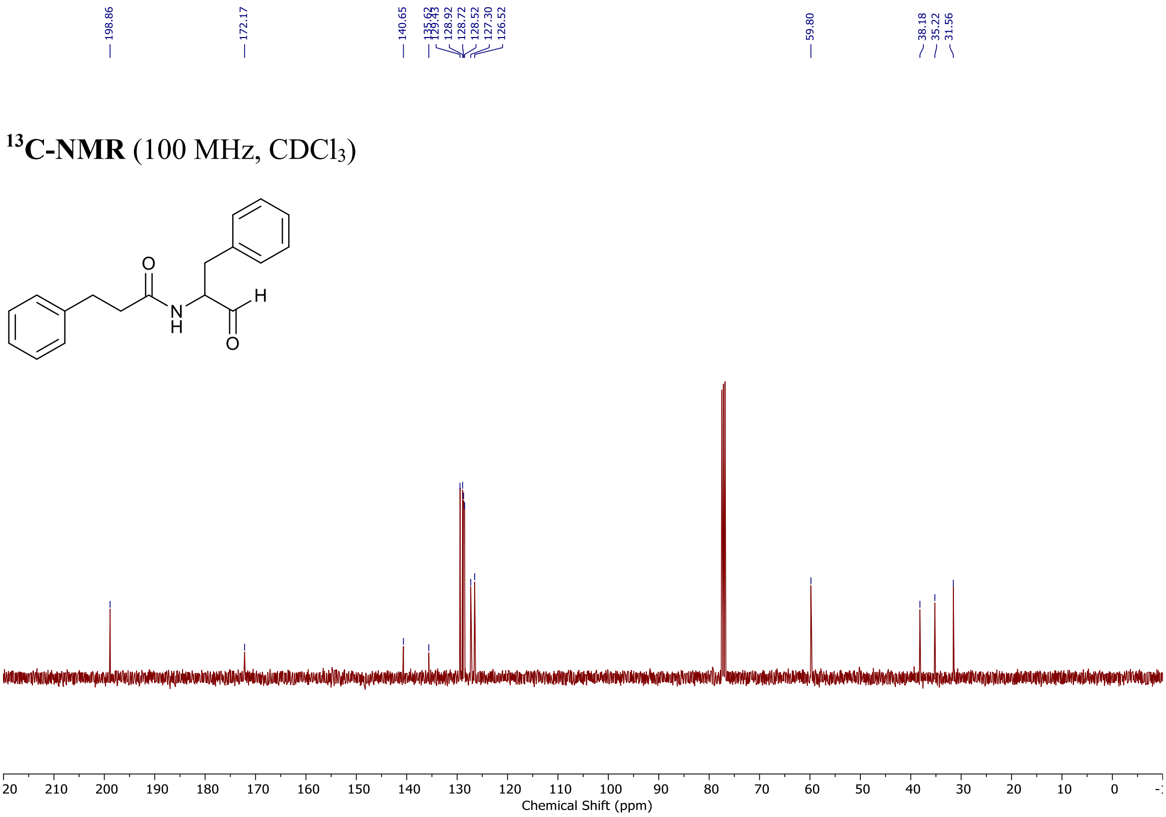

N-(1-oxopropan-2-yl)-3-phenylpropanamide, 1x

^{1}H-NMR (400 MHz, $CDCl_3$)

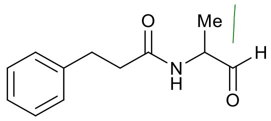

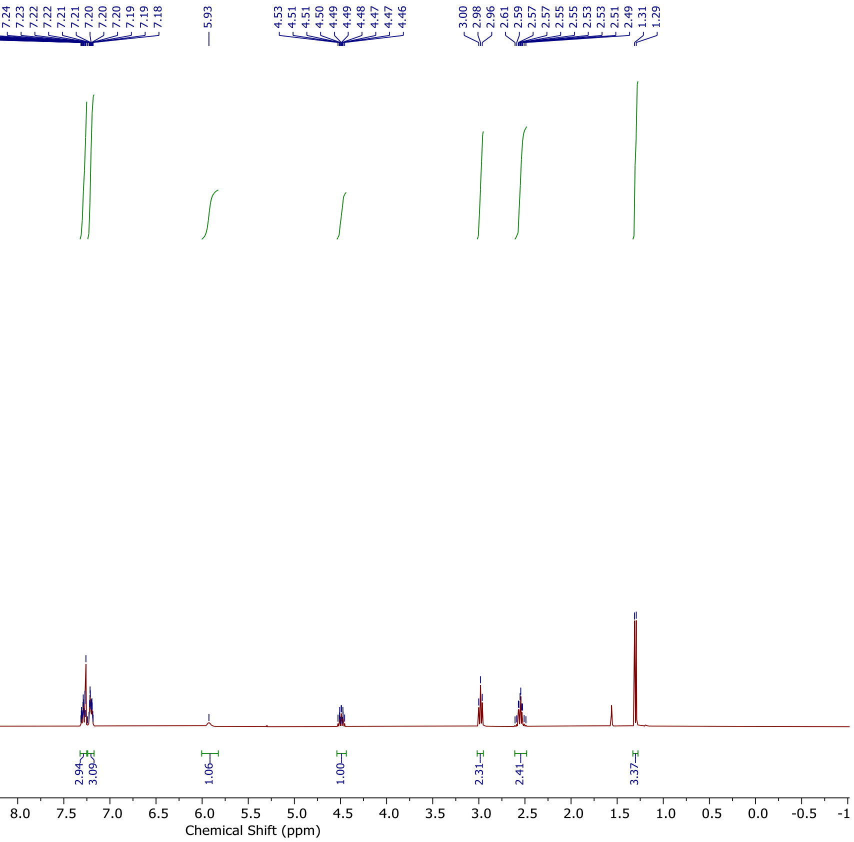

^{13}C-NMR (100 MHz, $CDCl_3$)

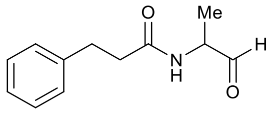

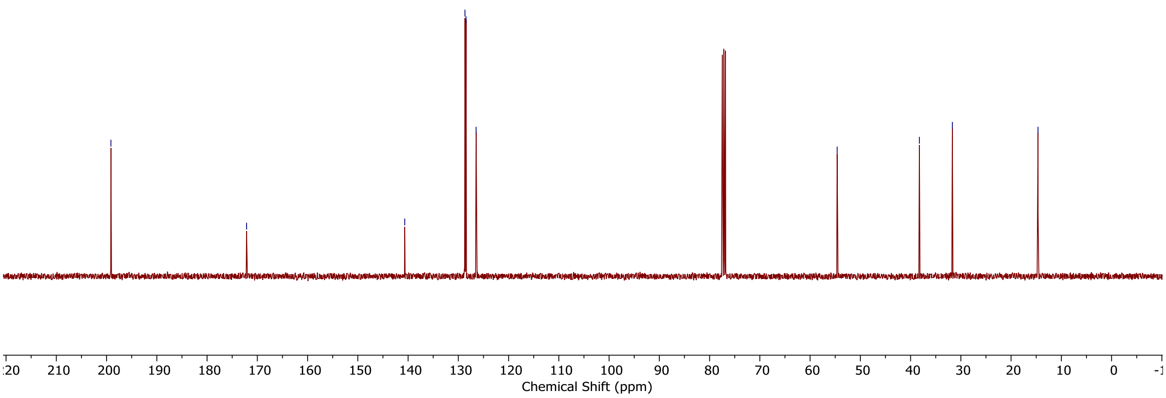

***N*-(4-methyl-1-oxopentan-2-yl)-3-phenylpropanamide, 1y**

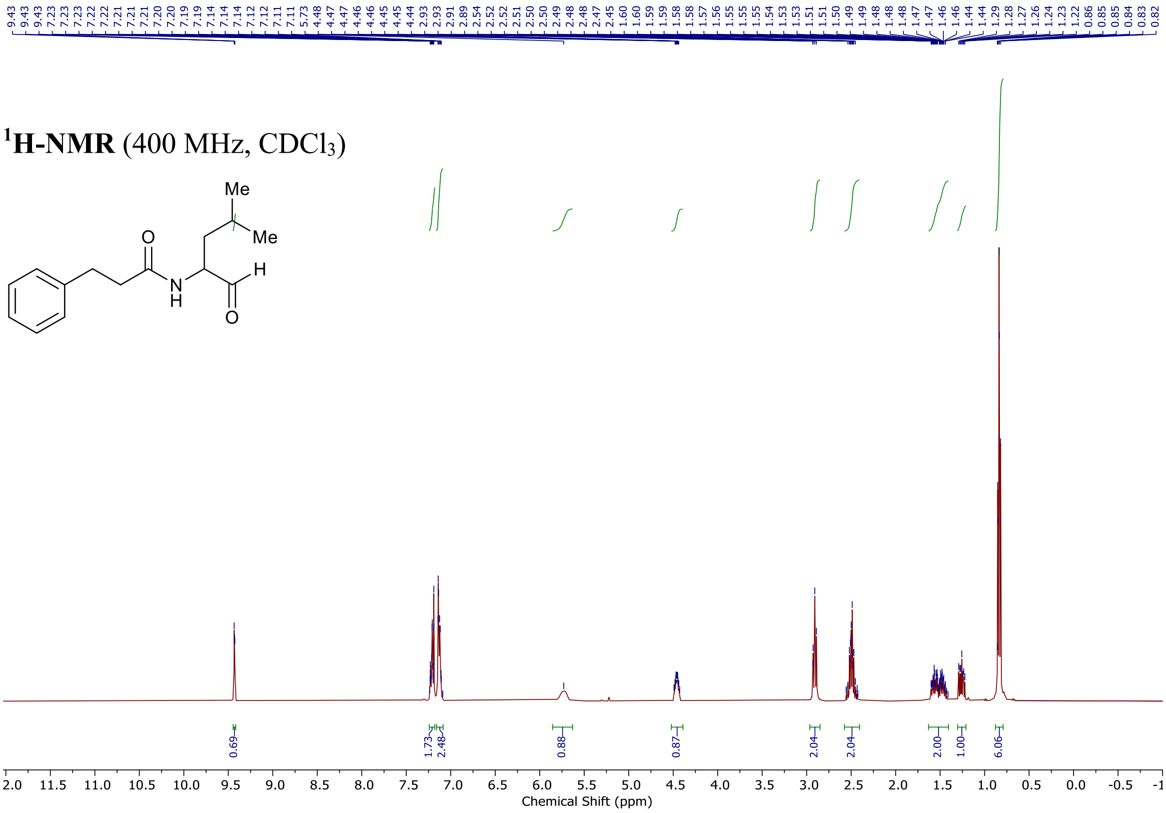

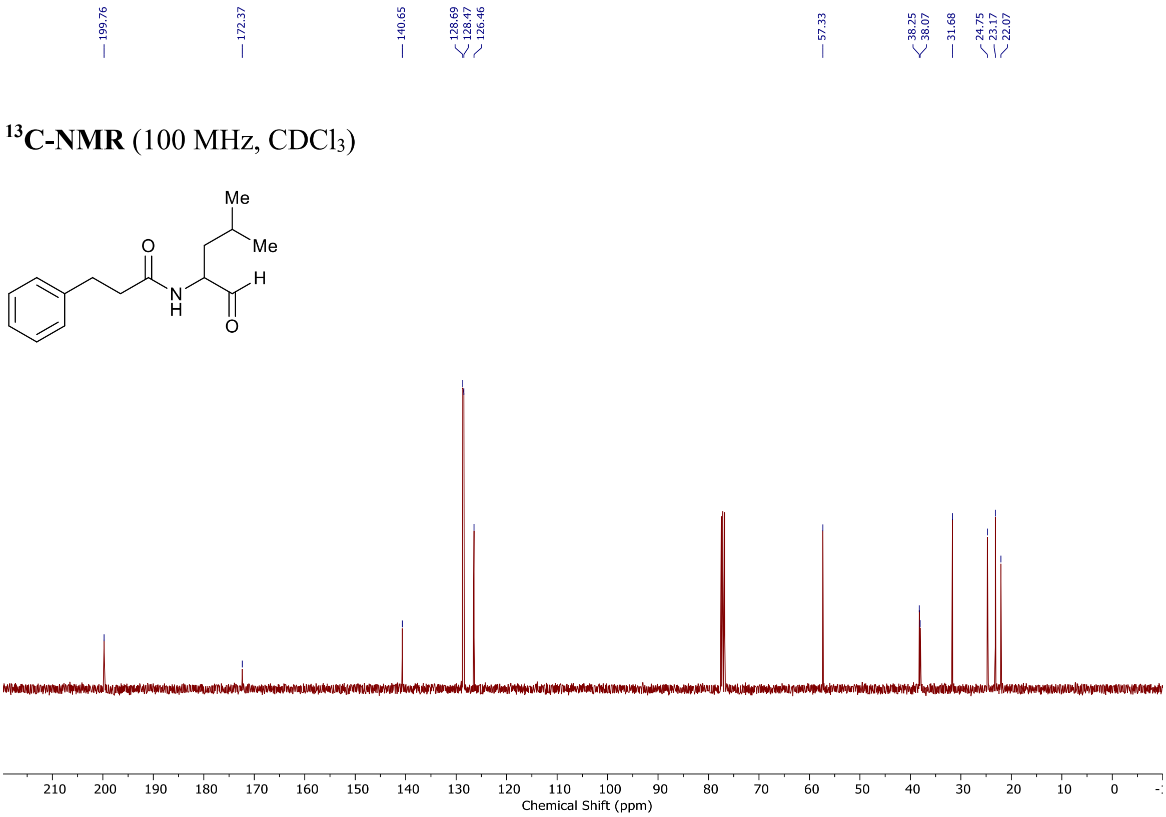

N-(3-methyl-1-oxobutan-2-yl)-3-phenylpropanamide, 1z

^{1}H-NMR (400 MHz, $CDCl_3$)

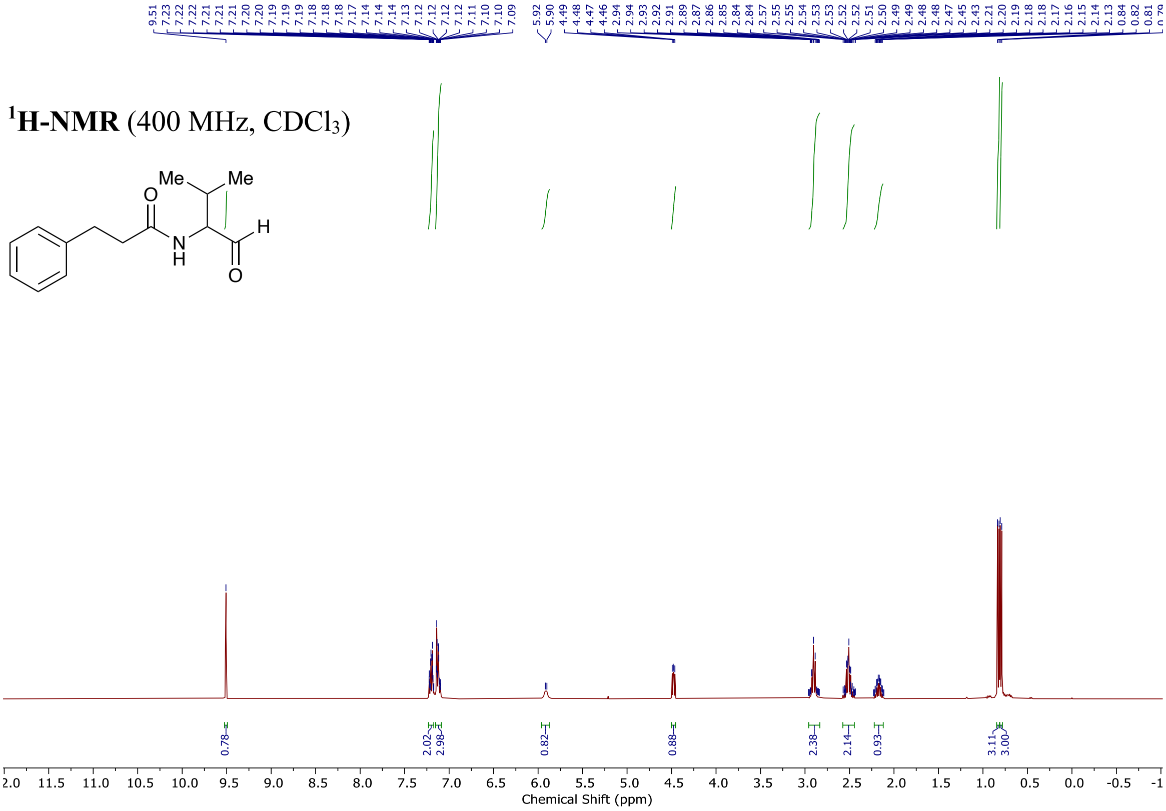

^{13}C-NMR (100 MHz, $CDCl_3$)

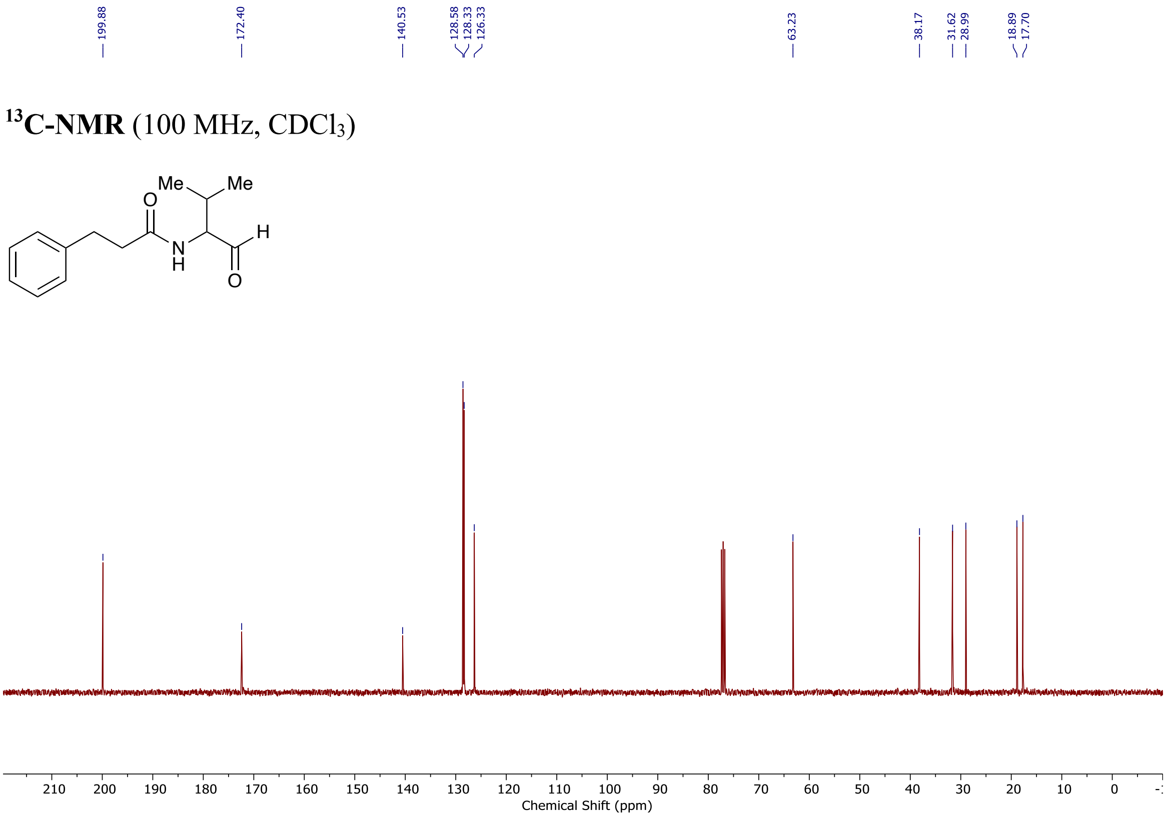

N-benzyl-*N*-(2-oxoethyl)acetamide, 1ab

[1]**H-NMR** (400 MHz, $CDCl_3$)

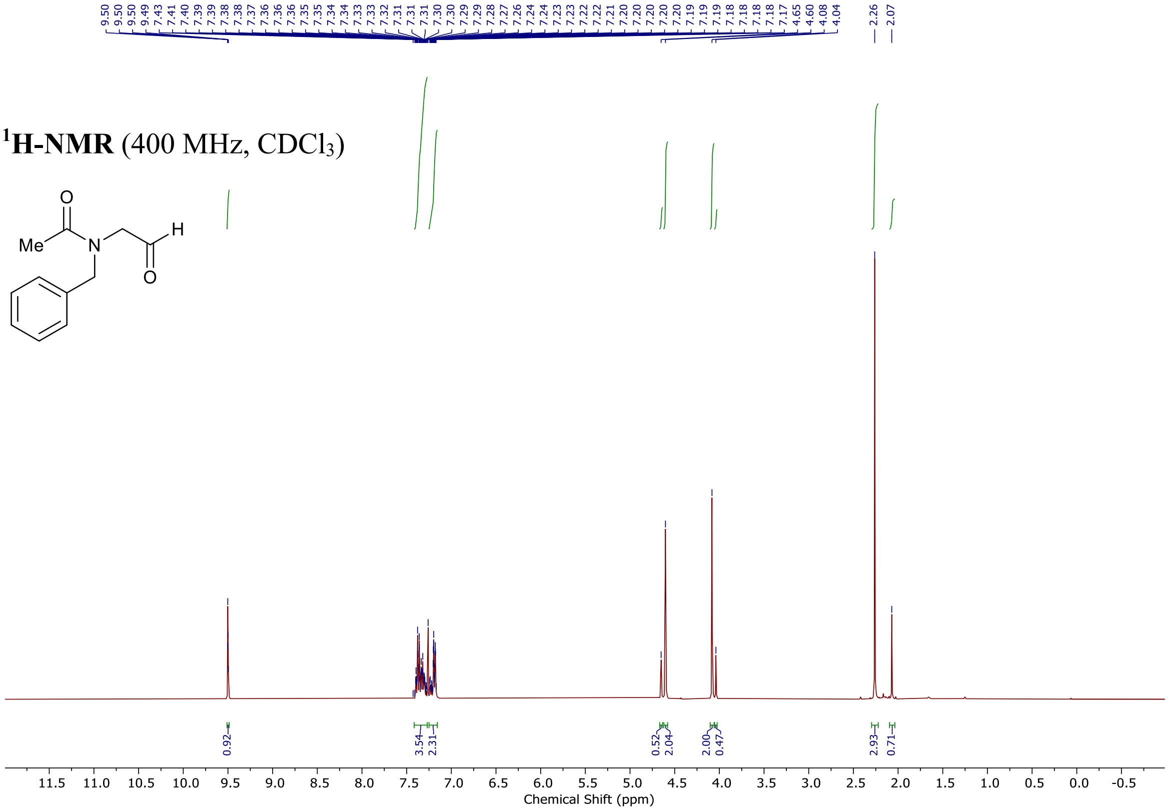

[13]**C-NMR** (100 MHz, $CDCl_3$)

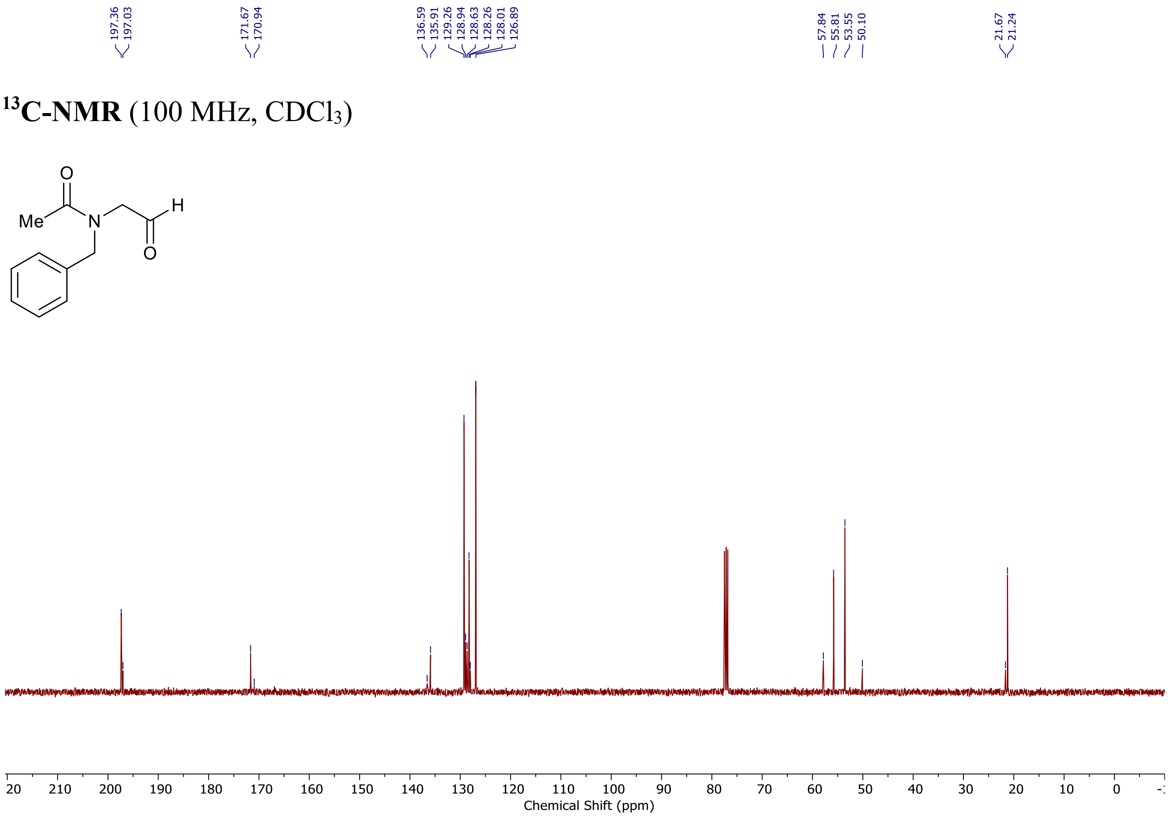

Benzyl benzyl(2-oxoethyl)carbamate, 1ac

^{1}H-NMR (400 MHz, $CDCl_3$)

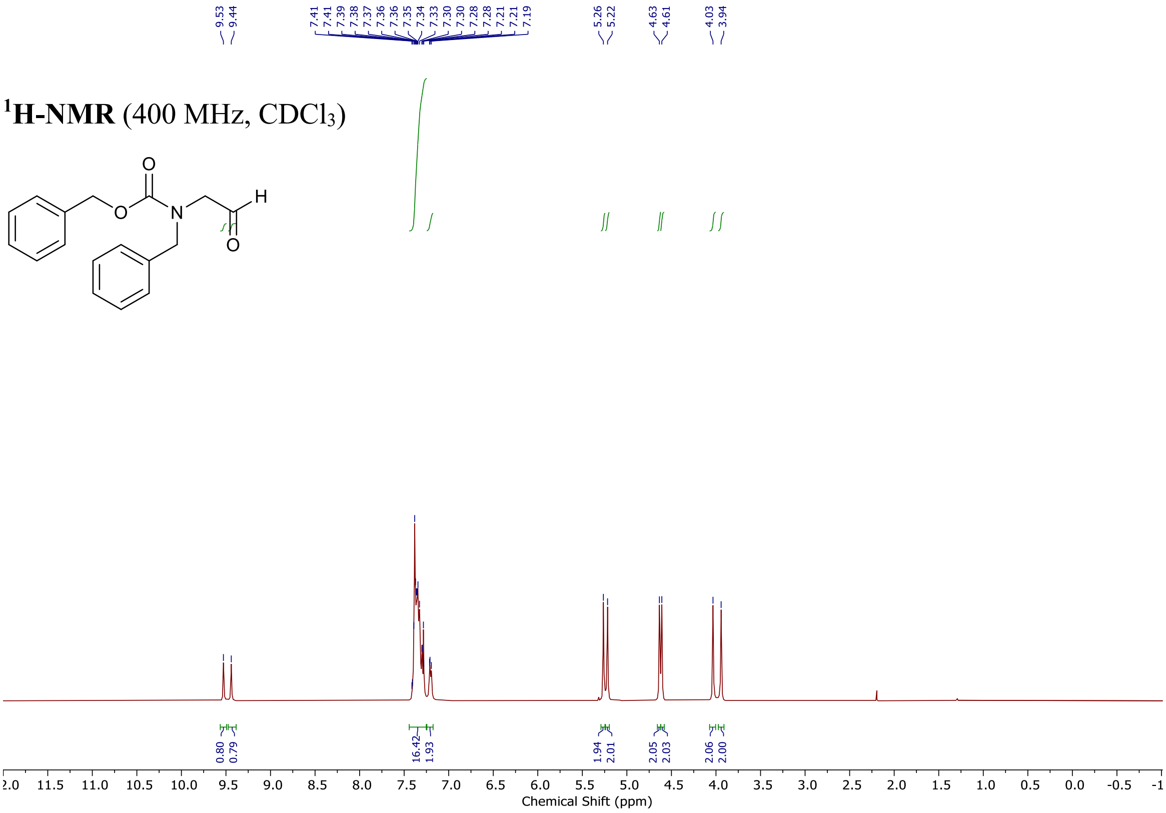

^{13}C-NMR (100 MHz, $CDCl_3$)

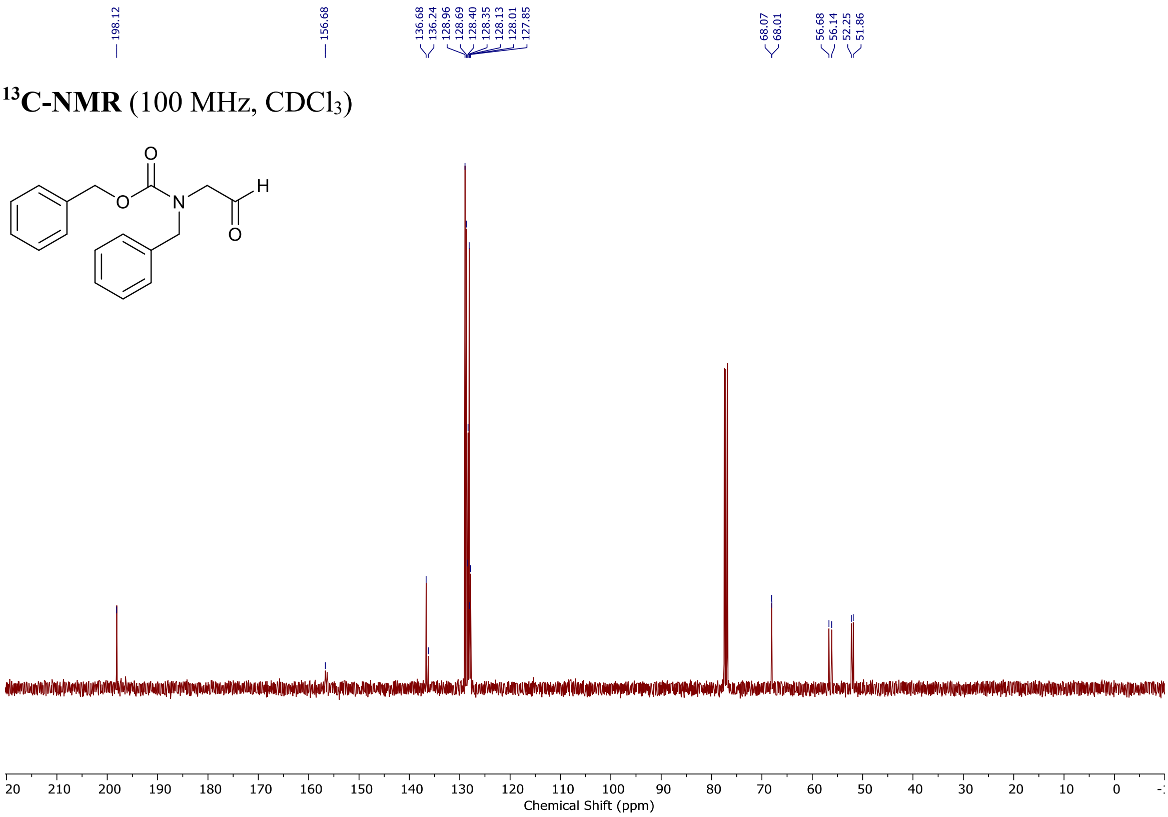

N-benzyl-*N*-(2-oxoethyl)cyclohexanecarboxamide, 1ad

¹H-NMR (400 MHz, $CDCl_3$)

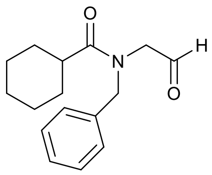

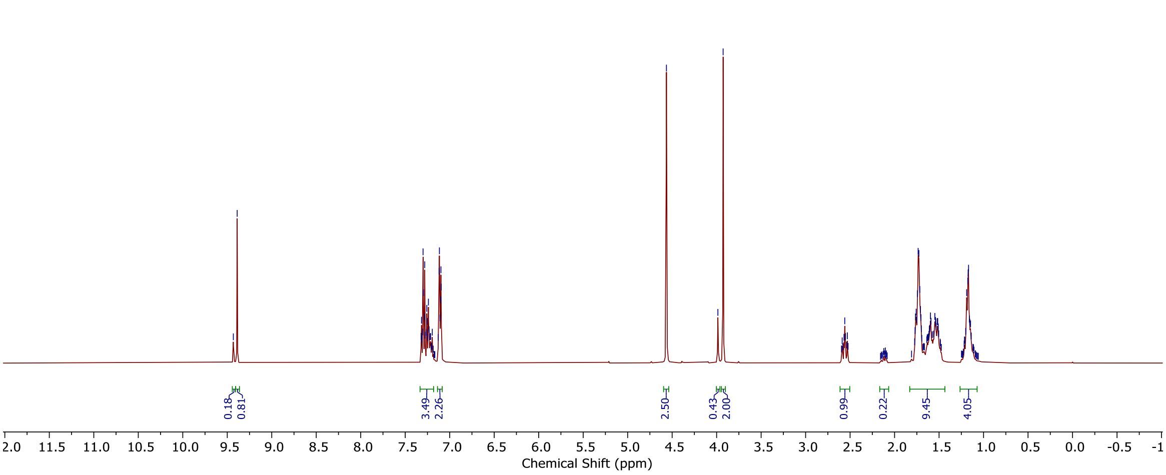

¹³C-NMR (100 MHz, $CDCl_3$)

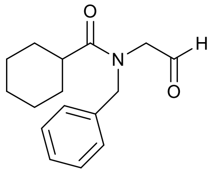

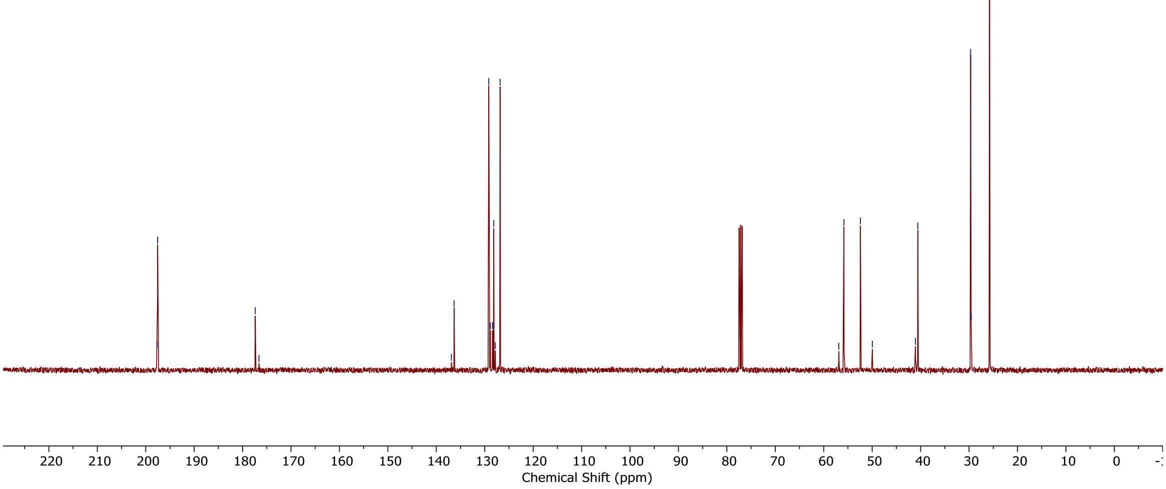

N-(1-oxo-3-phenylpropan-2-yl)cyclohexanecarboxamide, 1ae

^{1}H-NMR (400 MHz, $CDCl_3$)

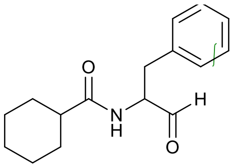

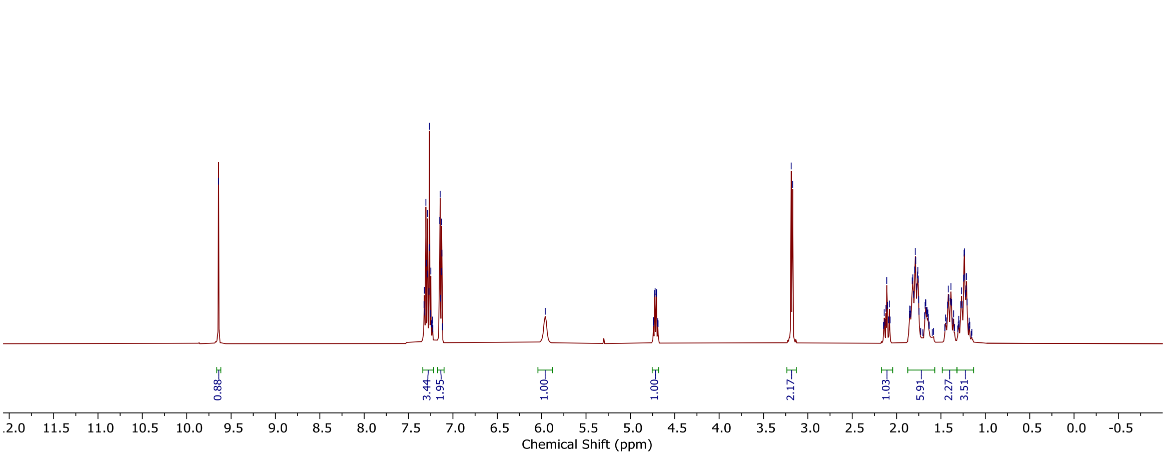

^{13}C-NMR (100 MHz, $CDCl_3$)

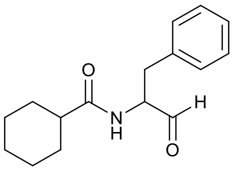

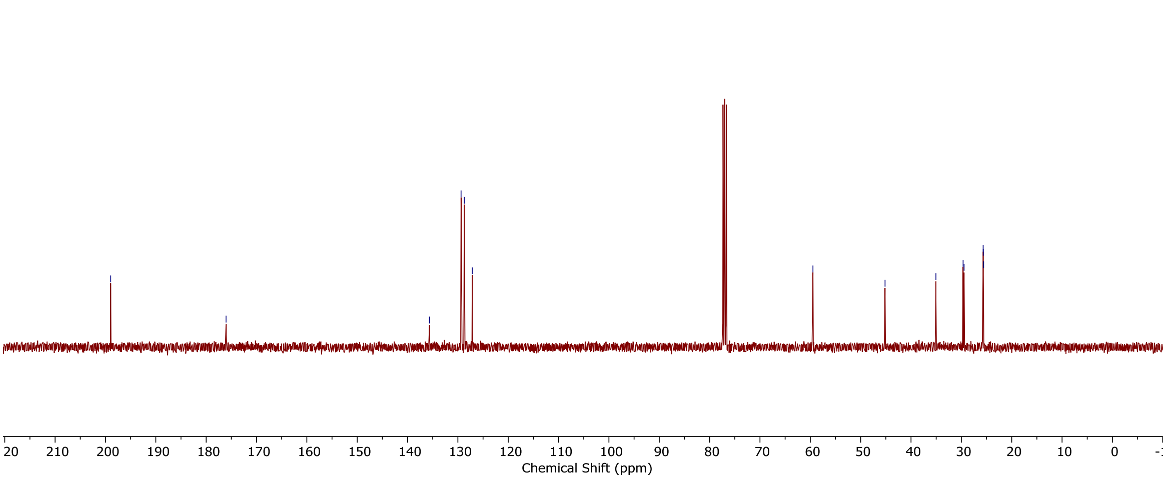

(*E*)-*N*-benzyl-*N*-(2-oxodec-3-en-1-yl)-3-phenylpropanamide, 2a

^{1}H-NMR (400 MHz, $CDCl_3$)

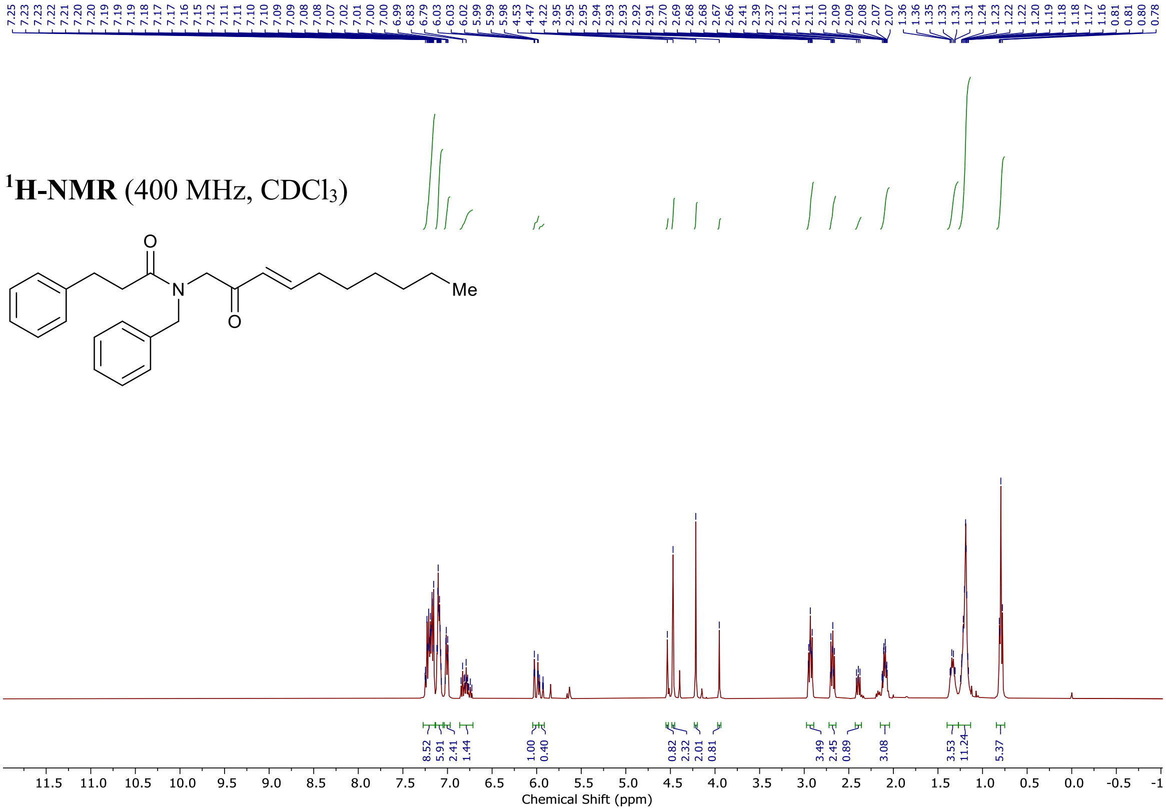

^{13}C-NMR (100 MHz, $CDCl_3$)

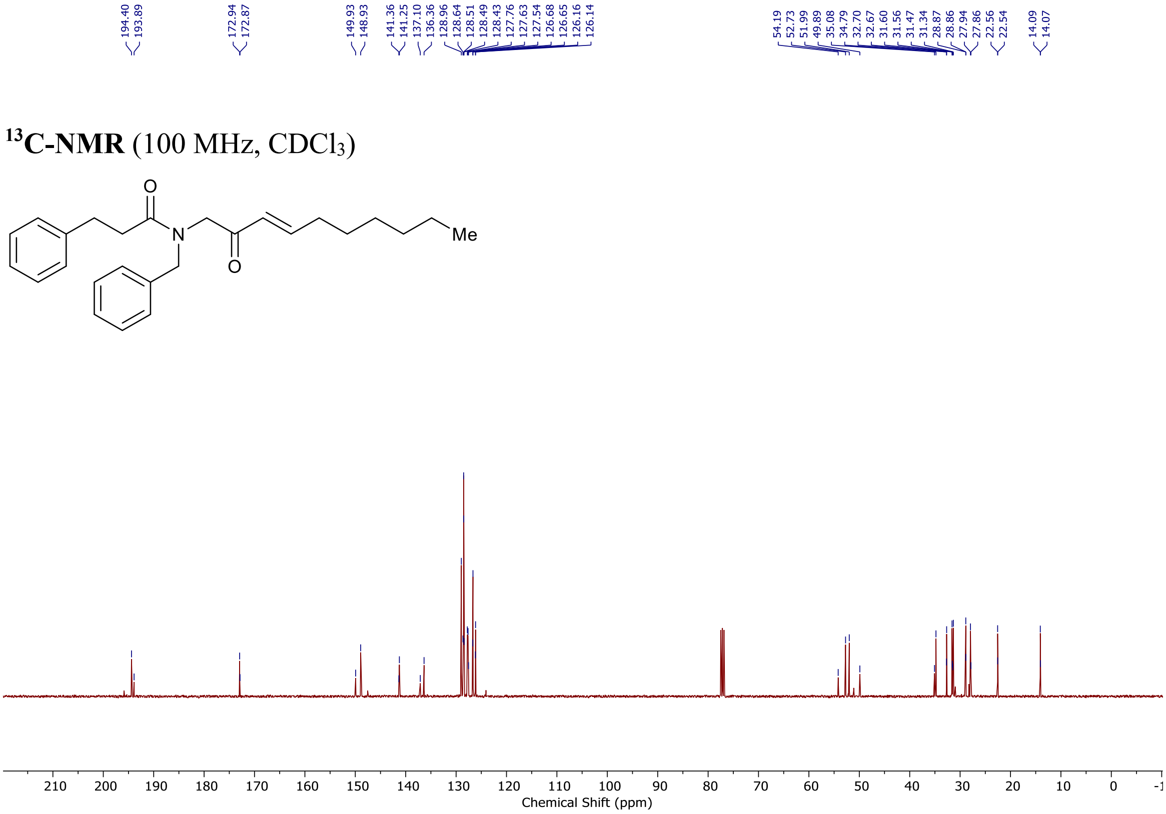

(*E*)-*N*-benzyl-*N*-(7-methyl-2-oxooct-3-en-1-yl)-3-phenylpropanamide, 2b

^{1}H-NMR (400 MHz, $CDCl_3$)

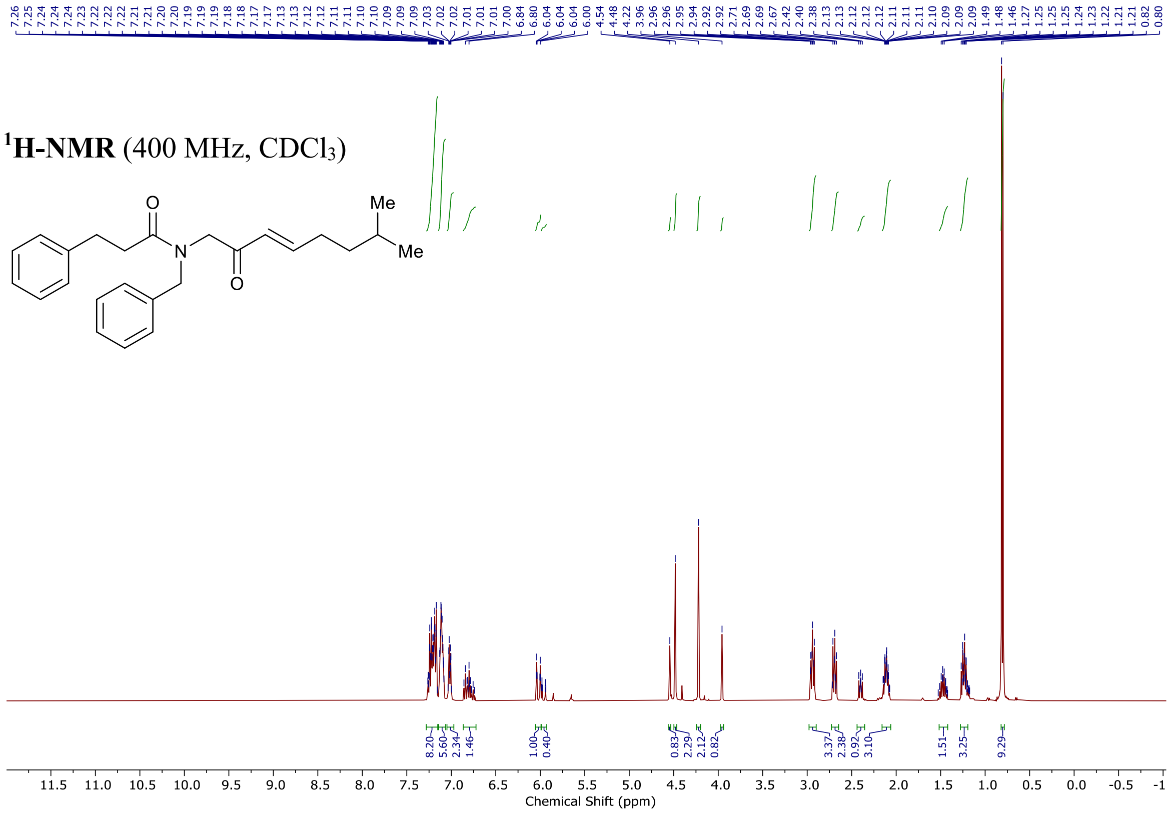

^{13}C-NMR (100 MHz, $CDCl_3$)

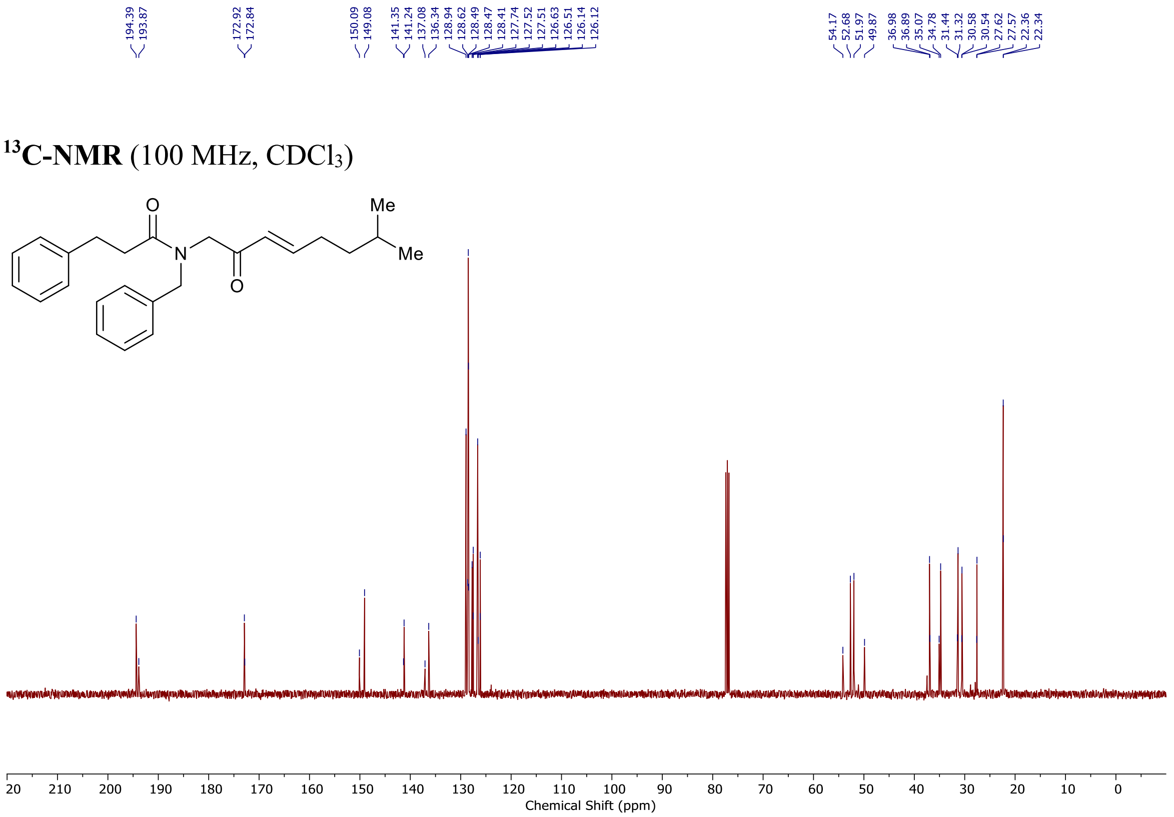

(*E*)-*N*-benzyl-*N*-(5-cyclohexyl-2-oxopent-3-en-1-yl)-3-phenylpropanamide, 2c

^{1}H-NMR (400 MHz, $CDCl_3$)

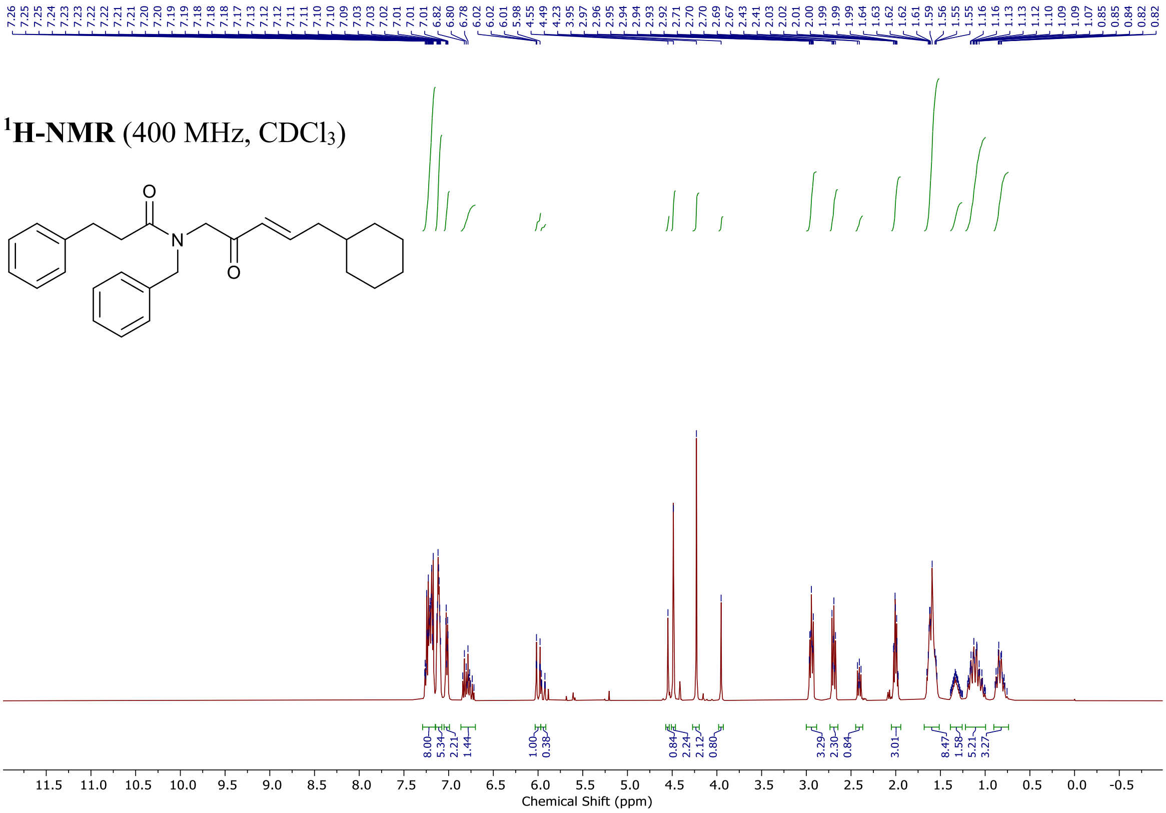

^{13}C-NMR (100 MHz, $CDCl_3$)

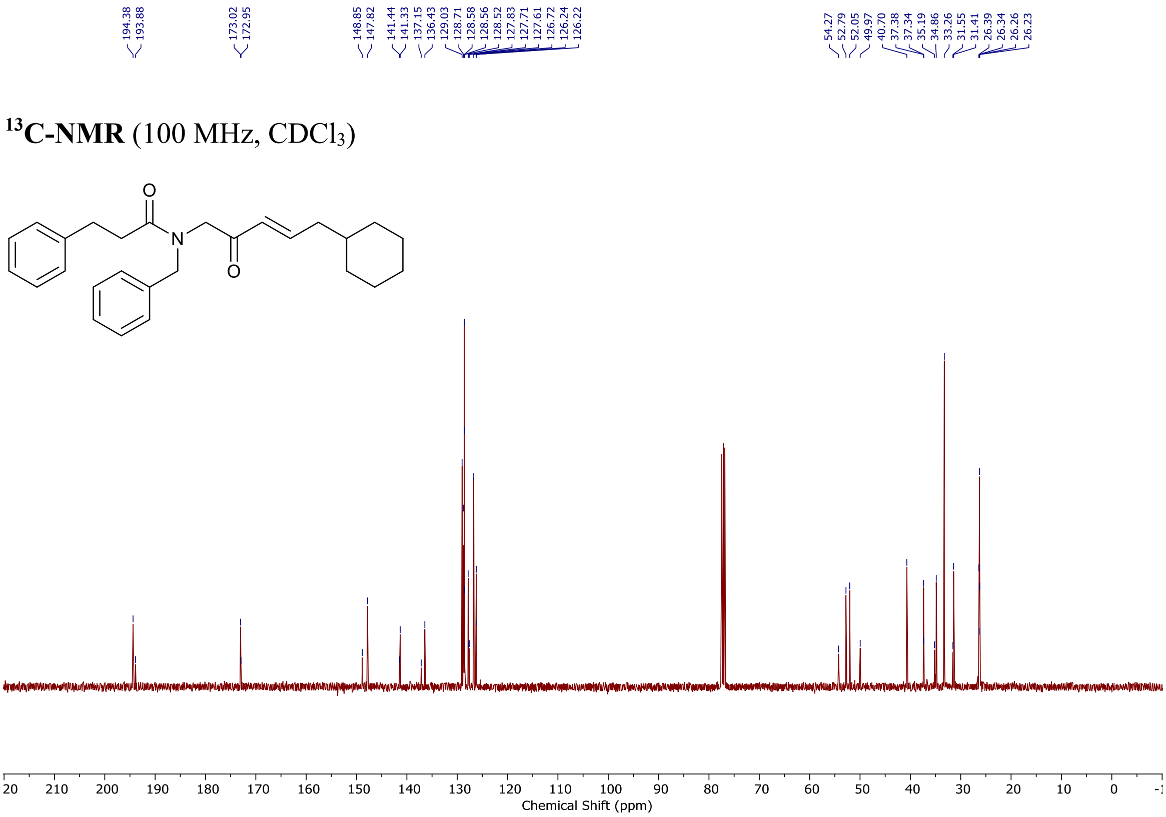

(*E*)-*N*-benzyl-*N*-(4-cyclopropyl-2-oxobut-3-en-1-yl)-3-phenylpropanamide, 2d

^{1}H-NMR (400 MHz, $CDCl_3$)

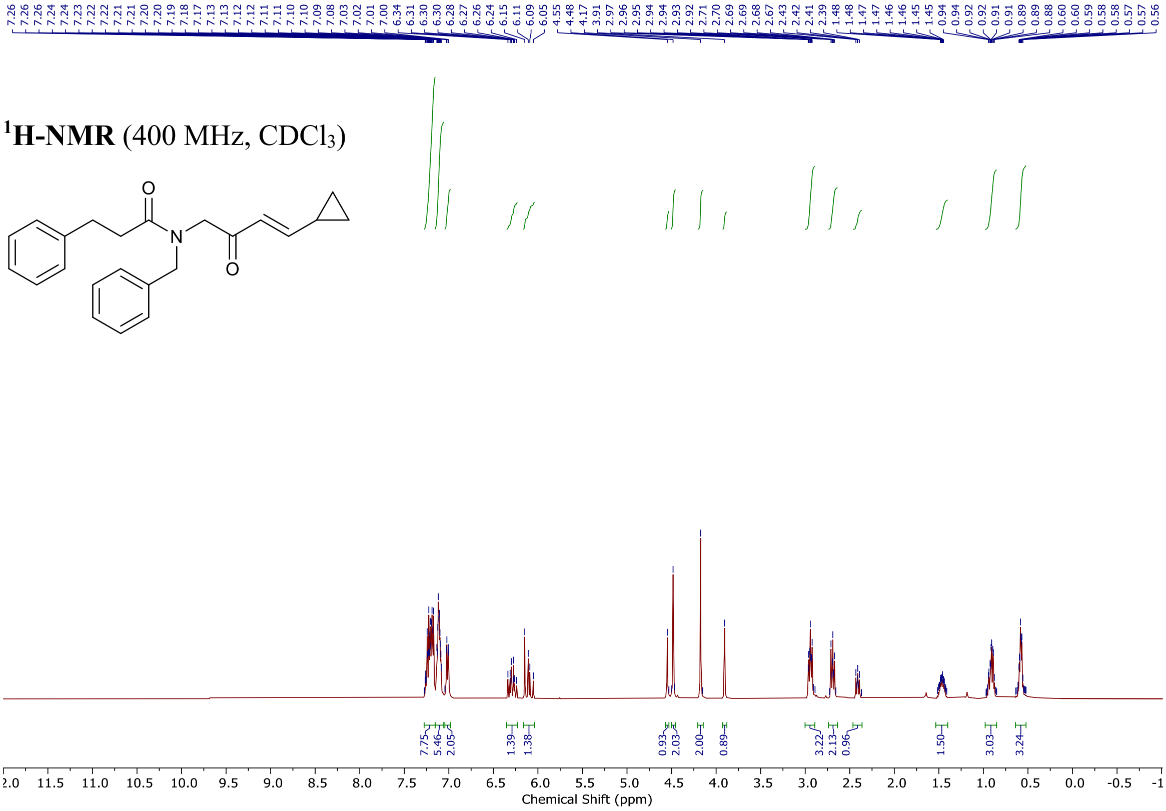

^{13}C-NMR (100 MHz, $CDCl_3$)

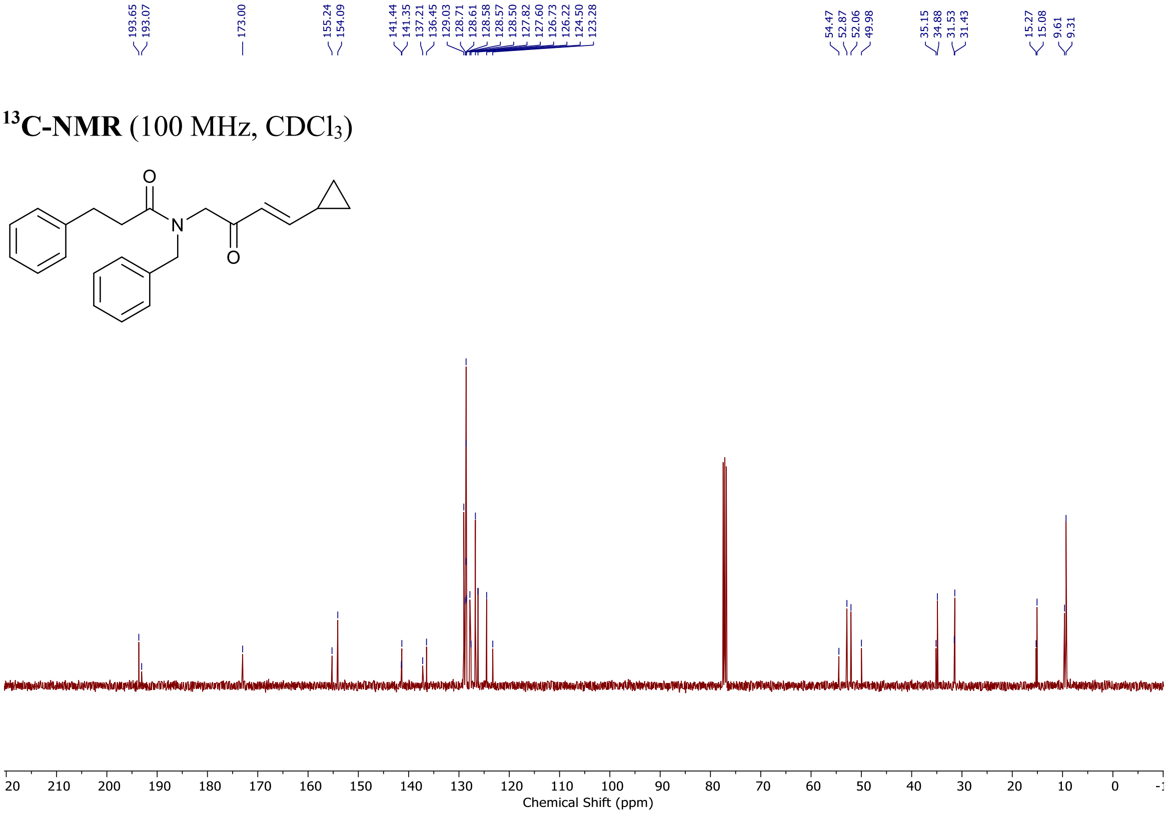

(*E*)-*N*-benzyl-*N*-(2-oxo-5-phenylpent-3-en-1-yl)-3-phenylpropanamide, 2e

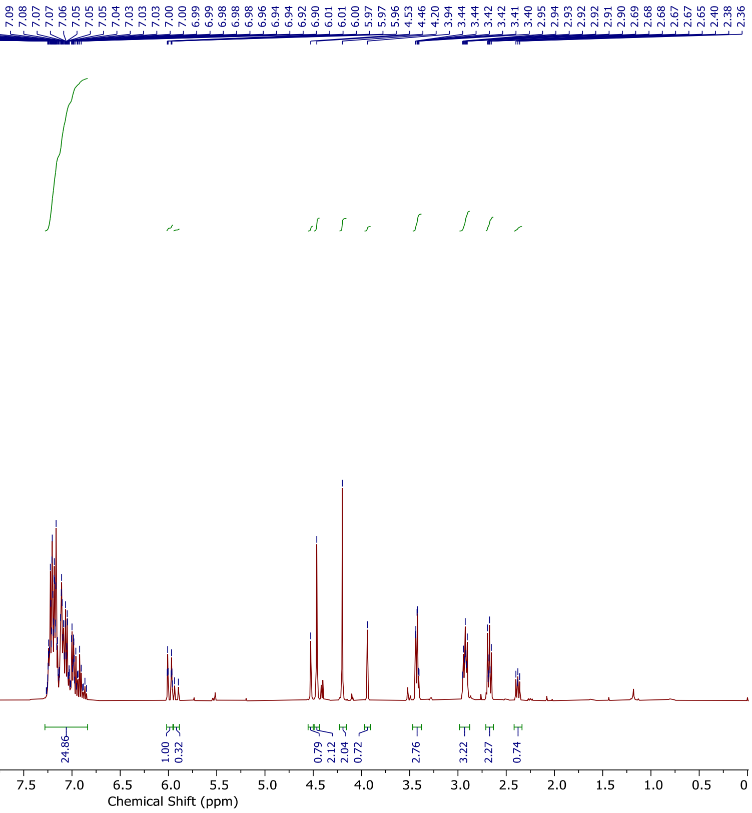

^{1}H-NMR (400 MHz, $CDCl_3$)

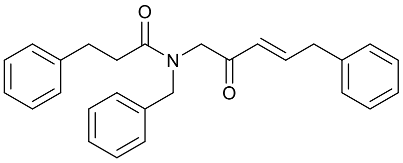

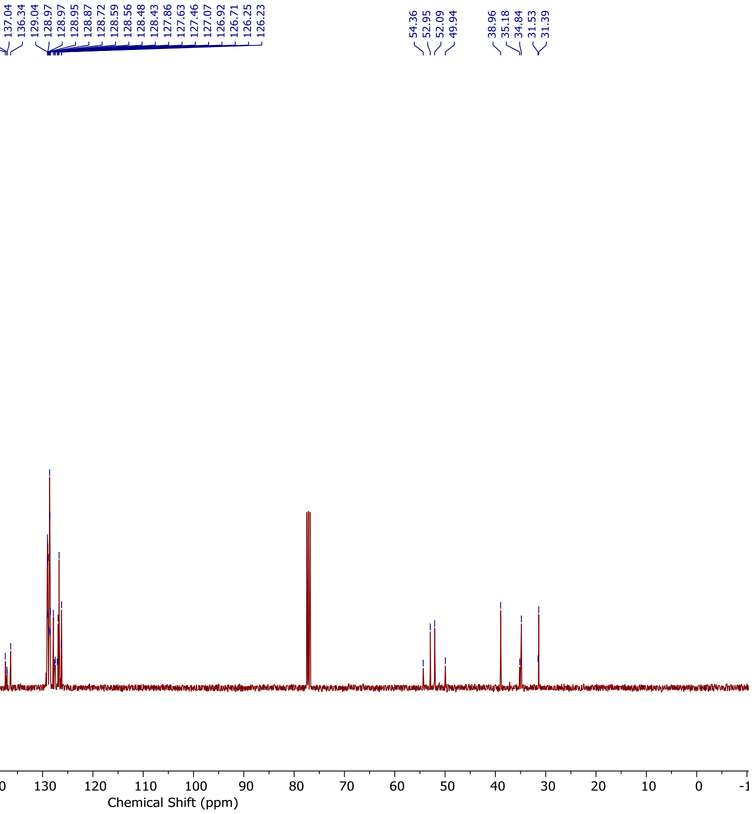

^{13}C-NMR (100 MHz, $CDCl_3$)

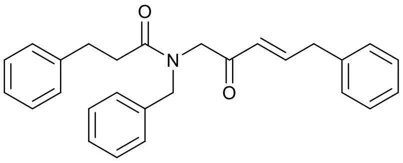

(*E*)-*N*-benzyl-*N*-(4-(cyclohex-1-en-1-yl)-2-oxobut-3-en-1-yl)-3-phenylpropanamide, 2f

^{1}H-NMR (400 MHz, $CDCl_3$)

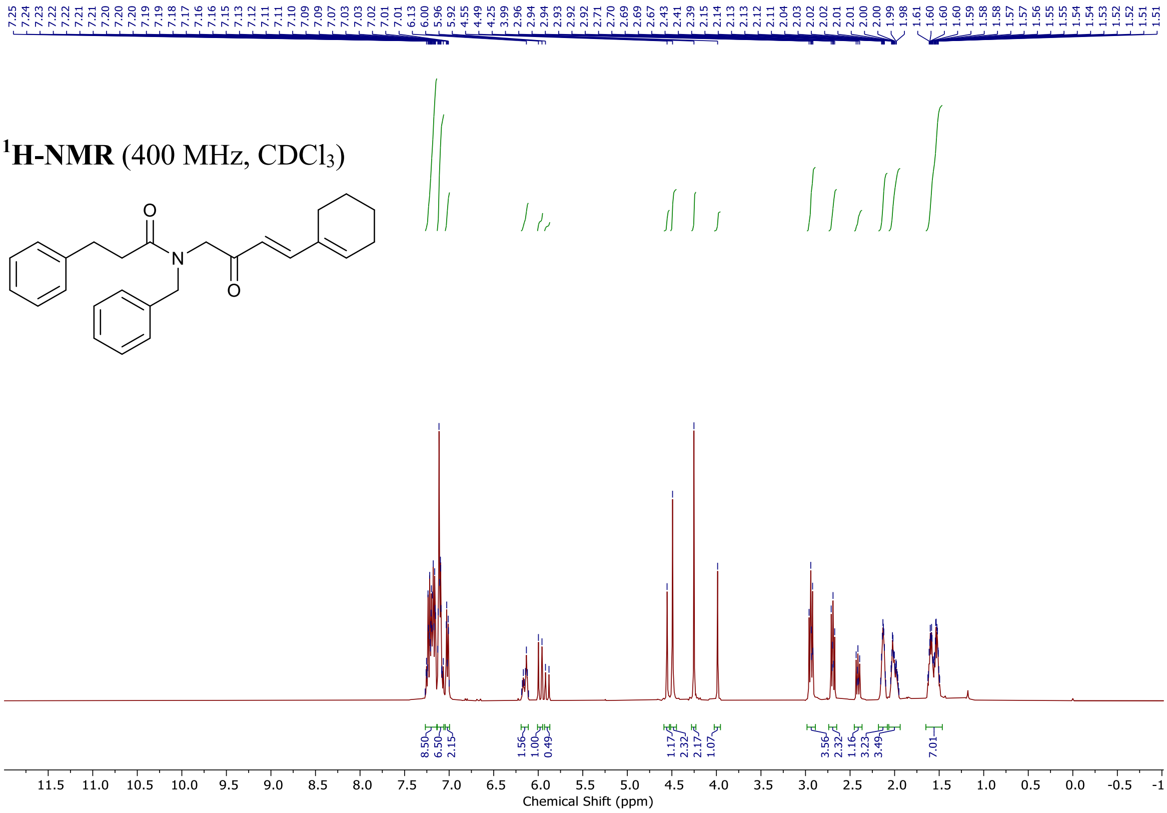

^{13}C-NMR (100 MHz, $CDCl_3$)

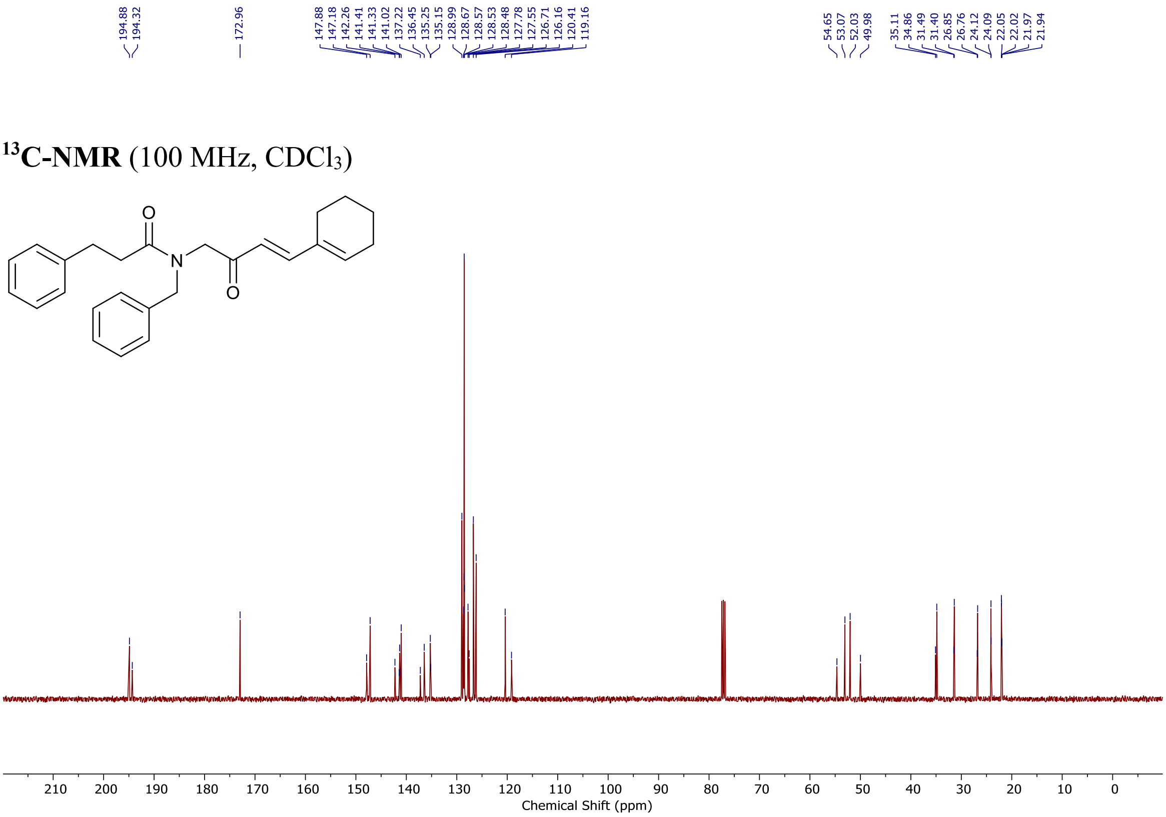

(*E*)-*N*-benzyl-*N*-(2-oxo-4-phenylbut-3-en-1-yl)-3-phenylpropanamide, 2g

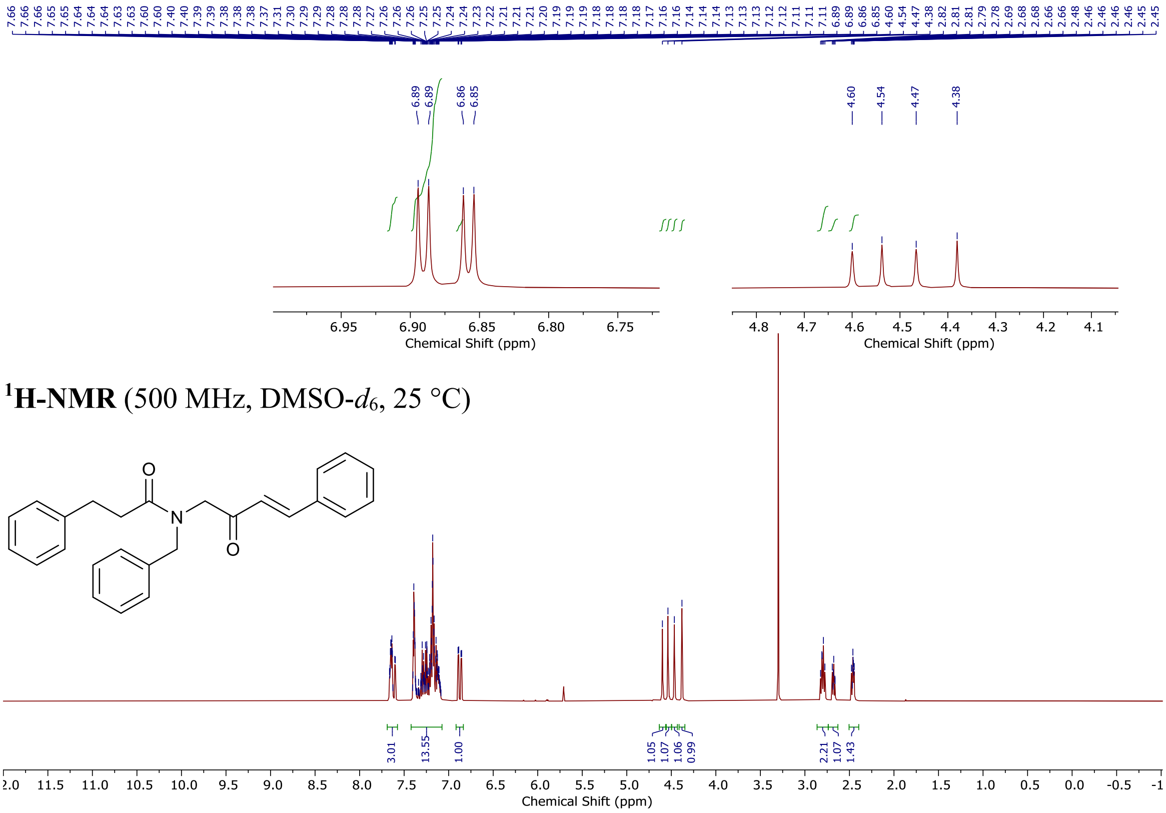

^{1}H-NMR (500 MHz, DMSO-d_6, 25 °C)

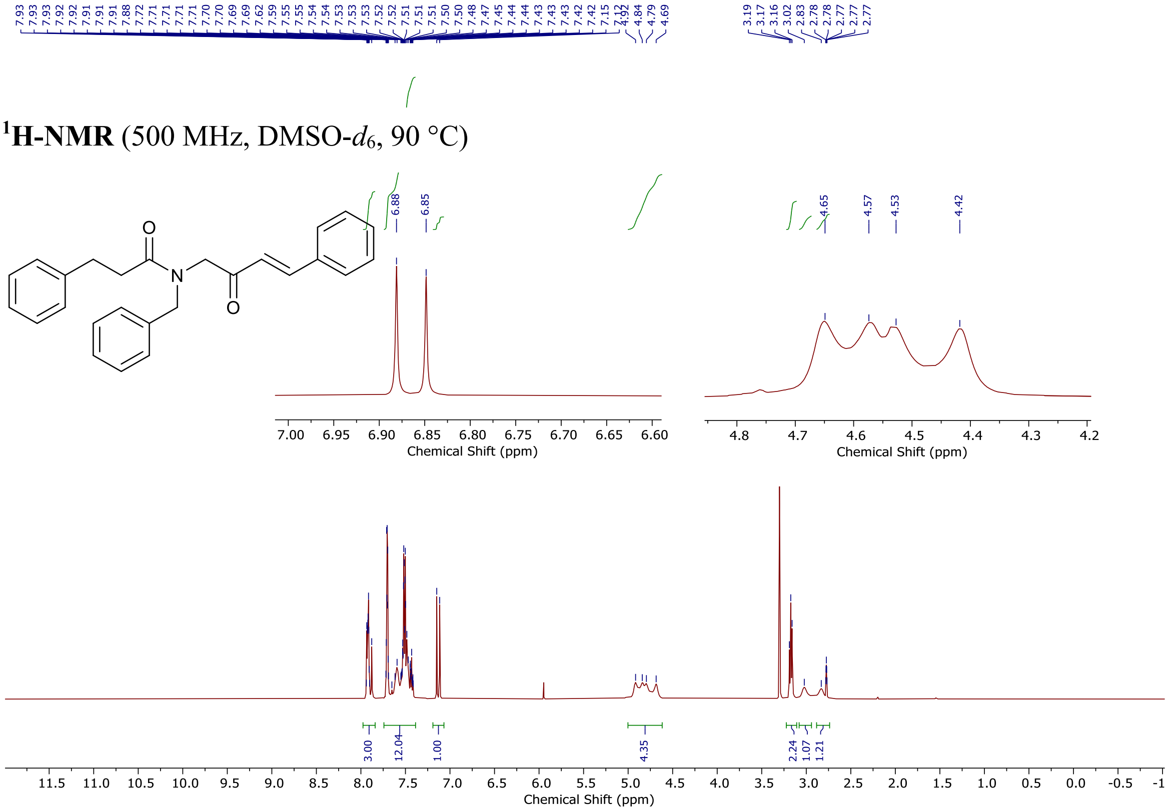

^{1}H-NMR (500 MHz, DMSO-d_6, 90 °C)

(*E*)-*N*-benzyl-*N*-(2-oxo-4-phenylbut-3-en-1-yl)-3-phenylpropanamide, 2g

^{1}H-NMR (400 MHz, $CDCl_3$)

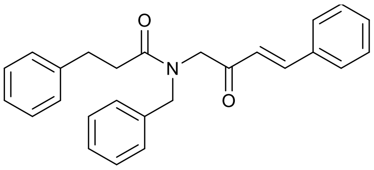

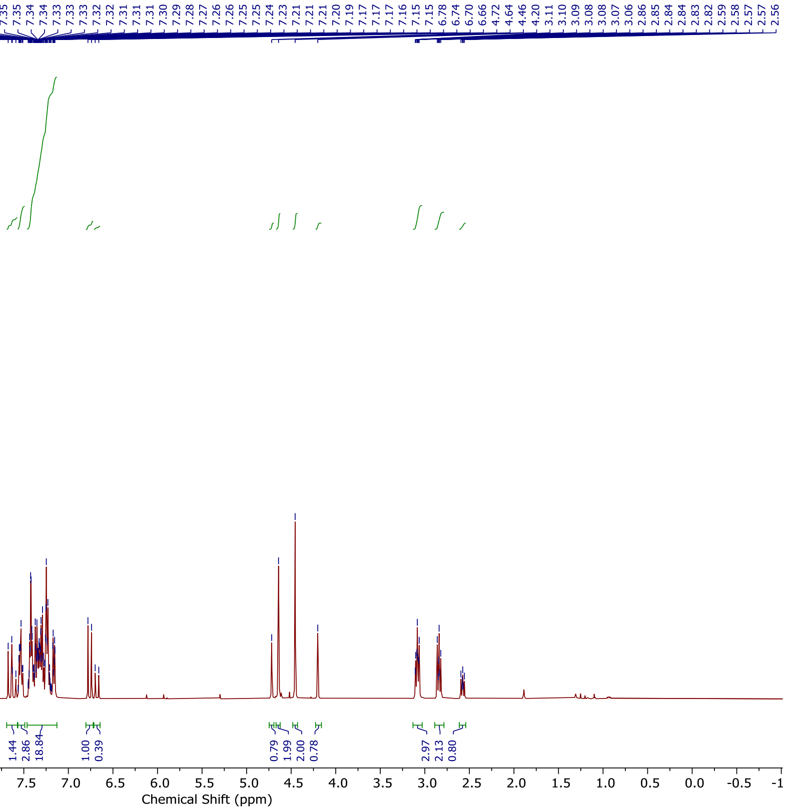

^{13}C-NMR (100 MHz, $CDCl_3$)

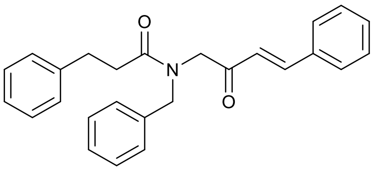

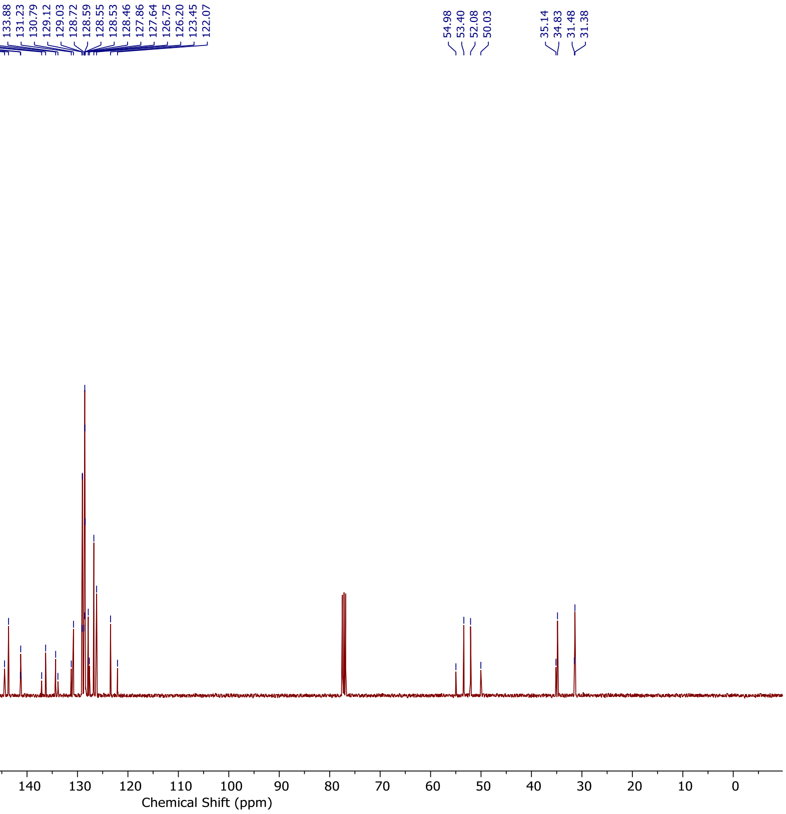

(*E*)-*N*-benzyl-*N*-(4-(4-bromophenyl)-2-oxobut-3-en-1-yl)-3-phenylpropanamide, 2h

^{1}H-NMR (400 MHz, $CDCl_3$)

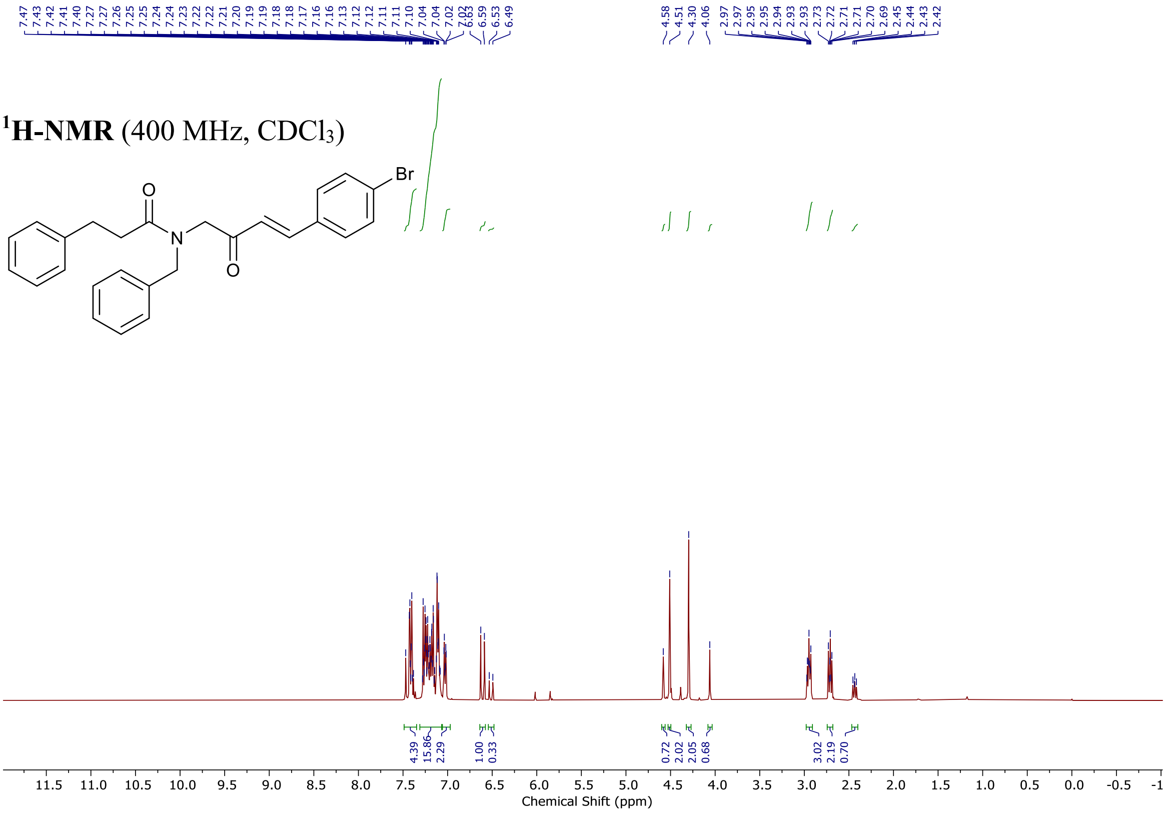

^{13}C-NMR (100 MHz, $CDCl_3$)

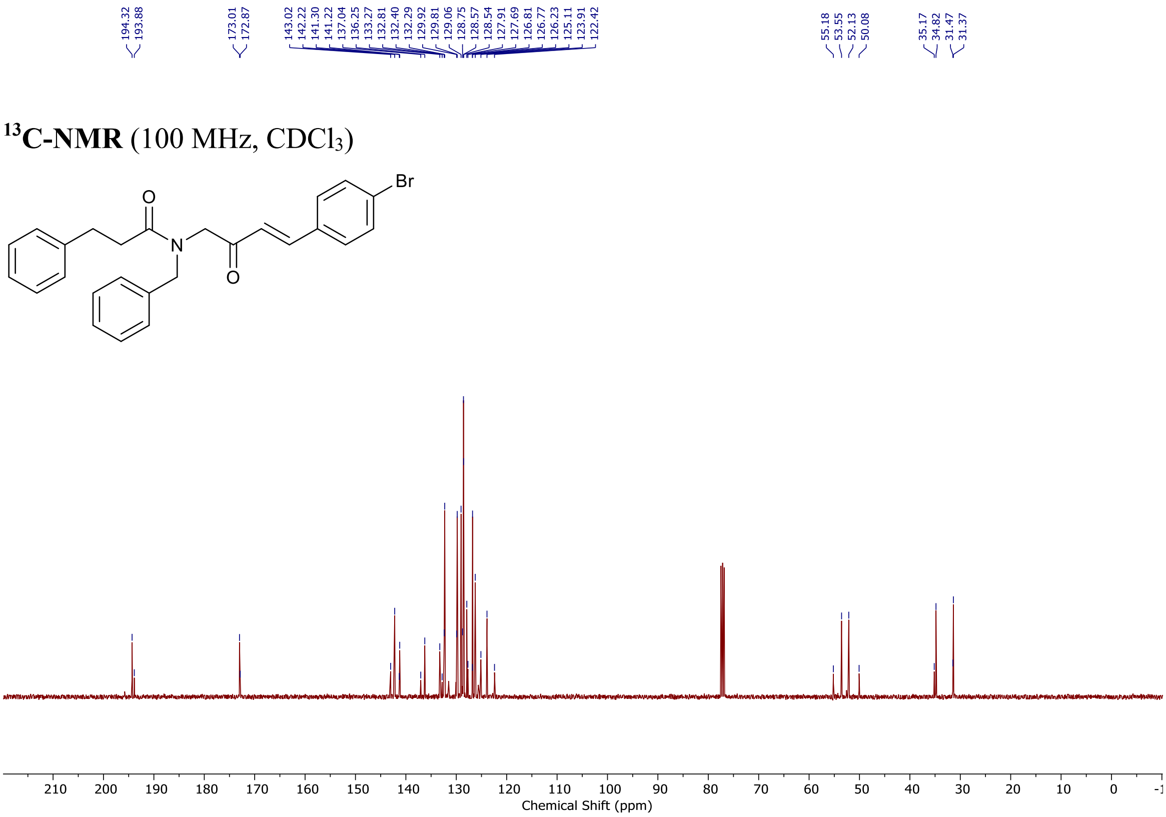

(*E*)-*N*-benzyl-*N*-(4-(4-methoxyphenyl)-2-oxobut-3-en-1-yl)-3-phenylpropanamide, 2i

^{1}H-NMR (400 MHz, $CDCl_3$)

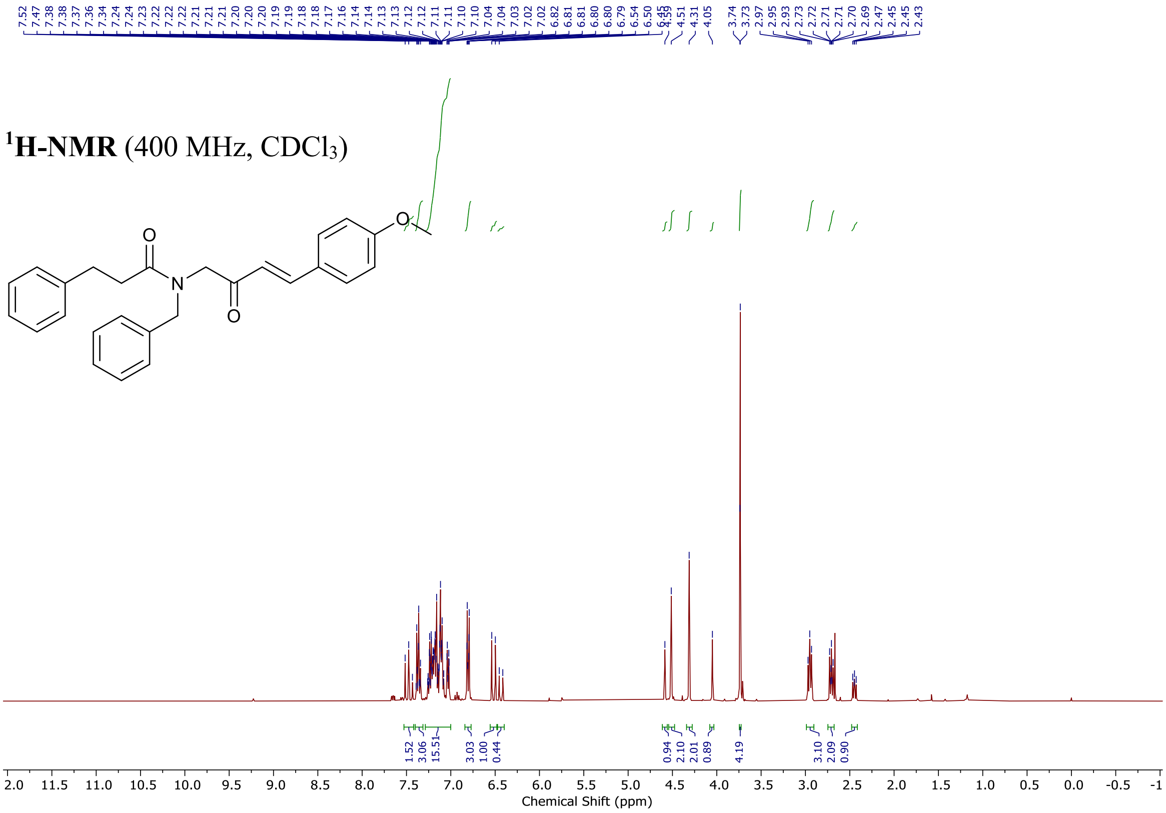

^{13}C-NMR (100 MHz, $CDCl_3$)

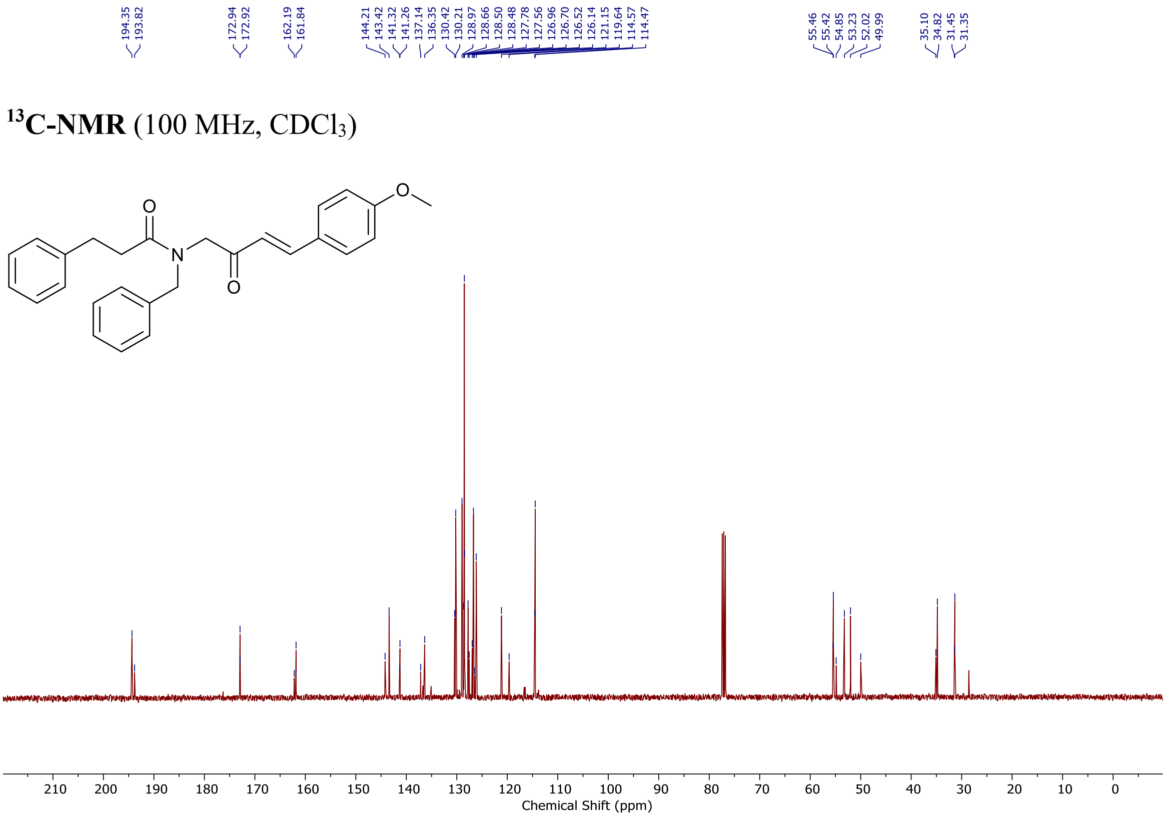

(*E*)-*N*-benzyl-*N*-(2-oxo-4-(thiophen-3-yl)but-3-en-1-yl)-3-phenylpropanamide, 2j

^{1}H-NMR (400 MHz, $CDCl_3$)

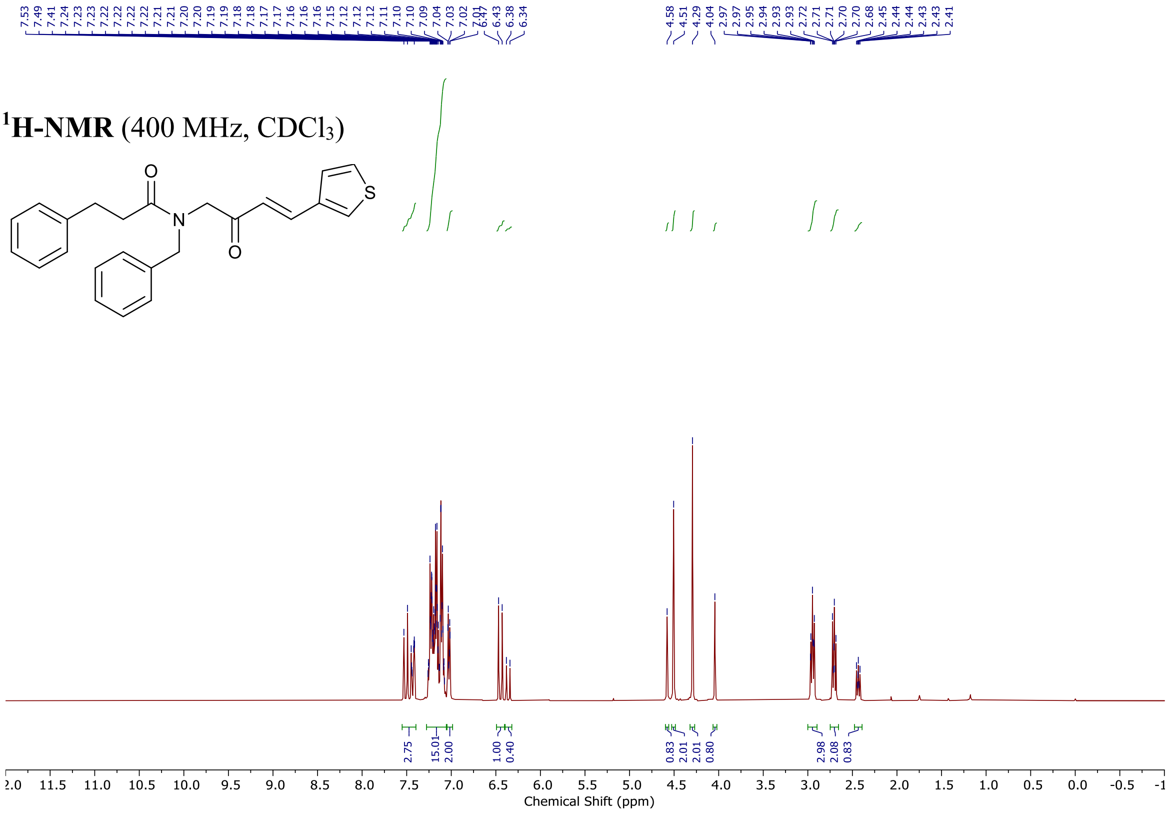

^{13}C-NMR (100 MHz, $CDCl_3$)

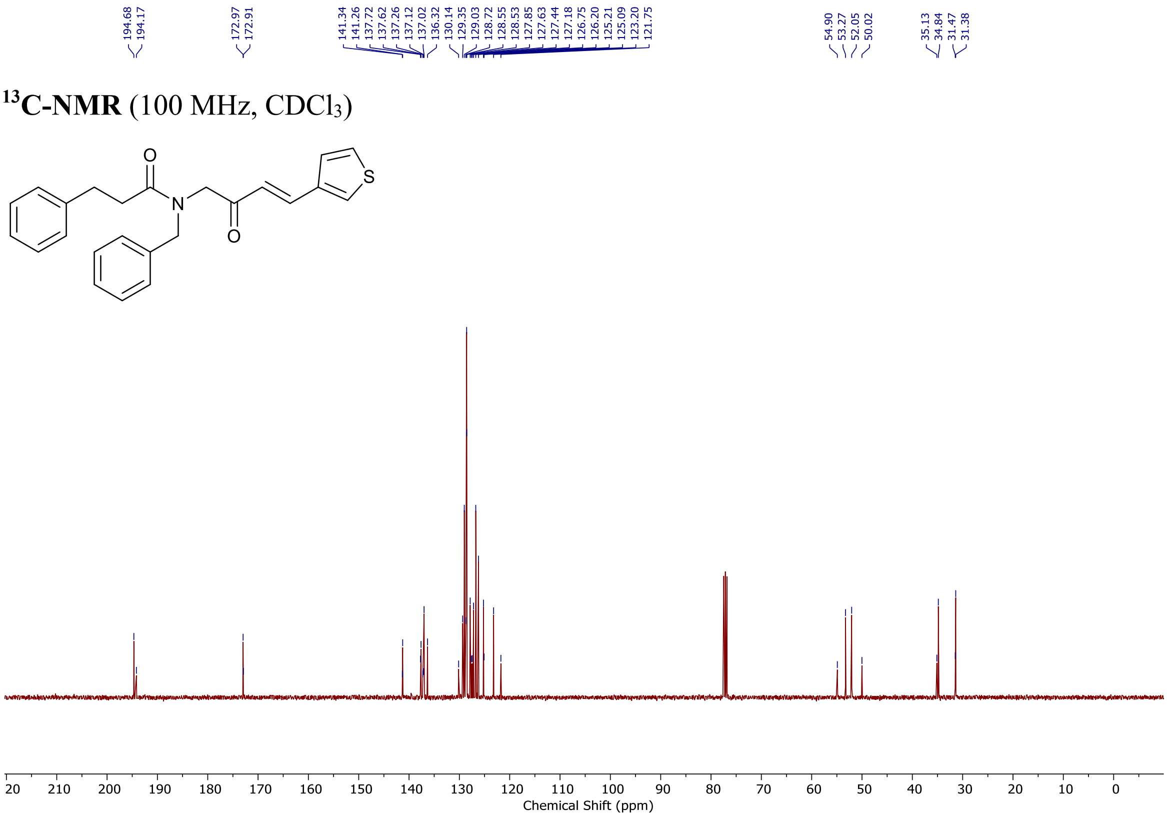

(*E*)-*N*-benzyl-*N*-(5,5-dimethyl-2-oxohex-3-en-1-yl)-3-phenylpropanamide, 2k

^{1}H-NMR (400 MHz, $CDCl_3$)

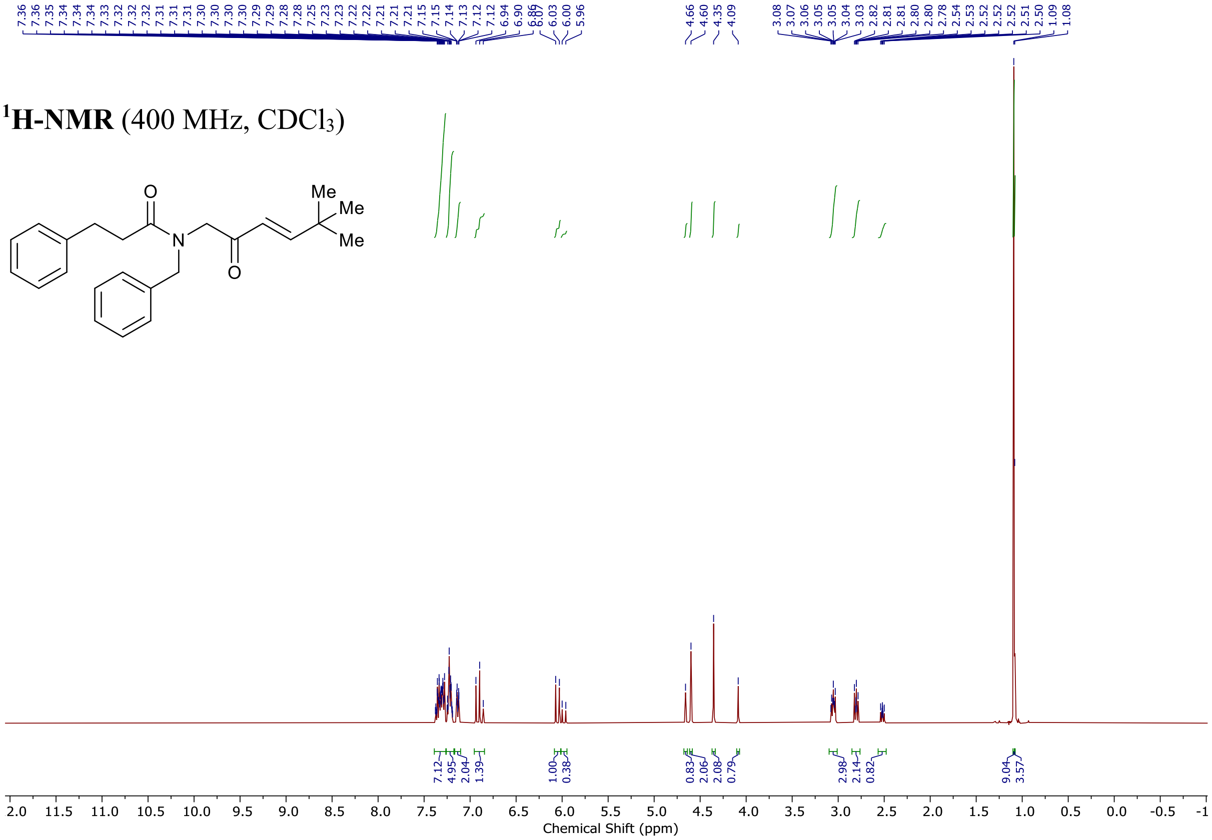

^{13}C-NMR (100 MHz, $CDCl_3$)

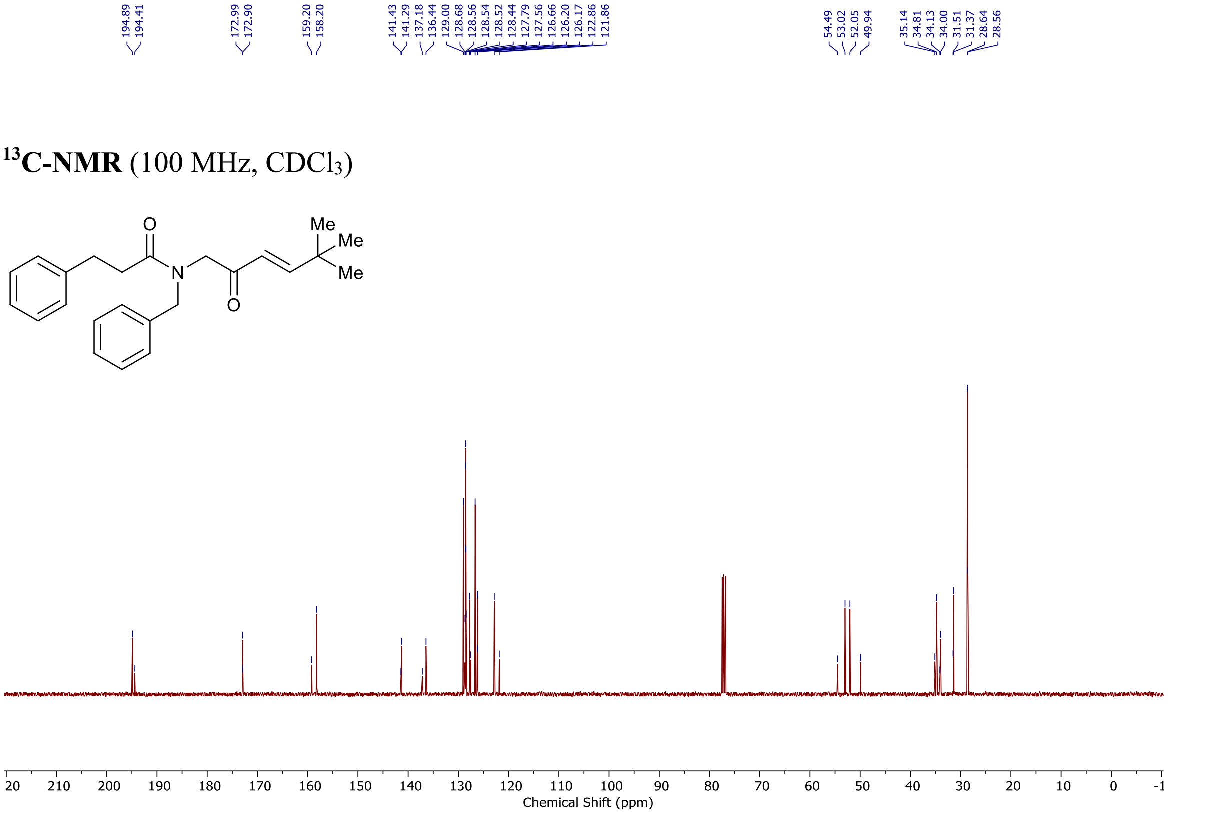

(*E*)-*N*-benzyl-*N*-(2-oxo-4-(trimethylsilyl)but-3-en-1-yl)-3-phenylpropanamide, 2l

^{1}H-NMR (400 MHz, $CDCl_3$)

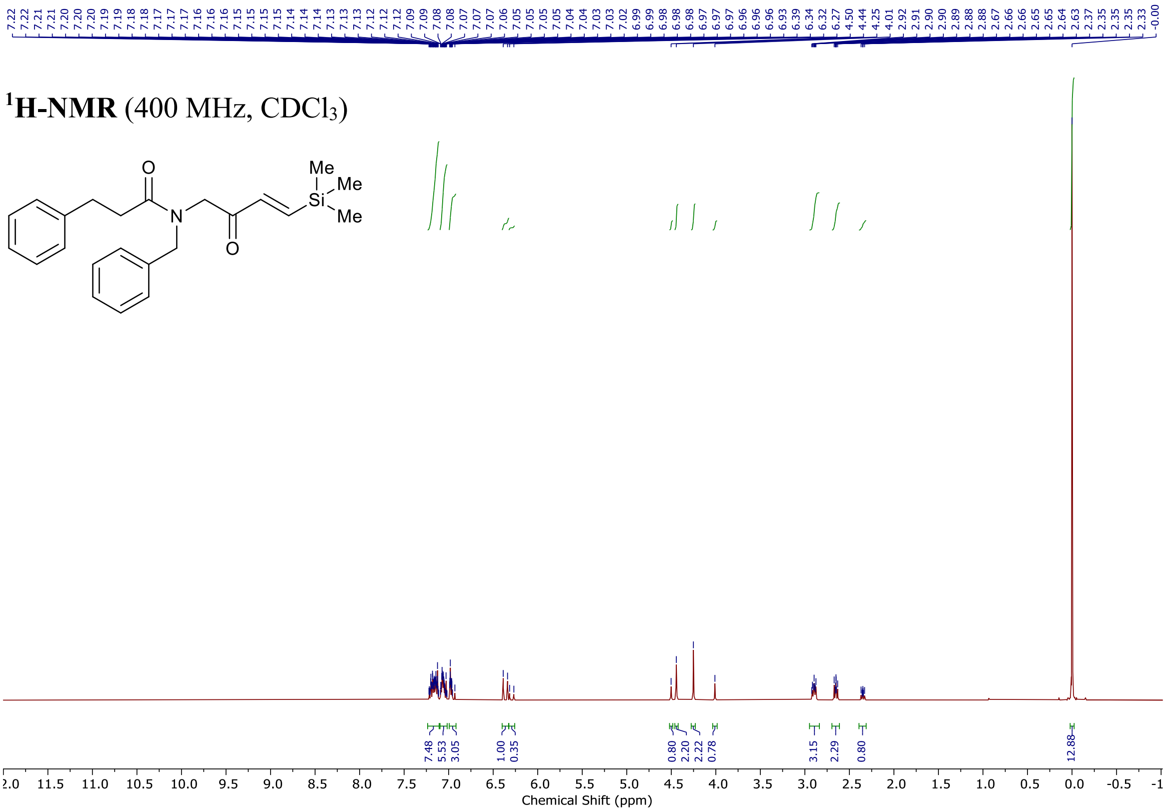

^{13}C-NMR (100 MHz, $CDCl_3$)

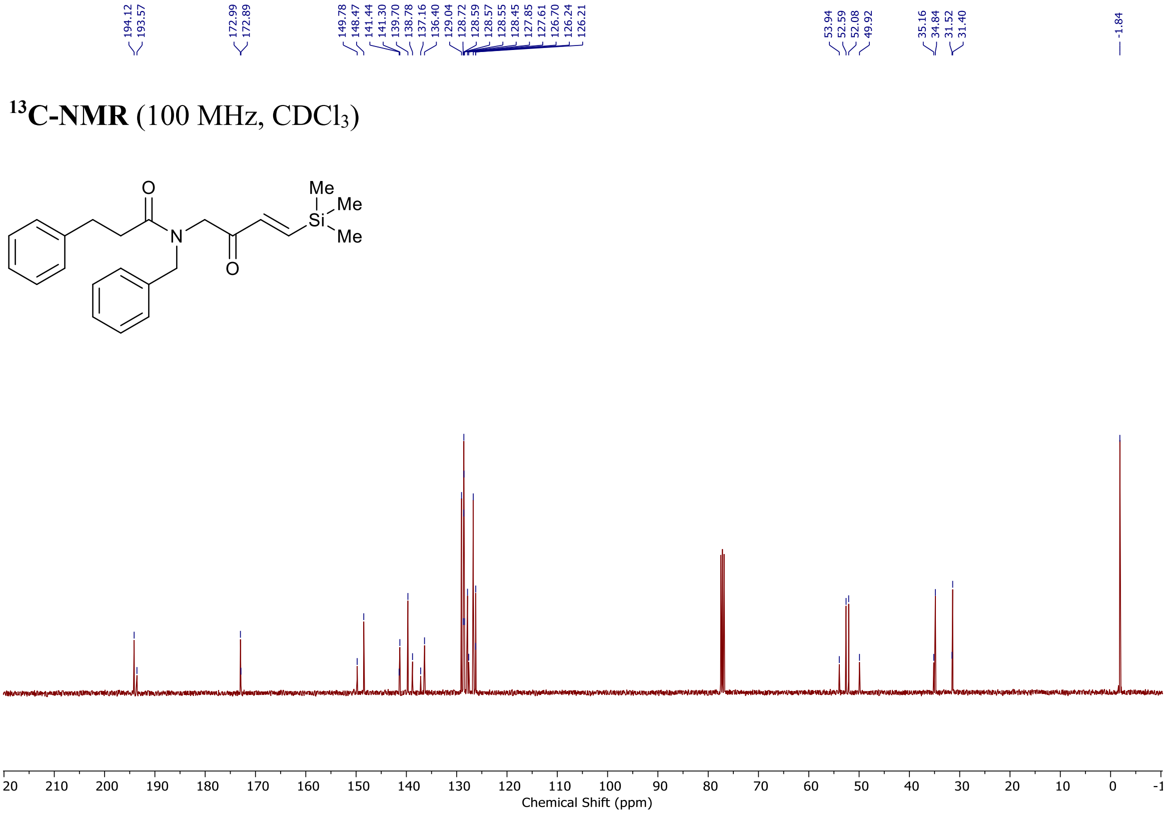

(*E*)-*N*-benzyl-*N*-(7-chloro-2-oxohept-3-en-1-yl)-3-phenylpropanamide, 2m

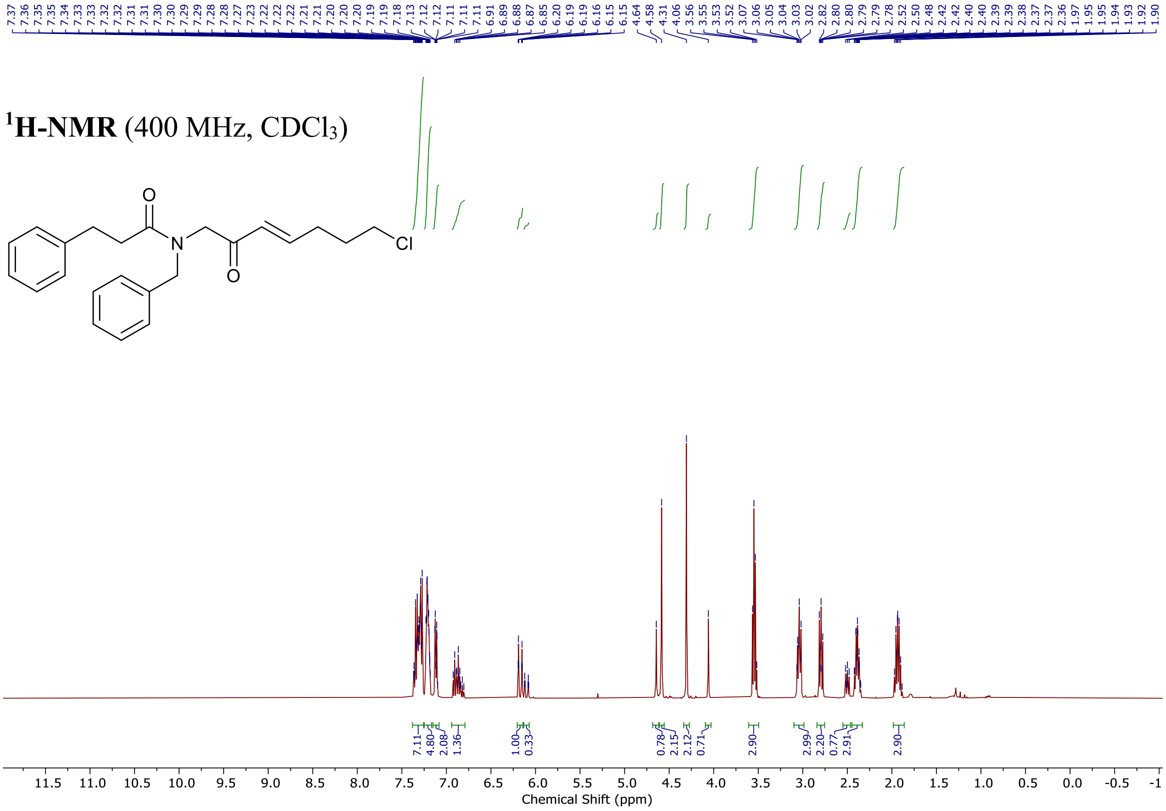

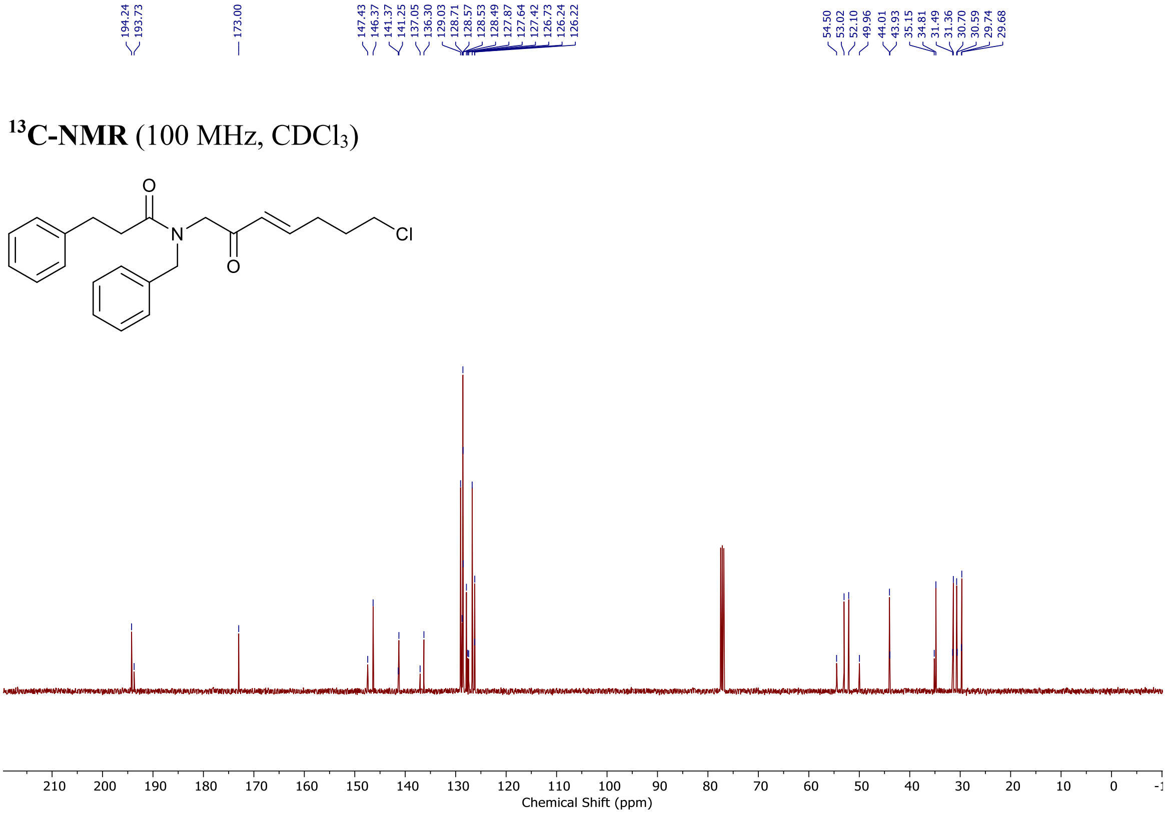

(*E*)-*N*-benzyl-*N*-(6-hydroxy-2-oxohept-3-en-1-yl)-3-phenylpropanamide, 2n

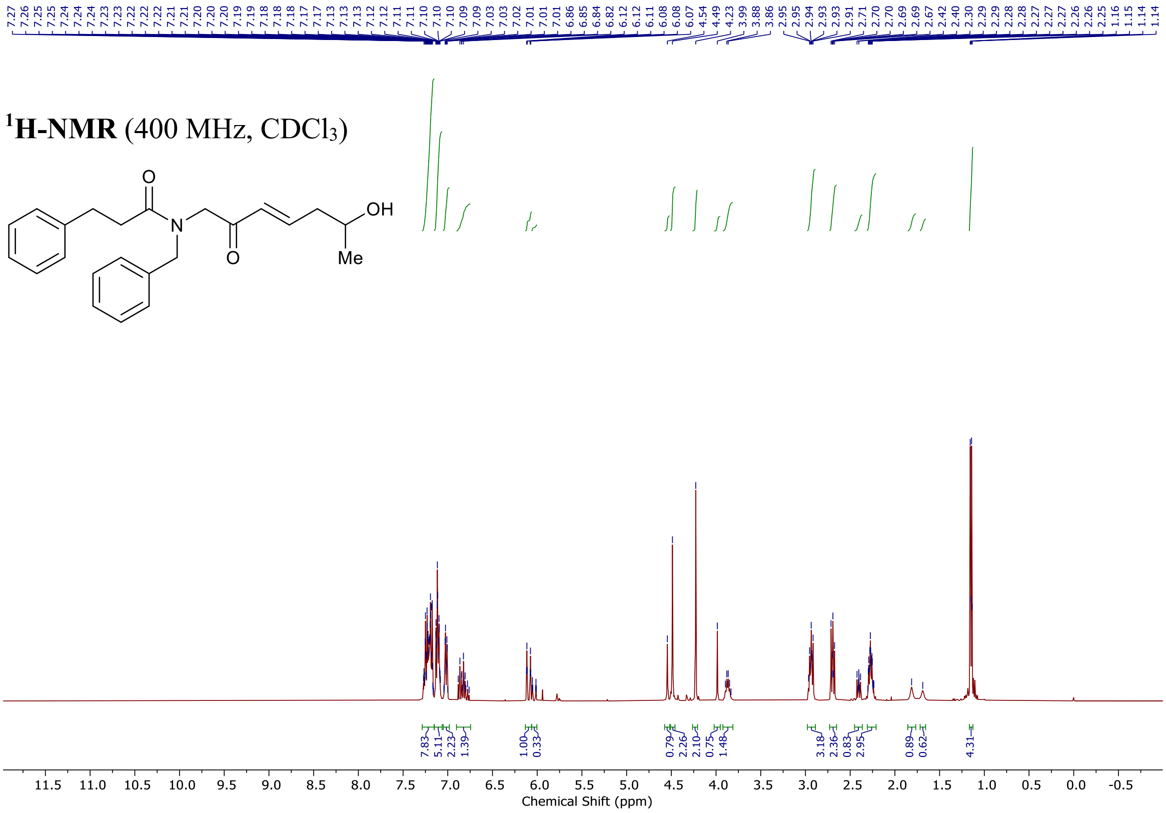

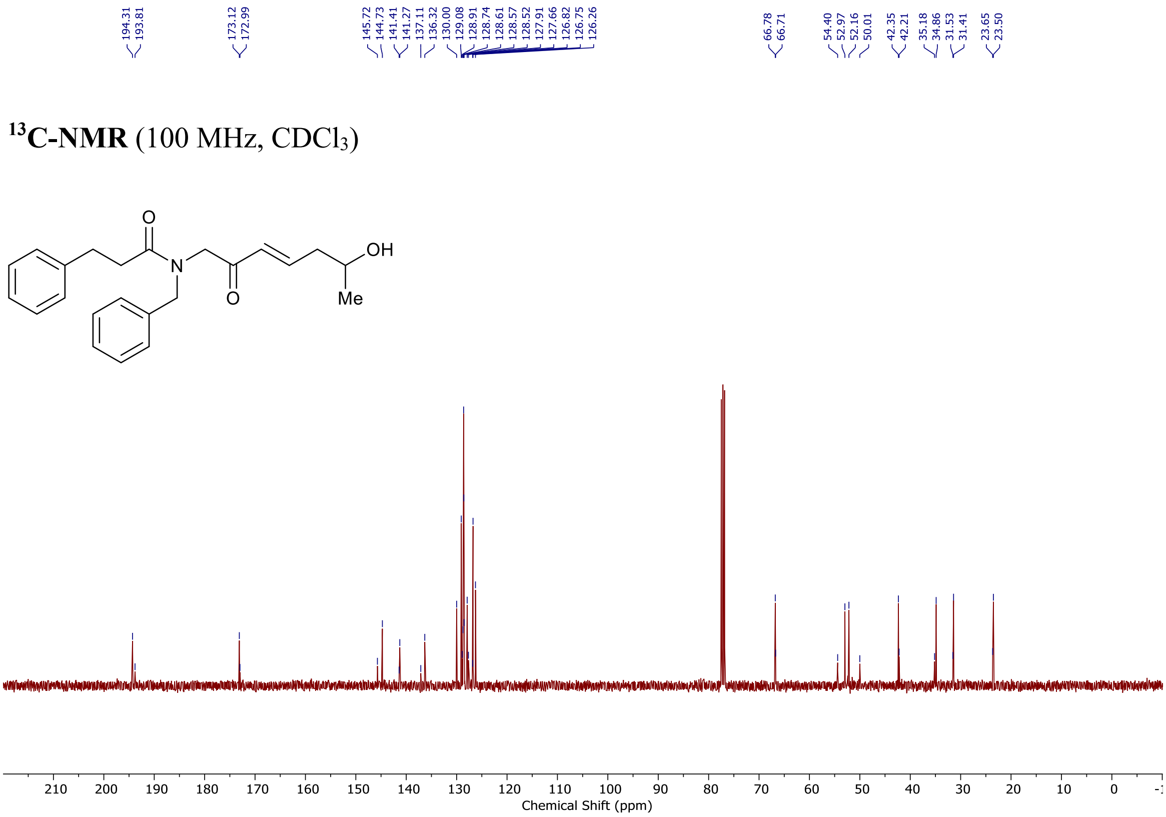

(*E*)-*N*-benzyl-*N*-(5,5-diethoxy-2-oxopent-3-en-1-yl)-3-phenylpropanamide, 2o

^{1}H-NMR (400 MHz, $CDCl_3$)

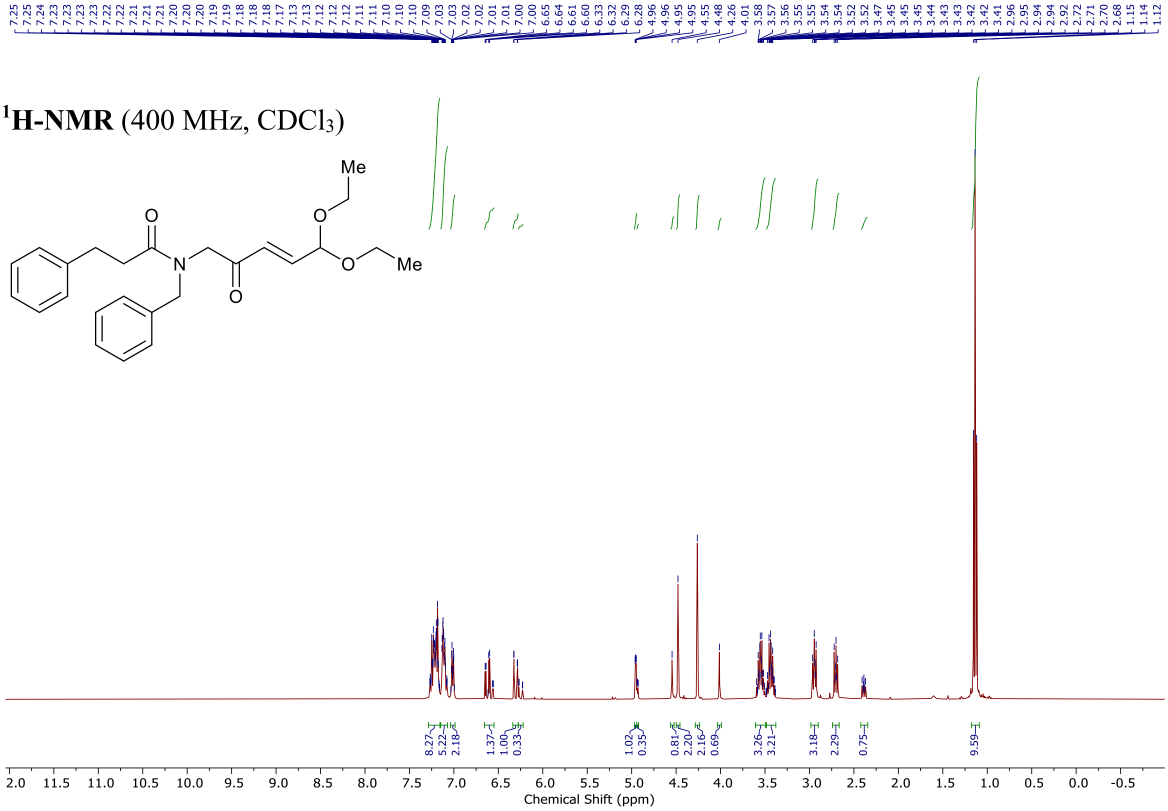

^{13}C-NMR (100 MHz, $CDCl_3$)

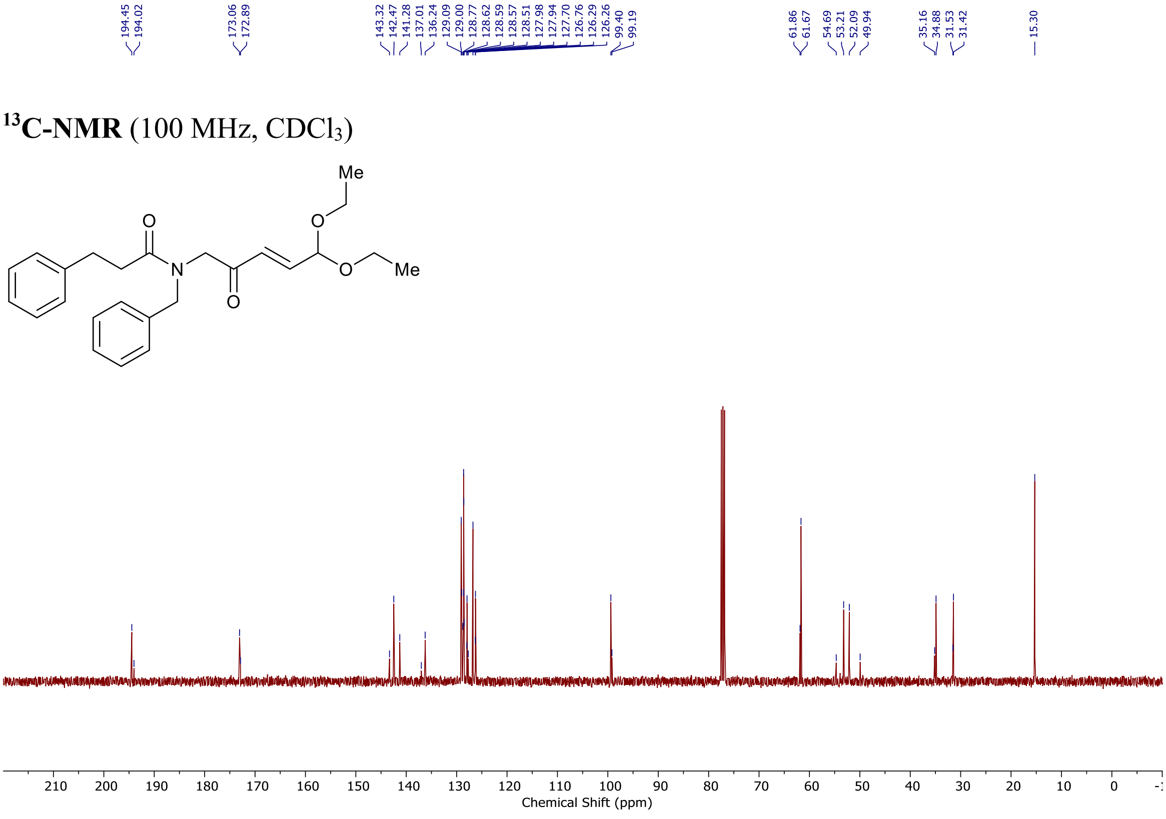

(*E*)-*N*-benzyl-*N*-(8-(1,3-dioxoisoindolin-2-yl)-2-oxooct-3-en-1-yl)-3-phenylpropanamide, 2p

^{1}H-NMR (400 MHz, $CDCl_3$)

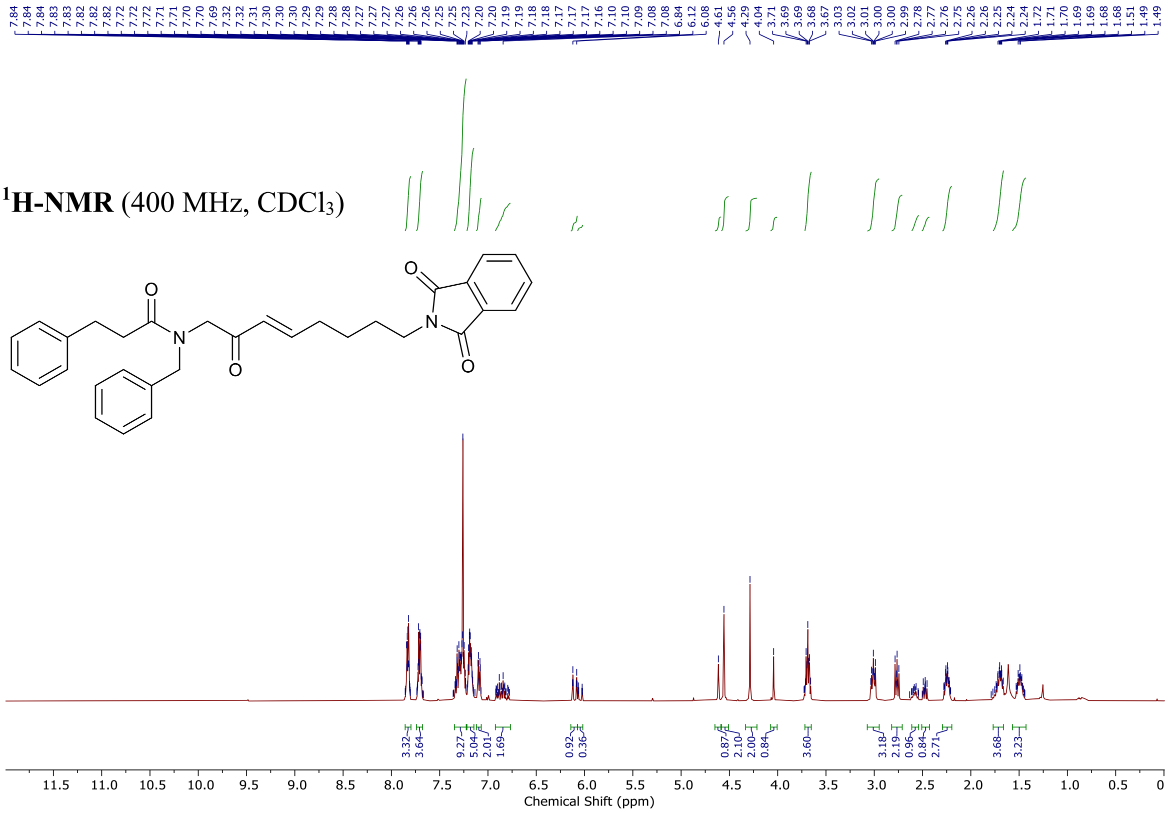

^{13}C-NMR (100 MHz, $CDCl_3$)

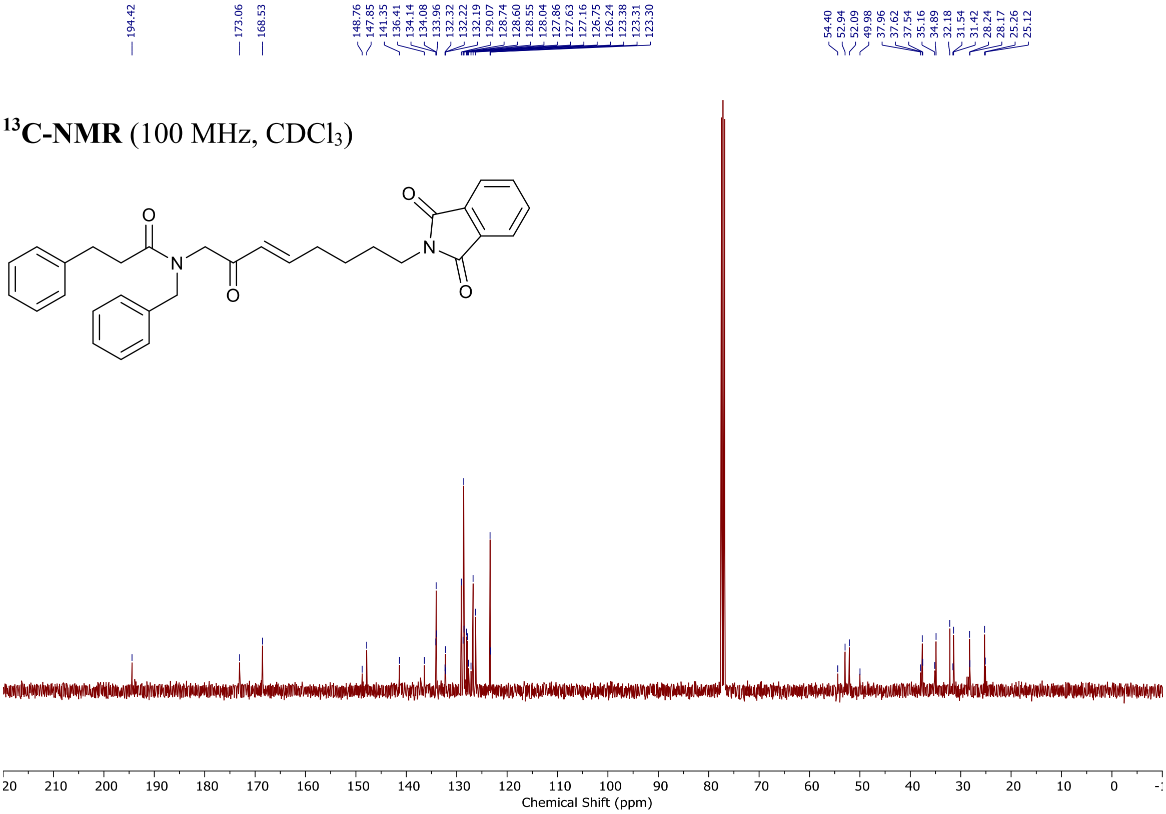

(*E*)-*N*-benzyl-*N*-(2-oxo-3,4-diphenylbut-3-en-1-yl)-3-phenylpropanamide, 2q

^{1}H-NMR (400 MHz, $CDCl_3$)

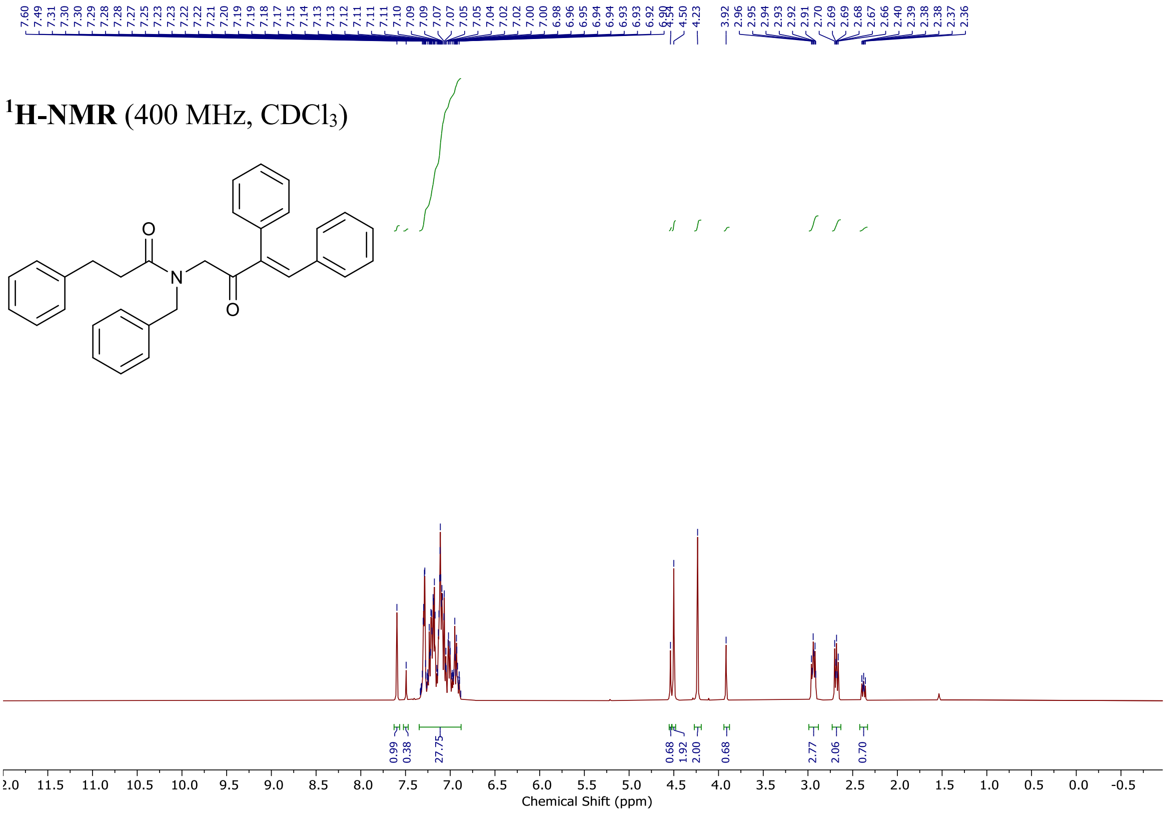

^{13}C-NMR (100 MHz, $CDCl_3$)

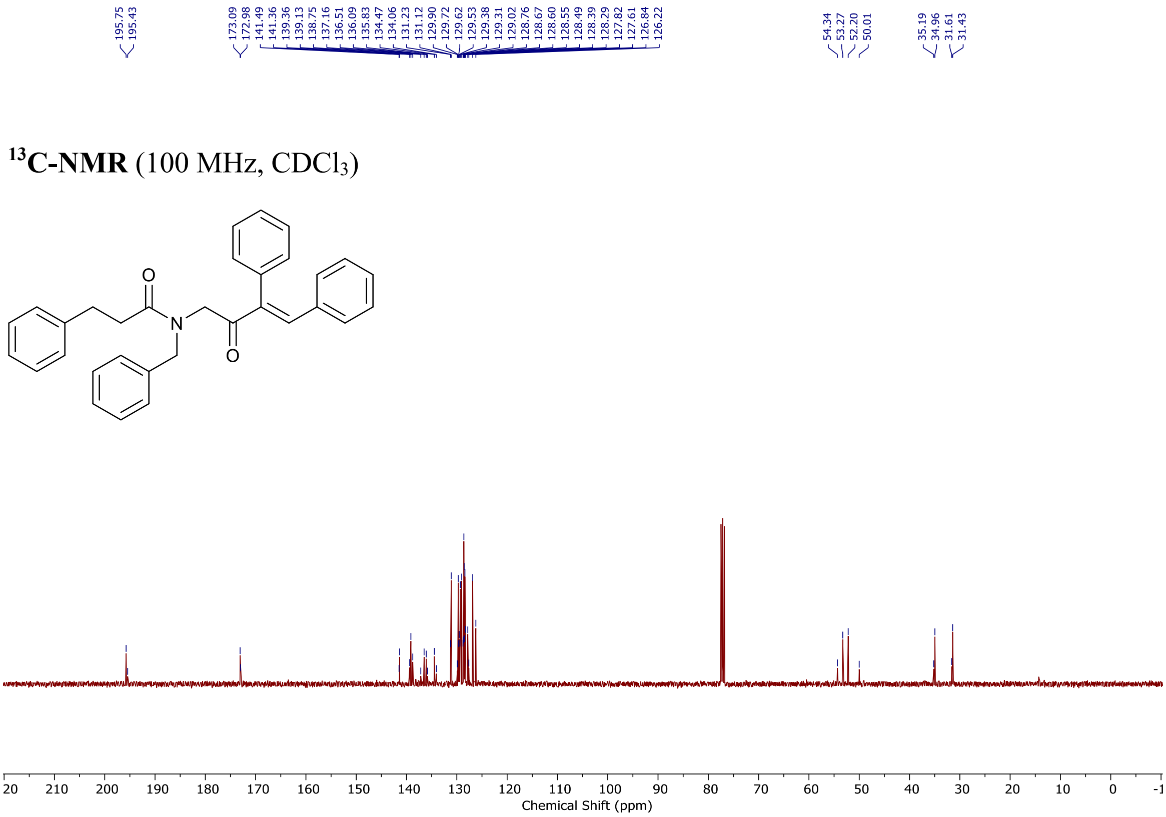

(*E*)-*N*-benzyl-*N*-(2-oxo-3-propylhept-3-en-1-yl)-3-phenylpropanamide, 2r

^{1}H-NMR (400 MHz, $CDCl_3$)

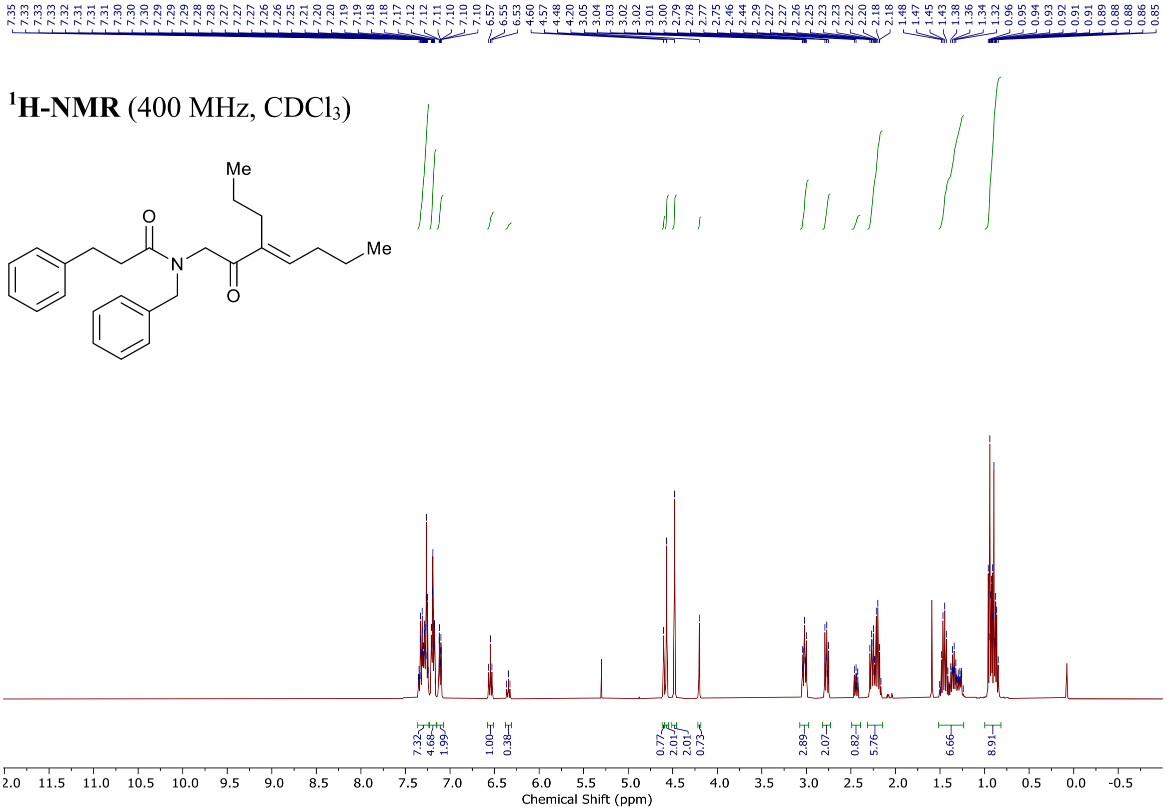

^{13}C-NMR (100 MHz, $CDCl_3$)

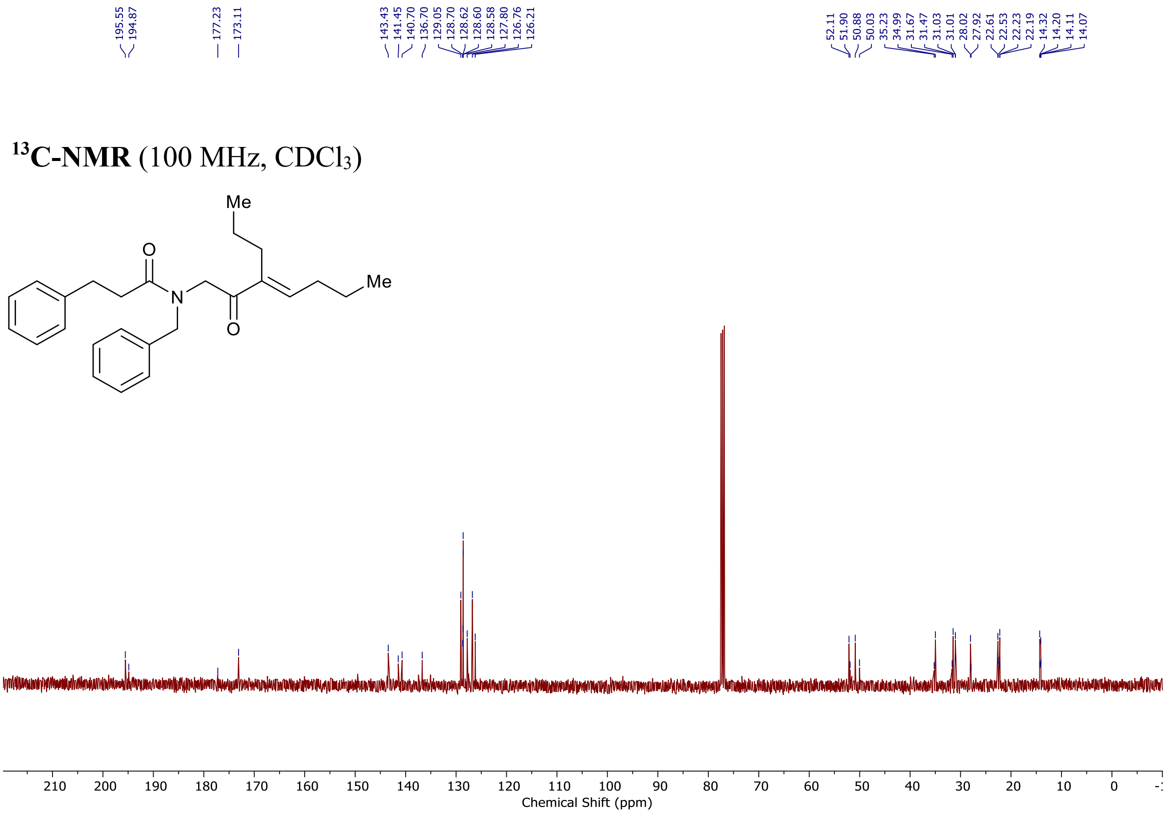

(*E*)-*N*-methyl-*N*-(2-oxo-4-phenylbut-3-en-1-yl)-3-phenylpropanamide, 2s

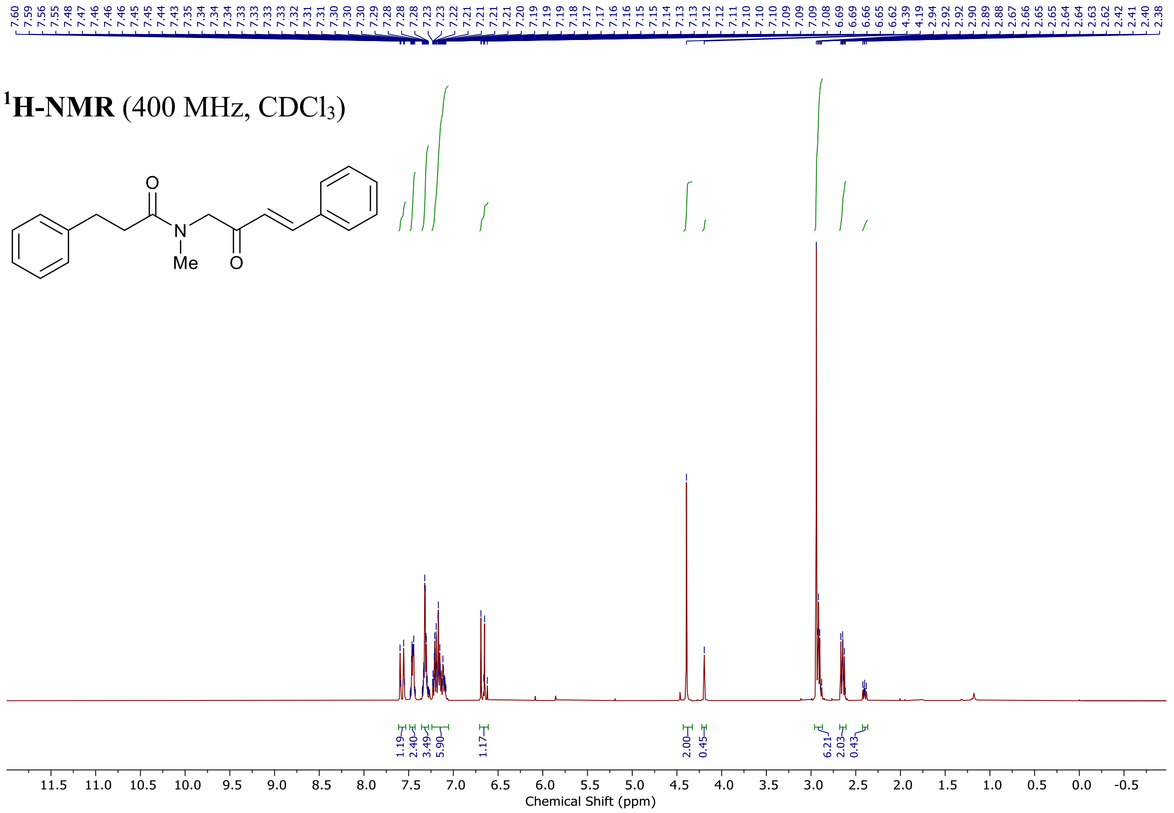

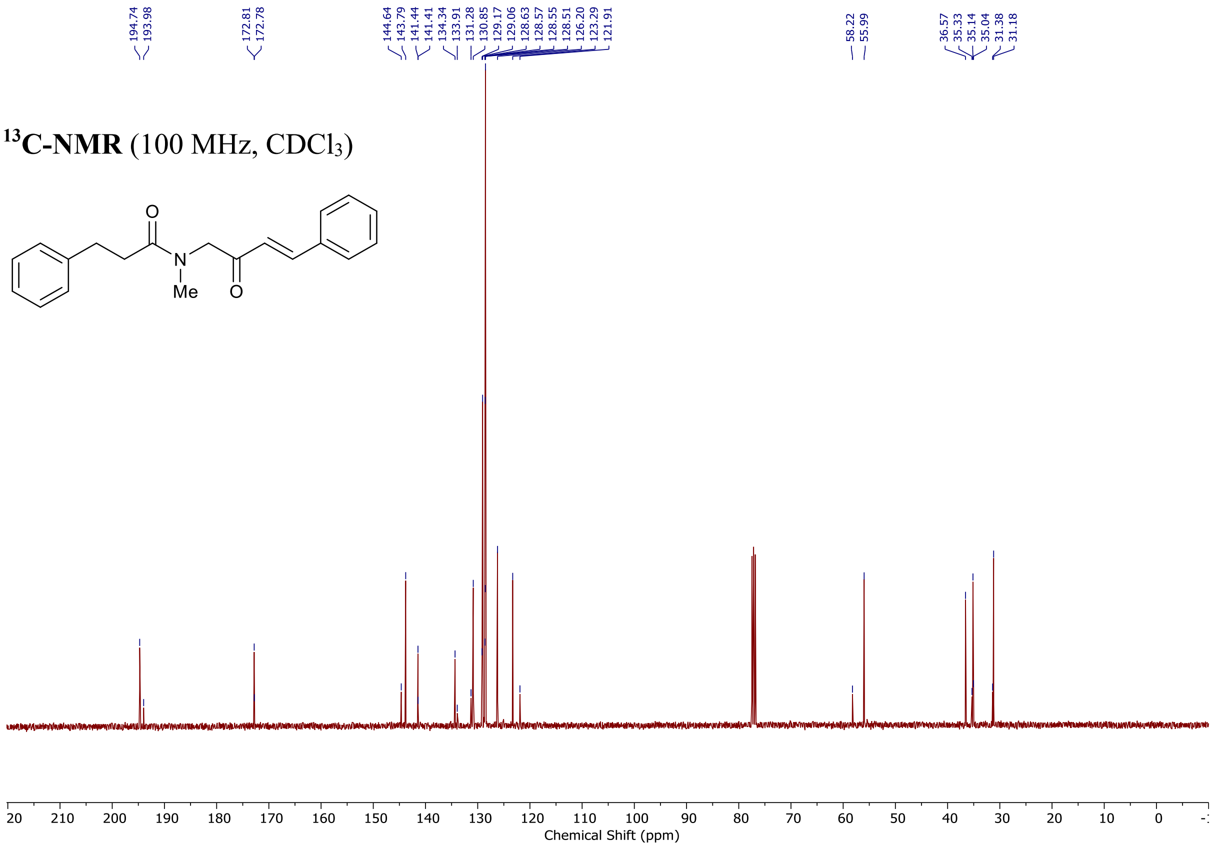

(*E*)-*N*-(2-oxo-4-phenylbut-3-en-1-yl)-*N*,3-diphenylpropanamide, 2t

^{1}H-NMR (400 MHz, $CDCl_3$)

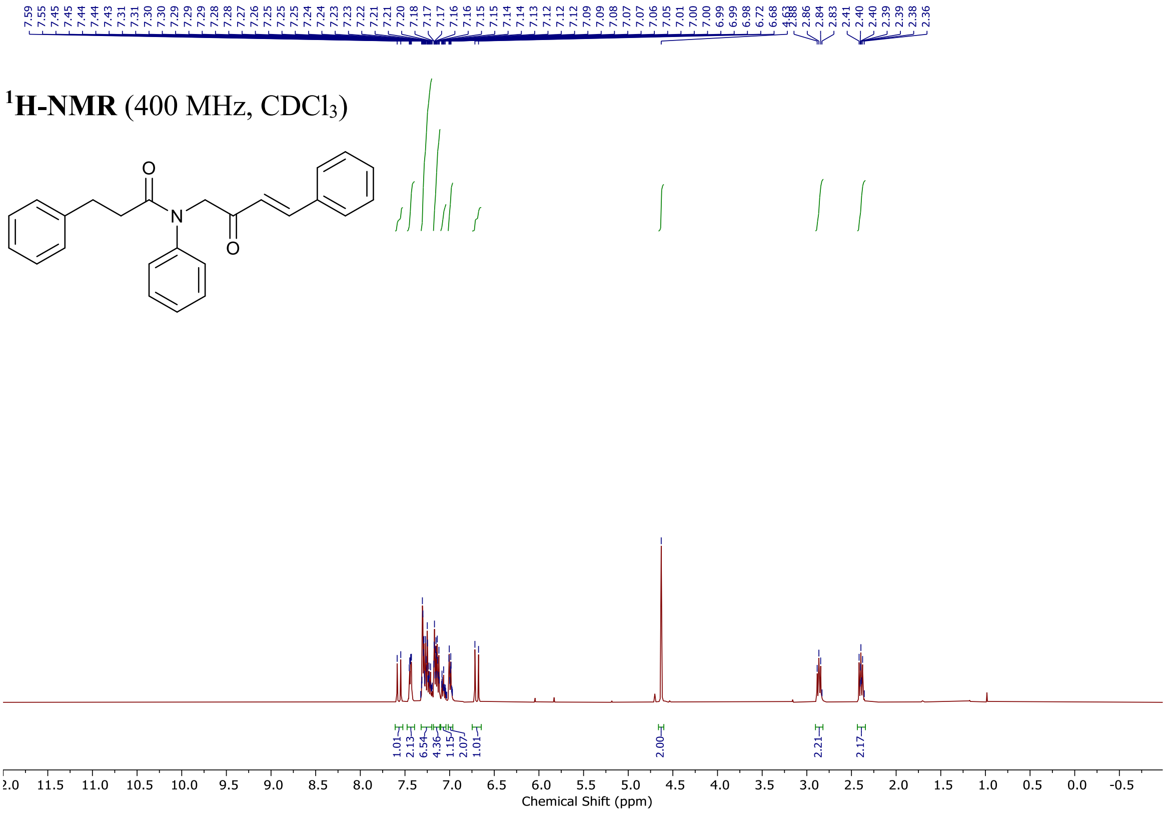

^{13}C-NMR (100 MHz, $CDCl_3$)

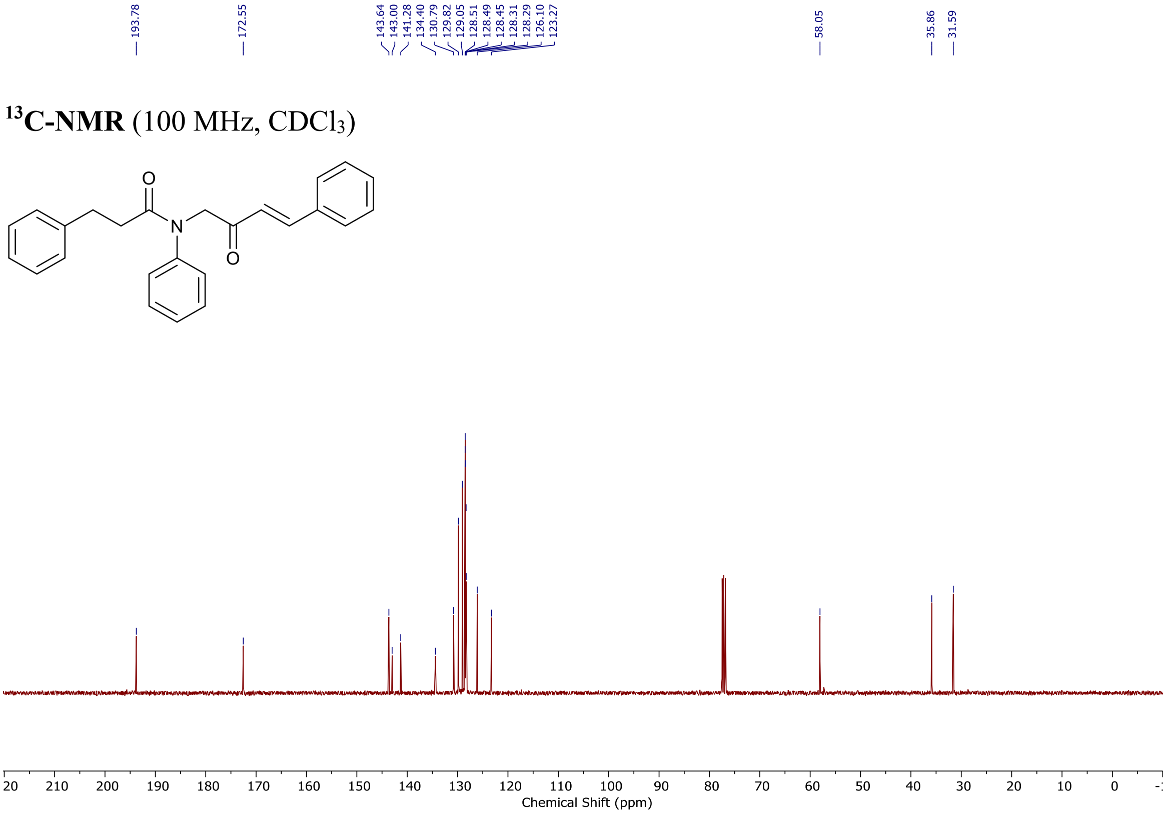

(*E*)-3-phenyl-1-(1-(3-phenylpropanoyl)pyrrolidin-2-yl)prop-2-en-1-one, 2u

^{1}H-NMR (400 MHz, CDCl$_3$)

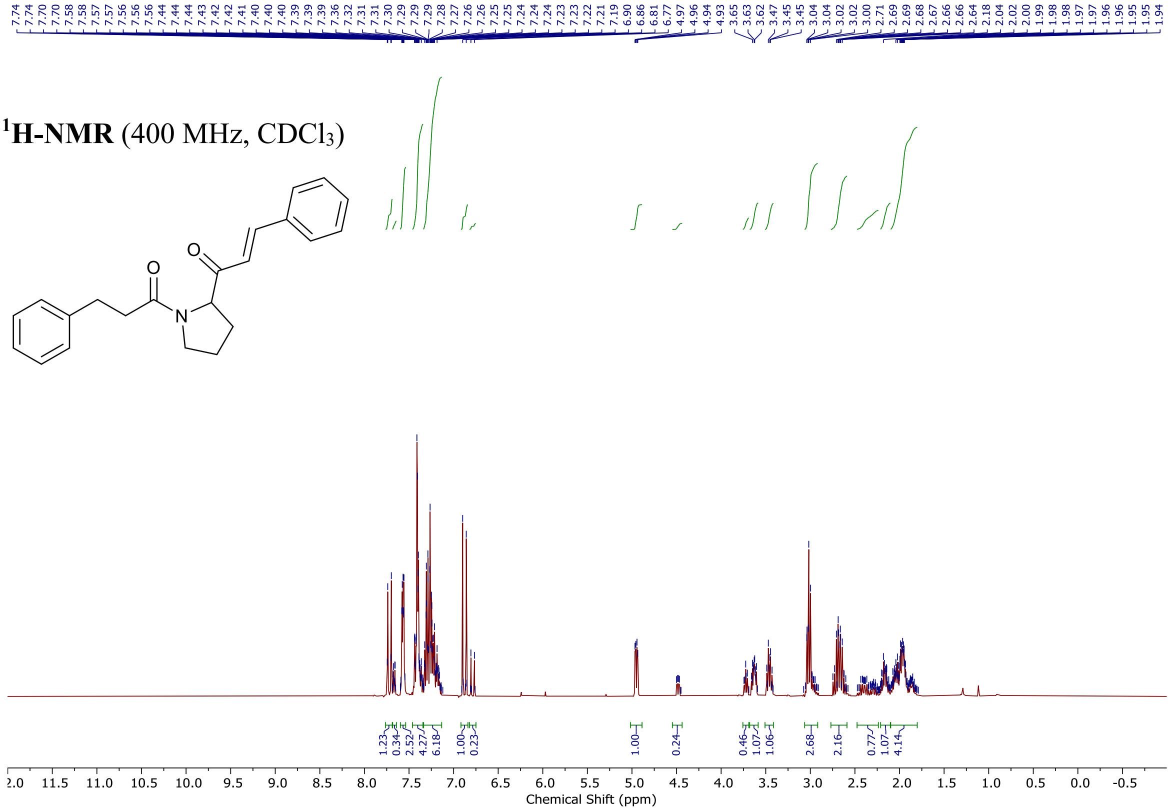

^{13}C-NMR (100 MHz, CDCl$_3$)

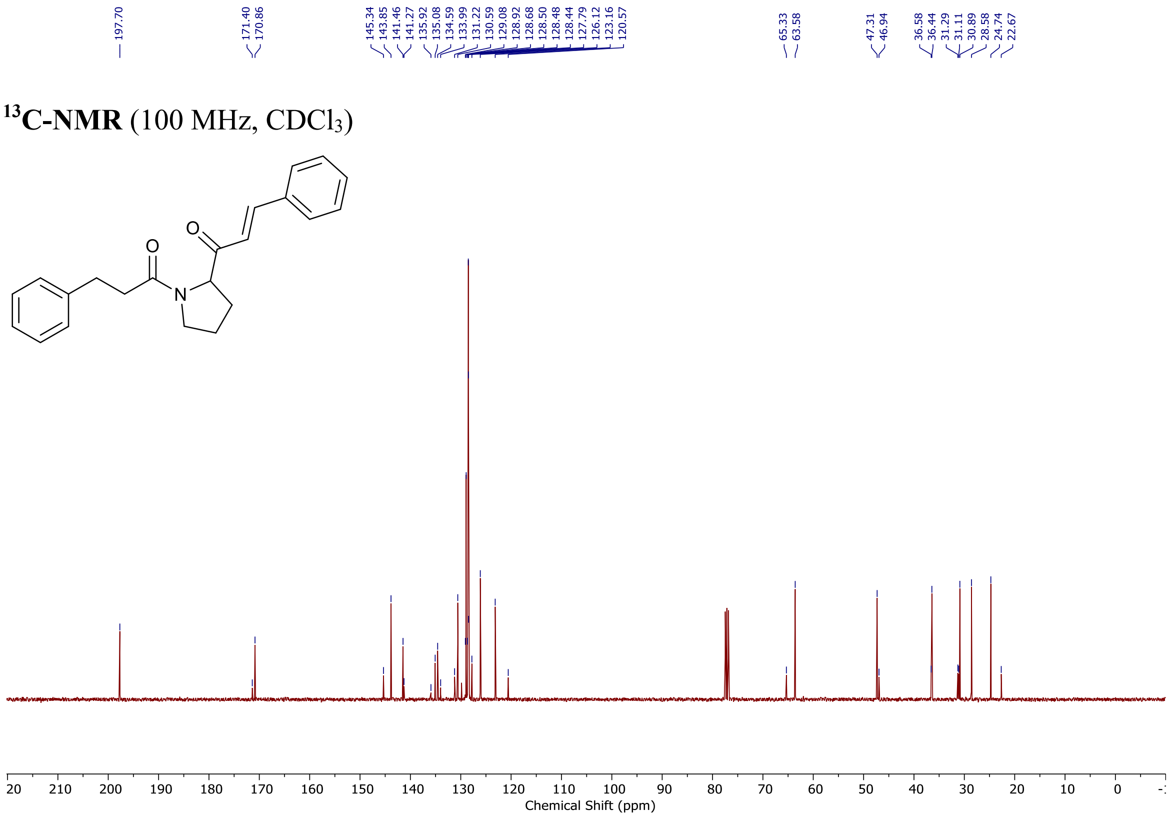

(*E*)-*N*-(2-oxo-4-phenylbut-3-en-1-yl)-3-phenylpropanamide, 2v

^{1}H-NMR (400 MHz, CDCl$_3$)

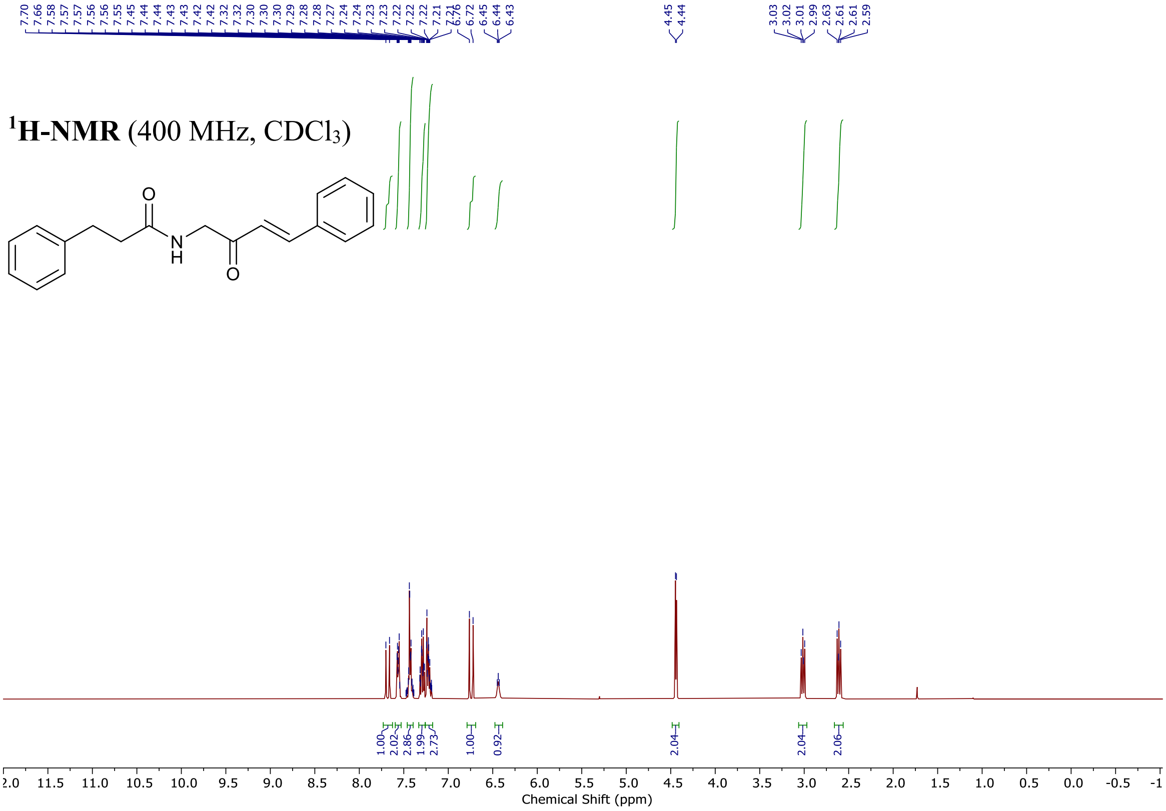

^{13}C-NMR (100 MHz, CDCl$_3$)

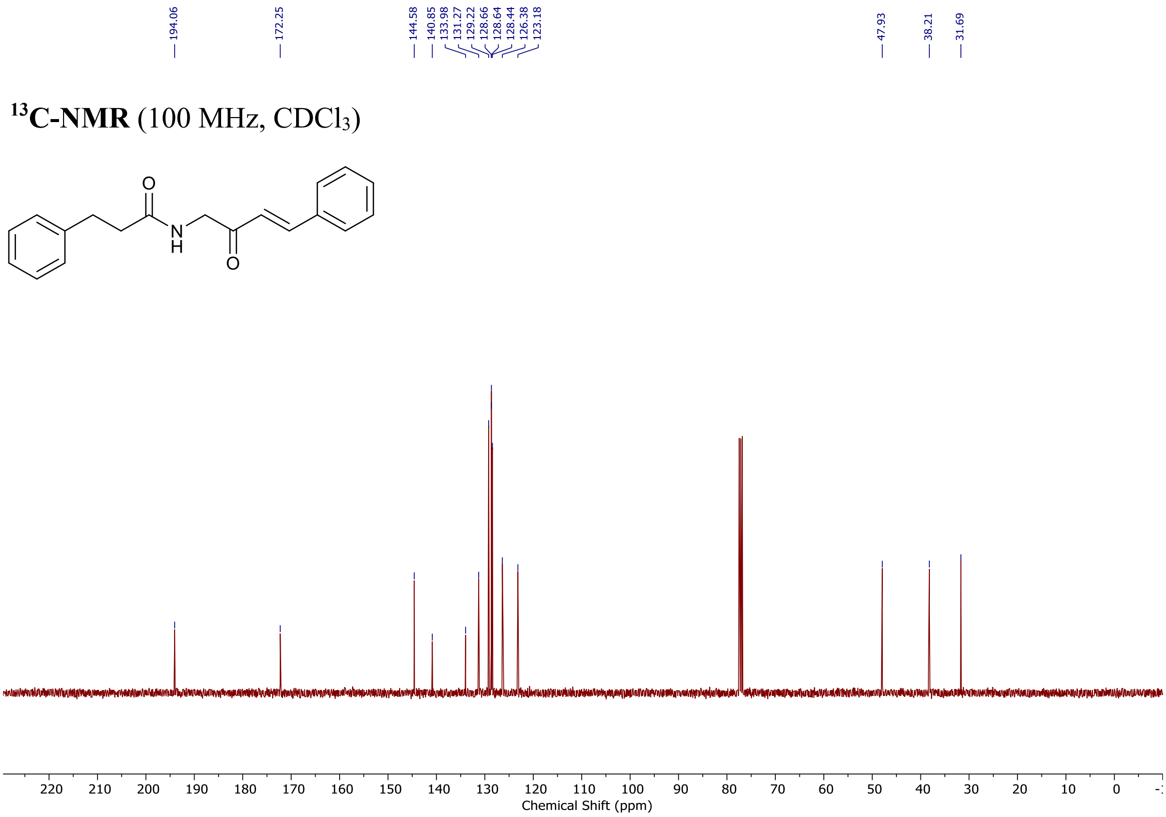

(*E*)-*N*-(3-oxo-1,5-diphenylpent-4-en-2-yl)-3-phenylpropanamide, 2w

^{1}H-NMR (400 MHz, $CDCl_3$)

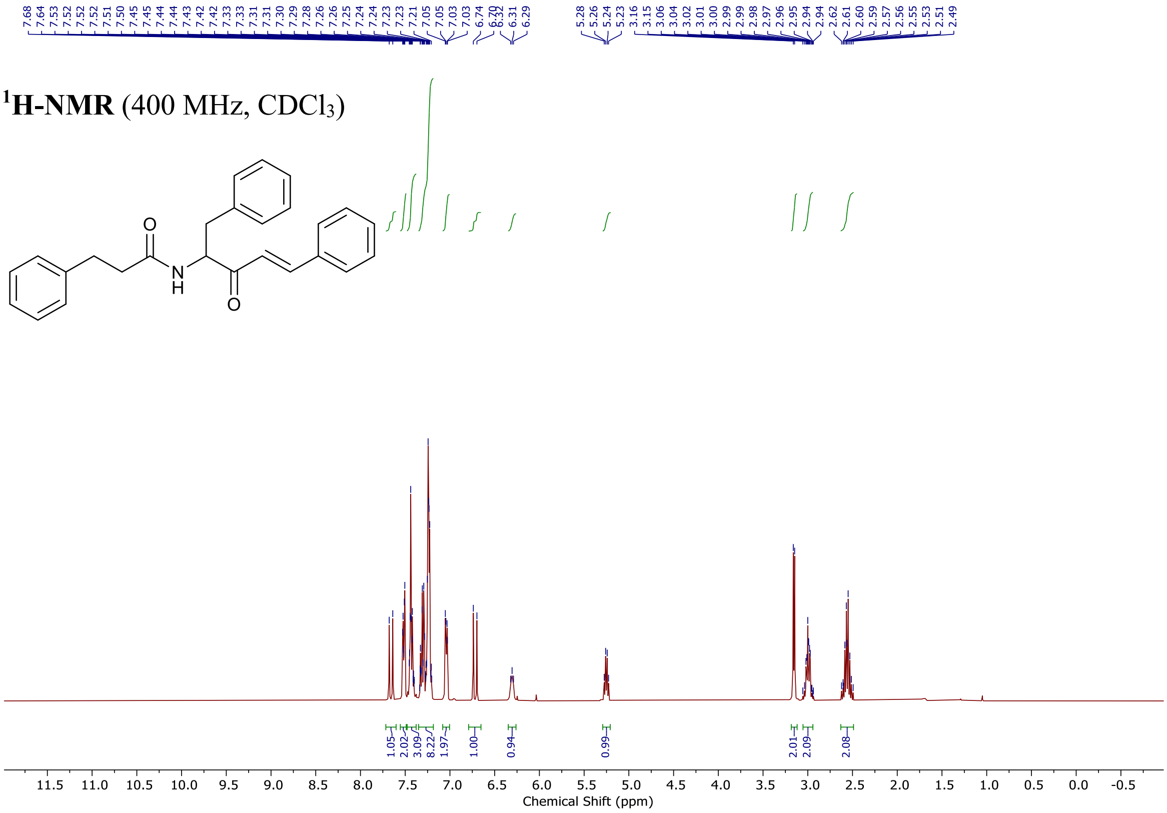

^{13}C-NMR (100 MHz, $CDCl_3$)

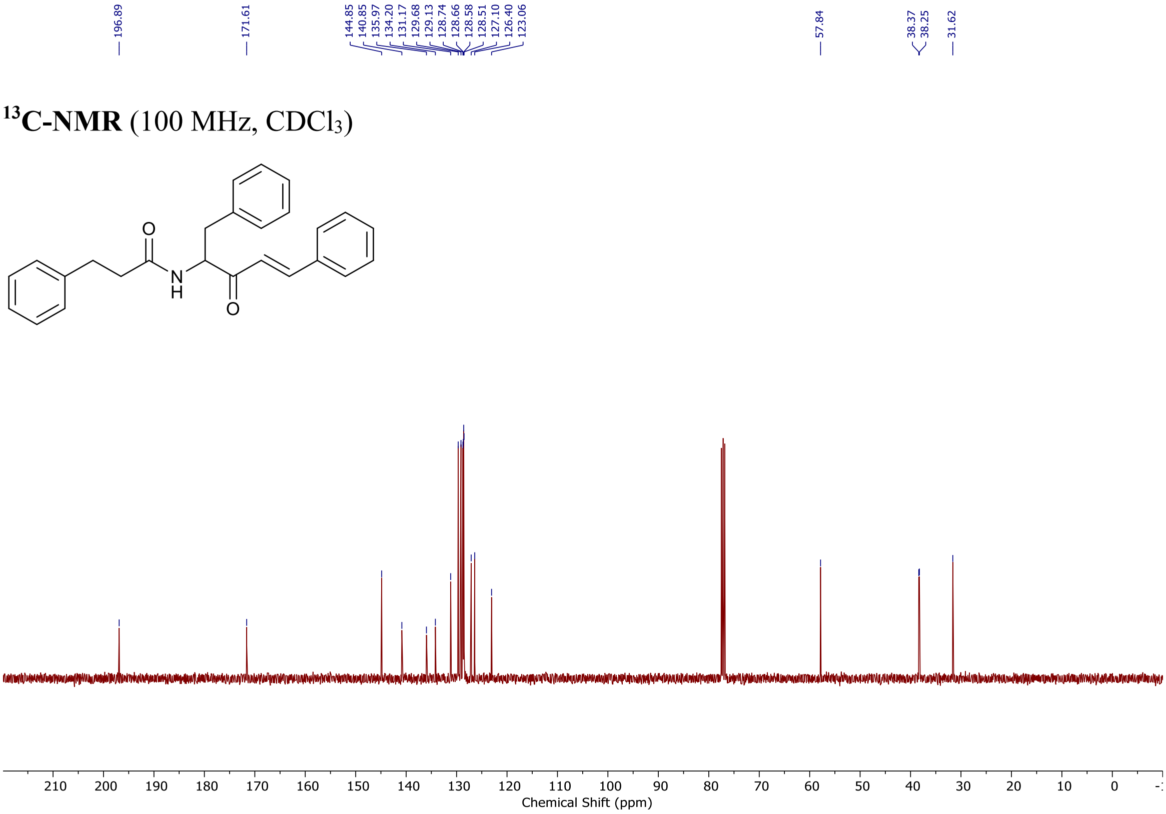

(*E*)-*N*-(3-oxo-5-phenylpent-4-en-2-yl)-3-phenylpropanamide, 2x

^{1}H-NMR (400 MHz, $CDCl_3$)

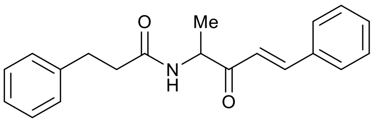

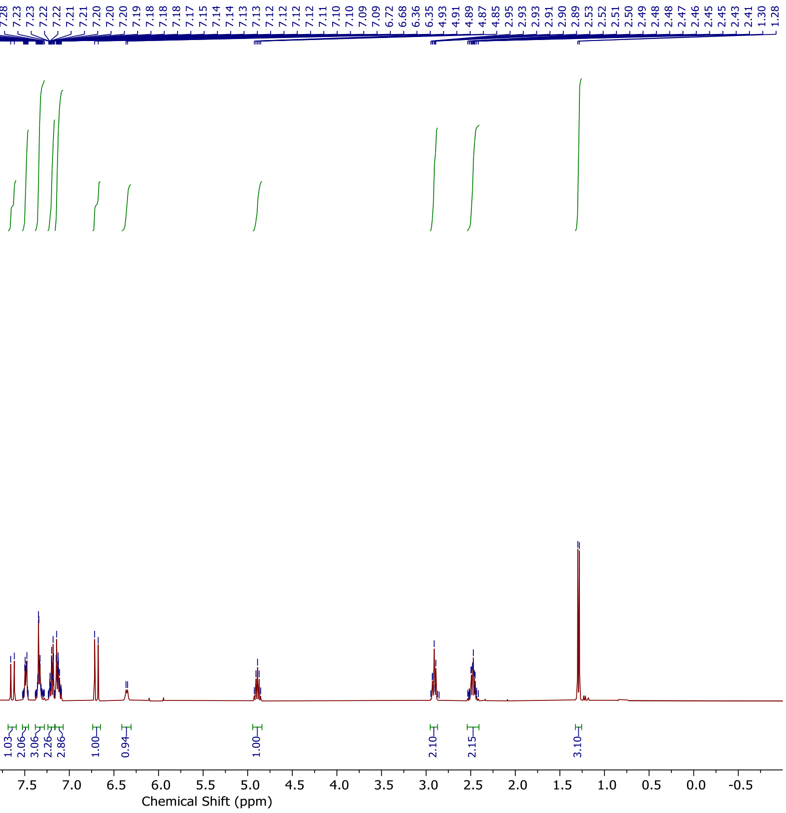

^{13}C-NMR (100 MHz, $CDCl_3$)

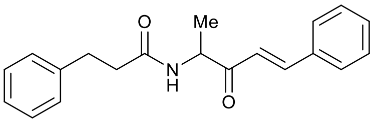

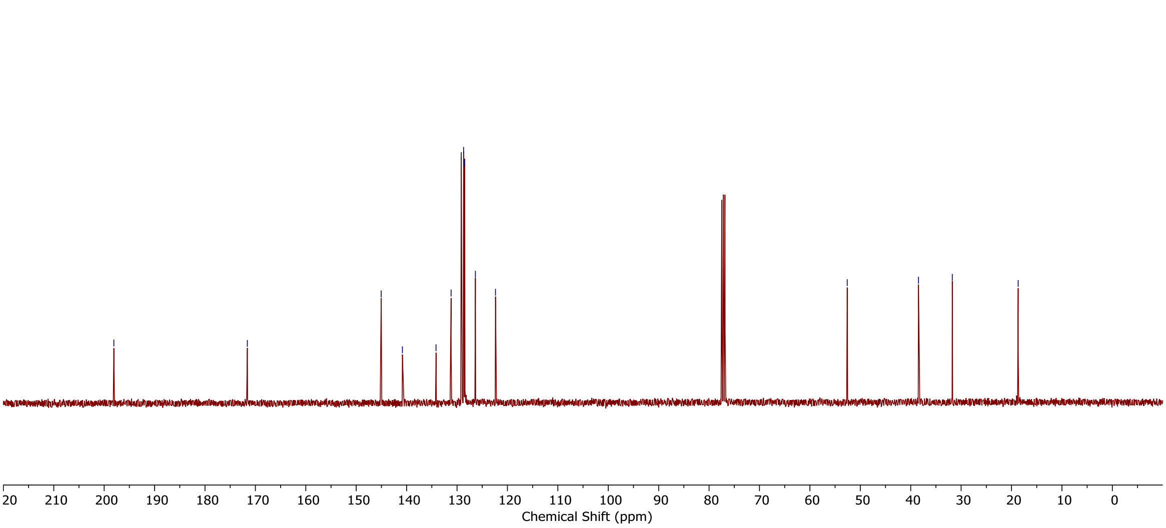

(*E*)-*N*-(6-methyl-3-oxo-1-phenylhept-1-en-4-yl)-3-phenylpropanamide, 2y

^{1}H-NMR (400 MHz, $CDCl_3$)

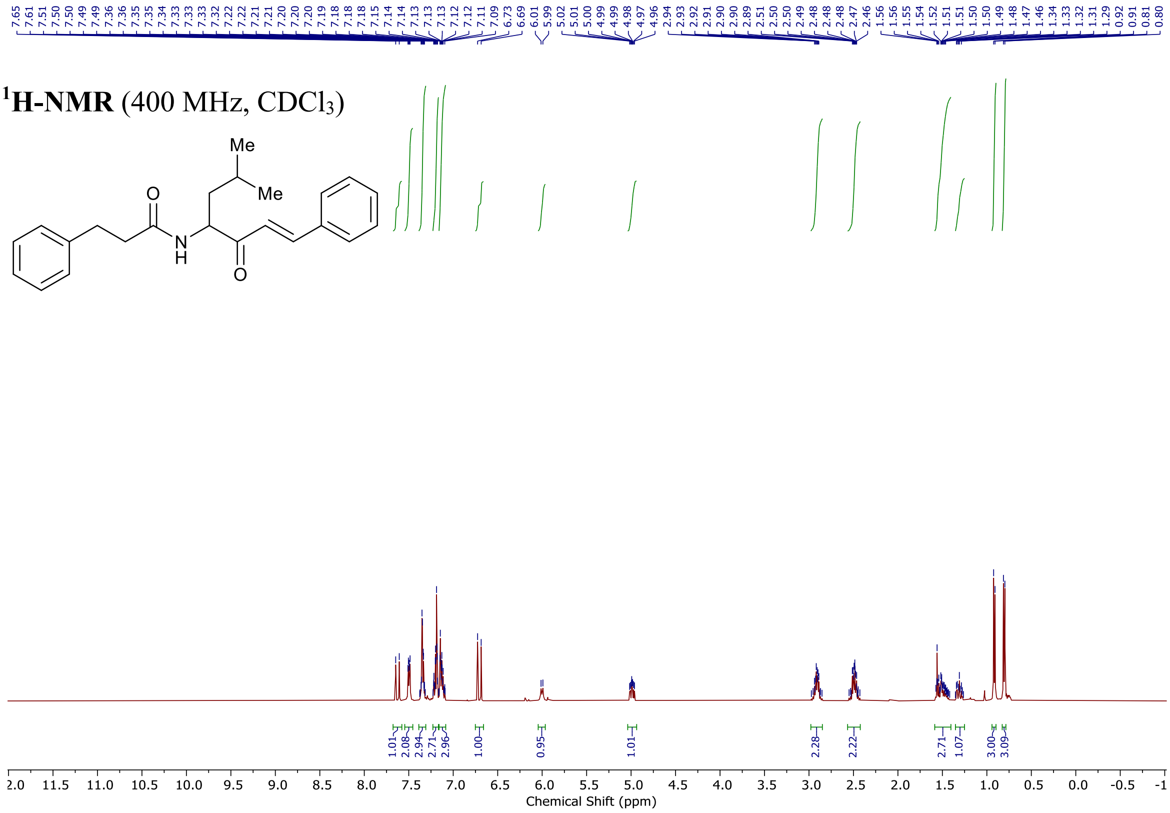

^{13}C-NMR (100 MHz, $CDCl_3$)

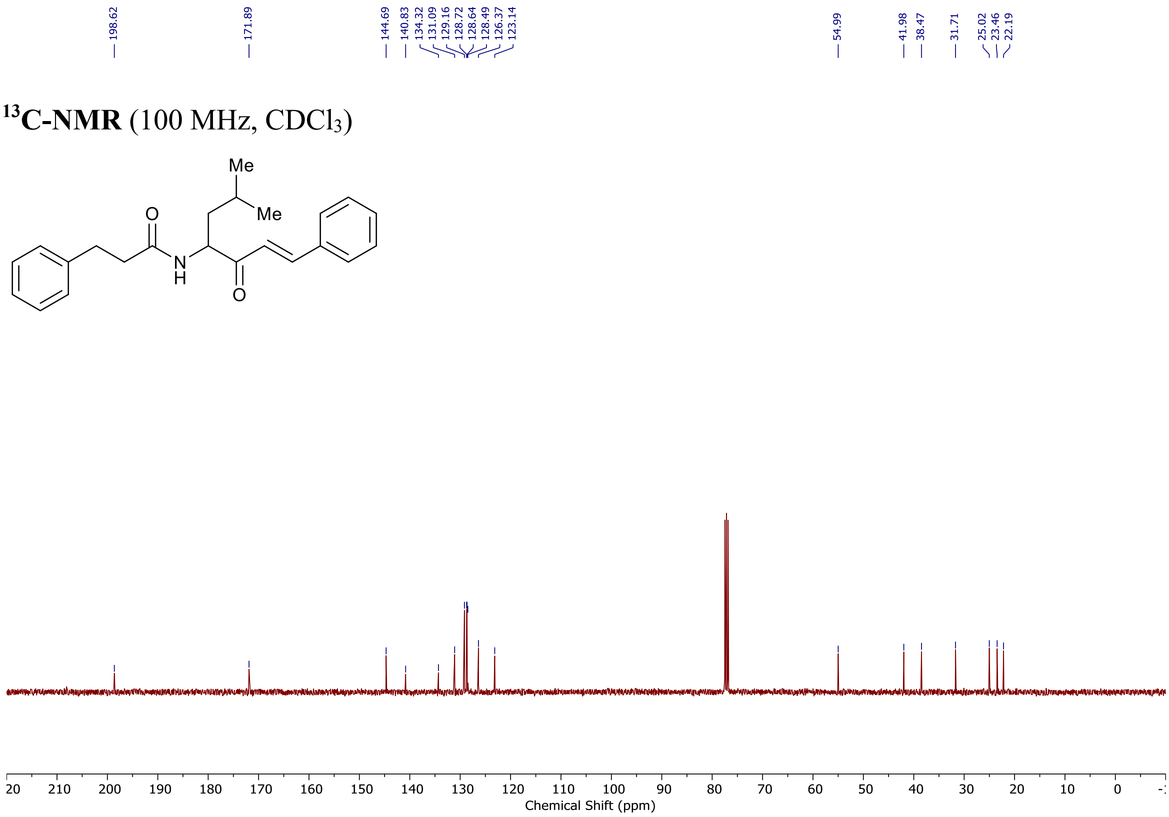

(*E*)-*N*-(2-methyl-4-oxo-6-phenylhex-5-en-3-yl)-3-phenylpropanamide, 2z

^{1}H-NMR (400 MHz, $CDCl_3$)

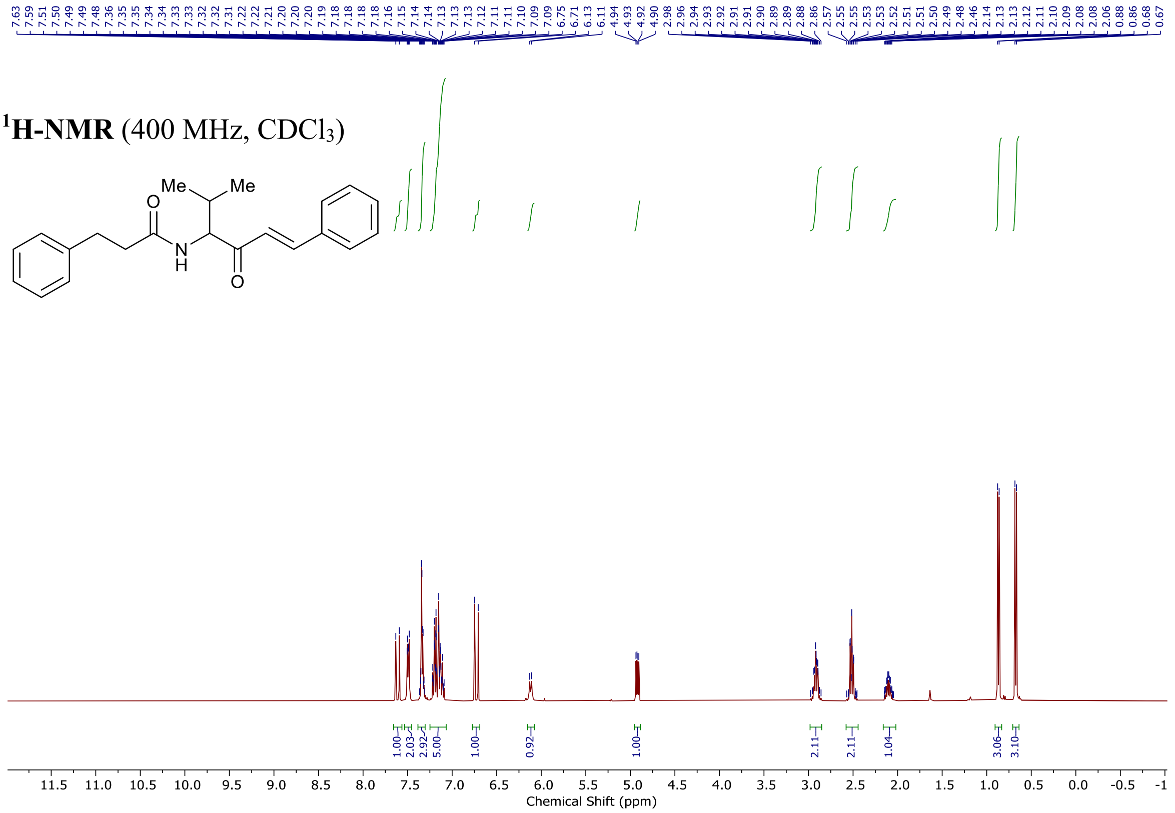

^{13}C-NMR (100 MHz, $CDCl_3$)

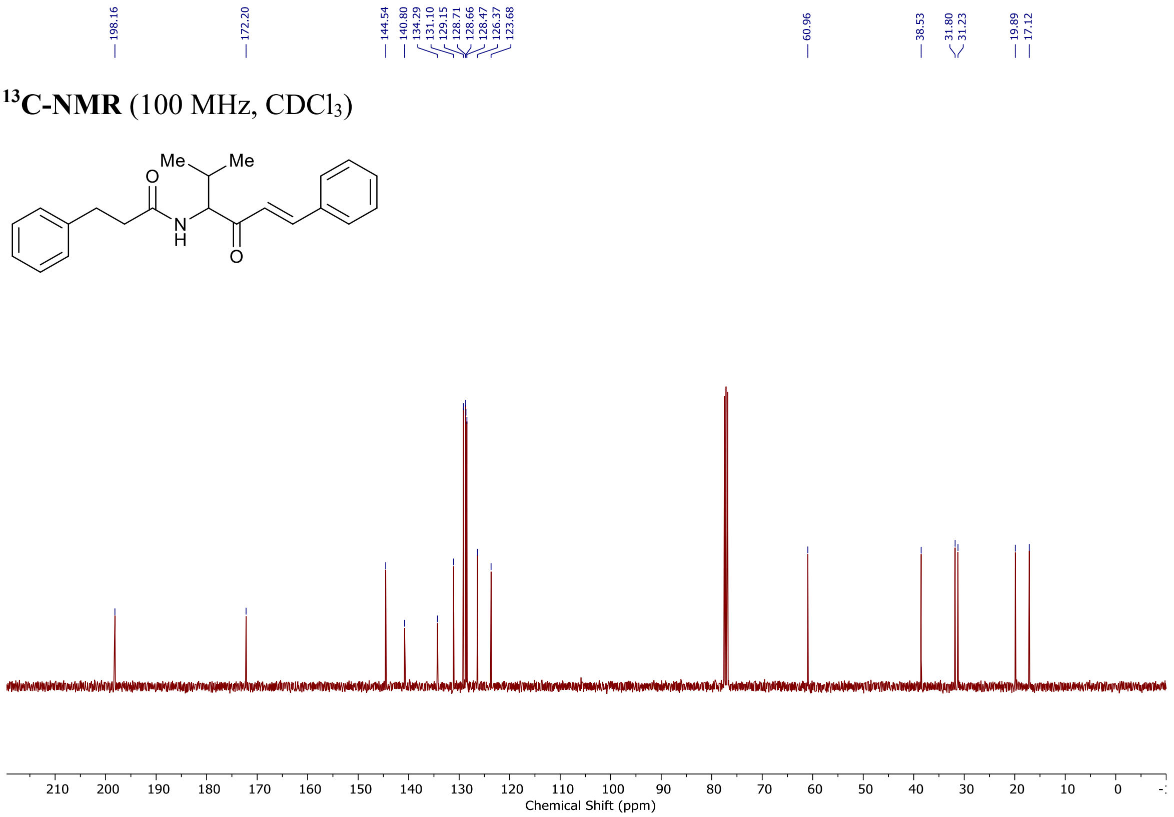

(*E*)-*N*-benzyl-*N*-(2-oxo-4-phenylbut-3-en-1-yl)benzamide, 2aa

^{1}H-NMR (400 MHz, $CDCl_3$)

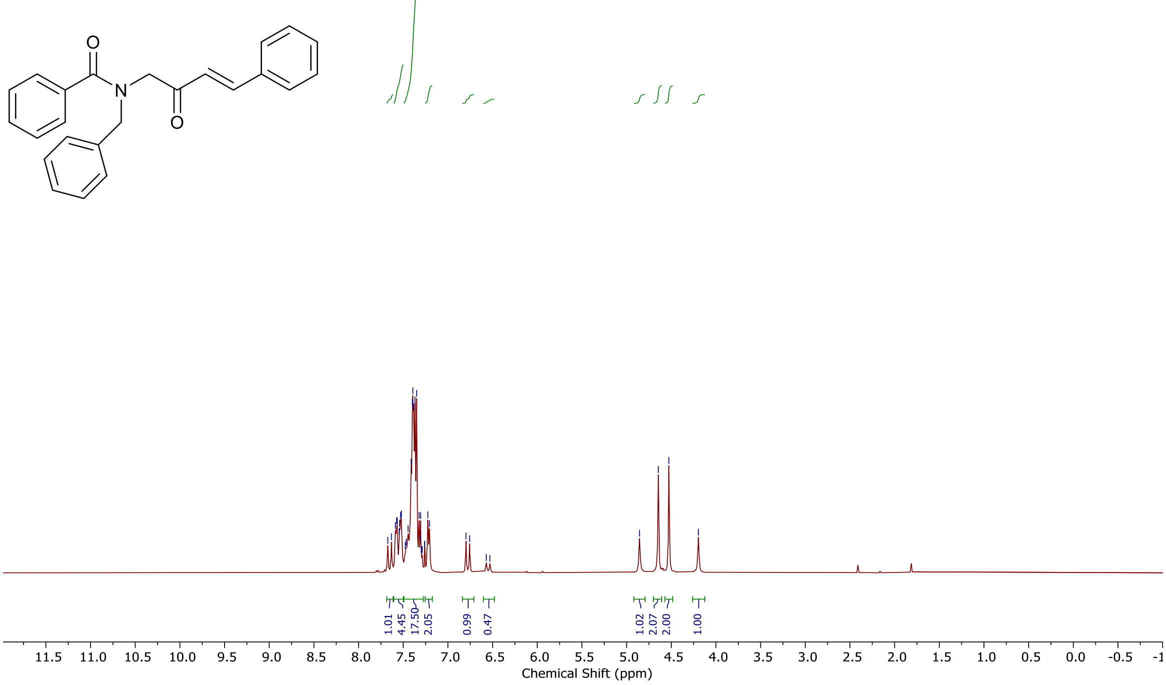

^{13}C-NMR (100 MHz, $CDCl_3$)

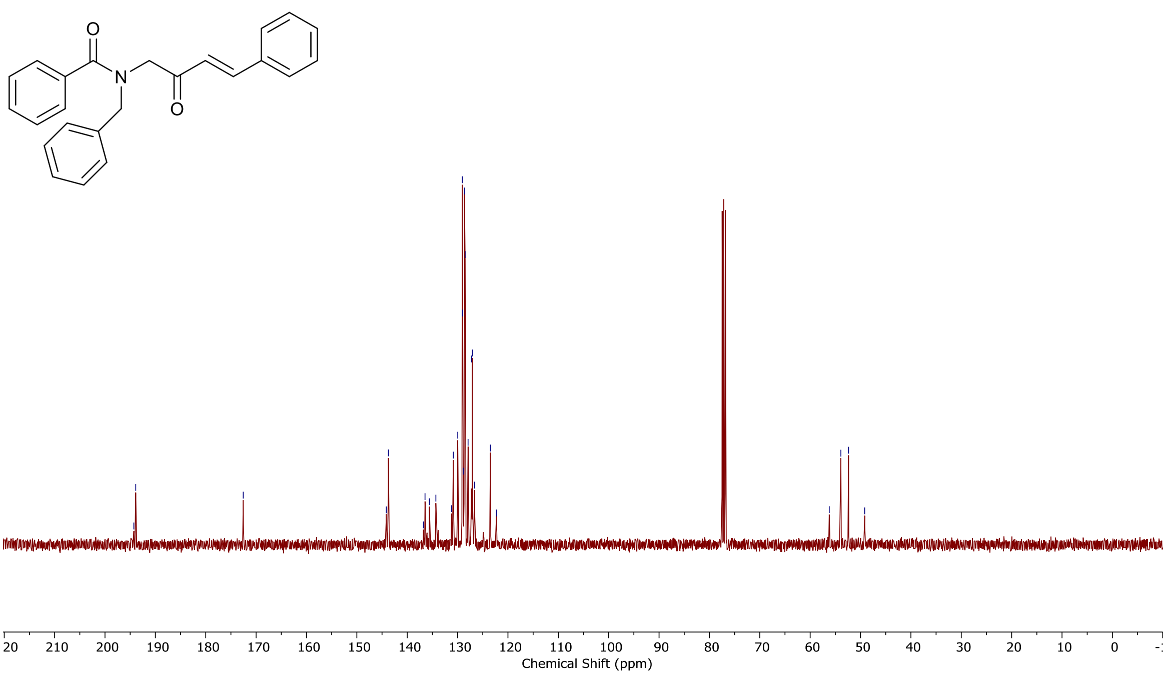

(*E*)-*N*-benzyl-*N*-(2-oxo-4-phenylbut-3-en-1-yl)acetamide, 2ab

^{1}H-NMR (400 MHz, $CDCl_3$)

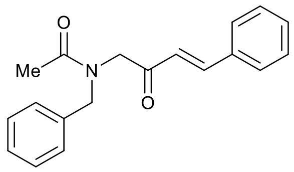

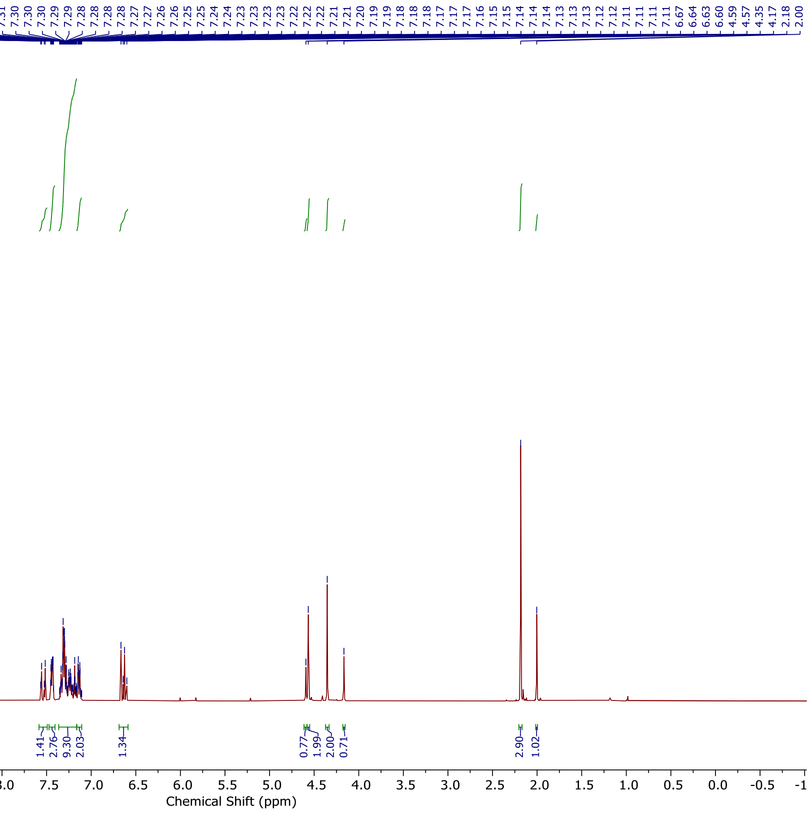

^{13}C-NMR (100 MHz, $CDCl_3$)

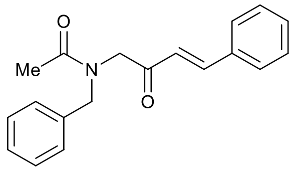

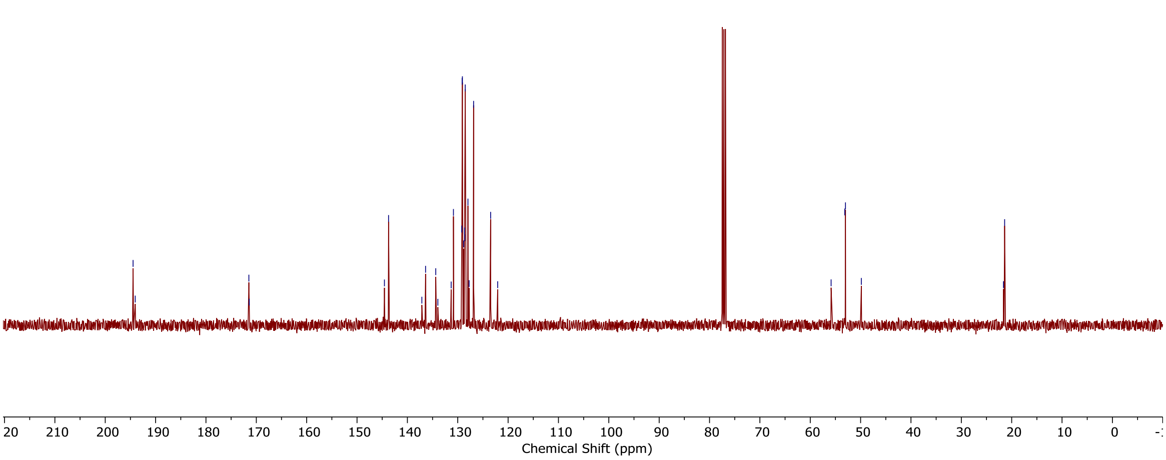

Benzyl (*E*)-benzyl(2-oxo-4-phenylbut-3-en-1-yl)carbamate, 2ac

^{1}H-NMR (400 MHz, $CDCl_3$)

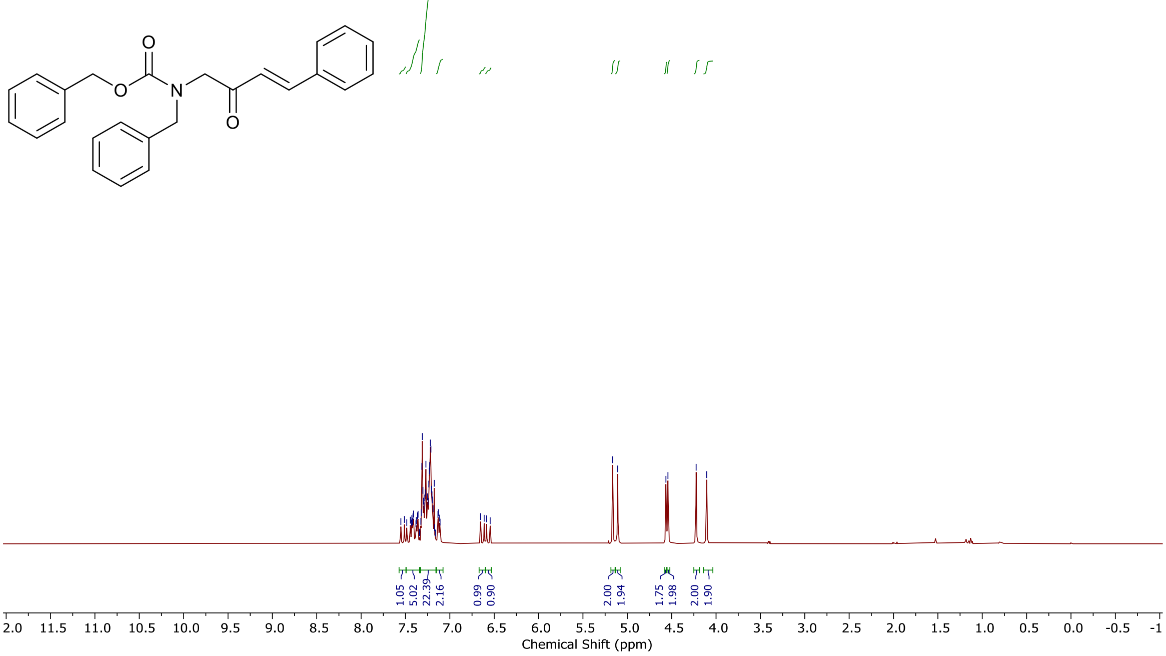

^{13}C-NMR (100 MHz, $CDCl_3$)

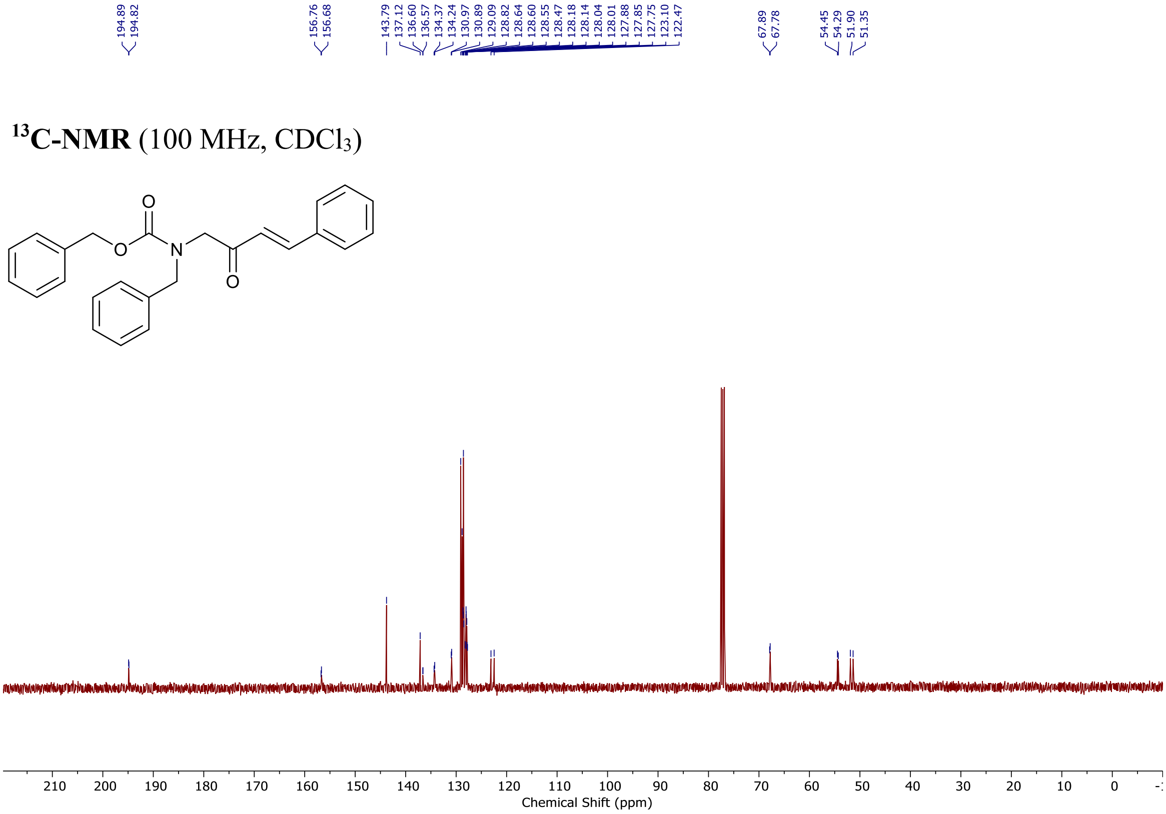

(*E*)-*N*-benzyl-*N*-(2-oxo-4-phenylbut-3-en-1-yl)cyclohexanecarboxamide, 2ad

^{1}H-NMR (400 MHz, $CDCl_3$)

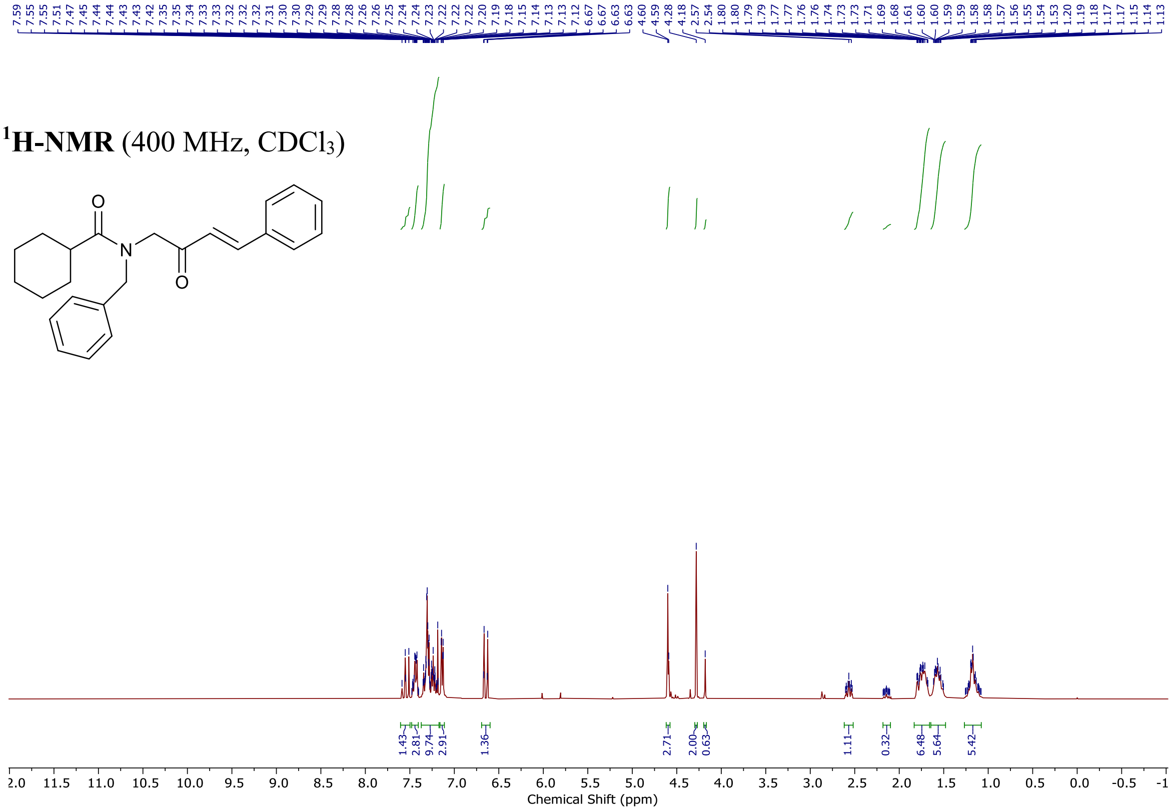

^{13}C-NMR (100 MHz, $CDCl_3$)

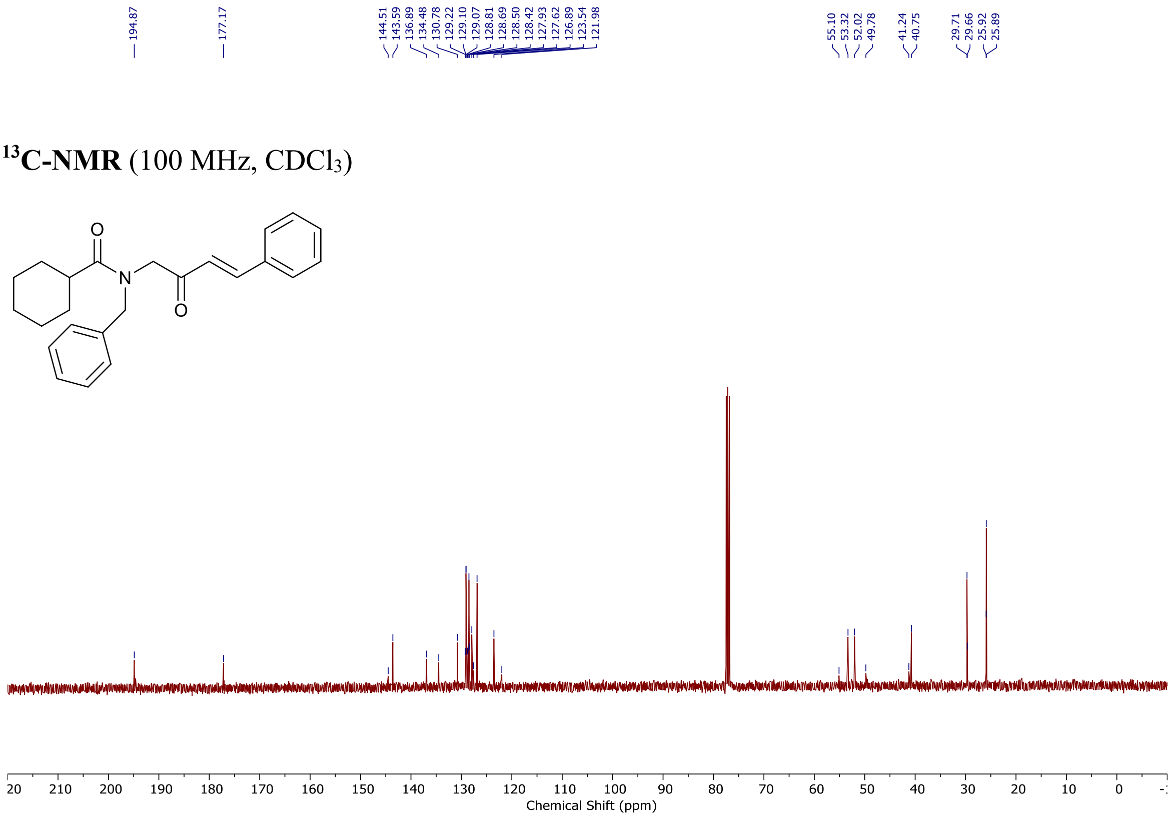

(*E*)-*N*-(3-oxo-1,5-diphenylpent-4-en-2-yl)cyclohexanecarboxamide, 2ae

^{1}H-NMR (400 MHz, $CDCl_3$)

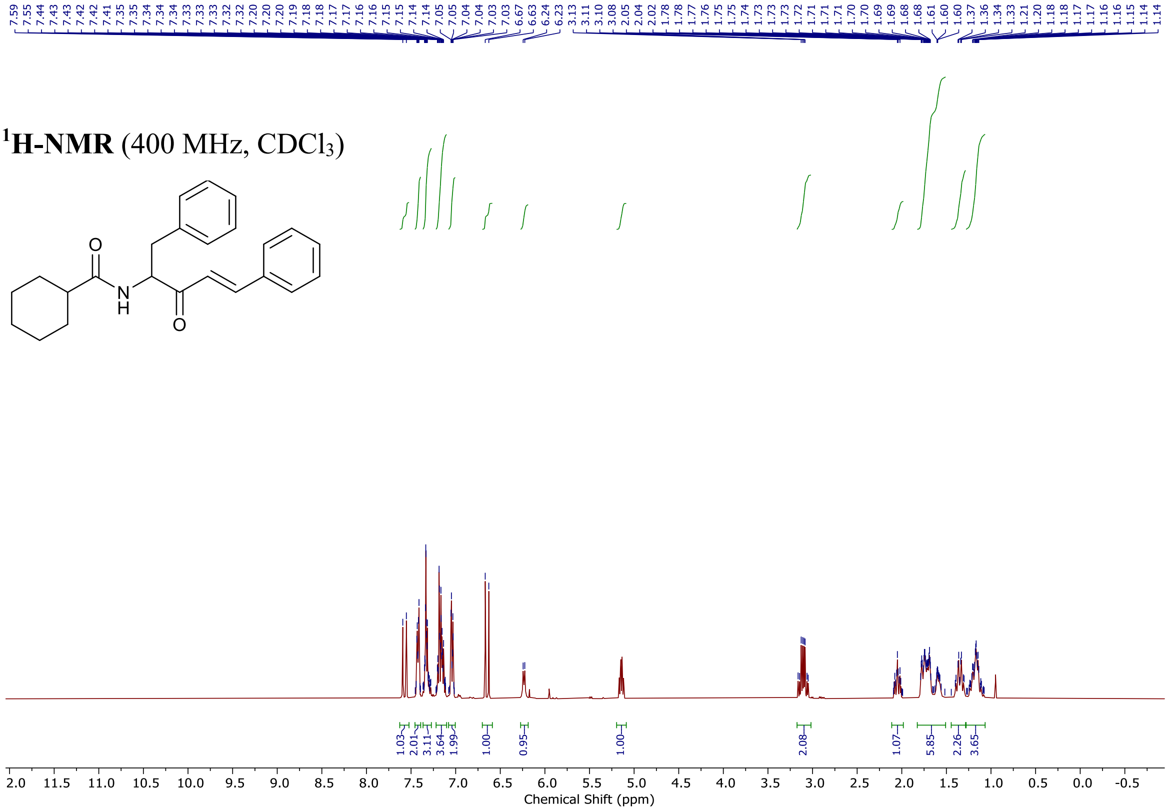

^{13}C-NMR (100 MHz, $CDCl_3$)

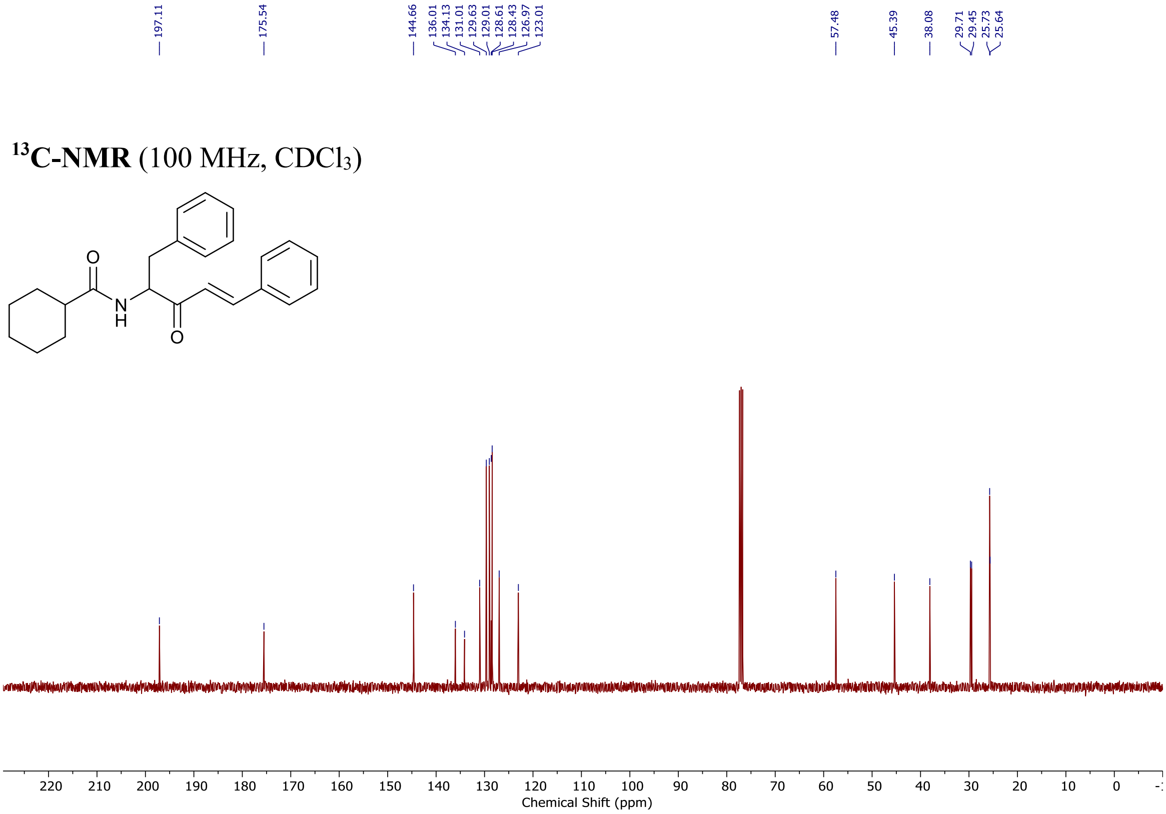

(*E*)-4-benzyl-2-phenethyl-5-styryloxazole, 3

^{1}H-NMR (400 MHz, $CDCl_3$)

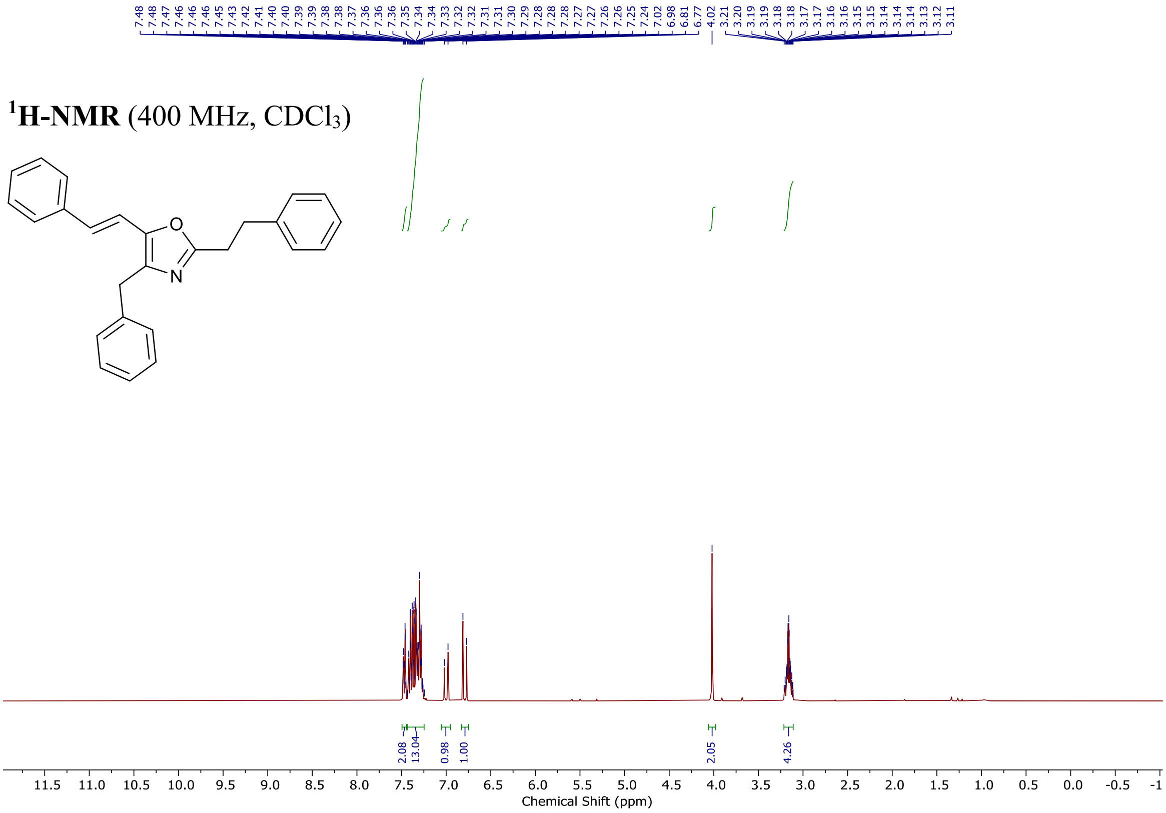

^{13}C-NMR (100 MHz, $CDCl_3$)

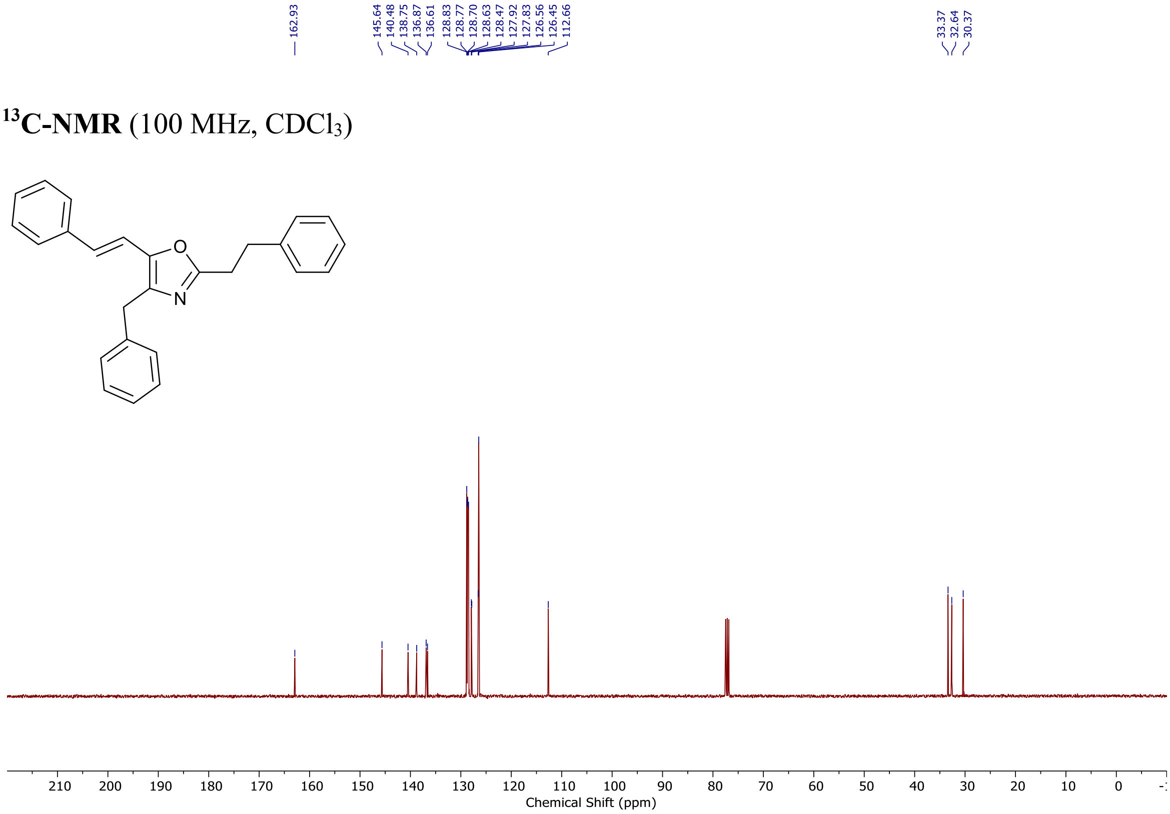

Supplement: Supplementary file 1 — Supplementary [file CHEM-26-11710-s001.pdf]
